# Early Chronic Fluoxetine Treatment of Ts65Dn Mice Rescues Synaptic Vesicular Deficits and Prevents Aberrant Proteomic Alterations

**DOI:** 10.3390/genes15040452

**Published:** 2024-04-03

**Authors:** S. Hossein Fatemi, Elysabeth D. Otte, Timothy D. Folsom, Arthur C. Eschenlauer, Randall J. Roper, Justin W. Aman, Paul D. Thuras

**Affiliations:** 1 Department of Psychiatry and Behavioral Sciences, University of Minnesota Medical School, Minneapolis, MN 55455, USA; aman52495@gmail.com; 2 Department of Biology, Indiana University, Indianapolis, IN 46202, USA; edallman@indiana.edu; 3 Department of Pediatrics, University of Minnesota Medical School, Minneapolis, MN 55455, USA; folso013@umn.edu; 4 Minnesota Supercomputing Institute, University of Minnesota, Minneapolis, MN 55455, USA; esch0041@umn.edu; 5 Department of Biology, Indiana University-Purdue University, Indianapolis, IN 46202, USA; rjroper@iu.edu; 6 Department of Psychiatry and Behavioral Sciences, University of Minnesota Medical School and VA Health Care System, One Veterans Drive, Minneapolis, MN 55417, USA

**Keywords:** Fluoxetine, Ts65Dn, vesicular traffic, proteomics, Down syndrome

## Abstract

Down syndrome (DS) is the most common form of inherited intellectual disability caused by trisomy of chromosome 21, presenting with intellectual impairment, craniofacial abnormalities, cardiac defects, and gastrointestinal disorders. The Ts65Dn mouse model replicates many abnormalities of DS. We hypothesized that investigation of the cerebral cortex of fluoxetine-treated trisomic mice may provide proteomic signatures that identify therapeutic targets for DS. Subcellular fractionation of synaptosomes from cerebral cortices of age- and brain-area-matched samples from fluoxetine-treated vs. water-treated trisomic and euploid male mice were subjected to HPLC-tandem mass spectrometry. Analysis of the data revealed enrichment of trisomic risk genes that participate in regulation of synaptic vesicular traffic, pre-synaptic and post-synaptic development, and mitochondrial energy pathways during early brain development. Proteomic analysis of trisomic synaptic fractions revealed significant downregulation of proteins involved in synaptic vesicular traffic, including vesicular endocytosis (CLTA, CLTB, CLTC), synaptic assembly and maturation (EXOC1, EXOC3, EXOC8), anterograde axonal transport (EXOC1), neurotransmitter transport to PSD (SACM1L), endosomal-lysosomal acidification (ROGDI, DMXL2), and synaptic signaling (NRXN1, HIP1, ITSN1, YWHAG). Additionally, trisomic proteomes revealed upregulation of several trafficking proteins, involved in vesicular exocytosis (Rab5B), synapse elimination (UBE3A), scission of endocytosis (DBN1), transport of ER in dendritic spines (MYO5A), presynaptic activity-dependent bulk endocytosis (FMR1), and NMDA receptor activity (GRIN2A). Chronic fluoxetine treatment of Ts65Dn mice rescued synaptic vesicular abnormalities and prevented abnormal proteomic changes in adult Ts65Dn mice, pointing to therapeutic targets for potential treatment of DS.

## 1. Introduction

Down syndrome (DS) is one of the most common forms of inherited intellectual disability, which is caused by trisomy of chromosome 21 (Ts21) [1,2]. DS is primarily characterized by varying degrees of intellectual impairment accompanied with craniofacial abnormalities [3], congenital cardiac defects [4], and gastrointestinal disorders [5]. Other common medical comorbidities include obesity, sleep apnea, and seizure disorder [6,7]. Currently, the incidence of DS in newborns is 1 in 737 in the United States [8] and 1 in 1000 live births worldwide [9]. DS can be classified as both a neurodevelopmental disorder due to pathological changes in the brain that occur during fetal and neonatal development [10] as well as a neurodegenerative disorder, as 2–5% of DS individuals display symptoms of Alzheimer’s disease (AD) and dementia by age 40, with that percentage approaching 100% by age 70 [11].

Multiple gross brain abnormalities have been observed in individuals with DS including reduced overall brain volume as well as reduced volume of the hippocampus, frontal and temporal lobes, cerebellum, and brainstem [12,13]. Moreover, reduced neurogenesis is one of the main neurodevelopmental deficits which may underlie intellectual disability in individuals with DS [13]. Hippocampal and para-hippocampal tissues from Ts21 fetuses have shown reduced number of neurons and increased numbers of astrocytes [14]. Brain abnormalities associated with AD, including amyloid plaques, neurofibrillary tangles, and neuronal cell loss are common in individuals with DS over the age of 40 [15,16].

Mouse models have been developed to study the impact of trisomy on brain development and phenotypes associated with DS as well as serve as preclinical models. The Ts65Dn model was the first model developed [17] and is still widely used [2]. This model replicates many abnormalities of DS including delayed acquisition of motor skills, impairments in context discrimination, spatial and learning memory, working and reference memory, and motor coordination [18,19,20,21,22,23,24].

Morphologically, this animal model also displays reduced brain volume, reduced neuronal density, reduced cerebellar volume, impaired neurogenesis, reduced dendritic density, and endosomal abnormalities similar to brain abnormalities in individuals with DS [18,19,20,21,22,23,24,25]. Proteomic studies have provided some insight into changes in protein expression in this mouse model [26,27,28,29]. An initial study of synaptic junctions of Ts65Dn mice [26] found minimal changes in levels of synaptic proteins and in their phosphorylation. A more recent study of subsynaptic compartments found significant changes in kinases and phosphatases as well as phosphorylation of ionotropic glutamate receptors (iGluRs) in the hippocampi of Ts65Dn mice [27]. A study that compared the hippocampal and cerebellar proteomes of Ts65Dn mice identified differential expression of 272 proteins [29]. Moreover, 132 genes were differentially expressed between the two age groups (6- and 12 months of age) and 141 were differentially expressed between the two brain regions [29].

Several preclinical studies involving Ts65Dn mice suggest that treatment with fluoxetine can reverse anatomical, electrophysiological, and cognitive impairments in this model. Treatment of neonatal mice [beginning at postnatal day 3 (P3)] with fluoxetine for 24 days restored neurogenesis in the dentate gyrus, subgranular and granular layers of the hippocampus, the subventricular zone, striatum, and neocortex [19,20]. Further studies found that early treatment (P3–P15) with fluoxetine improved dendritic arborization in the dentate gyrus and restored synaptic connectivity between the dentate gyrus and CA3 pyramidal neurons as indicated by increased frequency of miniature excitatory postsynaptic currents (mEPSCs) and miniature inhibitory postsynaptic currents (mIPSCs) [21,22]. Moreover, treatment with fluoxetine for eight weeks beginning at P60 resulted in normalized GABA release and synaptic plasticity in the hippocampus, and improved spatial working memory in Ts65Dn mice [18]. The increased dendritic arborization, hippocampal neurogenesis, and memory changes were found to persist in adult Ts65Dn mice that were treated with fluoxetine as neonates [23]. These changes can also occur when pregnant Ts65Dn mice are treated with fluoxetine in their drinking water (from embryonic day 10 (E10) through to delivery) rather than direct treatment of pups, and that these changes have been found to persist into adulthood [30]. In contrast, one study found that adult-onset fluoxetine treatment had negative outcomes, including seizures and increased mortality of Ts65Dn mice [31]. A case study also indicated presence of seizures in a patient with DS following fluoxetine treatment [32]. Finally, Trazzi et al. [33] found that fluoxetine treatment increased phosphorylation of glycogen synthase kinase 3-β (GSK3β) leading to reduction in its activity. This suggests that this pathway may be important in fluoxetine’s efficacy in this model. GSK3β is a negative regulator of neurogenesis, neuronal differentiation, and neuronal migration, [34], all of which are impaired in DS. The authors suggest that drugs like fluoxetine and lithium, which increase GSK3β phosphorylation, thus reducing its activity, may improve neurogenesis in subjects with DS [33]. Recently, work by Zhu et al. [2] implicated inhibition of the protein kinase RNA-activated (PKR) branch of the integrated stress response (ISR) as a mechanistic explanation for fluoxetine therapeutic effect in DS [34]. Emerging evidence in support of intracellular trafficking abnormalities in DS prompted us to test three hypotheses: (1) Can we identify proteomic biomarkers responsible for trafficking dysfunction in trisomic mice? (2) Would fluoxetine, a prototypical selective serotonin reuptake inhibitor with established ability to enhance vesicular trafficking, correct the trisomic intracellular deficits? (3) Would chronic administration of fluoxetine during a critical period of brain development and prior to full brain and sexual maturation correct trisomic brain dysfunction? Thus, we carried out subcellular proteomics of the effects of fluoxetine in Ts65Dn mice to further identify proteins that may be altered secondary to effects of this important agent, providing us with insights on how fluoxetine may help individuals with DS.

## 2. Materials and Methods

### 2.1. Animals

Female B6EiC3Sn a/A-Ts(17^16^)65Dn (Ts65Dn strain #001924) mice were obtained from Jackson Laboratories (Bar Harbor, ME) and then bred with B6C3F_1_ males in-colony. Offspring from (Ts65Dn x B6C3F1) mating were genotyped with polymerase chain reaction (PCR), using primer sequences “GTGGCAAGAGACTCAAATTCAAC” and “TGGCTTATTATTATCAGGGCATT”, that cross the breakpoint between chromosomes 17 and 16 of the small extra marker chromosome [35]. Male trisomic and euploid offspring were used for this study. Offspring were kept with their mothers until 3 weeks (21 days) of age, at which time the pups were weaned and separated by sex into cages containing 1–5 mice. All animals in this study were kept on a 12 h/12 h light-dark cycle. All mouse experiments were performed using mouse protocols approved by the Institutional Animal Care and Use Committee (IACUC) of the Indiana University-Purdue University Indianapolis School of Science.

### 2.2. Fluoxetine Treatment

Mice were housed individually and were supplied with nesting material and cardboard huts to deter agitation while housed individually. Preliminary testing was used to determine the average water intake per mouse per day to determine appropriate dosage. Seven euploid and five trisomic Ts65Dn mice were monitored from P28–P88 for daily water intake. The amount of water consumed between these initial mice did not vary significantly between subjects (Table S1A–F). 

At P28, male trisomic and euploid mice were age-matched and randomly assigned either water or 0.04 mg/mL of fluoxetine hydrochloride in the water (Sigma Aldrich, St. Louis, MO, USA, F132) for consumption ad libitum. Fluoxetine solution was prepared using water from the same source as the regular drinking water supplied to the mice. The mice were weighed weekly, and drug treatment was measured and replaced every 2 days. Fresh fluoxetine solution was prepared approximately every week, and kept at 4 °C when not in use. 

Fluoxetine or control water treatment was administered until 88 days of age, at which point the animals were euthanized by inhalation of isoflurane and cervical dislocation. Blood samples were collected via a cardiac puncture. The brains of animals were removed, and the cerebral cortices were dissected out and placed in labelled tubes, before being snap-frozen in a liquid nitrogen bath and stored at −80 °C. 

### 2.3. Subcellular Fractionation

Mouse cerebral samples (N = 4 per group) were subjected to homogenization following the techniques of Mueller et al. [36] and Taha et al. [37]. Brain samples were placed in 1 × isotonic extraction buffer (10 mM HEPES, 250 mM sucrose, 25 mM KCl, 1 mM EGTA, pH 7.8) plus protease inhibitors (at volume of 3 × the tissue’s weight) for 4 min on ice. Subsequently, the tissues were homogenized using a motorized pestle four times for 30 s each. A 60 µL aliquot of this was saved as total homogenate. A low-speed centrifugation (700 × *g*) (5415D centrifuge, Eppendorf North America, Hauppauge, NY, USA) for 10 min at 4 °C was conducted to remove intact nuclei and heavy membranes (pellet 1). Next, following a centrifugation at 15,000× *g* for 10 min at 4 °C, the supernatant (supernatant 1) was obtained and the crude membrane fraction isolated (pellet 2). Pellet 2 was reconstituted in a sucrose homogenization buffer and added to 3 mL of Triton X-100 buffer (10 mM Na3VO4, 5 mM NaF, 1 mM EDTA, 1 mM EGTA, 0.5% *v*/*v* Triton X-100, pH 7.4) in the presence of protease inhibitors and placed in ultracentrifuge tubes. A 30,000× *g* centrifugation step for 20 min (Optima L-90 K Ultracentrifuge, Beckman-Coulter, Indianapolis, IN, USA) resulted in a Triton-insoluble pellet (pellet 3) which represented the synaptic fraction. The synaptic fraction was reconstituted in 40 µL of PBS and protease inhibitors. Protein levels for each fraction were determined using the Bradford assay. In the final phase of the study, synaptic fractions for cortical tissues for all groups were subjected to proteomics.

### 2.4. Proteomic Methodology

#### 2.4.1. Protein Extraction and In-Solution Digestion

For each sample, a 20 µg aliquot was prepared by adding 80 µL of PBS extraction buffer (7 M Urea, 2 M Thiourea, 400 mM Tris pH 8, 10 mM TCEP, 40 mM Chloroacetamide, 20% Acetonitrile). The samples were sonicated at 30% amplitude for 7 s on ice with a Branson Digital Sonifier 250 (Branson Ultrasonics Corporation, Danbury, CT, USA). Samples were transferred to pressure cycling tubes for the Barocycler NEP2320 (Pressure Biosciences, Inc., South Easton, MA, USA) and barocycled between 35 kPSI for 20 s. and 0 kPSI for 10 s. for 60 cycles at 37 °C. All samples were transferred to new 1.5 mL microfuge Eppendorf Protein LoBind tubes and diluted fivefold with water, then treated with trypsin (Promega, Madison, WI, USA) in a 1:40 ratio of trypsin to total protein. Samples were then incubated overnight for 16 h at 37 °C.

#### 2.4.2. TMTpro 16plex^TM^ Isobaric Labeling

The 20 µg of digested peptides were lyophilized and cleaned with a 1 cc Oasis MCX cartridge (Waters Corporation, Milford, MA, USA). Eluates were lyophilized and resuspended with 0.1 M triethylammonium bicarbonate, pH 8.5, to a final concentration of 1 µg/µL. A 19 µL aliquot of each sample was made, and all aliquots were then brought to 25 µL with 0.1 M TEAB, pH 8.5. TMTpro 16plex™ Isobaric Label Reagent, 0.5 mg (Thermo Scientific, Watham, MA, USA), was resuspended in 70 µL of anhydrous acetonitrile. In total, 25 µL of sample and 20 µL of TMTpro 16plex™ reagent were mixed for each channel. Labeled peptides from each experiment were multiplexed together and lyophilized. The labeled multiplexed samples for each experiment were cleaned with a 1 cc C18 Sep-Pak cartridge (Waters Corporation, Milford, MA, USA) and lyophilized.

#### 2.4.3. Peptide Liquid Chromatography Fractionation

Samples were resuspended in 50 µL of 50 mM ammonium formate and fractionated offline with high pH C18 reversed phase (RP) chromatography [38] with the following changes. A Shimadzu Prominence HPLC (Shimadzu, Columbia, MD, USA) with a Hot Sleeve-25 L Column Heater (Analytical Sales & Products, Inc., Pompton Plains, NJ, USA) was used with a Security Guard precolumn housing a Gemini NX C18 cartridge (Phenomemex, Torrance, CA, USA) attached to a C18 XBridge column, 150 mm (column length) × 2.1 mm (internal diameter), 5 µm particle size (Waters Corporation, Milford, MA, USA). Buffer A was 20 mM ammonium formate, pH 10 in 98:2 water:acetonitrile, and Buffer B was 20 mM ammonium formate, pH 10 in 10:90 water:acetonitrile. The flow rate was 200 µL/min with a gradient from 2–7% buffer B over 0.5 min, 7–15% buffer B over 7.5 min, 15–35% buffer B over 45 min, and 35–60% buffer B over 15 min. Fractions were collected every 2 min, and UV absorbances were monitored at 215 nm and 280 nm. Peptide containing fractions were divided into two equally numbered groups, “early” and “late”. A volume equal to 15 milli-absorbance units of the first “early” fraction was concatenated with the first “late” fraction, and so on. Concatenated fractions were lyophilized and cleaned with Stop and Go Extraction Protocol (STAGE tip) using Waters Oasis MCX material.

#### 2.4.4. Orbitrap Eclipse Liquid-Chromatography–Mass-Spectrometry Analysis

The concatenated, dried peptide fractions were reconstituted in load solvent (94.9:5:0.1, H_2_O:acetonitrile (ACN):formic acid (FA)) and analyzed between 7.5 and 25% of each peptide pool by capillary LC–MS with a Thermo Fisher Scientific, Inc. (Waltham, MA, USA) Dionex UltiMate 3000 system in-line with Orbitrap Eclipse mass spectrometer equipped with High-Field Asymmetric Waveform Ion Mobility Spectrometry (FAIMS). Peptides were loaded directly on-column in load solvent. Peptides were separated on a self-packed c18 column (Dr. Maisch GmbH ReproSil-PUR; Beim Brückle 14, 72119 Ammerbuch-Endringen, Germany) 1.9 µm, 120 A C18aq, 100 µm ID × 30 cm length at 55 °C with a biphasic gradient starting at 5% solvent B at a flow rate of 325 nL/min. The starting conditions were held for 2 min and increased to 8% solvent B by 2.5 min. The flow was reduced to 315 nL/minute and the gradient increased to 21% B at 135 min and 34% B by 180 min. Finally, the gradient was increased to 90% B by 182 min with a flowrate of 325 nL/min and held to 188 min followed by a return to starting conditions at 5% B at 190 min and held to 197 min. The solvent A composition was 0.1% FA in H_2_O and solvent B composition was 0.1% FA in ACN. The FAIMS total carrier gas flow was 4.6 L/min, the cooling gas flow was 5.0 L/min, and the inner and outer electrodes were set to 100 °C. We scanned the CVs (compensation voltages) at −45, −60, and −75, and performed data-dependent tandem MS2 with a 1 s cycle time per CV using the following parameters: ESI voltage 2.1 kV, ion transfer tube 275 °C; Orbitrap MS1 scan 120k resolution in profile mode from 400–1400 *m*/*z* with 50 msec maximum injection time (IT), 100% (4 × 10^5^) automatic gain control (AGC); minimum MS2 trigger intensity 2.5 × 10^4^ counts; 0.7 Da quadrupole isolation window; fixed HCD activation with 38% collision energy; Orbitrap detection with 50K resolution at 200 *m*/*z*; first mass fixed at 110 *m*/*z*; 150 msec max injection time; and 250% (1.25 × 10^5^) AGC and 10 s dynamic exclusion (DE) duration with +/− 10 ppm mass tolerance. Lock mass was not employed for internal calibration. MS2 was triggered on a single charge state per precursor, and the DE was not shared among FAIMS CVs.

#### 2.4.5. Database Search

Peptide tandem MS were processed using Sequest (Thermo Fisher Scientific, San Jose, CA, USA, in Proteome Discoverer 2.5). The mouse (taxonlD 10090) Universal Proteome (UP000000589) target protein sequence database was downloaded from UniProt (www.uniprot.org/ accessed on 6 June 2023) and merged with a common lab contaminant protein database (http://www.thegpm.org/CRAP/index.html accessed on 6 June 2023); there was a total of 55,498 protein sequences. For the digestion enzyme trypsin, the fragment ion mass tolerance was 0.05 Da and the precursor tolerance was 15 ppm. We set the variable modifications for oxidation of methionine, pyroglutamic acid conversion from glutamine, deamidation of asparagine, acetyl and/or met-loss of the protein N-terminus, and TMT16plex of lysine and peptide N-terminus. We specified carbamidomethyl of cysteine as a fixed modification.

#### 2.4.6. Criteria for Protein Identification

Scaffold Q+ (version 5.1.2, Proteome Software Inc., Portland, OR, USA) was used for validation of tandem MS-based peptide and protein identifications. Peptide identifications were accepted if they could be established at greater than 92.0% probability to achieve a false discovery rate (FDR) less than 1.0% using the Percolator posterior error probability calculation [39]. Protein identifications were accepted if they could be established at greater than 6.0% probability to achieve an FDR less than 1.0% and contained at least 2 identified peptides. Protein probabilities were assigned by the Protein Prophet algorithm [40]. Proteins that contained similar peptides and could not be differentiated based on MS/MS analysis alone were grouped to satisfy the principles of parsimony. Proteins sharing significant peptide evidence were grouped into clusters. 

#### 2.4.7. Protein Quantification in Scaffold

Scaffold Q+ (version 5.1.2, Proteome Software Inc., Portland, OR, USA) was used for TMT-based peptide and protein quantification. Reporter ion intensities were adjusted by correction factors in all samples according to the algorithm described in i-Tracker [41] (according to the TMTpro 16plex Lot Number WF324548 product data sheet from ThermoFisher Scientific, Waltham, MA, USA). Normalization was performed iteratively (across samples and spectra) on intensities, as described in the Statistical Analysis of Relative Labeled Mass Spectrometry Data from Complex Samples using ANOVA [42]. Medians were used for averaging. Spectra data were log-transformed, pruned of those matched to multiple proteins, and weighted by an adaptive intensity weighting algorithm. Differentially expressed proteins were determined by applying a permutation with unadjusted significance level *p* < 0.05 corrected using the Benjamini–Hochberg procedure [43]. 

#### 2.4.8. Linear Modeling and Multivariate Analysis

The “Protein Quantitation Report” XML was exported from Scaffold, converted to JSON format using the “dataknead” program (https://github.com/hay/dataknead accessed on 6 June 2023), and imported into R using the “jsonlite” package (https://cran.r-project.org/web/packages/jsonlite accessed on 6 June 2023), resulting in a “data matrix” relating each log_2_-transformed quantity to its protein ID, sample, and treatment. Intensities for 2554 proteins were retrieved from the Scaffold 5 file.

Quantitative data were fitted to a linear model to assess simultaneously significance of condition (trisomy vs. euploid), treatment (fluoxetine vs. water), and the interaction between condition and treatment.

Principal Components Analysis (PCA) was performed using the BioConductor “ropls” R package (https://doi.org/10.18129/B9.bioc.ropls accessed on 6 June 2023), retaining seven principal components (PCs) that accounted for about 76% of the variance in the data model. Each of these seven PC components was tested for significant fit to the linear model, and randomized data were used to estimate the FDR. PC 3 was found to be significant for trisomy genotype, for fluoxetine treatment, and for the interaction between the two; PC 4 was significant only for fluoxetine treatment; and the FDR was estimated to be less than 0.3%, based on results of simulations applying randomization to the data matrix.

To characterize the proteins affected by fluoxetine in the trisomic genetic background, Gene Set Enrichment Analysis (GSEA) was performed using the 2554 proteins, ranked by their loadings for PC 3, using Reactome pathways as the gene sets. The normalized enrichment score was computed for each enriched Reactome pathway, and BH-adjustment of the *p*-value was performed for FDR control.

## 3. Results

The daily fluoxetine dose consumed by euploid mice (mean ± SD, 8.9 ± 1.35 mg/kg/day) did not vary significantly from trisomic mice (mean ± SD, 10.14 ± 1.71 mg/kg/day, *p* = 0.29; Supplementary Table S1A). The daily water intake for all euploid mice (mean ± SD, 6.13 ± 0.87 mL) did not vary significantly from all trisomic mice (mean ± SD, 5.63 ± 1.06 mL, *p* = 0.25, Table S1B). By the same token, cortical weights between euploid and trisomic fluoxetine and water-treated groups did not vary significantly (*p* = 0.64, *p* = 0.72, respectively, Table S1C,D). A two-way ANOVA also found no significant differences in cortical weight between euploid and trisomic mice, nor between mice in fluoxetine and water conditions. A two-way ANOVA was also used to examine the impact of fluoxetine vs. water and euploid vs. trisomy on body weights at P28 and P88. Comparison of body weights between fluoxetine-treated versus water-treated trisomic and euploid mice at either P28 or P88 did not show any significant differences (*p* = 0.31, p28; *p* = 0.32, p88, Table S1E). In contrast, body weights for euploid versus trisomic mice at P28 or P88 regardless of treatment condition showed statistically significant reductions in trisomic mice (F(1,18 = 18.2, *p* < 0.001 at P28) and F(1,18 = 11.5, *p* < 0.003 at P88)). The reduction in body weights for trisomic mice versus euploid mice has been observed previously, and likely represents phenotypic differences, as previously seen in Ts65Dn mice [44].

### 3.1. Multivariate Analysis of the Proteomic Data

**Figure 1:**
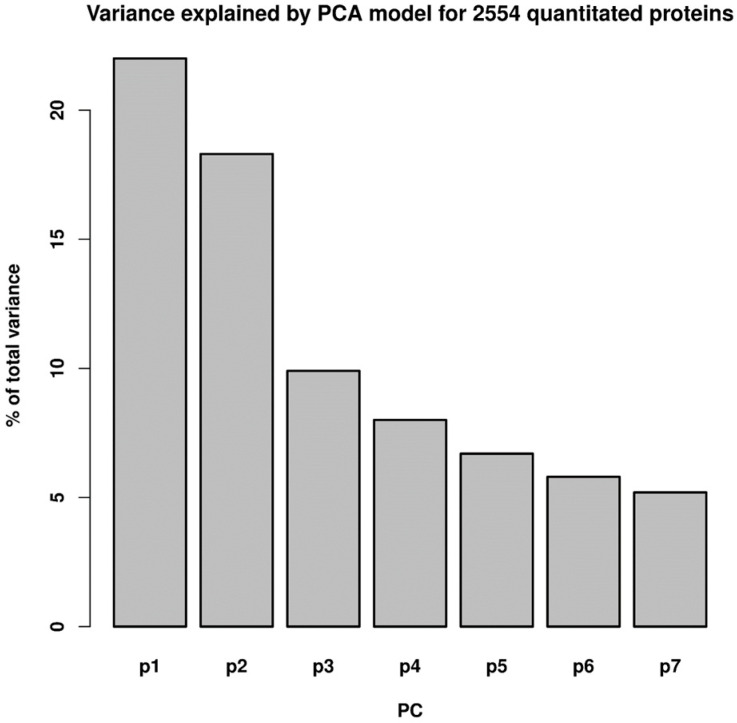
Variance explained by the first seven principal components from the PCA model for all 2554 quantitated proteins.

**Figure 2:**
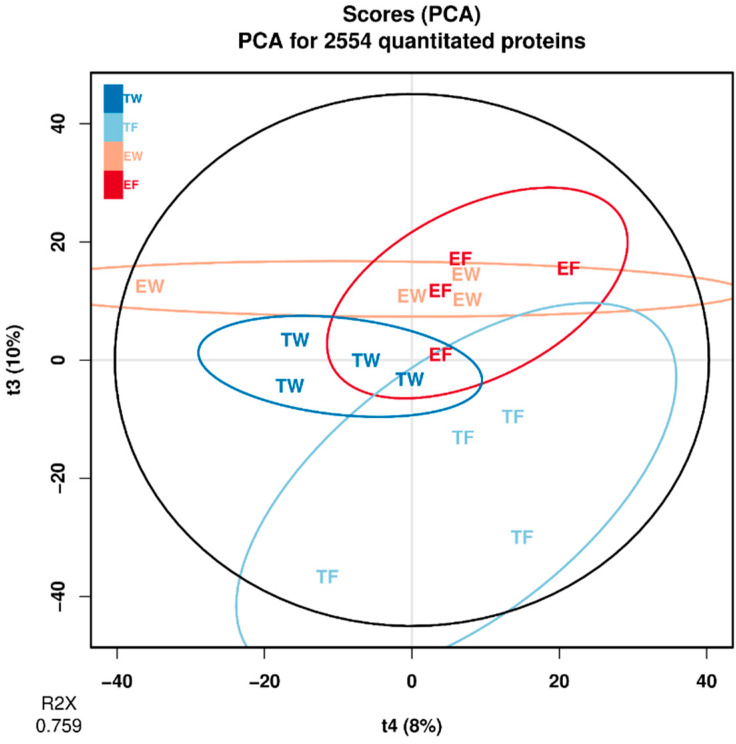
Scores for principal components 3 and 4 from PCA for all 2554 quantitated proteins. Point labels indicate treatment and animal genotype. The black oval represents 95% confidence region for all samples; the deep blue oval represents the trisomy-model animals treated with water (TW); the light blue oval represents trisomy with fluoxetine (TF); the light red oval represents euploid animals with water (EW); and the deep red oval represents euploid with fluoxetine (EF). For each sample, their position along the horizontal axis (“t4”) indicates the score for principal component 4; and along the vertical axis (“t3”), for PC3. Parenthesized percentages indicate the percentage of variation accounted for by the corresponding principal component. “R2X” indicates proportion of variation explained by all seven PCs.

Principal Components Analysis (PCA) was performed using the ropls package (obtained from BioConductor.org accessed on 6 June 2023). The seven principal components for the PCA model accounted for about 76% of the variance in the model (Figure 1). Each PC was tested for significant fit to the statistical model, and randomized data were used to estimate the FDR (false discovery rate). PC 3 was found to be significant for trisomy genotype, for fluoxetine treatment, and for the interaction between the two; PC 4 was significant only for fluoxetine treatment. The FDR was estimated to be less than 0.003 (0.3%), based on results of simulations applying randomization to the data matrix. A PCA scores plot of PCs 3 and 4 reveals the greater degree of effect that fluoxetine treatment has on the identified synaptic proteins in trisomic mice relative to euploid mice (Figure 2). There is little separation in the PC 3 × PC 4 space for the euploid treatments; trisomy + fluoxetine and trisomy + water samples both cluster fairly separately from the others, suggesting that fluoxetine’s effect in a trisomy background is not a simple reversal of the effect of trisomy + water relative to euploid + water, and that fluoxetine has a comparatively lesser effect in this space on euploid samples. 

**Figure 3:**
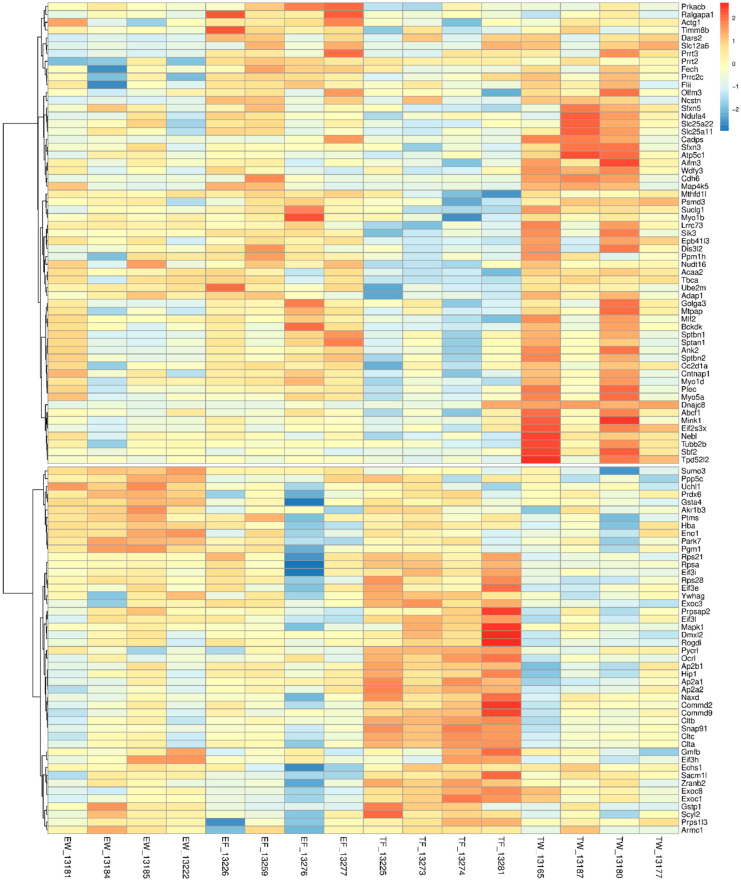
Heat map for proteins having significant interaction between treatment and genotype. Quantities elevated relative to the central value are shaded brown; reduced, blue. Quantities for each protein are standard-scaled (mean zero, variance one). Proteins are hierarchically clustered using Pearson correlation as the distance metric and the “ward.D2” agglomerative clustering method. The cluster tree is “cut” at the first branch point and proteins in “cut 1” generally are lower in trisomy-fluoxetine samples than trisomy-water; in “cut 2”, proteins are generally higher in trisomy-fluoxetine samples when compared to trisomy-water.

PCA results showed separation of the water-treated trisomic data from the fluoxetine-treated trisomic data (Figure 2). Analysis of variance (ANOVA) for PC 3 showed statistical significance of trisomic genotype, fluoxetine treatment, and their interaction (*p* values < 0.01, 0.015, and 0.021, respectively). These data strongly support a significant effect of fluoxetine in a trisomic genetic background that is distinct from (and more pronounced than) a euploid genetic background. To minimize reliance upon missing values when computing treatment effects, proteins that were not quantified in more than one sample were eliminated from the analysis. Following this step, 2066 of the 2554 quantified proteins were retained for further analysis. Proteins potentially playing a role in phenotypic rescue were identified using ANOVA analysis of the individual proteins. A nominal *p*-value threshold of 0.05 for the interaction was applied as decision rule criteria when selecting proteins, demonstrating the interaction for further analysis. In total, 106 proteins of the 2066 proteins having no more than one missing value were retained for further analysis (Tables S2–S5 and Figure 3). The heat map (Figure 3) for proteins having statistically significant interactions between treatment and genotype shows two clusters, as the first branch point proteins in “cut” one are lower in trisomy-fluoxetine samples (Table S3) than trisomy-water; the “cut” two exhibits higher values for the trisomy-fluoxetine group versus trisomic-water samples (Figure 3; Table S4).

### 3.2. GSEA of Proteins Ranked by PC 3 Loading

To characterize the proteins affected by fluoxetine in the trisomic genetic background, Gene Set Enrichment Analysis (GSEA) was performed using the 2554 proteins, ranked by their loadings for PC 3, using Reactome pathways as the gene sets. Enriched Reactome pathways are listed in order of normalized enrichment score. For each enriched pathway, the *p*-value reflects FDR control after BH-adjustment (Table S6).

### 3.3. Identification and Differential Expression of Trisomic Synaptic Trafficking Proteins and Their Rescue by Fluoxetine

Initially we investigated and confirmed the presence of several Down syndrome-related risk proteins in the synaptic proteome of Ts65Dn mice, including ARID1B, CCT8, DYRK1A, EZR, GART, INA, ITSN1, MRPL39, PCDH19, SNX9, and SYNJ1 [45,46,47,48]. This confirmation assured us that the synaptic fraction in our samples represented the well-characterized DS mouse model [45,46,48]. Many of the synaptic proteins differentially expressed in trisomic synaptosomes included proteins representing synaptic assembly (EXOC8), synaptic vesicle cycle (SYN3, NRXN1), synaptic vesicle endocytosis (SYNX9, PARK7, AP2A1, AP2A2, AP2B1), vesicle clustering and retrieval (PCLO, SYN3), vesicle exocytosis (RAB5A), vesicle acidification (DMXL2), vesicle endocytosis and replenishment (CLTA, CLTB, CLTC, OCRL), coated vesicle-associated kinase (SCYL2), synaptic maturation (NEFL1, EXOC1, EXOC3), synaptic signaling (NRXN1, HIP1, FMR1, ITSN1, YWHAG), synaptic traffic (EXOC1, SACM1L), and synaptic elimination (UBE3A) (Table 1 and Table 2 and Tables S2–S5). Several other brain proteins included endoplasmic reticulum (ER) transport protein (MYO5A), pre- and post-synaptic glutamate receptor protein (GRIN2A), and PSD cytoskeletal protein DBN1 (Table 1 and Table 2). 

Of these differentially expressed synaptic proteins in trisomic brains, several classes of proteins dealing with synaptic assembly, synaptic vesicular cycle, synaptic maturation and synaptic signaling, and trafficking were downregulated in water-treated trisomic brains (Table 1). Fluoxetine treatment subsequently caused upregulation of these trisomic proteins (Table 1 and Tables S2–S5).

Additionally, we identified downregulation in levels of several non-synaptic proteins such as eukaryotic translation initiation factors 2 and 3 (EiF family), ribosomal proteins (RPSA, RPS21, RPS28), mitochondrial proteins (PYCR3, NAXD, ECHS1), endosomal trafficking proteins (COMMD2, COMMD9), and immune-function-related glial maturation protein (GMFB) (Table 1). These differentially expressed significant alterations in synaptic and mitochondrial proteins were further verified using GSEA (Table 1 and Tables S6–S13) analyses.

Further analysis of proteomes for genes affected by treatment with fluoxetine versus water in Ts65Dn and euploid mice showed clear separation between water-treated trisomic mouse proteomes and fluoxetine-treated mouse proteomes versus the proteomes from euploid proteomic data (Figure 2). To characterize the proteins affected by fluoxetine in the trisomic genetic background, GSEA was performed using the 2554 proteins, ranked by their loading for PC3. Enriched pathways are listed in order of normalized scores (Table S6). For each enriched pathway, the *p*-value reflects FDR control after BH-adjustment (Table S6). Genes of enriched pathways are cited in Tables S6 and S7. Enriched pathways included gluconeogenesis, ubiquitination, electron transport membrane trafficking, lysosomes, trans-Golgi vesicle budding, and clathrin-mediated endocytosis (Tables S6 and S7). A heat map (Figure 3) for proteins having significant interaction between fluoxetine treatment and trisomic genotype shows two groups in trisomic proteomes, one group having upregulated trisomic proteins and the other having downregulated trisomic proteins (Table 1 and Table 2 and Tables S2–S5). P-interaction is the *p*-value for the significance of the interaction of trisomy and fluoxetine treatment based on ANOVA analysis (Tables S3–S5). These proteins potentially contribute to “phenotypic-rescue”. Some of the proteins that responded to fluoxetine will be discussed below. 

**Figure 4:**
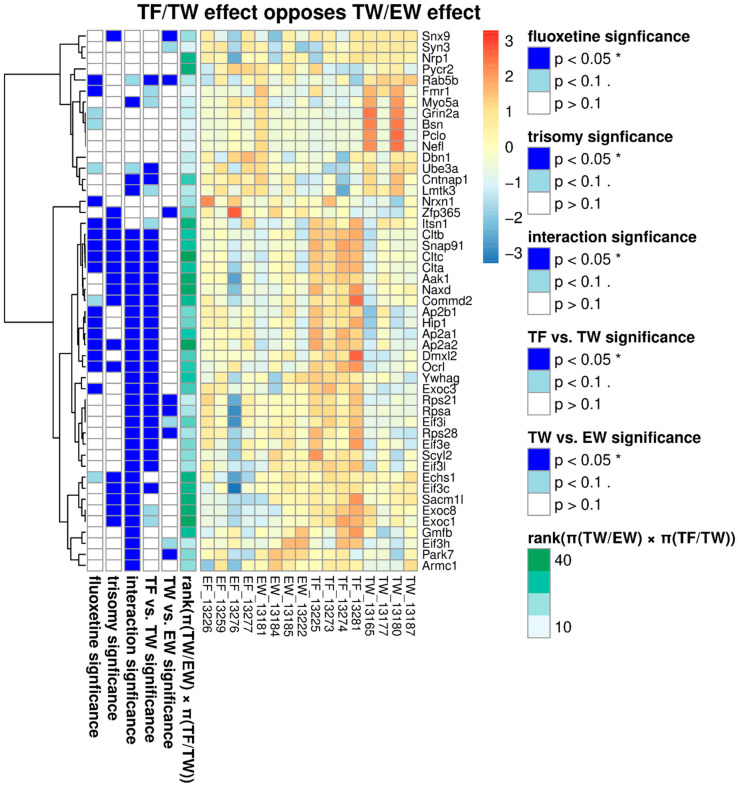
Heat map for selected synaptic proteins separated based on genotype (trisomic vs. euploid), treatment (fluoxetine vs. water), trisomy-by-fluoxetine interaction effect (*p* < 0.05), and rank (based on π value, which takes into account the effects of biological relevance and statistical significance of each protein). * indicates P at the 5% level (*p* < 0.05); period (.) indicates p at the 10% level (*p* < 0.1).

**Figure 5:**
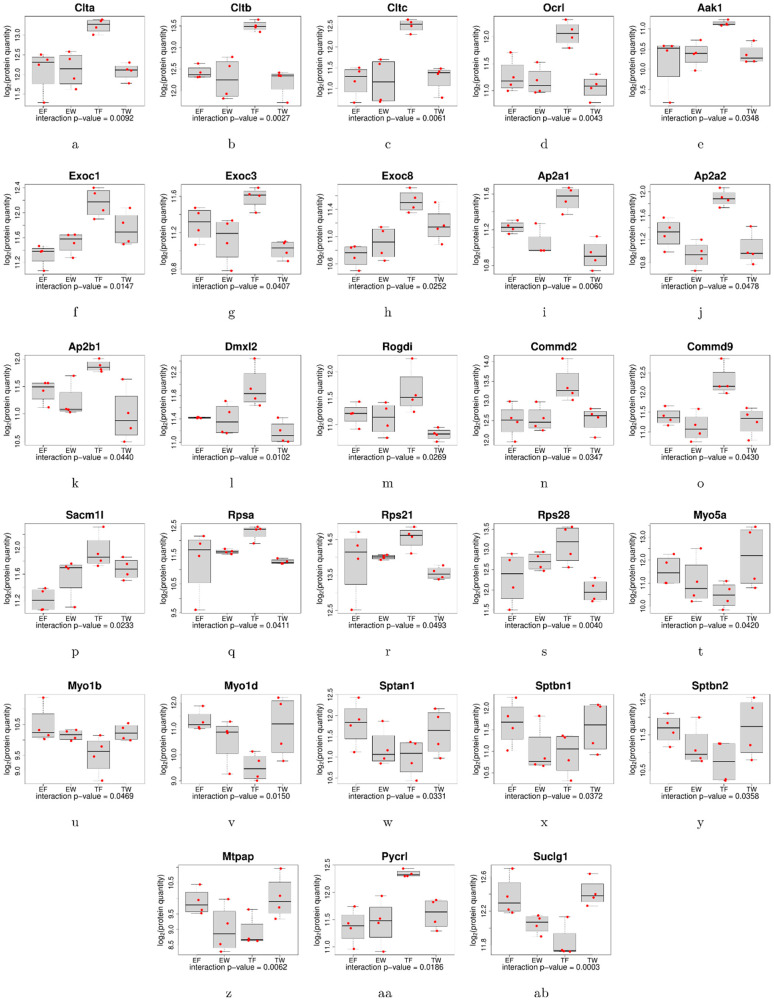
Boxplots of log_2_ (protein quantity) values for trisomic or euploid mice treated with fluoxetine or water. Each boxplot represents interquartile range, with the heavy band representing the median for each experimental group. Proteins have been grouped together based on general synaptic functions; clathrin-mediated endocytosis (**a**–**e**); synaptic maturation and assembly (**f**–**h**); vesicular endocytosis (**i**–**k**); acidification (**l**–**m**); endosomal and synaptic trafficking (**n**–**p**); ribosomal proteins (**q**–**s**); organelle and motor trafficking (**t**–**v**); synaptogenesis and neurotransmitter release (**w**–**y**); and mitochondrial proteins (**z**–**ab**).

Trisomic proteomes showed significant down-regulation in multiple synaptic proteins from different classes of cerebral-cortical genes (Table 1). Comparisons of the trisomic synaptic proteins treated with water (TW) versus trisomic fluoxetine-treated proteins (TF) revealed a rescue of the same neural markers after chronic treatment with fluoxetine (Table 1). Further evaluation of these proteins showed a significant (*p* < 0.05) trisomic-by-fluoxetine interaction effect (Table 1 and Tables S3–S5). Examples of synaptic classes of proteins that exhibited abnormalities in trisomic mice but were rescued following fluoxetine treatment included proteins involved in synaptic assembly (EXOC8, TW log_2_ = 11.07 ± 0.13; TF log_2_ = 11.52 ± 0.08; *p* = 0.0593; interaction *p*-value = 0.0252), synaptic vesicle endocytosis (AP2A1, TW log_2_ = 10.92 ± 0.08; TF log_2_ = 11.55 ± 0.07; *p* = 0.001; interaction *p*-value = 0.006), synaptic vesicle acidification (DMXL2, TW log_2_ = 11.17 ± 0.1, TF log_2_ = 11.94 ± 0.18; *p* = 0.0088; interaction *p*-value = 0.0102), synaptic vesicle replenishment (CLTA, TW log_2_ = 12.08 ± 0.11, TF log_2_ = 13.24 ± 0.09; *p* = 0.0002; interaction *p*-value = 0.0092), and coated vesicle-associated kinase (SCYL 2, TW log_2_ = 12.02 ± 0.09, TF log_2_ = 12.43 ± 0.09; *p* = 0.0170; interaction *p*-value = 0.0057). Indeed, in some cases, proteins associated with various synaptic vesicle functions included more than one protein per family/class of proteins. This was especially true with respect to multiple members of the clathrin family of proteins (clathrin A, clathrin B, and clathrin C) which were downregulated in trisomic synaptosomes but increased in protein levels after treatment with fluoxetine (Table 1 and Tables S2–S5, Figure 3, Figure 4 and Figure 5).

Other synaptic function abnormalities observed in trisomic mice included dysfunction in steps of synaptic maturation (EXOC1, EXOC2, Table 1 and Tables S3–S5, Figure 3, Figure 4 and Figure 5), synaptic signaling (HIP1, ITSN1, NRXN1, YWHAG, Table 1), and synaptic trafficking (EXOC1, SACM1L). Several family members of postsynaptic density proteins (PSD) and their partners exhibited abnormalities in trisomic protein levels which normalized after fluoxetine treatment; such as Eif3c (TW log_2_ = 10.31 ± 0.19, TF = 10.86 ± 0.10; *p* = 0.0414; interaction *p*-value = 0.0327), as well as Eif3e, Eif3h, Eif3i, and Eif3l (Table 1 and Tables S2–S5, Figure 3 and Figure 4). Several non-synaptic classes of proteins exhibiting abnormalities in trisomic mice which responded to fluoxetine treatment included ribosomal proteins (RPSA, RPS21, RPS28, Table 1), mitochondrial proteins (PYCR1, ECHS1), endosomal trafficking proteins (ARMC1, COMMD2, COMMD9), and NAXD and DRD2 splicing factor (ZRANB2). 

Another group of brain proteins which exhibited significant upregulation in the water-treated trisomic mice responded favorably to chronic treatment with fluoxetine and included synaptic, mitochondrial, and postsynaptic density molecules (Table 2). Fluoxetine effect rescued several of these trisomic proteins (Table 2) and included those related to the following classes: synaptogenesis (Sptbn3), synaptic structure (Eif2s3x, TPD52l2), synaptic transmission (MINK1), neurotransmitter release (Sptan1), synaptic trafficking (Sptbn2), and cytoskeleton (Nebulette/Nebl). Additional synaptic proteins are included and detailed in Table 2 and Tables S2–S5, and include MLF2, ACTG1, Slc25a22, and OLFM 3. An important protein concerned with presynaptic and synaptic elimination is UBE3A [49]. Levels of this protein were elevated in water-treated trisomic mice (TW log_2_ = 8.36 ± 1.50; TF log_2_ = 2.13 ± 1.27; *p* = 0.0191, interaction *p* = 0.0608, π = −20.5864) with a significant decrease following treatment with fluoxetine (Table 2 and Tables S2–S5). Non-synaptic mitochondrial proteins with significant trisomic-fluoxetine interaction (*p* < 0.05) included SFXN3, SFXN5, SlC25A11, NDUFA4, AIFM3, FECH, TIMM8B, ACAA2, and BCKDK.

To understand the significance of trisomic proteins which were rescued by fluoxetine, we analyzed enrichment of differentially expressed gene sets (Tables S6–S13) using gene set enrichment analysis (GSEA) of trisomic proteome in presence or absence of chronic treatment with fluoxetine. GSEA for TF/EW genes ranked using −log_10_(*p*-value) × log_2_(TF/EW) identified several important pathways in proteome of trisomic mice treated with fluoxetine, which included the modulation of clathrin-mediated endocytosis, trans-Golgi network vesicle budding, signaling by neurotrophic tyrosine receptor kinases (NTRKs), protein–protein interactions at the synapse, unblocking NMDA receptors, and glutamate binding and activators (Tables S8–S11). Reactome enrichment analysis confirmed the role of synaptic trafficking genes and pathways in the DS mouse model, and validated the potential efficacy of fluoxetine in rescue of dysfunctional genes in the mouse model.

Finally, there were 193 differentially expressed proteins in the fluoxetine-treated euploid mice (FDR-adjusted *p* < 0.05) (Table S2). A total of 30 proteins were affected by fluoxetine-treatment in trisomic and euploid mice (FDR-adjusted *p* < 0.05; Table S2); ten proteins were associated with cytoskeletal function (ACTG1, ANK2, AP2A2, BSN, DBN1, HBA, PCLO, PLEC, SPTAN1, SPTBN2), and one protein (SUCLG1) with mitochondrial function. The latter eleven proteins responded to fluoxetine and constituted some of the trisomic-responsive proteins identified earlier, but were also seen in the fluoxetine-treated euploid group as well. We surmise that fluoxetine has a major role to play in normal cytoskeletal functions [50].

## 4. Discussion

The current study evaluated the proteome of Ts65Dn mouse—an animal model of DS following chronic treatment with fluoxetine. We tested the hypothesis that treatment with fluoxetine during childhood will rescue important synaptic proteins involved in etiopathology of DS. We performed proteomic analysis of synaptosomal subcellular fractions of cerebral tissue from trisomic or euploid animals treated with fluoxetine or water, and quantified 2554 proteins. Our multivariate analysis showed that, for PCA scores of principal component 3, there is a significant interaction between fluoxetine treatment and genotype, strongly suggestive that the observed phenotypic rescue by fluoxetine is in large measure attributable to compensatory effects in trisomic animals that are distinct from fluoxetine’s effects in euploid animals. We identified significant alterations in levels of proteins involved in synaptic vesicle trafficking, endocytosis, exocytosis, synaptic maturation, and synaptic elimination in Ts65Dn mouse brain. Treatment with fluoxetine for two months resulted in partial or full rescue in many of the deregulated Ts65Dn proteins.

Ranking of differentially expressed proteins with significant impact (FDR adjusted *p* < 0.05, Table S2) in Ts65Dn mice, showed the fragile X mental retardation 1 protein (FMRP) to have the highest effect in the trisomic mice (π = −222.23). This result is not surprising, as FMRP targets ~1000 brain mRNAs, and thus stalls ribosomal translocation on mRNAs which are linked to synaptic functions [51]. Indeed, several of the proteins involved in the synaptic trafficking in the trisomic brain are targets of FMRP. Thus, significant upregulation of FMRP (TW/EW log_2_ FC = 6.23, FDR adjusted *p* = 0.0410) was normalized after treatment with fluoxetine (TF/TW log_2_ FC = −8.61, FDR adjusted *p* = 0.0010; Table 2 and Table S2). FMRP helps to sustain neurotransmitter release via activity-dependent bulk endocytosis [52]. Upregulation of FMRP as seen in trisomic mice led to inhibition of density core vesicle transport to synaptic boutons, and most likely led to altered anterograde axonal transport to synapses. [52,53,54]. Indeed, increases in FMRP levels led to increases in its targets, GRIN2A, WDFY3, synaptojanin 1, and MYO5A in trisomic brains (Table S2; FDR adjusted *p* < 0.05). Treatment with fluoxetine caused reversal in levels of these proteins, potentially helping to restore synaptic transmission and trafficking (Table S2; FDR adjusted *p* < 0.05 for GRIN2A, WDFY3, and MYO5A, except synaptojanin 1, where *p* = 0.230). More importantly, levels of two of FMRP targets that are involved in vesicular endocytosis (clathrin marker CLTC) and synaptic signaling (NRXN1) are restored and normalized following treatment with fluoxetine. Lastly, a recent report [55] confirms the upregulation of FMRP targets with normalization of synaptic proteins following treatment with fluoxetine in a mouse model of neurodevelopmental disorder, supporting the data presented here.

The exocyst complex (EXOC) is an evolutionarily-conserved member of the mammalian constitutive secretory pathway of proteins composed of eight genes [56]; these proteins are involved in multiple important functions including cell migration, autophagy, and fusion of secretory vesicles. Knockout of exocyst subunits can result in intracellular accumulation of secretory cargo [57]. We identified non-significant alterations in levels of EXOC1, EXOC3, and EXOC8 in trisomic water-treated synaptic fractions (Table S2; Figure 5). Treatment with fluoxetine resulted in statistically significant upregulation in levels of EXOC1, EXOC3, and EXOC8, members of the EXOC family (FDR adjusted *p* < 0.05 for EXOC1, EXOC3, and FDR adjusted trend for significance *p* < 0.073 for EXOC8, Table S2). Evaluation of the fluoxetine effect showed a significant trisomic-by-fluoxetine interaction effect in EXOC 1 (interaction *p* = 0.0147), EXOC3 (interaction *p* = 0.0407), and EXOC 8 (interaction *p* = 0.025; Table S4). Previous reports have documented the presence of variants in EXOC 7 and EXOC 8 genes [56] causing novel disorders of cerebral cortical development. The presence of fluoxetine effects as related to EXOC family of proteins in trisomic synaptic fractions is both novel and confirmatory of the potential therapeutic effects of fluoxetine in DS (Figure 5). Interestingly, as the yeast sec 6/8 complex (homologue of EXOC protein in yeast) accumulates in the trans-Golgi network after treatment with Brefeldin A (which interrupts transit through Golgi network) [58], normal distribution of these proteins at a steady state depends on continuous exocytic vesicle trafficking. It is tempting to speculate that fluoxetine’s beneficial effect in trisomic mice is through enhancement of vesicular trafficking [59,60] (Figure 5) by upregulating and normalizing levels of EXOC proteins. Indeed, several recent reports indicate that fluoxetine’s mechanism of action in treatment of depression is via enhancement of synaptic vesicle trafficking [59,60], as well as through synaptic transcriptional reprogramming [55].

We identified significant downregulation in clathrin family members CLTA, CLTB, and CLTC proteins in cortical synaptosomes in trisomic mice (Table S4). Clathrin-mediated vesicular endocytosis is the mechanism for delivery and recycling of synaptic vesicles [61]. Other functions for clathrin proteins include neurotransmission, signal transduction, movement of nutrients into neurons, and help in degradation of plasma membranes in lysosomes. Multiple proteins/genes are involved in the process of clathrin-dependent endocytosis. While it has been suggested that synaptic vesicle recycling is unaffected in Ts65Dn mice [62], we present novel data that indicate presence of abnormal cycling of synaptic vesicle trafficking in Ts65Dn mice (Table 1). Abnormalities of clathrin function can adversely affect synaptic transmission, and have been partially responsible for synaptic dysfunction in neurogenerative disorders (e.g., Parkinson’s disease and Alzheimer’s disease) [63,64]. Interestingly, fluoxetine treatment has been associated with improvement in synaptic vesicle trafficking in animal models of chronic social stress [59]. Thus, treatment of Ts65Dn mice with fluoxetine rescued brain levels of all three members of clathrin proteins (Table S4; Figure 5). Multivariate analysis of the clathrin protein data was significant for trisomy genotype, fluoxetine treatment, and for the interaction between fluoxetine and trisomy (CLTA interaction *p* = 0.0092; CLTB interaction *p* = 0.0027; CLTC interaction *p* = 0.0061; Table S4). Further analysis of GSEA of the synaptic data showed statistically significant involvement of the clathrin-mediated endocytosis pathway and proteins CLTB and CLTC (FDR adjusted *p* = 0.00001; normalized enrichment score = −2.29878, Table S6). Furthermore, the GSEA of Reactome pathway-enriched data exhibited similar involvement of CLTB and CLTC genes (FDR adjusted *p* = 0.0002; normalized enrichment score = 2.169) in the clathrin-dependent endocytosis (Tables S8 and S9).

Further analysis of our proteomic data showed the involvement of 56 proteins in the clathrin-mediated endocytosis (Table S6) pathway. One such protein family involved in vesicular endocytosis and synaptic vesicle cycle is the family of adaptor-related protein complex 2 subunits 2a1, 2a2, and 2b1 (AP2A1, AP2A2, AP2B1) [65,66]. Levels of all three AP2 proteins were downregulated significantly in trisomic brain cortices (Table 1, Tables S4 and S5, Figure 5). Treatment with fluoxetine corrected the deficits in these proteins (AP2A1, interaction *p* = 0.006; AP2A2, interaction *p* = 0.0478; AP2B1, interaction *p* = 0.044). AP2 is a 4-protein multimer, and is an evolutionarily-conserved protein family present in plants, fungi, and invertebrate species [67] which cooperates with clathrin proteins and participates in vesicular endocytosis. Fred et al. [68] demonstrated the presence of an interaction between fluoxetine and several other antidepressants with the TRKB receptor complex and endocytic adaptor complex AP2 proteins leading to the disruption of the TRKB-AP2 complex. These authors suggest that fluoxetine causes the disruption of the above-mediated complex promoting TRKB cell surface expression and subsequent BDNF signaling [68]. A recent report indicates colocalization of AP2A1 with neurofibrillary tangles in AP2A1 and AP2A2 plasmid-transfected cultured cells, suggesting the involvement of AP2 proteins in etiology of late-onset Alzheimer’s disease [69].

The level of synaptosome-associated protein 91 (SNAP91) was significantly reduced in trisomic-water treated synaptosomes (*p* = 0.0005; Tables S4 and S5). Treatment with fluoxetine-increased SNAP91 protein level significantly in the trisomic brain (trisomic-fluoxetine interaction *p* = 0.0002). SNAP91 is localized to presynaptic endocytic zone membrane, where it regulates clathrin-coated synaptic vesicle function [70]. SNAP91 is involved in synaptic vesicle endocytosis and recycling [71], and is considered to be a risk gene in schizophrenia [70]. Indeed, SNAP91 is a substrate of DYRK1A, a gene which plays a major role in pathology of DS [72,73]. Murakami et al. [73] reported DYRK1A can phosphorylate AP180 (SYNAP91) clathrin-coated-vesicles in both bound and unbound forms [73]. As levels of clathrin adaptor-related protein complexes are reduced in Ts65Dn synaptosomes, reductions in levels of (AP180/SNAP91) could either be due to effects of DYRK1A or due to interactions between SNAP91 and clathrin proteins. However, as levels of DYRK1A did not change in trisomic mice (Table S2), it is likely that other non-DYRK1A-related proteins may be involved in the downregulation of SNAP91 (Table S4). More importantly, fluoxetine’s identical effects on upregulating SNAP91 and clathrin may have therapeutic potential in treatment of DS. A previous report [74] has shown that clozapine increased expression of genes for a number of adaptor and clathrin assembly proteins (AP2A2, AP2B1, AP180, CLINT1, HIP1, ITSN, and PICALM) in human neuroblastoma cell lines, potentially due to involvement of AP180, serotonin 1A, and lysophosphatidic acid receptor 2, indicating a similar mechanism being operational in the actions of clozapine and fluoxetine.

Level of SACM1L/SAC1, an important lipid phosphatase that shuttles between ER and the Golgi complex [75,76], was downregulated non-significantly in the cerebral cortex of water-treated trisomic mice (Tables S2 and S5; Figure 5). Following treatment with fluoxetine, SACM1L protein level increased significantly (trisomic *p* = 0.0038; trisomic-fluoxetine interaction *p* = 0.0233, Tables S2 and S4). SACM1L converts phosphatidylinositol-4-phosphate (PI(4)P) to phosphatidylinositol to promote anterograde transport of secretory proteins from the Golgi complex to PSD [76]. In quiescent cells, SACM1L accumulates in the Golgi complex. However, upon activation by growth factors, SACM1L triggers retrograde traffic from Golgi to ER [75], accelerating constitutive secretion of the secretory proteins. Thus, maintaining the normal function of SACM1L via fluoxetine helped both anterograde and retrograde trafficking from the Golgi complex to the plasma membrane. Forrest et al. [77], reported that downregulation of SACM1L can disrupt axonal transport in a fly model of Amyotrophic Lateral Sclerosis (ALS) and in humans who have ALS, implicating a crucial role of phosphoinositide levels in ALS etiology.

An important protein which acts as a regulator of organelle acidification is DMXL2 (Rabconnectin-3a), which was significantly downregulated in the trisomic cerebral cortex (Table 1, Tables S4 and S5). Treatment of trisomic mice with fluoxetine increased protein level for DMXL2 (Trisomic-fluoxetine interaction *p* = 0.0102; TW-TF *p* value = 0.0088, Tables S4 and S5). Zebrafish mutants for rabconnectin-3a had high pH with deficient vacuolar-type activity (V-ATPase) in the synaptic vesicles and exhibited reduced firing rates and reduced action potentials [78]. These authors suggested that their results are consistent with deacidification of synaptic vesicles leading to impairment in synaptic transmission in the mutant zebrafish [78]. As tight control of pH levels in ER, Golgi compartments, and lysosomes/endosomes are important in normal functioning of vesicular traffic; decreased levels of DMXL2 in trisomic mice may contribute to trafficking dysfunction in Ts65Dn brain function. Elevation of DMXL2 by fluoxetine helps ameliorate organelle acidification in trisomic mice.

Several trisomic proteins that were upregulated in trisomic mice included members of protein families that deal with vesicle exocytosis (Rab5B), synaptic signaling (FMR1), synaptic elimination (UBE3A; recent evidence supports the role of UBE3A in excessive synaptic elimination in Angelman’s syndrome and autism [49]), ER-PSD transport (MYO5A), PSD cytoskeleton (DBN1), a-synuclein transport (MYO1D), and NMDA receptor activity (GRIN2A). Fluoxetine treatment led to normalization of all of these proteins (Table 2). A protein of great interest is MYO1D, a molecular motor protein which transports a-synuclein from blood to brain [79], was upregulated in trisomic brain (TW log_2_ 11.09 ± 0.59; trend *p* = 0.0512) but decreased significantly (TF log_2_ = 9.52 ± 0.26) after treatment with fluoxetine (interaction *p*-value = 0.015; Tables S2 and S3). MYO1D has been considered as a target for pharmacotherapy in Parkinson’s disease [79]. The potential efficacy of fluoxetine in reducing this protein may be valuable in the treatment of DS and other neurodevelopmental/neurodegenerative disorders. Mutations in MYO1D have been linked to autism [80], confirming etiopathogenesis of this protein in neurodevelopmental as well as neurodegenerative disorders.

Based on unbiased proteomic data presented here, we suggest that DS is a neurodevelopmental clathrin-mediated synaptopathy which impacts normal synaptic signaling adversely in a susceptible individual (Table 1 and Table 2). Fluoxetine treatment during a sensitive period of brain development (P28–88 postnatal days) rescues synaptic proteins involved in clathrin-mediated functions restoring synaptic communication in the Ts65Dn mouse model. As fluoxetine enhances vesicular trafficking, it may be a potential treatment for DS.

There are limitations with the Ts65Dn mouse model in this study. Ts65Dn mice contain only about half of the genes that are homologous to human chromosome 21 (Hsa21). Additionally, the freely segregating extra chromosome contains ~35 protein-coding genes that are not homologous to Hsa21 [35,81], which may skew the comparison of these results to individuals with DS.

In conclusion, proteomic investigation of the cerebral cortex in a trisomic mouse model of DS demonstrated significant downregulation of proteins involved in vesicular trafficking, including vesicular endocytosis (CLTA, CLTB, CLTC), synaptic assembly and maturation (EXOC1, EXOC3, EXOC8), anterograde axonal transport (EXOC1), retrograde intracellular transport (SAC1), endosomal-lysosomal acidification (DMXL2), and synaptic signaling (NRXN1, ITSN1, HIP1, YWHAG). Additionally, trisomic proteomes revealed upregulation of several trafficking proteins involved in vesicular exocytosis (Rab5B), synapse elimination (UBE3A), scission of endocytosis (DBN1), transport of ER in dendritic spines (MYO5A), presynaptic activity-dependent bulk endocytosis (FMR1), and NMDA receptor activation (GRIN2A). Early chronic fluoxetine treatment of Ts65Dn mice rescued synaptic vesicular abnormalities and prevented abnormal proteomic changes in adult Ts65Dn mice, pointing to fluoxetine’s efficacy in treatment of DS.

## Figures and Tables

**Table 1:** Selected downregulated Trisomic proteins and their response to chronic administration of fluoxetine in Ts65Dn mouse model.

Class	Protein	Trisomic-Water	Trisomic-Fluoxetine	Significant T–F Interaction	TF Significance	
					FDR-adj P	Nominal P
**I. Synaptic Function**						
**A. Synaptic Assembly**	EXOC8	↓	↑	P	+	Trend +
**B. Synaptic Vesicle Cycle**	NRXN1	↓	↑	P	+	−
**Vesicle Endocytosis**	PARK7	↓	↑	P	+	−
	AP2A1	↓	↑	P	+	+
	AP2A2	↓	↑	P	+	+
	AP2B1	↓	↑	P	+	+
**Vesicle Recycling**	SNAP91	↓	↑	P	+	+
**Vesicle Acidification**	DMXL2	↓	↑	P	+	+
**Vesicle Endocytosis and Replenishment**	CLTA CLTB	↓ ↓	↑ ↑	P P	+ +	+ +
	CLTC	↓	↑	P	+	+
	OCRL	↓	↑	P	+	+
**Coated Vesicle-Associated Kinase**	SCYL2	↓	↑	P	+	+
**C. Synaptic Maturation**	EXOC1	↓	↑	P	+	Trend +
	EXOC3	↓	↑	P	+	+
**D. Synaptic Signaling**	NRXN1	↓	↑	P	+	−
	HIP1	↓	↑	P	+	+
	ITSN1	↓	↑	A	+	−
	YWHAG	↓	↑	P	+	+
**E. Synaptic Traffic**	EXOC1	↓	↑	P	+	Trend +
	SACM1L	↓	↑	P	+	-
**II. Ribosomal Proteins**	RPSA	↓	↑	P	+	+
	RPS21	↓	↑	P	+	+
	RPS28	↓	↑	P	+	+
**III. mRNA Translation**	EIF3C	↓	↑	P	−	+
	EIF3E	↓	↑	P	+	+
	EIF3H	↓	↑	P	+	−
	EIF3I	↓	↑	P	+	+
	EIF3L	↓	↑	P	+	+
**IV. Mitochondria**	PYCR1	↓	↑	P	ND	+
	NAXD	↓	↑	P	+	+
	ECHS1	↓	↑	P	−	−
	EIF3L	↓	↑	P	+	+
**V. Endosomal Traffic**	ARMC1	↓	↑	P	+	−
	COMDD2	↓	↑	P	+	+
	COMMD9	↓	↑	P	+	+
**VI. Dopamine receptor D2 Splicing Factor**	ZRANB2	↓	↑	P	+	+
**VII. Immune**	GMFB	↓	↑	P	−	−

P = Presence of significant trisomic-by-fluoxetine interaction; A = Absence of significant trisomic-by-fluoxetine interaction; ↑ = upregulation; ↓ = downregulation; FDR-adjusted *p*-values for trisomic-fluoxetine ratios (Table S2); nominal *p*-values for trisomic-fluoxetine values (Tables S3–S5); ND, not determined; + = significant statistically; − = nonsignificant statistically.

**Table 2:** Selected upregulated trisomic proteins and their response to chronic administration of fluoxetine in Ts65Dn mouse model.

Class	Protein	Trisomic-Water	Trisomic-Fluoxetine	Significant T–F Interaction	TF Significance	
					FDR-adj P	Nominal P
**A.** **Synaptogenesis**	Sptbn2	↑	↓	P	+	−
**B.** **Synaptic Structure**	EIF2S3X TPD52L2	↑ ↑	↓ ↓	P P	− +	+ +
**C.** **Synaptic Transmission**	MINK1 TUBB2B	↑ ↑	↓ ↓	P P	+ ND	− +
**D.** **Neurotransmitter Release**	Sptan1	↑	↓	P	+	−
**E.** **Dendritic Spine**	SPtbn2	↑	↓	P	+	−
**F.** **Synaptic Vesicle Endocytosis**	ACTG1	↑	↓	P	+	−
**G.** **Synaptic Vesicle Exocytosis**	RAB5B	↑	↓	P	+	+
**H.** **Synaptic Interaction Functions**	MLF2 SLC25A22	↑ ↑	↓ ↓	P P	+ -	+ +
**I.** **Pre- and Post-Synapse** **Presynaptic and Synaptic Elimination**	NRXN4/CNTNAP1 Sptbn2 MYO5A CC2D1A UBE3A	↑ ↑ ↑ ↑ ↑	↓ ↓ ↓ ↓ ↓	P P P P Trend	+ + + + +	+ − Trend + − +
**J.** **PSD** **ER-PSD Transport** **Pre- and Postsynaptic Potential** **Cytoskeleton**	MINK1 Sptan1 Sptbn2 MYO5A GRIN2A DBN1	↑ ↑ ↑ ↑ ↑ ↑	↓ ↓ ↓ ↓ ↓ ↓	P P P P A A	+ + + + + +	− − − Trend + − −
**K.** **Cytoskeleton**	NEBL MYO1B	↑ ↑	↓ ↓	P P	+ −	+ Trend +
**L.** **Organelle and Vesicle Traffic** **Golgi Complex**	MYO1B GOLGA3 Sptbn3	↑ ↑ ↑	↓ ↓ ↓	P P P	− − +	Trend + − −
**M.** **Proteosomal Degradation**	P5MD3	↑	↓	P	+	+
**N.** **Mitochondria**	SUCLG1 MTPAP SFXN3 SFXN5 SLC25A11 SLC25A22 NDUFA4 AIFM3 FECH TIMM8B ACAA2 ATP5C1 BCKDK	↑ ↑ ↑ ↑ ↑ ↑ ↑ ↑ ↑ ↑ ↑ ↑ ↑	↓ ↓ ↓ ↓ ↓ ↓ ↓ ↓ ↓ ↓ ↓ ↓ ↓	P P P P P P P P P P P P P	+ Trend + + + + − + + + − + + +	+ + + + + + − + − − + + +
**O.** **Scaffold**	ANK2	↑	↓	P	+	Trend +
**P.** **Gene Stabilization**	NUDT16	↑	↓	P	+	ND
**Q.** **Transport**	MYO1D MYO5A	↑ ↑	↓ ↓	P P	+ +	Trend + Trend +
**R.** **Traffic**	LMTK3	↑	↓	P	+	Trend +
**S.** **Vesicle Traffic**	PLEC	↑	↓	P	+	Trend +
**T.** **Axon**	TUBB2B MYO1B Sptan1	↑ ↑ ↑	↓ ↓ ↓	P P P	ND − +	+ Trend + −
**U.** **Other Functions**	FMR1 WDFY3 UBE2M PRRC2C FLii DIS3L2 LRRC73 ISCA2 SIK3 OLFM3 ARFGAP/ADAP1 PPM1H	↑ ↑ ↑ ↑ ↑ ↑ ↑ ↑ ↑ ↑ ↑ ↑	↓ ↓ ↓ ↓ ↓ ↓ ↓ ↓ ↓ ↓ ↓ ↓	A P P P P P P P P P P P	+ + − − − + + Trend + + + + −	Trend + + + − − − + ND + − + ND

P = Presence of significant trisomic-by-fluoxetine interaction; A = Absence of significant trisomic-by-fluoxetine interaction; ↑ = upregulation; ↓ = downregulation; FDR-adjusted *p*-values for trisomic-fluoxetine ratios (Table S2); nominal *p*-values for trisomic-fluoxetine values (Tables S3–S5); ND, not determined; + = significant statistically; − = nonsignificant statistically.

## Data Availability

The mass spectrometry proteomics data have been deposited to the Proteome Xchange Consortium via the PRIDE partner repository with the data set identifier PXD048722 and DOI 10.6019/PXD048722.

## Supplementary Materials

The following supporting information can be downloaded at: https://www.mdpi.com/article/10.3390/genes15040452/s1. Table S1 contains demographic data related to trisomic and euploid body weights, brain weights, and intake of water and fluoxetine; Table S2 contains differential expression of 2554 in synaptic fractions of all experimental animals; Tables S3 and S4 contain lists of dendrograms cut 1 and cut 2; Table S5 includes protein levels and their response to fluoxetine for selected synaptic brain markers; Tables S6 and S7 contain GSEA-enriched pathways and genes for PC3 data; Tables S8 and S9 contain Reactome-enriched pathways for genes and proteins ranked by π (TF/EW); Tables S10 and S11 contain GSEA-enriched pathways for genes and proteins ranked by π (TW/EW); Tables S12 and S13 contain GSEA-enriched pathways for genes and proteins ranked by π (EF/EW).
