# Supplementary material for: Early Chronic Fluoxetine Treatment of Ts65Dn Mice Rescues Synaptic Vesicular Deficits and Prevents Aberrant Proteomic Alterations
Source: Genes (Basel). 2024 Apr 3;15(4):452. doi: 10.3390/genes15040452 (PMC11049293; doi:10.3390/genes15040452)
Supplement: Supplementary file 1 [file genes-15-00452-s001.zip › genes-2917550-supplementary.pdf]

Supplementary Table S1 (1A-1F): Mean ± SD and T-Test data for various parameters in euploid versus trisomic mice.

1A. Fluoxetine Intake:

Euploid			Ts65Dn			
Fluoxetine (mg/kg/day)	SD	N	Fluoxetine (mg/kg/day)	SD	N	P Value
8.92	1.35	4	10.14	1.72	5	0.29

1B. Average water intake (Euploid vs. Ts65Dn):

Euploid			Ts65Dn			
Average Daily Water Intake (mL)	SD	N	Average Daily Water Intake (mL)	SD	N	P Value
6.13	0.87	10	5.63	1.06	12	0.25

1C. Cortical Weight (Treatment vs. Control):

Euploid							Ts65Dn						
Fluoxetine			Control				Fluoxetine			Control			
Cortical Weight (g)	SD	N	Cortical Weight (g)	SD	N	P Value	Cortical Weight (g)	SD	N	Cortical Weight (g)	SD	N	P Value
0.19	0.04	4	0.17	0.02	6	0.51	0.18	0.04	5	0.17	0.04	7	0.64

1D. Cortical Weight (Euploid vs. Ts65Dn):

Fluoxetine							Control						
Euploid			Ts65Dn				Euploid			Ts65Dn			
Cortical Weight (g)	SD	N	Cortical Weight (g)	SD	N	P Value	Cortical Weight (g)	SD	N	Cortical Weight (g)	SD	N	P Value
0.19	0.04	4	0.18	0.04	5	0.75	0.17	0.02	6	0.17	0.04	7	0.72

1E. Body Weights (Comparing Treatment vs. Control):

	Euploid							Ts65Dn						
	Fluoxetine			Control				Fluoxetine			Control			
Age (Days)	Weight (g)	SD	N	Weight (g)	SD	N	P Value	Weight (g)	SD	N	Weight (g)	SD	N	P Value
28	18.28	2.096	4	19.42	1.512	6	0.34	13.16	4.247	5	15.06	1.809	7	0.31
88	30.32	2.388	4	31.57	2.633	6	0.47	24.66	4.312	5	27.1	3.763	7	0.32

1F. Body Weights (Euploid vs. Ts65Dn):

	Fluoxetine							Control						
	Euploid			Ts65Dn				Euploid			Ts65Dn			
Age (Days)	Weight (g)	SD	N	Weight (g)	SD	N	P Value	Weight (g)	SD	N	Weight (g)	SD	N	P Value
28	18.28	2.096	4	13.16	4.247	5	0.07	19.42	1.512	6	15.06	1.809	7	< 0.01
88	30.32	2.388	4	24.66	4.312	5	0.05	31.57	2.633	6	27.1	3.763	7	0.03

Table S2: Differentially expressed proteins in cerebral cortex

gene	$\pi_{TW/EW} \times \pi_{TF/TW}$	log2FC TW/EW	padj TW/EW	log2FC TF/TW	padj TF/TW	log2FC EF/EW	padj EF/EW	log2FC TF/EW	padj TF/EW	description	accession
2410002F23Rik	-19.6676	6.06	0.23	-7.05	0.19	-0.78	0.82	-1.00	0.78	RIKEN cDNA 2410002F23 gene	Q3TE80
2900026A02Rik	-4.7211	3.09	0.065	-2.20	0.26	0.99	0.43	0.89	0.47	RIKEN cDNA 2900026A02 gene	A0A1B0GR85
4833439L19Rik	0.0000	0.01	0.85	-0.02	0.7	0.12	0.99	-0.01	0.8	Putative monooxygenase p33MONOX	D3Z1F7
6430548M08Rik	0.0000	0.02	0.86	0.00	0.88	-0.23	0.0270	0.02	0.98	RIKEN cDNA 6430548M08 gene	Q3TA40
Aacs	-0.3068	3.09	0.92	-7.08	0.41	3.49	0.55	-3.99	0.62	Acetoacetyl-CoA synthetase	Q9D2R0
Aak1	0.0565	0.05	0.42	0.75	0.0001	-0.04	0.44	0.79	0.0001	AP2-associated protein kinase 1	Q3UHJ0
Aars1	0.0012	-0.31	0.85	-0.07	0.17	-0.18	0.11	-0.38	0.17	Alanine–tRNA ligase, cytoplasmic	Q8BGQ7
Abat	0.0033	-0.16	0.52	-0.16	0.35	-0.19	0.22	-0.32	0.089	4-aminobutyrate aminotransferase, mitochondrial	P61922
Abcb1a	-0.0025	0.16	0.5	-0.16	0.47	0.09	0.76	0.00	0.86	ATP-dependent translocase ABCB1	P21447
Abcb7	-0.2556	0.36	0.15	-0.77	0.076	-0.14	0.78	-0.42	0.56	ATP-binding cassette sub-family B member 7, mitochondrial	Q61102
Abcb8	0.0006	0.22	0.37	0.13	0.89	0.35	0.36	0.34	0.43	Mitochondrial potassium channel ATP-binding subunit	Q9CXJ4
Abcd2	-1.5618	1.63	0.0110	-0.91	0.29	-0.19	0.8	0.72	0.0200	ATP-binding cassette sub-family D member 2	A0A0R4J0U5
Abcd3	0.1102	1.42	0.0480	0.22	0.54	-0.72	0.62	1.65	0.0026	ATP-binding cassette sub-family D member 3	P55096
Abce1	-0.0541	0.36	0.75	-1.08	0.077	0.01	0.75	-0.72	0.083	ATP-binding cassette sub-family E member 1	P61222
Abcf1	-3.2378	0.70	0.0220	-1.05	0.0022	-0.02	0.57	-0.35	0.34	ATP-binding cassette sub-family F member 1	Q6P542
Abcf2	0.0057	1.63	0.08	0.73	0.99	-0.21	0.85	2.36	0.15	ATP-binding cassette sub-family F member 2	Q99LE6
Abcg2	0.0001	-0.24	0.32	-0.02	0.93	0.37	0.35	-0.26	0.37	ATP-binding cassette sub-family G member 2	A0A0R4J0B6
Abhd12	-0.1108	0.42	0.0150	-0.22	0.22	-0.53	0.51	0.21	0.12	Lysophosphatidylserine lipase ABHD12	Q99LR1
Abhd16a	-0.0651	0.41	0.41	-0.95	0.37	0.40	0.62	-0.54	0.66	Phosphatidylserine lipase ABHD16A	Q9Z1Q2
Abi2	-0.0671	0.29	0.23	-0.34	0.086	0.17	0.65	-0.05	0.62	Abi2 protein	Q6AXD2
Ablim2	-0.2260	1.27	0.7	-2.02	0.27	0.87	0.62	-0.75	0.45	Actin-binding LIM protein 2 (Fragment)	F6VVW4
Abr	0.1887	0.21	0.41	0.58	0.0001	0.06	0.63	0.79	0.0001	Active breakpoint cluster region-related protein	Q5SSL4
Acaa2	-0.0894	0.12	0.38	-0.56	0.0007	0.03	0.76	-0.44	0.0260	3-ketoacyl-CoA thiolase, mitochondrial	Q8BWT1
Acaca	0.0991	1.97	0.11	0.42	0.75	1.57	0.2	2.39	0.12	Acetyl-CoA carboxylase 1	Q5SWU9
Acad11	0.0316	0.55	0.31	0.30	0.42	0.64	0.2	0.85	0.1	Acyl-CoA dehydrogenase family member 11	A0A0R4J0I6
Acad12	-0.3452	-0.52	0.076	0.53	0.076	-0.16	0.45	0.00	1	Acyl-Coenzyme A dehydrogenase family, member 12	D3Z7X0

Table S2: Differentially expressed proteins in cerebral cortex *(continued)*

gene	$\pi_{TW/EW} \times \pi_{TF/TW}$	log2FC TW/EW	padj TW/EW	log2FC TF/TW	padj TF/TW	log2FC EF/EW	padj EF/EW	log2FC TF/EW	padj TF/EW	description	accession
Acad8	-0.2321	3.53	0.84	-3.79	0.59	4.99	0.12	-0.26	0.71	Isobutyryl-CoA dehydrogenase, mitochondrial	A0A0R4J0P1
Acad9	0.0000	-0.01	0.37	-0.01	0.23	0.15	0.66	-0.02	0.85	Complex I assembly factor ACAD9, mitochondrial	Q8JZN5
Acadl	-0.0736	0.29	0.0220	-0.24	0.23	-0.43	0.22	0.04	0.83	Long-chain specific acyl-CoA dehydrogenase, mitochondrial	A0A0R4J083
Acadm	-0.0015	0.04	0.62	-0.20	0.12	0.06	0.92	-0.16	0.38	Medium-chain specific acyl-CoA dehydrogenase, mitochondrial	P45952
Acads	-0.0001	-0.07	0.95	0.37	0.59	-0.01	0.88	0.30	0.59	Short-chain specific acyl-CoA dehydrogenase, mitochondrial	Q07417
Acadsb	0.0002	0.12	0.17	0.09	0.94	-0.07	0.78	0.21	0.14	Short/branched chain specific acyl-CoA dehydrogenase, mitochondrial	Q9DBL1
Acadvl	-0.0003	0.03	0.64	-0.10	0.37	-0.06	0.7	-0.07	0.85	Very long-chain-specific acyl-CoA dehydrogenase, mitochondrial	B1AR28
Acan	0.1793	-0.46	0.0370	-0.55	0.32	0.10	0.59	-1.01	0.0010	Aggrecan core protein	Q61282
Acap2	-0.0001	-0.20	0.93	0.30	0.85	0.15	0.54	0.10	0.87	Arf-GAP with coiled-coil, ANK repeat and PH domain-containing protein 2	D4AFX6
Acat1	-0.0028	0.08	0.42	-0.12	0.17	0.04	0.97	-0.04	0.69	Acetyl-CoA acetyltransferase, mitochondrial	Q8QZT1
Acly	0.0040	0.28	0.13	0.10	0.69	0.21	0.57	0.38	0.2	ATP-citrate synthase	Q3V117
Aco1	0.0019	-0.17	0.22	-0.10	0.67	-0.72	0.0008	-0.27	0.0020	Cytoplasmic aconitate hydratase	P28271
Aco2	0.0483	-0.09	0.29	-0.37	0.0020	0.05	0.96	-0.45	0.3	Aconitate hydratase, mitochondrial	Q99KI0
Acot11	-0.0462	0.71	0.29	-0.38	0.48	-0.07	0.93	0.33	0.76	Acyl-coenzyme A thioesterase 11	A2AVR6
Acot13	-0.2120	0.22	0.16	-0.48	0.0030	-0.40	0.16	-0.26	0.26	Acyl-coenzyme A thioesterase 13	Q9CQR4
Acot7	0.0246	-0.22	0.74	-0.93	0.12	-0.43	0.68	-1.15	0.098	Cytosolic acyl coenzyme A thioester hydrolase	Q91V12
Acot9	1.7074	0.80	0.0046	0.57	0.0250	0.15	0.79	1.37	0.0001	Acyl-coenzyme A thioesterase 9, mitochondrial	Q9R0X4
Acox1	-0.0106	0.34	0.37	-0.22	0.47	0.19	0.66	0.12	0.65	Peroxisomal acyl-coenzyme A oxidase 1	Q9R0H0
Acsbg1	-0.0003	0.20	0.82	-0.21	0.81	0.50	0.24	-0.01	0.95	Long-chain-fatty-acid–CoA ligase ACSBG1	Q99PU5
Acsl1	-0.0134	0.17	0.5	-0.33	0.16	-0.02	0.71	-0.16	0.33	Long-chain-fatty-acid–CoA ligase 1	D3Z041
Acsl6	-0.0047	0.16	0.84	-0.55	0.2	-0.15	0.83	-0.39	0.42	Long chain acyl-CoA synthetase 6 isoform 3	Q5ICG5
Acss1	-0.1186	0.75	0.7	-1.46	0.2	0.41	0.72	-0.71	0.59	Acetyl-coenzyme A synthetase 2-like, mitochondrial	Q99NB1
Actg1	-1.0448	0.33	0.0350	-0.64	0.0004	0.73	0.0170	-0.31	0.23	Actin, cytoplasmic 2	P63260
Actn1	-1.1986	0.71	0.17	-1.12	0.0110	1.12	0.0270	-0.41	0.27	Alpha actinin 1a	A1BN54
Actn4	-0.0003	-0.09	0.61	0.09	0.63	0.65	0.077	0.00	0.96	Alpha-actinin-4	A0A1L1SV25

Table S2: Differentially expressed proteins in cerebral cortex *(continued)*

gene	$\pi_{TW/EW} \times \pi_{TF/TW}$	log2FC TW/EW	padj TW/EW	log2FC TF/TW	padj TF/TW	log2FC EF/EW	padj EF/EW	log2FC TF/EW	padj TF/EW	description	accession
Actr10	0.0017	0.04	0.63	0.18	0.063	0.50	0.0270	0.22	0.052	Actin-related protein 10	Q9QZB7
Actr1a	0.0003	0.06	0.84	0.27	0.52	0.89	0.0013	0.33	0.071	Alpha-centractin	P61164
Actr1b	-0.0036	-0.07	0.76	0.36	0.064	0.27	0.093	0.29	0.0110	Beta-centractin	Q8R5C5
Actr2	0.0000	-0.14	0.68	0.00	0.73	0.12	0.49	-0.14	0.47	Actin-related protein 2	P61161
Actr3	0.0002	-0.11	0.97	-0.21	0.24	0.05	0.82	-0.32	0.27	Actin-related protein 3	Q99JY9
Actr3b	0.0000	-0.12	0.99	-0.06	0.17	0.00	0.76	-0.18	0.0220	Actin-related protein 3B	Q641P0
Acyp1	0.1313	-0.67	0.0060	-0.32	0.53	-0.27	0.11	-0.99	0.0003	Acylphosphatase	E9QJT5
Adam10	0.0000	-0.10	0.98	0.17	0.5	-0.07	0.92	0.07	0.61	Disintegrin and metalloproteinase domain-containing protein 10	O35598
Adam11	-0.1013	0.28	0.0340	-0.32	0.17	-0.07	0.74	-0.04	0.77	Adam11 protein	Q7TQG7
Adam22	0.0080	0.14	0.0140	0.05	0.24	0.16	0.34	0.19	0.27	Disintegrin and metalloproteinase domain-containing protein 22	D3YUP9
Adam23	-0.0001	0.07	0.87	-0.17	0.73	0.47	0.067	-0.11	0.4	Disintegrin and metalloproteinase domain-containing protein 23	Q9R1V7
Adap1	-0.0156	0.11	0.81	-0.74	0.0080	-0.01	0.95	-0.64	0.0070	ArfGAP with dual PH domains 1	E9PY16
Adcy1	-0.1756	1.51	0.41	-1.23	0.57	0.22	0.65	0.28	0.73	Adenylate cyclase type 1	O88444
Adcy2	-0.0179	0.36	0.11	-0.13	0.4	-0.14	0.68	0.23	0.94	Adenylate cyclase type 2	Q80TL1
Adcy5	0.0038	0.12	0.61	0.41	0.44	0.47	0.054	0.53	0.29	Adenylate cyclase type 5	P84309
Adcy9	-0.0001	0.23	0.43	-0.03	0.93	0.46	0.22	0.20	0.46	Adenylate cyclase type 9	P51830
Add1	0.0000	-0.04	0.94	-0.09	0.58	0.24	0.42	-0.13	0.77	Alpha-adducin	Q9QYC0
Add2	0.0003	-0.06	0.87	-0.10	0.11	0.30	0.0010	-0.16	0.11	Beta-adducin	Q9QYB8
Add3	-0.0052	-1.20	0.46	0.16	0.83	-0.64	0.76	-1.03	0.67	Gamma-adducin	Q9QYB5
Adgra1	0.0209	-0.50	0.84	-0.74	0.18	-0.85	0.33	-1.24	0.0350	Adhesion G protein-coupled receptor A1	Q8C4G9
Adgrb1	-0.0001	0.28	0.93	-0.14	0.87	-0.10	0.75	0.14	0.91	Adhesion G protein-coupled receptor B1	Q3UHD1
Adgrb3	0.0301	-0.31	0.29	-0.30	0.25	0.08	0.81	-0.61	0.054	Adhesion G protein-coupled receptor B3 (Fragment)	Q68FL1
Adgrl1	-0.0507	0.22	0.13	-0.25	0.091	0.16	0.54	-0.02	0.67	Adhesion G protein-coupled receptor L1	H7BX15
Adgrl3	-0.0007	0.16	0.93	-0.55	0.56	-0.88	0.58	-0.39	0.49	Adhesion G protein-coupled receptor L3	D3Z3G4
Adh5	-0.5693	-0.91	0.0010	0.28	0.18	-0.49	0.068	-0.63	0.0280	Alcohol dehydrogenase class-3	P28474
Ado	0.0132	-0.19	0.19	-0.17	0.27	0.17	0.18	-0.36	0.0220	2-aminoethanethiol dioxygenase	Q6PDY2
Adss2	0.0050	-0.06	0.54	-0.38	0.15	-0.19	0.86	-0.44	0.65	Adenylosuccinate synthetase isozyme 2	P46664
Afdn	-1.7335	0.75	0.0170	-0.68	0.0120	0.36	0.27	0.07	0.66	Afadin	E9Q852
Afg3l2	-1.4831	0.63	0.0034	-0.45	0.0076	0.13	0.8	0.18	0.61	AFG3-like protein 2	Q8JZQ2
Agap2	-1.2053	1.00	0.3	-1.83	0.055	-1.01	0.21	-0.83	0.34	Arf-GAP with GTPase, ANK repeat and PH domain-containing protein 2	Q3UHD9
Agap3	-0.0168	1.08	0.38	-0.31	0.76	0.37	0.62	0.77	0.54	Arf-GAP with GTPase, ANK repeat and PH domain-containing protein 3	F8VQE9
Agfg1	-0.0504	0.60	0.056	-0.07	0.11	0.24	0.49	0.53	0.69	Arf-GAP domain and FG repeat-containing protein 1 (Fragment)	A0A087WR52

Table S2: Differentially expressed proteins in cerebral cortex *(continued)*

gene	$\pi_{TW/EW} \times \pi_{TF/TW}$	log2FC TW/EW	padj TW/EW	log2FC TF/TW	padj TF/TW	log2FC EF/EW	padj EF/EW	log2FC TF/EW	padj TF/EW	description	accession
Agk	0.0002	0.21	0.94	0.12	0.47	0.14	0.83	0.34	0.27	Acylglycerol kinase, mitochondrial	Q9ESW4
Agl	-0.0048	-0.11	0.52	0.24	0.23	-0.13	0.88	0.13	0.47	4-alpha-glucanotransferase	F8VPN4
Ago1	-0.0001	-0.26	0.91	0.12	0.91	-1.15	0.32	-0.14	0.72	Protein argonaute-1	A0A6I8MX18
Ago2	0.0000	0.19	1	-5.07	0.25	-0.30	0.92	-4.88	0.15	Protein argonaute-2	Q8CJG0
Agpat5	-11.2876	2.71	0.0460	-2.41	0.051	-0.85	0.47	0.31	0.81	1-acyl-sn-glycerol-3-phosphate acyltransferase epsilon	F8WGD9
Ahcy	-0.0199	-0.34	0.0390	0.12	0.45	-0.15	0.39	-0.22	0.14	Adenosylhomocysteinase	P50247
Ahcyl1	-0.0346	-0.25	0.0270	0.34	0.55	-0.14	0.28	0.09	0.94	S-adenosylhomocysteine hydrolase-like protein 1	Q80SW1
Ahnak	-0.3071	0.43	0.45	-0.83	0.0033	-1.30	0.26	-0.40	0.9	AHNAK nucleoprotein (desmoyokin)	E9Q616
Ahsa1	-0.0094	0.13	0.58	-0.32	0.11	-0.26	0.28	-0.19	0.36	Activator of 90 kDa heat shock protein ATPase homolog 1	Q8BK64
Aifm1	-0.3861	0.45	0.12	-0.60	0.0280	0.17	0.58	-0.15	0.31	Apoptosis-inducing factor 1, mitochondrial	B1AU25
Aifm3	-3.5424	0.41	0.0010	-0.72	0.0001	0.00	0.58	-0.31	0.0004	Apoptosis-inducing factor 3	Q3TY86
Aimp1	0.0008	0.13	0.47	0.10	0.64	0.12	0.63	0.23	0.19	Aminoacyl tRNA synthase complex-interacting multifunctional protein 1	P31230
Aimp2	0.0000	0.09	0.85	0.00	0.6	-0.05	0.85	0.08	0.73	Aminoacyl tRNA synthase complex-interacting multifunctional protein 2	Q8R010
Aip	-0.0046	-0.09	0.75	0.43	0.11	0.26	0.57	0.33	0.22	AH receptor-interacting protein	O08915
Ajm1	-3.6457	0.80	0.16	-1.73	0.0005	0.19	0.41	-0.93	0.0440	Apical junction component 1 homolog	A2AJA9
Ak1	0.0192	-0.07	0.5	-0.68	0.0460	-0.95	0.44	-0.75	0.43	Adenylate kinase isoenzyme 1	Q9R0Y5
AK157302	-2.3408	0.70	0.17	-1.61	0.0020	-0.46	0.24	-0.91	0.095	cDNA sequence AK157302	I3ITR1
Ak2	-0.0292	-0.45	0.39	0.41	0.41	0.11	0.71	-0.03	0.98	Adenylate kinase 2, mitochondrial	Q9WTP6
Ak3	0.0011	-0.04	0.9	-0.46	0.0460	-0.49	0.082	-0.50	0.0110	GTP:AMP phosphotransferase AK3, mitochondrial	Q9WTP7
Ak4	0.0013	-0.02	0.76	-0.60	0.12	-0.14	0.86	-0.62	0.25	Adenylate kinase 4, mitochondrial	Q9WUR9
Ak5	-0.0078	0.30	0.43	-0.38	0.65	-0.58	0.27	-0.07	0.77	Adenylate kinase isoenzyme 5	Q920P5
Akap5	0.0000	0.01	0.44	0.05	0.89	0.02	0.31	0.06	0.52	A-kinase anchor protein 5	D3YVF0
Akr1a1	0.0018	-0.04	0.64	-0.27	0.14	0.15	0.17	-0.32	0.36	Aldo-keto reductase family 1 member A1	Q9JII6
Akr1b1	-0.0124	-0.52	0.51	0.27	0.5	-0.57	0.0010	-0.24	0.99	Aldo-keto reductase family 1 member B1	P45376
Akr7a2	-0.0010	-0.48	0.28	0.03	0.75	-0.46	0.59	-0.45	0.42	Aflatoxin B1 aldehyde reductase member 2	Q8CG76
Alb	-0.3687	-0.71	0.0001	0.18	0.19	-0.44	0.0001	-0.53	0.0001	Albumin	P07724
Aldh18a1	-0.0001	0.10	0.74	-0.07	0.82	0.57	0.22	0.04	1	Delta-1-pyrroline-5-carboxylate synthase	Q9Z110
Aldh1l1	0.0090	-0.16	0.19	-0.18	0.37	0.15	0.92	-0.35	0.0010	Cytosolic 10-formyltetrahydrofolate dehydrogenase	Q8R0Y6
Aldh2	0.0077	-0.37	0.69	-0.30	0.37	-0.48	0.29	-0.67	0.2	Aldehyde dehydrogenase, mitochondrial	A0A0G2JEU1
Aldh3a2	-0.0262	0.38	0.062	-0.19	0.5	0.15	0.41	0.20	0.98	Aldehyde dehydrogenase	B1ATI0

Table S2: Differentially expressed proteins in cerebral cortex *(continued)*

gene	$\pi_{TW/EW} \times \pi_{TF/TW}$	log2FC TW/EW	padj TW/EW	log2FC TF/TW	padj TF/TW	log2FC EF/EW	padj EF/EW	log2FC TF/EW	padj TF/EW	description	accession
Aldh3b1	-1.9819	1.19	0.11	-1.29	0.0450	0.42	0.22	-0.09	0.78	Aldehyde dehydrogenase family 3 member B1	Q80VQ0
Aldh5a1	-0.0280	0.10	0.19	-0.29	0.0460	-0.33	0.1	-0.19	0.61	Succinate-semialdehyde dehydrogenase, mitochondrial	Q8BWF0
Aldh6a1	0.0028	-0.26	0.0260	-0.07	0.8	-0.16	0.23	-0.34	0.0020	Methylmalonate-semialdehyde dehydrogenase [acylating], mitochondrial	Q9EQ20
Aldh7a1	0.0396	-0.89	0.21	-0.20	0.47	-0.39	0.37	-1.09	0.0040	Alpha-aminoadipic semialdehyde dehydrogenase	Q9DBF1
Aldoa	-0.0010	0.08	0.47	-0.08	0.35	-0.03	0.96	0.00	0.79	Fructose-bisphosphate aldolase	A6ZI44
Aldoc	-0.0005	-0.14	0.35	0.01	0.14	-0.19	0.19	-0.13	0.27	Fructose-bisphosphate aldolase C	P05063
Alyref	-0.0160	0.32	0.64	-0.65	0.4	-0.32	0.46	-0.33	0.24	THO complex subunit 4	O08583
Amer2	-0.0008	0.32	0.43	-0.10	0.85	-0.36	0.21	0.22	0.42	APC membrane recruitment protein 2	Q8CCJ4
Amot	-0.0066	0.32	0.68	-0.46	0.54	0.17	0.5	-0.13	0.88	Angiomotin	Q8VHG2
Ampd2	0.0004	0.12	0.84	0.30	0.69	0.03	0.93	0.42	0.52	AMP deaminase	A2AE27
Amph	0.0000	0.00	0.47	0.36	0.27	0.08	0.44	0.36	0.0360	Amphiphysin	A0A0G2JEG8
Amy2	0.0165	-0.70	0.076	-0.23	0.81	-0.33	0.4	-0.93	0.09	Pancreatic alpha-amylase	P00688
Ank1	0.0874	-0.45	0.32	-0.51	0.17	-0.69	0.0250	-0.97	0.0005	Ankyrin-1	A0A0R4J1N7
Ank2	-17.3302	0.97	0.0003	-1.24	0.0001	0.54	0.0070	-0.27	0.14	Ankyrin-2	S4R2F3
Ank3	-1.7575	1.11	0.12	-1.23	0.0400	0.40	0.42	-0.11	0.59	Ankyrin-3 (Fragment)	A0A087WNU5
Ank3	-14.3409	2.74	0.0140	-2.17	0.05	0.19	0.92	0.57	0.59	Ankyrin-3	S4R165
Ank3	-0.1256	0.64	0.39	-0.73	0.22	0.27	0.78	-0.09	0.94	Ankyrin-3	W4VSQ0
Ankrd29	-2.0992	-0.58	0.0250	0.80	0.0015	0.55	0.23	0.22	0.45	Ankyrin repeat domain 29	D3YTU5
Ankrd63	-0.0409	0.47	0.67	-1.01	0.32	0.28	0.77	-0.54	0.61	Ankyrin repeat domain-containing protein 63	A2ARS0
Anks1b	-12.0282	1.79	0.054	-2.46	0.0070	-0.35	0.36	-0.68	0.1	Ankyrin repeat and sterile alpha motif domain-containing protein 1B	S4R2Q2
Anpep	0.0000	-0.38	0.33	-0.01	1	-0.02	0.97	-0.38	0.19	Aminopeptidase N	P97449
Anxa2	0.4566	-0.81	0.6	-4.10	0.24	-3.94	0.84	-4.91	0.26	Annexin (Fragment)	B0V2N7
Anxa3	-0.0054	-0.42	0.2	0.11	0.68	-0.03	0.97	-0.31	0.4	Annexin A3	O35639
Anxa5	0.2255	-0.34	0.0100	-0.32	0.092	-0.59	0.0110	-0.66	0.0030	Annexin A5	P48036
Anxa6	0.0021	-0.52	0.0110	-0.04	0.89	-0.52	0.12	-0.55	0.0300	Annexin	F8WIT2
Anxa7	0.0008	-0.27	0.61	-0.14	0.8	-0.09	0.96	-0.41	0.39	Annexin	A0A2C9F2D2
Ap1g1	0.5222	0.56	0.57	1.91	0.0100	1.20	0.13	2.47	0.0010	AP-1 complex subunit gamma	Q8CBB7
Ap1g2	-0.4779	-0.73	0.22	0.81	0.059	0.55	0.11	0.08	0.89	AP-1 complex subunit gamma-like 2	O88512
Ap1m1	0.0015	0.46	0.48	0.08	0.75	-0.73	0.22	0.54	0.49	AP-1 complex subunit mu-1	P35585
Ap1s1	0.4280	0.48	0.0280	0.47	0.06	0.02	0.9	0.95	0.0280	AP-1 complex subunit sigma-1A	P61967
Ap2a1	-0.0414	-0.14	0.56	0.70	0.0210	0.18	0.27	0.56	0.0010	AP-2 complex subunit alpha-1	P17426
Ap2a2	0.2007	0.07	0.15	0.87	0.0001	0.35	0.0330	0.94	0.0001	AP-2 complex subunit alpha-2	P17427

Table S2: Differentially expressed proteins in cerebral cortex *(continued)*

gene	$\pi_{TW/EW} \times \pi_{TF/TW}$	log2FC TW/EW	padj TW/EW	log2FC TF/TW	padj TF/TW	log2FC EF/EW	padj EF/EW	log2FC TF/EW	padj TF/EW	description	accession
Ap2b1	-0.1565	-0.17	0.54	0.86	0.0001	0.27	0.27	0.69	0.0001	AP complex subunit beta	H3BKM0
Ap2m1	-2.4140	0.50	0.0001	-0.33	0.0002	-0.29	0.0048	0.17	0.29	AP-2 complex subunit mu	P84091
Ap2s1	-0.0882	-0.30	0.0080	0.44	0.48	0.25	0.0120	0.15	0.5	AP-2 complex subunit sigma	P62743
Ap3b2	0.0653	0.19	0.18	0.56	0.15	0.06	0.77	0.75	0.0001	AP-3 complex subunit beta-2	Q9JME5
Ap3d1	-4.4061	2.46	0.3	-3.72	0.12	0.38	0.69	-1.26	0.58	AP-3 complex subunit delta-1	O54774
Ap3m1	0.0091	0.37	0.7	0.54	0.51	-0.61	0.55	0.91	0.48	AP-3 complex subunit mu-1	A0A286YDZ6
Ap3m2	0.3689	1.15	0.0067	0.45	0.47	-0.24	0.58	1.60	0.0030	AP-3 complex subunit mu-2	Q8R2R9
Ap3s2	-0.0316	0.85	0.35	-0.38	0.61	1.26	0.21	0.48	0.83	AP-3 complex subunit sigma-2	Q8BSZ2
Apba1	-0.4075	-0.60	0.34	2.01	0.19	0.23	0.67	1.41	0.39	Amyloid-beta A4 precursor protein-binding family A member 1	B2RUJ5
Apba2	-3.1607	2.52	0.14	-2.37	0.24	0.88	0.28	0.15	0.64	Amyloid-beta A4 precursor protein-binding family A member 2	A0A0U1RPM0
Apc	-3.5419	1.26	0.091	-1.32	0.0090	0.09	0.78	-0.05	0.76	Adenomatosis polyposis coli	B2RUG9
Apeh	-0.0004	-0.24	0.094	0.03	0.89	-0.12	0.4	-0.22	0.24	Acyl-peptide hydrolase (Fragment)	A0A0R4J107
Apex1	-0.0001	0.28	0.6	-0.09	0.97	-0.06	0.94	0.19	0.64	DNA-(apurinic or apyrimidinic site) endonuclease (Fragment)	F6QA74
Api5	0.0002	0.12	0.31	0.01	0.56	-0.24	0.11	0.13	0.47	Apoptosis inhibitor 5	O35841
Aplp1	0.0020	0.34	0.65	0.16	0.64	0.59	0.23	0.51	0.4	Amyloid-like protein 1	A0A0R4IZZ1
Apoa1	0.0178	-0.76	0.57	-0.31	0.49	-0.79	0.29	-1.07	0.29	Apolipoprotein A-I	Q00623
Apoe	0.0198	-0.10	0.5	-0.36	0.0150	0.19	0.47	-0.46	0.0390	Apolipoprotein E	P08226
Apoo	-0.0256	0.52	0.32	-0.33	0.5	-1.22	0.35	0.20	0.84	MICOS complex subunit Mic26	Q9DCZ4
App	0.0004	0.23	0.94	0.42	0.7	1.45	0.3	0.65	0.69	Amyloid-beta A4 protein	A0A2I3BPT1
Appl1	0.0078	0.28	0.12	0.11	0.53	0.13	0.6	0.39	0.0320	DCC-interacting protein 13-alpha	Q8K3H0
Appl2	0.0436	0.22	0.34	0.30	0.0390	0.87	0.0004	0.52	0.0190	DCC-interacting protein 13-beta	Q8K3G9
Aqp4	-0.0001	-0.23	0.12	0.02	0.95	-0.43	0.19	-0.21	0.094	Aquaporin-4	A0A0R4J0Z3
Arap2	-59.2207	7.16	0.12	-8.47	0.087	-1.24	0.58	-1.31	0.52	Arf-GAP with Rho-GAP domain, ANK repeat and PH domain-containing protein 2	E9QP44
Arc	-0.2436	0.75	0.45	-1.34	0.2	0.35	0.54	-0.59	0.32	Activity-regulated cytoskeleton-associated protein	Q9WV31
Arcn1	0.0480	0.51	0.0330	0.56	0.77	0.38	0.16	1.08	0.064	Coatomer subunit delta	Q5XJY5
Arf4	0.0036	-0.05	0.8	-0.45	0.0220	-0.23	0.25	-0.50	0.0150	ADP-ribosylation factor 4	P61750
Arfgap1	-0.0222	0.25	0.26	-0.21	0.19	0.34	0.88	0.04	0.75	ADP-ribosylation factor GTPase-activating protein 1	Q3TGS9
Arfgap2	-9.9405	2.38	0.14	-3.15	0.0280	1.82	0.46	-0.78	0.82	ADP-ribosylation factor GTPase-activating protein 2	Q99K28
Arfgef2	0.0016	0.32	0.48	0.09	0.67	0.34	0.33	0.41	0.27	Brefeldin A-inhibited guanine nucleotide-exchange protein 2	A2A5R2

Table S2: Differentially expressed proteins in cerebral cortex *(continued)*

gene	$\pi_{TW/EW} \times \pi_{TF/TW}$	log2FC TW/EW	padj TW/EW	log2FC TF/TW	padj TF/TW	log2FC EF/EW	padj EF/EW	log2FC TF/EW	padj TF/EW	description	accession
Arfgef3	-0.0004	0.11	0.58	-0.12	0.73	0.04	0.85	0.00	0.98	Brefeldin A-inhibited guanine nucleotide-exchange protein 3	Q3UGY8
Arhgap12	0.1429	1.25	0.14	0.31	0.37	-2.57	0.34	1.56	0.0160	Rho GTPase-activating protein 12	A0A0A0MQ95
Arhgap17	-0.0984	0.32	0.16	-0.34	0.073	0.02	0.94	-0.02	0.92	Rho GTPase-activating protein 17	E9QAJ9
Arhgap21	-0.0176	1.92	0.34	-0.62	0.93	0.47	0.86	1.30	0.16	Rho GTPase-activating protein 21	A0A0A0MQE8
Arhgap23	-0.0001	0.11	0.76	-0.13	0.86	-0.53	0.33	-0.02	0.97	Rho GTPase-activating protein 23	B1AQY2
Arhgap26	0.0186	0.30	0.0020	0.38	0.87	0.45	0.12	0.69	0.0470	Rho GTPase-activating protein 26	E9QAQ3
Arhgap32	-2.0184	0.88	0.088	-1.21	0.0160	-0.31	0.45	-0.33	0.64	Rho GTPase-activating protein 32	Q811P8
Arhgap35	-0.0559	0.19	0.17	-0.34	0.075	-0.09	0.97	-0.15	0.84	Rho GTPase-activating protein 35	Q91YM2
Arhgap39	-2.0322	0.83	0.46	-2.42	0.0010	-0.48	0.34	-1.59	0.0030	Rho GTPase-activating protein 39	H7BX46
Arhgap44	-0.2421	0.30	0.0250	-0.34	0.0330	-0.27	0.27	-0.04	0.98	Rho GTPase-activating protein 44	Q5SSM3
Arhgdia	-0.0018	-0.48	0.0002	0.02	0.89	-0.22	0.11	-0.46	0.0001	Rho GDP-dissociation inhibitor 1	Q99PT1
Arhgef11	0.0011	-0.15	0.74	-0.33	0.67	0.41	0.67	-0.49	0.49	Rho guanine nucleotide exchange factor (GEF) 11 (Fragment)	E9Q0A3
Arhgef12	0.0893	-0.87	0.53	-3.45	0.78	0.67	0.83	-4.32	0.32	Rho guanine nucleotide exchange factor 12	F8VQN6
Arhgef2	-2.4190	0.72	0.065	-1.23	0.0050	-0.31	0.37	-0.51	0.23	Guanine nucleotide exchange factor H1	H3BKH9
Arhgef26	-0.7939	2.06	0.22	-1.69	0.45	-0.07	0.93	0.38	0.8	Rho guanine nucleotide exchange factor (GEF) 26	D3YYY8
Arhgef7	-0.1834	1.78	0.12	-0.22	0.31	1.59	0.21	1.56	0.17	Rho guanine nucleotide exchange factor 7	Q9ES28
Arhgef9	-0.1215	0.51	0.25	-0.62	0.23	-0.01	0.97	-0.11	0.57	Collybistin	B1AXI6
Arid1b	0.0000	0.30	0.6	0.07	1	0.20	0.7	0.37	0.46	AT-rich interactive domain-containing protein 1B	A0A338P6U8
Arl8b	-0.0039	0.36	0.2	-0.08	0.64	0.32	0.52	0.28	0.43	ADP-ribosylation factor-like protein 8B	Q9CQW2
Armc1	-0.5358	-0.59	0.052	0.54	0.0490	-0.61	0.25	-0.05	0.91	Armadillo repeat-containing protein 1	Q9D7A8
Armc10	-0.0006	-1.00	0.34	0.27	0.99	0.20	0.62	-0.74	0.47	Armadillo repeat-containing protein 10	Q9D0L7
Armc6	0.0000	0.04	0.88	-0.08	0.9	0.15	0.47	-0.03	0.93	Armadillo repeat-containing protein 6	Q8BNU0
Armcx3	-0.0301	0.84	0.59	-0.55	0.52	-0.45	0.6	0.29	0.93	Armadillo repeat-containing X-linked protein 3	Q8BHS6
Arpc1a	-0.0140	-0.34	0.0160	0.07	0.47	0.06	0.62	-0.28	0.052	Actin-related protein 2/3 complex subunit 1A	Q9R0Q6
Arpc2	0.0010	-0.26	0.051	-0.02	0.7	0.06	0.58	-0.29	0.0048	Actin-related protein 2/3 complex subunit 2	Q9CVB6
Arpc3	-0.0835	-0.36	0.0240	0.18	0.16	0.03	0.92	-0.18	0.21	Actin-related protein 2/3 complex subunit 3	Q9JM76
Arpc4	-0.0057	-0.32	0.11	0.07	0.54	-0.06	0.73	-0.25	0.0070	Actin-related protein 2/3 complex subunit 4	P59999
Arpc5	-0.0001	-0.31	0.79	0.04	0.82	-0.05	0.79	-0.27	0.5	Actin-related protein 2/3 complex subunit 5	Q9CPW4

Table S2: Differentially expressed proteins in cerebral cortex *(continued)*

gene	$\pi_{TW/EW} \times \pi_{TF/TW}$	log2FC TW/EW	padj TW/EW	log2FC TF/TW	padj TF/TW	log2FC EF/EW	padj EF/EW	log2FC TF/EW	padj TF/EW	description	accession
Arpc5l	0.0005	-0.22	0.0030	-0.01	0.81	0.02	0.97	-0.23	0.0030	Actin-related protein 2/3 complex subunit 5-like protein	Q9D898
Arrb1	-0.0016	2.53	0.98	-2.65	0.94	-0.16	0.87	-0.12	0.8	Beta-arrestin-1	J3QNU6
Arsb	-0.0013	-0.85	0.58	0.09	0.85	-2.50	0.16	-0.76	0.62	Arylsulfatase B	A0A0R4J138
Arvcf	0.0016	0.57	0.8	0.57	0.89	1.10	0.42	1.14	0.5	Armadillo repeat protein deleted in velo-cardio-facial syndrome homolog	P98203
Arxes2	0.0201	0.15	0.62	0.61	0.087	-0.09	0.85	0.75	0.0280	Adipocyte-related X-chromosome expressed sequence 2	C0HK80
Asap1	0.0072	0.76	0.0460	0.05	0.72	0.56	0.39	0.81	0.14	Arf-GAP with SH3 domain, ANK repeat and PH domain-containing protein 1	E9QN63
Asap2	-0.1366	-0.50	0.22	0.54	0.17	-0.83	0.12	0.04	0.79	Arf-GAP with SH3 domain, ANK repeat and PH domain-containing protein 2	E9PX52
Asrgl1	0.0000	-0.22	1	-0.16	0.1	-0.09	0.7	-0.38	0.0015	Isoaspartyl peptidase/L-asparaginase	Q8C0M9
Ass1	-0.0003	0.04	0.74	-0.17	0.47	-0.23	0.11	-0.13	0.26	Argininosuccinate synthase	P16460
Astn1	-0.0046	-0.23	0.45	0.25	0.59	0.15	0.76	0.02	0.9	Astrotactin-1	Q61137
Astn2	0.0000	-0.01	0.87	0.13	0.59	-0.06	0.83	0.12	0.72	Astrotactin-2	Q80Z10
Atad1	-0.1533	-0.39	0.35	0.64	0.0450	0.17	0.8	0.25	0.18	Outer mitochondrial transmembrane helix translocase	A0A494B9L7
Atad3	-1.8814	0.47	0.0190	-0.74	0.0007	-0.23	0.44	-0.27	0.17	ATPase family AAA domain-containing protein 3	Q925I1
Atat1	-0.0129	0.14	0.47	-0.51	0.28	0.38	0.38	-0.37	0.67	Alpha-tubulin N-acetyltransferase 1	Q8K341
Atg2b	-0.8207	0.58	0.0300	-0.58	0.0250	0.12	0.51	0.00	0.98	Autophagy-related protein 2 homolog B	Q80XK6
Atg3	-0.2542	-1.18	0.38	1.22	0.38	0.39	0.55	0.04	1	Ubiquitin-like-conjugating enzyme ATG3	Q9CPX6
Atic	-0.0004	-0.41	0.45	0.07	0.91	-0.43	0.25	-0.34	0.17	Bifunctional purine biosynthesis protein ATIC	Q9CWJ9
Atp1a3	-0.0114	-0.23	0.0140	0.12	0.6	-0.12	0.0070	-0.10	0.0014	Sodium/potassium-transporting ATPase subunit alpha	A0A0G2JGX4
Atp1b1	0.0038	-0.16	0.34	-0.06	0.14	-0.14	0.28	-0.22	0.0110	Sodium/potassium-transporting ATPase subunit beta-1	P14094
Atp1b2	-0.0048	-0.47	0.0270	0.05	0.74	-0.45	0.14	-0.42	0.0046	Sodium/potassium-transporting ATPase subunit beta-2	P14231
Atp1b3	0.0175	-0.29	0.19	-0.39	0.61	-0.23	0.31	-0.67	0.13	Sodium/potassium-transporting ATPase subunit beta-3	P97370
Atp2a2	-0.0407	0.21	0.15	-0.19	0.058	-0.09	0.43	0.03	0.92	Sarcoplasmic/endoplasmic reticulum calcium ATPase 2	O55143
Atp2b1	0.0025	-0.08	0.29	-0.04	0.0350	-0.07	0.44	-0.13	0.41	Plasma membrane calcium-transporting ATPase 1	G5E829
Atp2b4	0.0039	-0.25	0.17	-0.07	0.51	-0.32	0.053	-0.32	0.0020	Calcium-transporting ATPase	E9Q828
Atp5c1	-21.0505	1.08	0.0005	-1.48	0.0001	-0.12	0.76	-0.40	0.37	ATP synthase subunit gamma	A2AKU9

Table S2: Differentially expressed proteins in cerebral cortex *(continued)*

gene	$\pi_{TW/EW}\times \pi_{TF/TW}$	log2FC TW/EW	padj TW/EW	log2FC TF/TW	padj TF/TW	log2FC EF/EW	padj EF/EW	log2FC TF/EW	padj TF/EW	description	accession
Atp5f1b	-2.1888	0.38	0.0001	-0.36	0.0001	-0.07	0.056	0.01	0.34	ATP synthase subunit beta, mitochondrial	P56480
Atp5f1d	-0.0076	0.25	0.5	-0.24	0.38	0.27	0.18	0.01	0.89	ATP synthase subunit delta, mitochondrial	Q9D3D9
Atp5f1e	0.0000	-0.09	0.81	0.03	0.93	-0.01	0.48	-0.07	0.81	ATP synthase subunit epsilon, mitochondrial	P56382
Atp5me	-0.7023	0.47	0.0020	-0.33	0.0210	0.17	0.95	0.14	0.17	ATP synthase subunit e, mitochondrial	Q06185
Atp5mf	-0.2560	0.47	0.083	-0.41	0.059	0.23	0.48	0.06	0.77	ATP synthase subunit f, mitochondrial	P56135
Atp5mg	-0.0649	0.30	0.088	-0.24	0.14	0.16	0.77	0.05	0.71	ATP synthase subunit g, mitochondrial	Q9CPQ8
Atp5pb	-0.0868	0.16	0.11	-0.30	0.0130	0.00	0.7	-0.14	0.26	ATP synthase F(0) complex subunit B1, mitochondrial	Q9CQQ7
Atp5pd	-0.0264	0.18	0.13	-0.18	0.12	0.15	0.17	0.00	0.8	ATP synthase subunit d, mitochondrial	Q9DCX2
Atp5po	-9.7920	0.72	0.0001	-0.85	0.0001	-0.04	0.32	-0.14	0.43	ATP synthase subunit O, mitochondrial	Q9DB20
Atp6ap2	-0.0004	-0.09	0.76	0.16	0.6	0.13	0.7	0.06	0.83	Renin receptor	Q9CYN9
Atp6v0a1	-0.0449	-0.42	0.057	0.21	0.39	0.06	0.55	-0.22	0.25	V-type proton ATPase subunit a	K3W4T3
Atp6v0d1	-0.5401	-0.29	0.0210	0.37	0.0010	0.09	0.95	0.08	0.24	V-type proton ATPase subunit d 1	P51863
Atp6v1a	-0.0115	-0.39	0.29	0.13	0.38	0.10	0.66	-0.25	0.086	V-type proton ATPase catalytic subunit A	P50516
Atp6v1b2	-0.0331	-0.31	0.23	0.32	0.3	0.13	0.81	0.01	0.79	V-type proton ATPase subunit B, brain isoform	P62814
Atp6v1c1	-0.3705	-0.41	0.0044	0.40	0.11	0.17	0.6	-0.01	0.45	V-type proton ATPase subunit C 1	Q9Z1G3
Atp6v1d	-0.0012	0.03	0.4	-0.10	0.095	-0.03	0.8	-0.07	0.4	V-type proton ATPase subunit D	P57746
Atp6v1e1	-0.5518	-0.27	0.0030	0.27	0.0010	0.24	0.8	0.00	0.98	V-type proton ATPase subunit E 1	P50518
Atp6v1f	-0.2578	-0.48	0.074	0.42	0.074	0.12	0.46	-0.06	0.64	V-ATPase 14 kDa subunit	A0A0N4SVE1
Atp6v1g1	-0.0003	-0.36	0.88	0.34	0.91	0.34	0.51	-0.02	0.77	V-type proton ATPase subunit G 1	Q9CR51
Atp6v1g2	-0.0005	-0.15	0.56	0.12	0.79	0.28	0.46	-0.03	0.36	V-type proton ATPase subunit G 2	Q9WTT4
Atp6v1h	-0.0195	-0.36	0.0310	0.13	0.53	-0.08	0.23	-0.23	0.0006	V-type proton ATPase subunit H	A0A0A6YX18
Atp8a1	-1.5390	-0.45	0.0010	0.38	0.0010	0.06	1	-0.07	0.53	Phospholipid-transporting ATPase IA	P70704
Atpif1	0.0041	0.12	0.25	0.13	0.37	0.17	0.73	0.25	0.0010	ATP synthase F1 subunit epsilon	E9PV44
Atxn2	-0.0011	0.56	0.0200	-0.02	0.88	-0.05	0.53	0.54	0.0070	Ataxin-2	E9QM77
Atxn2l	0.0147	0.34	0.13	0.22	0.6	-0.03	0.73	0.56	0.73	Ataxin-2-like protein	A0A0U1RPL0
Auh	0.0530	0.37	0.0100	0.18	0.4	0.66	0.0001	0.55	0.0002	Methylglutaconyl-CoA hydratase, mitochondrial	Q9JLZ3
Avl9	0.0030	-0.51	0.38	-0.34	0.91	-0.60	0.2	-0.85	0.0280	Late secretory pathway protein AVL9 homolog	Q80U56
Bag2	-0.2976	0.48	0.38	-0.95	0.0280	0.02	1	-0.47	0.094	BAG family molecular chaperone regulator 2	Q91YN9
Bag4	0.0109	0.12	0.52	0.32	0.1	0.24	0.29	0.44	0.06	BAG family molecular chaperone regulator 4	Q8CI61
Bag6	-2.9946	2.25	0.33	-4.46	0.24	1.37	0.56	-2.21	0.62	BCL2-associated athanogene 6	Q3UF95

Table S2: Differentially expressed proteins in cerebral cortex *(continued)*

gene	$\pi_{\mathrm{TW/EW}} \times \pi_{\mathrm{TF/TW}}$	log2FC TW/EW	padj TW/EW	log2FC TF/TW	padj TF/TW	log2FC EF/EW	padj EF/EW	log2FC TF/EW	padj TF/EW	description	accession
Baiap2	-2.8224	0.56	0.0010	-0.56	0.0010	0.55	0.0002	0.00	1	Brain-specific angiogenesis inhibitor 1-associated protein 2	B1AZ46
Basp1	-0.0014	0.03	0.88	-0.37	0.0055	-0.03	0.27	-0.34	0.0030	Brain acid soluble protein 1	Q91XV3
Bcan	0.3615	-0.51	0.0001	-0.20	0.13	-0.91	0.0009	-0.71	0.0010	Brevican core protein	Q61361
Bcap29	-0.0322	-0.08	0.56	0.63	0.0029	0.22	0.09	0.55	0.0010	B-cell receptor-associated protein 29	Q61334
Bcar1	-0.0004	0.25	0.69	-0.12	0.81	-0.37	0.56	0.13	0.84	Breast cancer anti-estrogen resistance protein 1	Q61140
Bcas1	-0.0602	0.78	0.0011	-0.23	0.77	0.20	0.61	0.56	0.0003	Breast carcinoma-amplified sequence 1 homolog	Q80YN3
Bcas2	-0.3374	2.08	0.73	-3.15	0.42	-1.57	0.52	-1.07	0.53	Pre-mRNA-splicing factor SPF27	Q9D287
Bcas3	-0.0522	0.32	0.17	-0.23	0.12	0.03	0.75	0.09	0.7	Bcas3 protein	Q0VF62
Bckdha	-0.0006	-0.18	0.54	0.08	0.69	-0.22	0.52	-0.10	0.78	2-oxoisovalerate dehydrogenase subunit alpha, mitochondrial	P50136
Bckdk	-0.2471	0.28	0.26	-0.70	0.0070	0.36	0.095	-0.41	0.0062	Protein-serine/threonine kinase	A0A0U1RNL7
Bcl2l13	-0.4107	0.37	0.32	-1.90	0.066	-0.73	0.38	-1.53	0.13	Bcl-2-like protein 13	P59017
Bcr	-0.0415	0.19	0.51	-0.57	0.0490	-0.40	0.25	-0.39	0.21	Breakpoint cluster region protein	Q6PAJ1
Bdh1	-0.0077	0.26	0.16	-0.12	0.49	0.03	0.86	0.13	0.3	D-beta-hydroxybutyrate dehydrogenase, mitochondrial	Q80XN0
Begain	-0.4486	0.79	0.49	-1.57	0.068	-0.42	0.69	-0.78	0.2	Brain-enriched guanylate kinase-associated protein	F8WIG2
Blvrb	-0.5537	-0.74	0.0030	0.36	0.15	0.19	0.35	-0.38	0.0360	Flavin reductase (NADPH)	Q923D2
Bola2	-0.0372	-0.50	0.0040	0.14	0.6	-0.19	0.41	-0.36	0.0040	BolA-like protein 2	Q8BGS2
Bphl	-3.7806	0.65	0.0390	-1.37	0.0010	-0.46	0.35	-0.73	0.26	Valacyclovir hydrolase	Q8R164
Bpnt1	0.0003	-0.30	0.22	-0.02	0.86	-1.16	0.052	-0.32	0.0440	3'(2'),5'-bisphosphate nucleotidase 1	A0A1B0GRV0
Brinp1	0.0000	0.01	0.72	-0.06	0.62	0.30	0.092	-0.05	0.73	BMP/retinoic acid-inducible neural-specific protein 1	Q920P3
Brk1	0.0000	-0.06	0.36	0.00	0.9	0.02	0.8	-0.05	0.37	Protein BRICK1	Q91VR8
Brsk1	-1.8558	0.94	0.0310	-0.78	0.0210	-0.38	0.36	0.16	0.71	Serine/threonine-protein kinase BRSK1	D3Z5P0
Brsk2	-9.1398	2.01	0.24	-6.04	0.061	-3.16	0.15	-4.03	0.056	Serine/threonine-protein kinase BRSK2 (Fragment)	G3UXM5
Bsg	-0.0073	-0.17	0.26	0.14	0.3	-0.11	0.6	-0.03	0.81	Basigin	K3W4Q8
Bsn	-40.2529	1.28	0.0001	-2.04	0.0001	-0.53	0.0010	-0.77	0.0001	Protein bassoon	O88737
Btbd17	-0.0005	0.35	0.32	-0.01	0.53	0.76	0.0240	0.34	0.7	BTB/POZ domain-containing protein 17	Q9DB72
Btbd8	-0.0504	0.63	0.26	-0.30	0.35	0.26	0.59	0.33	0.82	BTB/POZ domain-containing protein 8	A0A1D5RL96
Bub3	-2.4435	-3.56	0.47	4.23	0.32	-3.14	0.62	0.67	0.76	Mitotic checkpoint protein BUB3	A0A140LHA2
C1qc	-0.0212	0.45	0.73	-0.80	0.37	-0.20	0.48	-0.34	0.55	Complement C1q subcomponent subunit C	Q02105
C1qtnf4	-0.2373	1.29	0.31	-0.55	0.22	0.93	0.53	0.74	0.78	Complement C1q tumor necrosis factor-related protein 4	Q8R066

Table S2: Differentially expressed proteins in cerebral cortex *(continued)*

gene	$\pi_{TW/EW} \times \pi_{TF/TW}$	log2FC TW/EW	padj TW/EW	log2FC TF/TW	padj TF/TW	log2FC EF/EW	padj EF/EW	log2FC TF/EW	padj TF/EW	description	accession
C2cd2l	0.0002	-0.03	0.9	-0.23	0.27	-0.15	0.23	-0.26	0.067	Phospholipid transfer protein C2CD2L	Q80X80
C2cd4c	0.0021	-0.07	0.37	-0.30	0.58	-0.56	0.0400	-0.37	0.089	C2 calcium-dependent domain-containing protein 4C	Q5HZI2
Ca2	0.0007	-0.25	0.14	-0.04	0.83	-0.68	0.0001	-0.28	0.0100	Carbonic anhydrase 2	P00920
Cab39	-0.0008	0.17	0.14	-0.03	0.67	-0.36	0.061	0.14	0.13	Calcium-binding protein 39	Q06138
Cacna1a	-1.9055	0.92	0.23	-1.91	0.0200	-0.54	0.19	-0.98	0.0190	Voltage-dependent P/Q-type calcium channel subunit alpha	A0A571BET0
Cacna1b	0.0002	0.10	0.86	0.18	0.69	-0.02	0.94	0.28	0.45	Voltage-dependent N-type calcium channel subunit alpha	A2AIR7
Cacna1e	-0.5197	0.58	0.19	-0.98	0.054	-0.16	0.76	-0.40	0.4	Voltage-dependent R-type calcium channel subunit alpha	A0A087WS83
Cacna2d1	-0.0082	0.17	0.71	-0.38	0.14	-0.03	0.27	-0.21	0.19	Voltage-dependent calcium channel subunit alpha-2/delta-1	E9Q1X8
Cacna2d2	-0.0034	0.11	0.8	-1.11	0.52	-5.07	0.087	-1.00	0.63	Voltage-dependent calcium channel subunit alpha-2/delta-2	E9Q683
Cacna2d3	-0.1189	0.94	0.21	-0.62	0.5	0.39	0.24	0.32	0.54	Voltage-dependent calcium channel subunit alpha-2/delta-3	Q9Z1L5
Cacnb3	-1.6155	5.40	0.0280	-0.32	0.25	5.32	0.0280	5.08	0.15	Calcium channel voltage-dependent subunit beta 3	A0A2U3TZ49
Cacnb4	-0.0253	0.27	0.94	-1.45	0.0039	-0.52	0.26	-1.18	0.0010	Voltage-dependent L-type calcium channel subunit beta-4	Q8R0S4
Cacng2	0.0000	0.03	0.62	0.03	0.86	0.32	0.0260	0.06	0.53	Voltage-dependent calcium channel gamma-2 subunit	O88602
Cacng3	0.0050	-0.19	0.23	-0.11	0.42	0.17	0.22	-0.31	0.0070	Voltage-dependent calcium channel gamma-3 subunit	Q9JJV5
Cacng8	0.0001	-0.15	0.96	-0.15	0.73	0.00	0.96	-0.29	0.69	Voltage-dependent calcium channel gamma-8 subunit	F7CZ64
Cacybp	-0.0513	0.36	0.2	-0.31	0.22	0.15	0.6	0.06	0.93	Calcyclin-binding protein	Q9CXW3
Cad	-0.0016	0.59	0.52	-0.08	0.76	-0.49	0.72	0.51	1	CAD protein	B2RQC6
Cadm2	0.0040	-0.48	0.0007	-0.04	0.86	-0.09	0.53	-0.51	0.0020	Cell adhesion molecule 2 (Fragment)	G3UZM4
Cadm3	-0.0389	-0.38	0.59	0.72	0.24	0.87	0.51	0.34	0.65	Cell adhesion molecule 3	K4DI58
Cadm4	-0.0050	0.04	0.83	-1.49	0.09	0.55	0.51	-1.45	0.21	Cell adhesion molecule 4	Q8R464
Cadps	-0.0115	0.37	0.28	-0.12	0.34	0.18	0.65	0.25	0.73	Calcium-dependent secretion activator 1	K4DI76
Cadps2	-0.0191	0.45	0.088	-0.15	0.54	0.20	0.37	0.30	0.18	Calcium-dependent secretion activator 2	E9Q5C0
Calcoco1	-0.0047	0.85	0.0130	-0.03	0.8	0.77	0.0100	0.82	0.0280	Calcium-binding and coiled-coil domain-containing protein 1 (Fragment)	A0A2R8VHU8
Cald1	-0.2261	0.19	0.19	-0.55	0.0010	0.17	0.41	-0.36	0.0390	Caldesmon 1	Q8VCQ8
Calm2	0.0083	-0.29	0.47	-0.73	0.76	-0.43	0.97	-1.02	0.56	Calmodulin-2	P0DP27
Calr	-0.0264	-0.19	0.13	0.17	0.12	-0.22	0.0170	-0.02	0.98	Calreticulin	P14211

Table S2: Differentially expressed proteins in cerebral cortex *(continued)*

gene	$\pi_{TW/EW} \times \pi_{TF/TW}$	log2FC TW/EW	padj TW/EW	log2FC TF/TW	padj TF/TW	log2FC EF/EW	padj EF/EW	log2FC TF/EW	padj TF/EW	description	accession
Camk1d	0.0085	-0.49	0.44	-0.26	0.65	-0.70	0.0220	-0.74	0.06	Calcium/calmodulin-dependent protein kinase type 1D	Q8BW96
Camk2a	-11.2102	1.31	0.17	-2.78	0.0001	-0.66	0.0040	-1.47	0.0001	Calcium/calmodulin-dependent protein kinase type II subunit alpha	P11798
Camk2b	-23.9830	2.72	0.21	-4.82	0.0020	-1.63	0.051	-2.10	0.0010	Calcium/calmodulin-dependent protein kinase type II subunit beta	P28652
Camk2d	-1.5364	0.85	0.5	-2.38	0.0030	-0.86	0.0140	-1.54	0.0010	Calcium/calmodulin-dependent protein kinase	E9Q1W0
Camk4	-0.0140	0.08	0.44	-0.30	0.0230	0.12	0.43	-0.23	0.11	Calcium/calmodulin-dependent protein kinase type IV	P08414
Camkk1	-0.0035	0.09	0.94	-1.68	0.14	-1.11	0.25	-1.59	0.0430	Calcium/calmodulin-dependent protein kinase kinase 1 (Fragment)	B1AUD3
Camkk2	-0.0150	0.09	0.15	-0.18	0.075	-0.02	0.55	-0.09	0.48	Calcium/calmodulin-dependent protein kinase kinase 2 (Fragment)	A0A0G2JET2
Camkv	0.0001	-0.05	0.98	-0.42	0.47	-0.36	0.53	-0.47	0.34	CaM kinase-like vesicle-associated protein	A0A0A6YW88
Camsap2	-0.1825	0.74	0.17	-0.63	0.31	-0.18	0.7	0.11	0.53	Calmodulin-regulated spectrin-associated protein 2	A0A0A6YY67
Camsap3	-1.0946	1.27	0.0440	-0.69	0.12	0.69	0.14	0.58	0.4	Calmodulin-regulated spectrin-associated protein 3	Q80VC9
Cand1	-0.0005	-0.27	0.0110	0.21	0.99	0.10	0.35	-0.06	0.46	Cullin-associated NEDD8-dissociated protein 1	Q6ZQ38
Cand2	-0.2046	0.28	0.24	-0.90	0.0490	-0.43	0.087	-0.62	0.07	Cullin-associated NEDD8-dissociated protein 2	Q6ZQ73
Canx	-0.0026	-0.25	0.19	0.09	0.69	-0.16	0.61	-0.15	0.22	Calnexin	P35564
Cap1	0.0000	-0.08	0.94	0.13	0.71	0.30	0.0190	0.04	0.77	Adenylyl cyclase-associated protein 1	P40124
Cap2	0.0017	-0.20	0.59	-0.09	0.39	0.10	0.81	-0.29	0.14	Adenylyl cyclase-associated protein 2	Q9CYT6
Capn5	-0.0027	-0.09	0.86	0.39	0.069	0.47	0.0260	0.31	0.17	Calpain-5	O08688
Caprin1	0.0358	0.21	0.3	0.34	0.11	0.28	0.0280	0.55	0.0090	Caprin-1	Q60865
Capza2	0.0005	-0.24	0.91	-0.19	0.54	0.62	0.0240	-0.44	0.45	F-actin-capping protein subunit alpha-2	P47754
Capzb	-0.0001	0.17	0.92	-0.32	0.86	0.59	0.0390	-0.15	0.78	F-actin-capping protein subunit beta	P47757
Carmil2	-0.0089	0.24	0.52	-0.28	0.34	-2.43	0.0490	-0.04	0.76	Capping protein, Arp2/3 and myosin-I linker protein 2	Q3V3V9
Cars1	-0.0413	0.23	0.21	-0.31	0.14	0.10	0.29	-0.09	0.63	Cysteine–tRNA ligase, cytoplasmic	Q9ER72
Cask	-0.0006	0.10	0.83	-0.30	0.57	0.22	0.35	-0.19	0.7	Peripheral plasma membrane protein CASK (Fragment)	A0A067XG53
Caskin1	-4.6711	0.85	0.0160	-1.02	0.0010	0.11	0.94	-0.17	0.28	Caskin-1	Q6P9K8
Cat	-0.6058	-0.53	0.0040	0.31	0.0290	0.23	0.2	-0.22	0.069	Catalase	P24270
Cbr1	-0.0290	0.19	0.23	-0.23	0.091	-0.32	0.13	-0.05	0.72	Carbonyl reductase [NADPH] 1	P48758
Cbr2	0.0020	-0.15	0.3	-0.12	0.61	0.48	0.051	-0.27	0.23	Carbonyl reductase [NADPH] 2	P08074

Table S2: Differentially expressed proteins in cerebral cortex *(continued)*

gene	$\pi_{TW/EW} \times \pi_{TF/TW}$	log2FC TW/EW	padj TW/EW	log2FC TF/TW	padj TF/TW	log2FC EF/EW	padj EF/EW	log2FC TF/EW	padj TF/EW	description	accession
Cbr3	-0.0034	-0.18	0.84	0.31	0.16	-0.24	0.52	0.13	0.33	Carbonyl reductase [NADPH] 3	Q8K354
Cbr4	-0.0043	0.29	0.66	-0.38	0.61	-0.03	0.99	-0.09	0.98	Carbonyl reductase family member 4	Q91VT4
Cc2d1a	-1.1515	0.69	0.097	-1.05	0.0270	0.74	0.11	-0.36	0.47	Coiled-coil and C2 domain-containing protein 1A	Q8K1A6
Ccar1	-0.0604	-0.15	0.72	1.01	0.0016	-0.50	0.57	0.87	0.0024	Cell division cycle and apoptosis regulator protein 1	Q8CH18
Ccar2	0.0000	-0.04	0.95	0.08	0.82	0.11	0.79	0.04	0.78	Cell cycle and apoptosis regulator protein 2	Q8VDP4
Ccdc124	-0.0128	0.38	0.22	-0.17	0.5	0.13	0.63	0.22	0.44	Coiled-coil domain-containing protein 124	Q9D8X2
Ccdc127	0.0065	0.10	0.57	0.22	0.061	0.27	0.0280	0.32	0.0050	Coiled-coil domain-containing protein 127 (Fragment)	E0CYX9
Ccdc158	-0.2890	-0.77	0.0490	0.36	0.16	0.58	0.13	-0.41	0.11	Coiled-coil domain-containing protein 158	Q8CDI6
Ccdc177	-1.9484	1.29	0.18	-1.65	0.059	-0.11	0.74	-0.37	0.4	Coiled-coil domain-containing protein 177	Q3UHB8
Ccdc22	-0.7900	1.35	0.0015	-0.55	0.42	0.02	0.84	0.80	0.58	Coiled-coil domain-containing protein 22	Q9JIG7
Ccdc6	-1.4142	1.50	0.09	-1.25	0.19	0.24	0.93	0.26	0.96	Coiled-coil domain-containing protein 6	D3YZP9
Ccdc88a	-0.2416	3.18	0.71	-3.05	0.68	-1.23	0.56	0.13	0.97	Girdin	A0A6I8MX08
Ccdc92	-0.0384	0.40	0.26	-0.36	0.35	-1.07	0.27	0.04	0.88	Coiled-coil domain-containing protein 92	Q8VDN4
Ccdc93	-0.0671	0.80	0.0110	-0.17	0.56	-0.12	0.64	0.63	0.0050	Coiled-coil domain-containing protein 93	E9QAD4
Cck	0.0013	-0.10	0.77	-0.20	0.27	-0.01	0.82	-0.30	0.21	Cholecystokinin	P09240
Ccm2	-0.4826	0.92	0.0280	-0.41	0.15	0.04	0.82	0.51	0.17	Cerebral cavernous malformations protein 2 homolog	F7AVU1
Ccny	-0.0002	-0.26	0.77	0.07	0.76	0.27	0.79	-0.19	0.85	Cyclin-Y	Q8BGU5
Cct2	-0.0403	-0.30	0.45	0.47	0.15	0.01	0.95	0.17	0.54	T-complex protein 1 subunit beta	P80314
Cct3	-0.0002	-0.02	0.95	0.25	0.0083	0.00	0.23	0.23	0.0180	T-complex protein 1 subunit gamma	P80318
Cct4	-0.2715	-0.15	0.061	0.38	0.0001	-0.11	0.091	0.23	0.0030	T-complex protein 1 subunit delta	P80315
Cct5	-0.0130	-0.39	0.21	0.33	0.71	-0.12	0.48	-0.07	0.38	T-complex protein 1 subunit epsilon	P80316
Cct6a	0.0003	0.01	0.22	0.20	0.57	0.05	0.58	0.20	0.5	T-complex protein 1 subunit zeta	P80317
Cct7	-0.0009	-0.13	0.65	0.36	0.79	0.17	1	0.23	0.56	T-complex protein 1 subunit eta	P80313
Cct8	-0.1625	-0.25	0.27	0.42	0.0019	0.12	0.51	0.18	0.17	T-complex protein 1 subunit theta	P42932
Ccz1	0.0000	0.18	1	-3.56	1	1.23	0.51	-3.38	0.71	Vacuolar fusion protein CCZ1 homolog	Q8C1Y8
Cd200	-0.0086	-0.17	0.23	0.16	0.32	-0.07	0.37	-0.01	0.92	OX-2 membrane glycoprotein	E9PZI9
Cd47	0.0000	-0.28	1	0.47	0.47	0.30	0.22	0.18	0.39	Integrin-associated protein	A0A2R8VK70
Cdc23	-0.0004	-0.49	0.5	0.09	0.94	0.40	0.57	-0.39	0.38	Cell division cycle protein 23 homolog	A0A0R4J1W7
Cdc42	0.0040	-0.17	0.24	-0.09	0.38	-0.01	0.7	-0.26	0.0160	Cell division control protein 42 homolog	P60766
Cdc42bpb	-0.4174	0.74	0.16	-0.83	0.14	0.03	0.9	-0.09	0.84	Serine/threonine-protein kinase MRCK beta	Q7TT50
Cdc42ep4	-0.2346	0.27	0.054	-0.35	0.0110	-0.08	0.71	-0.08	0.41	Cdc42 effector protein 4	Q9JM96
Cdc5l	-18.0636	4.25	0.061	-3.80	0.12	-0.10	0.71	0.45	0.33	Cell division cycle 5-like protein	Q6A068

Table S2: Differentially expressed proteins in cerebral cortex *(continued)*

gene	$\pi_{TW/EW} \times \pi_{TF/TW}$	log2FC TW/EW	padj TW/EW	log2FC TF/TW	padj TF/TW	log2FC EF/EW	padj EF/EW	log2FC TF/EW	padj TF/EW	description	accession
Cdh10	-0.0013	-0.26	0.4	0.05	0.57	-0.13	0.88	-0.21	0.69	Cadherin-10	P70408
Cdh11	-0.0086	0.24	0.48	-0.29	0.41	0.25	0.14	-0.04	0.8	Cadherin-11	P55288
Cdh13	0.0000	-0.06	0.94	-0.06	0.51	0.19	0.78	-0.11	0.63	Cadherin-13	Q9WTR5
Cdh2	-9.3455	2.27	0.0410	-2.40	0.058	0.65	0.4	-0.13	0.9	Cadherin-2	D3YYT0
Cdh6	-63.9443	7.95	0.084	-7.80	0.11	1.72	0.3	0.15	0.63	Cadherin-6	P97326
Cdk5	-0.0010	0.21	0.34	-0.03	0.47	-0.28	0.63	0.18	0.88	Cyclin-dependent-like kinase 5	P49615
Cdk5r1	-0.3870	0.55	0.13	-0.65	0.06	-0.41	0.2	-0.10	0.6	Cyclin-dependent kinase 5 activator 1	P61809
Cdk5rap3	-0.0064	-0.19	0.79	0.97	0.46	-0.40	0.7	0.77	0.48	CDK5 regulatory subunit-associated protein 3	Q99LM2
Cdkl5	-0.9441	0.75	0.11	-1.10	0.064	-0.77	0.13	-0.35	0.58	Cyclin-dependent kinase-like 5	A0A0G2JF79
Cdkn2aip	0.0005	0.49	0.87	0.11	0.72	1.73	0.36	0.60	0.72	CDKN2A-interacting protein	Q8BI72
Cdv3	-0.0310	0.53	0.37	-0.39	0.45	0.21	0.65	0.14	0.76	Protein CDV3	A0A087WNP6
Celf2	-0.0011	-0.28	0.96	0.39	0.28	-0.23	0.83	0.11	0.66	CUGBP Elav-like family member 2	Q9Z0H4
Celsr2	0.0000	0.00	0.51	-0.16	0.22	0.35	0.25	-0.16	0.48	Cadherin EGF LAG seven-pass G-type receptor 2	Q9R0M0
Cend1	0.0001	0.10	0.82	0.08	0.65	0.38	0.47	0.17	0.31	Cell cycle exit and neuronal differentiation protein 1	Q9JKC6
Cenpv	-7.9217	1.42	0.0410	-1.49	0.0020	-0.32	0.62	-0.07	0.94	Centromere protein V	Q9CXS4
Cep162	-0.1327	0.61	0.0280	-0.17	0.15	-0.09	0.61	0.44	0.0280	Centrosomal protein of 162 kDa	Q6ZQ06
Cep170	-0.0338	0.72	0.55	-0.79	0.59	-0.30	0.66	-0.07	0.97	Centrosomal protein of 170 kDa	H7BX26
Cep170b	-0.1046	0.75	0.48	-1.10	0.4	-0.33	0.75	-0.35	0.8	AW555464 protein	B9EIX2
Cfh	-0.0088	-0.57	0.49	0.24	0.62	0.51	0.35	-0.33	0.55	Complement factor H	D6RGQ0
Cfl2	-0.0002	-0.15	0.65	0.06	0.78	0.28	0.5	-0.09	0.99	Cofilin-2	P45591
Chchd3	0.0529	0.50	0.0009	0.08	0.37	0.25	0.46	0.59	0.0130	MICOS complex subunit Mic19	Q9CRB9
Chchd6	-0.0099	0.41	0.31	-0.13	0.43	0.17	0.25	0.28	0.84	MICOS complex subunit Mic25	Q91VN4
Cherp	0.0015	-0.27	0.34	-0.05	0.58	-0.19	0.43	-0.32	0.088	Calcium homeostasis endoplasmic reticulum protein	A0A1D5RL92
Chid1	-0.1270	-0.14	0.22	0.46	0.0010	0.07	0.52	0.32	0.0130	Chitinase domain-containing protein 1	A0A0R4J242
Chl1	-0.3924	-0.77	0.11	0.60	0.13	0.21	0.62	-0.17	0.57	Neural cell adhesion molecule L1-like protein	P70232
Chmp1a	-0.5392	0.52	0.31	-1.20	0.0200	-0.30	0.15	-0.68	0.0008	Charged multivesicular body protein 1a	Q921W0
Chmp1b2	0.0156	-0.78	0.15	-0.32	0.84	0.63	0.093	-1.09	0.25	Charged multivesicular body protein 1b-2	Q9CQD4
Chmp2a	-0.2139	0.43	0.35	-0.97	0.075	-0.28	0.66	-0.55	0.32	Charged multivesicular body protein 2a	Q9DB34
Chmp2b	-0.5020	0.54	0.35	-1.20	0.0200	-0.28	0.18	-0.67	0.065	Charged multivesicular body protein 2b	Q8BJF9
Chmp4b	-0.0100	0.52	0.22	-0.32	0.81	0.11	0.44	0.20	0.16	Charged multivesicular body protein 4b	Q9D8B3
Chmp6	0.0000	-0.03	0.65	-0.15	0.88	-0.47	0.36	-0.18	0.78	Charged multivesicular body protein 6	P0C0A3
Cirbp	-0.0950	0.40	0.13	-0.36	0.18	-0.01	0.83	0.04	0.92	Cold-inducible RNA-binding protein	P60824
Cisd1	-0.0816	0.47	0.23	-0.33	0.15	0.60	0.26	0.14	0.98	CDGSH iron-sulfur domain-containing protein 1	Q91WS0

Table S2: Differentially expressed proteins in cerebral cortex *(continued)*

gene	$\pi_{TW/EW}\times$ $\pi_{TF/TW}$	log2FC TW/EW	padj TW/EW	log2FC TF/TW	padj TF/TW	log2FC EF/EW	padj EF/EW	log2FC TF/EW	padj TF/EW	description	accession
Cisd3	-0.5255	0.60	0.39	-0.92	0.0047	0.05	0.82	-0.33	0.23	CDGSH iron-sulfur domain-containing protein 3, mitochondrial	B1AR13
Cit	-31.9783	1.88	0.0460	-3.18	0.0001	-0.82	0.19	-1.30	0.0460	Citron Rho-interacting kinase	E9QL53
Ckap4	0.0365	0.49	0.0160	0.17	0.57	0.43	0.0320	0.66	0.0040	Cytoskeleton-associated protein 4	Q8BMK4
Ckap5	-0.0008	0.26	0.082	-0.01	0.53	0.16	0.67	0.25	0.25	Cytoskeleton-associated protein 5	A0A0R4J0K2
Ckb	0.0203	-0.36	0.72	-0.26	0.0300	-0.25	0.16	-0.62	0.0001	Creatine kinase B-type	Q04447
Ckmt1	0.0000	-0.35	0.055	0.16	1	0.24	0.0001	-0.19	0.0480	Creatine kinase U-type, mitochondrial	P30275
Clasp2	-6.5565	1.08	0.0002	-0.88	0.0140	0.43	0.0270	0.20	0.13	CLIP-associating protein 2	E9Q8N5
Clcn2	0.7692	1.38	0.12	0.56	0.083	-1.07	0.78	1.95	0.0100	Chloride channel protein	A0A338P673
Clcn3	0.0000	0.02	0.96	-0.09	0.59	-0.61	0.15	-0.07	0.69	Chloride channel protein	Q790S0
Clcn6	-0.0015	-0.08	0.81	0.29	0.2	0.71	0.0280	0.21	0.33	Chloride channel protein	A2A7F6
Cldn11	-0.8703	0.37	0.42	-2.81	0.0060	-0.09	0.84	-2.45	0.17	Claudin-11	Q60771
Clec4f	-0.0065	-0.19	0.69	0.49	0.37	-0.44	0.93	0.30	0.38	C-type lectin domain family 4 member F	A0A0R4IZZ5
Clint1	0.1711	0.52	0.19	0.38	0.063	0.45	0.51	0.90	0.0021	Clathrin interactor 1	Q5SUH7
Clip1	-0.0001	-0.19	0.46	0.04	0.88	0.42	0.081	-0.15	0.57	CAP-Gly domain-containing linker protein 1	D3Z2Z1
Clip2	-0.3697	0.66	0.0008	-0.19	0.11	0.07	0.93	0.47	0.23	CAP-Gly domain-containing linker protein 2	Q9Z0H8
Clip3	0.0066	0.71	0.88	0.31	0.29	-0.47	0.84	1.03	0.44	CAP-Gly domain-containing linker protein 3	B9EHT4
Clmn	-2.6798	3.41	0.52	-4.73	0.26	-1.25	0.58	-1.33	0.52	Calmin	Q8C5W0
Clpb	-0.0013	0.04	0.92	-1.45	0.23	-0.19	0.81	-1.40	0.15	Caseinolytic peptidase B protein homolog	E9PY58
Clpp	0.0042	-0.16	0.33	-0.17	0.48	-0.46	0.1	-0.33	0.0300	ATP-dependent Clp protease proteolytic subunit, mitochondrial	O88696
Clpx	-0.0893	2.92	0.0260	-0.17	0.77	0.08	0.86	2.75	0.083	ATP-dependent Clp protease ATP-binding subunit clpX-like, mitochondrial	Q6P8N8
Clstn1	-0.0075	-0.06	0.52	0.32	0.0420	-0.14	0.28	0.26	0.056	Calsyntenin-1	Q9EPL2
Clta	-0.0158	-0.05	0.81	1.15	0.0010	-0.01	0.95	1.10	0.0010	Clathrin light chain	B1AWD8
Cltb	-0.0345	-0.06	0.75	1.31	0.0003	0.18	0.11	1.25	0.0008	Clathrin light chain B	Q6IRU5
Cltc	0.4466	0.10	0.13	1.26	0.0001	0.08	1	1.36	0.0001	Clathrin heavy chain 1	Q68FD5
Clu	-0.3310	-0.45	0.0030	0.24	0.061	0.20	0.28	-0.21	0.1	Clusterin	Q06890
Clybl	-0.0847	0.10	0.052	-0.33	0.0100	0.06	0.37	-0.23	0.64	Citramalyl-CoA lyase, mitochondrial	Q8R4N0
Cmpk1	0.0021	-0.44	0.76	-0.29	0.73	-0.55	0.57	-0.73	0.57	UMP-CMP kinase	A0A0R4J093
Cmpk2	0.0174	-0.20	0.6	-0.46	0.14	-1.04	0.34	-0.66	0.16	UMP-CMP kinase 2, mitochondrial	Q3U5Q7
Cndp2	0.0038	-0.30	0.32	-0.06	0.37	-0.41	0.4	-0.35	0.0130	Cytosolic non-specific dipeptidase	Q9D1A2
Cnksr2	-0.4588	0.69	0.87	-3.97	0.0017	-0.47	0.77	-3.28	0.0001	Connector enhancer of kinase suppressor of ras 2	Q80YA9
Cnn3	-5.0098	1.15	0.11	-1.67	0.0019	0.33	0.62	-0.52	0.67	Calponin	A0A0G2JDV8
Cnnm1	-0.0003	-0.05	0.92	0.39	0.4	0.49	0.0280	0.34	0.4	Metal transporter CNNM1	Q0GA42

Table S2: Differentially expressed proteins in cerebral cortex *(continued)*

gene	$\pi_{TW/EW} \times \pi_{TF/TW}$	log2FC TW/EW	padj TW/EW	log2FC TF/TW	padj TF/TW	log2FC EF/EW	padj EF/EW	log2FC TF/EW	padj TF/EW	description	accession
Cnnm3	-0.4927	-3.91	0.17	0.56	0.51	1.71	0.052	-3.35	0.85	Metal transporter CNNM3	Q32NY4
Cnot1	0.0000	0.02	0.81	-0.01	0.96	-0.29	0.58	0.01	0.82	CCR4-NOT transcription complex subunit 1	A0A1D5RMJ8
Cnot3	0.0011	-0.04	0.9	-1.25	0.34	-0.28	0.57	-1.28	0.27	CCR4-NOT transcription complex subunit 3	Q8K0V4
Cnot9	-0.0002	0.50	0.0230	-0.01	0.95	-0.12	0.86	0.49	0.073	CCR4-NOT transcription complex subunit 9	Q9JKY0
Cnp	0.0159	-0.09	0.85	-0.93	0.0020	-0.23	0.52	-1.02	0.0039	2',3'-cyclic-nucleotide 3'-phosphodiesterase	P16330
Cnrip1	0.0477	-0.17	0.57	-0.38	0.0009	-0.16	0.38	-0.54	0.0001	CB1 cannabinoid receptor-interacting protein 1	Q5M8N0
Cntn1	0.0148	0.26	0.0040	0.19	0.75	0.37	0.16	0.45	0.0150	Contactin-1	P12960
Cntn2	-0.0056	0.09	0.56	-0.27	0.12	0.03	0.92	-0.18	0.091	Contactin-2	Q61330
Cntn4	0.0023	-0.64	0.0013	-0.04	0.93	0.26	0.15	-0.68	0.0004	Contactin-4	Q69Z26
Cntnap1	-0.0533	0.12	0.42	-0.59	0.0100	0.18	0.59	-0.46	0.11	Contactin-associated protein 1	O54991
Cntnap2	0.0000	-0.24	0.73	0.25	0.99	-0.06	0.52	0.01	0.8	Contactin-associated protein-like 2	E9QNF7
Coa3	-0.0036	0.25	0.2	-0.10	0.62	0.48	0.35	0.15	0.37	Cytochrome c oxidase assembly factor 3 homolog, mitochondrial	Q9D2R6
Coasy	0.0036	0.51	0.36	0.08	0.63	1.82	0.0130	0.58	0.12	Bifunctional coenzyme A synthase	Q9DBL7
Cobl	-0.0035	-0.37	0.38	0.19	0.76	-0.16	0.67	-0.18	0.44	Protein cordon-bleu	G3UWY3
Commd1	0.0004	0.03	0.97	0.92	0.061	-0.14	0.64	0.95	0.0280	COMM domain-containing protein 1 (Fragment)	G8JL54
Commd2	-0.0026	-0.03	0.91	0.89	0.0040	-0.05	0.82	0.85	0.0010	COMM domain-containing protein 2	Q8BXC6
Commd3	0.0080	0.04	0.83	0.98	0.0030	0.29	0.45	1.02	0.0030	COMM domain-containing protein 3	Q63829
Commd9	0.2037	0.15	0.36	1.02	0.0010	0.26	0.16	1.17	0.0010	COMM domain-containing protein 9	Q8K2Q0
Comtd1	-2.5440	2.30	0.14	-2.09	0.24	1.49	0.46	0.21	0.82	Catechol O-methyltransferase domain-containing protein 1	Q8BIG7
Copa	-0.0173	1.01	0.24	-0.32	0.82	0.26	0.42	0.70	0.22	Coatomer subunit alpha	F8WHL2
Copb2	0.1361	0.18	0.27	0.65	0.0090	-0.10	0.45	0.83	0.0002	Coatomer subunit beta'	O55029
Cope	-0.2288	-0.25	0.25	0.82	0.0140	0.43	0.0280	0.56	0.0420	Coatomer subunit epsilon	O89079
Copg2	0.0000	0.10	0.93	0.23	0.99	0.17	0.77	0.33	1	Coatomer subunit gamma-2	Q9QXK3
Cops2	-0.0006	0.20	0.37	-0.06	0.76	0.33	0.18	0.14	0.51	COP9 signalosome complex subunit 2	P61202
Cops4	0.0011	0.04	0.49	0.13	0.22	0.24	0.0440	0.17	0.12	COP9 signalosome complex subunit 4	O88544
Cops6	0.0206	0.73	0.14	0.11	0.5	0.90	0.0380	0.84	0.067	COP9 signalosome complex subunit 6	D3Z0F5
Cops7a	-0.0101	0.35	0.34	-0.21	0.51	-0.19	0.56	0.14	0.78	COP9 signalosome complex subunit 7a	Q9CZ04
Cops8	0.0071	0.22	0.56	0.28	0.35	0.47	0.15	0.50	0.11	COP9 signalosome complex subunit 8	Q8VBV7
Coq6	-0.0216	0.44	0.79	-1.24	0.41	-0.25	0.85	-0.79	0.52	Ubiquinone biosynthesis monooxygenase COQ6, mitochondrial	Q8R1S0
Coro1a	0.0102	-0.15	0.84	-0.30	0.0010	-0.10	0.056	-0.45	0.0001	Coronin-1A	O89053

Table S2: Differentially expressed proteins in cerebral cortex *(continued)*

gene	$\pi_{TW/EW} \times \pi_{TF/TW}$	log2FC TW/EW	padj TW/EW	log2FC TF/TW	padj TF/TW	log2FC EF/EW	padj EF/EW	log2FC TF/EW	padj TF/EW	description	accession
Coro1b	0.8370	-0.31	0.0010	-0.30	0.0010	0.28	0.0010	-0.61	0.0001	Coronin-1B	Q9WUM3
Coro1c	-0.0015	-0.16	0.42	0.07	0.44	0.34	0.0010	-0.09	0.79	Coronin-1C	Q9WUM4
Coro2a	0.3454	-1.16	0.66	-4.38	0.42	1.55	0.38	-5.54	0.18	Coronin	B1AVH5
Coro2b	-3.7832	0.93	0.2	-1.94	0.0010	0.13	0.62	-1.01	0.0280	Coronin-2B	Q8BH44
Coro6	0.0015	-0.44	0.0260	-0.02	0.78	0.24	0.11	-0.46	0.0009	Coronin-6	Q920M5
Coro7	0.0003	-0.08	0.89	-0.19	0.46	-0.39	0.5	-0.27	1	Coronin-7	Q9D2V7
Cotl1	0.0025	-0.01	0.84	-0.98	0.0004	-0.29	0.49	-0.99	0.08	Coactosin-like protein	Q9CQI6
Cox4i1	-0.1988	0.35	0.11	-0.44	0.0450	0.27	0.73	-0.09	0.46	Cytochrome c oxidase subunit 4 isoform 1, mitochondrial	P19783
Cox5a	-0.1194	0.17	0.18	-0.50	0.0130	0.36	0.51	-0.33	0.13	Cytochrome c oxidase subunit 5A, mitochondrial	P12787
Cox5b	-0.2283	0.31	0.085	-0.41	0.0210	0.32	0.1	-0.10	0.69	Cytochrome c oxidase subunit 5B, mitochondrial	P19536
Cox6b1	-0.1605	0.36	0.29	-0.62	0.0460	0.12	0.49	-0.26	0.2	Cytochrome c oxidase subunit 6B1	P56391
Cox6c	-0.0075	0.19	0.64	-0.38	0.29	0.42	0.62	-0.19	0.35	Cytochrome c oxidase subunit 6C	Q9CPQ1
Cox7a2	-0.0096	0.30	0.41	-0.26	0.48	0.55	0.1	0.04	0.87	Cytochrome c oxidase subunit 7A2, mitochondrial	P48771
Cox7a2l	-0.0234	-0.54	0.85	0.85	0.19	-1.00	0.58	0.31	0.4	Cytochrome c oxidase subunit 7A-related protein, mitochondrial	Q61387
Cox7c	-0.2151	0.58	0.073	-0.41	0.16	0.22	0.61	0.17	0.49	Cytochrome c oxidase subunit 7C, mitochondrial	P17665
Cpe	-0.6180	1.47	0.094	-0.68	0.25	0.32	0.69	0.79	0.62	Carboxypeptidase E	Q00493
Cpeb3	-0.2099	0.98	0.38	-1.03	0.32	-0.89	0.41	-0.05	0.85	Cytoplasmic polyadenylation element-binding protein 3	A0A0R4J102
Cplx1	-0.0095	-0.10	0.44	0.29	0.12	-0.16	0.24	0.19	0.2	Complexin-1	P63040
Cpne1	-0.0004	-0.50	0.059	0.04	0.96	0.18	0.39	-0.46	0.17	Copine-1	Q8C166
Cpne6	0.0036	-0.84	0.49	-0.27	0.89	0.01	0.38	-1.10	0.41	Copine-6	Q9Z140
Cpsf7	-0.0007	-0.16	0.84	0.31	0.66	-0.19	0.8	0.15	0.76	Cleavage and polyadenylation specificity factor subunit 7	Q8BTV2
Cracd	-0.0199	0.31	0.73	-0.55	0.14	-0.48	0.56	-0.25	0.71	RIKEN cDNA C530008M17 gene	E9Q5L4
Cracdl	-0.1841	0.52	0.11	-0.28	0.0480	0.26	0.97	0.25	1	Capping protein-inhibiting regulator of actin-like	E9Q3M9
Crat	0.0035	0.28	0.0320	0.03	0.53	0.38	0.57	0.30	0.17	Carnitine O-acetyltransferase	P47934
Creld1	-0.0004	-0.05	0.85	0.27	0.35	-0.10	0.34	0.22	0.41	Protein disulfide isomerase Creld1	Q91XD7
Crip2	-0.9990	1.16	0.32	-1.89	0.12	-0.19	0.96	-0.73	0.45	Cysteine-rich protein 2	Q9DCT8
Crk	-0.0310	1.17	0.53	-0.99	0.8	1.85	0.0280	0.18	0.77	Adapter molecule crk	Q64010
Crmp1	-0.0048	-0.12	0.42	0.18	0.26	0.09	0.46	0.07	0.62	Crmp1 protein	Q6P1J1
Crocc	-0.0053	0.10	0.91	-1.51	0.14	0.61	0.52	-1.41	0.12	Rootletin	Q8CJ40
Crtac1	0.0000	0.04	0.72	-0.02	0.93	0.29	0.28	0.02	0.69	Cartilage acidic protein 1	A0A571BE99

Table S2: Differentially expressed proteins in cerebral cortex *(continued)*

gene	$\pi_{TW/EW} \times \pi_{TF/TW}$	log2FC TW/EW	padj TW/EW	log2FC TF/TW	padj TF/TW	log2FC EF/EW	padj EF/EW	log2FC TF/EW	padj TF/EW	description	accession
Cryab	0.0000	0.07	0.85	-0.02	0.89	0.21	0.49	0.05	0.81	Alpha-crystallin B chain	P23927
Crym	0.0114	-0.55	0.0001	-0.02	0.55	-0.32	0.0060	-0.57	0.0001	Ketimine reductase mu-crystallin	O54983
Cryz	1.8467	0.67	0.14	0.92	0.0003	-0.50	0.68	1.59	0.0010	Quinone oxidoreductase (Fragment)	A0A0A6YXR4
Cryzl1	0.0006	-0.04	0.57	-0.13	0.34	0.22	0.43	-0.18	0.82	Quinone oxidoreductase-like protein 1	Q921W4
Cs	0.0962	-0.24	0.13	-0.30	0.0310	0.14	0.87	-0.54	0.0017	Citrate synthase, mitochondrial	Q9CZU6
Csde1	0.0000	-0.15	0.64	0.01	1	-0.02	0.81	-0.14	0.37	Cold shock domain-containing protein E1	A0A0G2JF72
Csnk1a1	-0.3246	0.56	0.51	-1.78	0.077	0.02	0.68	-1.22	0.11	Casein kinase I isoform alpha	E9Q2U6
Csnk1e	-0.0285	0.22	0.86	-1.74	0.073	-0.70	0.33	-1.52	0.11	Casein kinase I isoform epsilon	Q9JMK2
Csnk1g3	-20.2042	4.00	0.29	-8.65	0.082	-2.99	0.82	-4.65	0.077	Casein kinase I isoform gamma-3	Q8C4X2
Cspg4	-0.0951	3.52	0.72	-3.13	0.87	0.69	0.86	0.39	0.75	Chondroitin sulfate proteoglycan 4	Q8VHY0
Cspg5	-0.0122	-0.20	0.52	0.41	0.3	-0.17	0.59	0.21	0.66	Chondroitin sulfate proteoglycan 5	Q71M36
Cst3	-0.0185	0.26	0.27	-0.26	0.33	0.23	0.27	0.00	0.74	Cystatin-C	P21460
Cstf2	-0.2516	0.34	0.54	-2.25	0.059	-0.71	0.57	-1.92	0.21	Cleavage stimulation factor subunit 2	Q8BIQ5
Ctbp1	-0.0016	-0.05	0.53	0.27	0.38	-0.03	0.37	0.22	0.087	C-terminal-binding protein 1	O88712
Ctnna2	-0.2391	0.44	0.17	-0.65	0.082	0.34	0.5	-0.20	0.95	Alpha N-catenin	E0CXB9
Ctnnb1	-0.2644	0.38	0.12	-0.64	0.066	0.21	0.69	-0.26	0.96	Catenin beta-1	Q02248
Ctnnd2	-0.0004	0.45	0.82	-0.63	0.96	0.18	0.63	-0.18	0.76	Catenin delta-2	B7ZNF6
Ctsd	-0.0090	-0.33	0.0270	0.10	0.67	-0.30	0.065	-0.23	0.071	Cathepsin D	F8WIR1
Cttn	0.0009	-0.16	0.95	-0.16	0.0260	0.22	0.0005	-0.32	0.0430	Src substrate cortactin	Q60598
Cttnbp2	-0.0004	0.07	0.94	-0.38	0.27	0.22	0.51	-0.31	0.27	Cortactin-binding protein 2	B9EJA2
Cul1	-0.0186	0.33	0.13	-0.16	0.4	-0.03	0.76	0.16	0.32	Cullin-1 (Fragment)	Q3TPM3
Cul3	0.0001	-0.02	0.99	-1.46	0.16	-0.96	0.26	-1.49	0.14	Cullin-3	Q9JLV5
Cul5	-0.0001	0.03	0.85	-0.23	0.77	0.19	0.89	-0.20	0.91	Cullin-5	E9PV12
Cyb5a	-0.2363	-1.38	0.34	1.25	0.51	-1.22	0.52	-0.13	0.78	Cytochrome b5	G5E850
Cyb5b	-0.0004	0.06	0.84	-0.53	0.68	0.38	0.66	-0.48	0.54	Cytochrome b5 type B	Q9CQX2
Cyb5r3	-1.0101	0.42	0.061	-0.66	0.0010	-0.02	0.74	-0.25	0.62	NADH-cytochrome b5 reductase	F2Z456
Cyc1	-3.7422	0.58	0.0020	-0.65	0.0002	0.18	0.98	-0.07	0.89	Cytochrome c1, heme protein, mitochondrial	Q9D0M3
Cycs	-0.0003	0.05	0.94	-0.55	0.43	0.51	0.36	-0.50	0.46	Cytochrome c, somatic	P62897
Cyfip2	0.0002	0.13	0.38	0.15	0.94	0.01	0.78	0.27	0.43	Cytoplasmic FMR1-interacting protein 2	Q5SQX6
Cyld	-0.7121	0.58	0.25	-1.30	0.0270	0.47	0.52	-0.72	0.14	Ubiquitin carboxyl-terminal hydrolase CYLD	Q80TQ2
Cyp46a1	0.0000	0.40	0.0010	0.00	0.9	0.19	0.97	0.40	0.0160	Cholesterol 24-hydroxylase	Q9WVK8
Cyrib	0.0000	-0.06	0.92	0.01	0.98	-0.41	0.76	-0.05	0.97	CYFIP-related Rac1 interactor B	Q921M7
Cyth2	-0.0270	0.69	0.4	-0.61	0.69	0.31	0.66	0.08	0.51	Cytohesin-2	A0A1B0GRX7
D430041D05Rik	-0.0210	0.27	0.52	-0.60	0.35	0.15	0.62	-0.33	0.93	RIKEN cDNA D430041D05 gene	A0A2R8VKG2
D630045J12Rik	-0.1228	0.16	0.5	-0.85	0.0010	0.05	0.87	-0.69	0.0001	RIKEN cDNA D630045J12 gene	D3YTS3

Table S2: Differentially expressed proteins in cerebral cortex *(continued)*

gene	$\pi_{TW/EW} \times \pi_{TF/TW}$	log2FC TW/EW	padj TW/EW	log2FC TF/TW	padj TF/TW	log2FC EF/EW	padj EF/EW	log2FC TF/EW	padj TF/EW	description	accession
Daam1	-0.5297	0.49	0.0001	-0.25	0.083	-0.14	0.38	0.24	0.11	Disheveled-associated activator of morphogenesis 1	Q8BPM0
Daam2	-0.8254	1.10	0.0280	-0.67	0.19	0.13	0.53	0.43	0.24	Disheveled-associated activator of morphogenesis 2	Q80U19
Dagla	-0.0002	0.03	0.74	-0.10	0.31	0.28	0.86	-0.07	0.5	Diacylglycerol lipase-alpha	Q6WQJ1
Dap3	-1.0736	0.88	0.1	-1.22	0.1	0.53	0.22	-0.34	0.67	28S ribosomal protein S29, mitochondrial	G3X9M0
Dapk1	-0.0160	0.16	0.81	-1.09	0.1	0.71	0.19	-0.93	0.1	Death-associated protein kinase 1	Q80YE7
Dars1	0.0000	-0.08	0.63	0.07	0.91	0.04	0.6	-0.01	0.58	Aspartate–tRNA ligase, cytoplasmic	Q922B2
Dars2	-1.0995	7.83	0.052	-2.67	0.91	8.64	0.0280	5.16	0.12	Aspartate–tRNA ligase, mitochondrial	Q8BIP0
Dbn1	-4.6948	0.72	0.0130	-1.03	0.0004	1.42	0.0020	-0.31	0.24	Drebrin	A0A0R4J1E3
Dbnl	0.2740	-0.27	0.23	-0.53	0.0010	0.04	0.95	-0.80	0.0002	Drebrin-like protein	Q62418
Dbt	-0.4508	0.68	0.14	-0.81	0.11	-0.11	0.55	-0.13	0.9	Lipoamide acyltransferase component of branched-chain alpha-keto acid dehydrogenase complex, mitochondrial	P53395
Dclk1	-1.7059	0.80	0.0170	-0.65	0.0140	-0.19	0.55	0.15	0.58	Serine/threonine-protein kinase DCLK1	A0A0G2JGQ6
Dclk2	-140.6945	7.85	0.0260	-7.70	0.0340	1.54	0.45	0.15	0.8	Serine/threonine-protein kinase DCLK2	A0A0A6YX71
Dctn1	-0.2190	0.39	0.26	-0.49	0.0110	0.05	0.54	-0.10	0.17	Dynactin subunit 1	E9Q3M3
Dctn2	-0.0033	-0.06	0.1	0.23	0.58	0.27	0.069	0.17	0.0210	Dynactin subunit 2	Q99KJ8
Dctn3	-1.6800	-0.28	0.0010	0.50	0.0001	0.36	0.91	0.22	0.0450	Dynactin subunit 3	Q9Z0Y1
Dctn4	-0.9070	0.92	0.15	-1.08	0.078	0.18	0.55	-0.16	0.65	Dynactin subunit 4	Q8CBY8
Dcun1d1	-2.0393	1.02	0.73	-9.42	0.0280	-3.27	0.55	-8.41	0.077	DCN1-like protein 1	Q9QZ73
Ddah1	0.0081	-0.32	0.51	-0.28	0.49	-0.44	0.15	-0.60	0.0380	N(G),N(G)-dimethylarginine dimethylaminohydrolase 1	Q9CWS0
Ddb1	0.0000	-0.28	1	0.34	0.51	0.16	0.97	0.06	0.58	DNA damage-binding protein 1	Q3U1J4
Ddc	0.0002	-0.12	0.82	-0.09	0.69	-0.03	0.95	-0.21	0.45	Aromatic-L-amino-acid decarboxylase	O88533
Ddost	0.6083	0.21	0.083	0.67	0.0001	0.28	0.25	0.88	0.0001	Dolichyl-diphosphooligosaccharide–protein glycosyltransferase 48 kDa subunit	O54734
Ddx1	-0.3829	0.58	0.0026	-0.31	0.15	0.17	0.25	0.28	0.0300	ATP-dependent RNA helicase DDX1	Q91VR5
Ddx17	-0.0147	0.32	0.29	-0.22	0.41	-0.43	0.096	0.10	0.73	RNA helicase	Q3U741
Ddx19b	-0.0002	0.04	0.92	-0.29	0.34	-0.01	1	-0.25	0.33	RNA helicase	Q8BZY3
Ddx39b	-0.0021	-0.21	0.74	0.32	0.58	-0.24	0.72	0.11	0.98	Spliceosome RNA helicase Ddx39b	Q9Z1N5
Ddx46	0.0019	0.13	0.46	0.13	0.46	0.11	0.59	0.27	0.76	RNA helicase	F8WHR6
Ddx5	-0.0070	0.70	0.88	-0.93	0.64	-0.43	0.53	-0.23	0.64	Probable ATP-dependent RNA helicase DDX5	Q61656
Ddx6	0.0002	0.51	0.18	0.02	0.93	0.32	0.62	0.54	0.23	Probable ATP-dependent RNA helicase DDX6	P54823
Decr1	0.5659	0.36	0.0120	0.40	0.0090	0.18	0.44	0.76	0.0001	2,4-dienoyl-CoA reductase [(3E)-enoyl-CoA-producing], mitochondrial	Q9CQ62

Table S2: Differentially expressed proteins in cerebral cortex *(continued)*

gene	$\pi_{TW/EW} \times \pi_{TF/TW}$	log2FC TW/EW	padj TW/EW	log2FC TF/TW	padj TF/TW	log2FC EF/EW	padj EF/EW	log2FC TF/EW	padj TF/EW	description	accession
Dennd1a	-0.0101	-0.11	0.57	0.28	0.0450	-0.06	0.75	0.16	0.25	DENN domain-containing protein 1A	Q8K382
Denr	0.0008	-0.05	0.46	-0.11	0.36	0.14	0.31	-0.16	0.98	Density-regulated protein (Fragment)	E9Q0G1
Derpc	-0.1117	-0.37	0.0160	0.16	0.089	0.13	0.21	-0.22	0.19	Decreased expression in renal and prostate cancer protein	P0CG14
Dgkb	-2.0151	1.38	0.0240	-0.87	0.092	0.48	0.36	0.51	0.49	Diacylglycerol kinase beta	Q6NS52
Dgkd	-0.0011	0.15	0.69	-0.23	0.63	-0.80	0.57	-0.08	0.76	Diacylglycerol kinase delta	E9PUQ8
Dgke	0.0015	-0.09	0.97	-4.14	0.49	-4.51	0.19	-4.23	0.45	Diacylglycerol kinase (ATP)	F2Z490
Dgki	0.0030	-0.06	0.82	-0.61	0.11	-0.09	0.87	-0.67	0.095	Diacylglycerol kinase iota	D3YWQ0
Dgkz	-0.0623	0.63	0.28	-0.53	0.46	0.35	0.48	0.10	0.37	Diacylglycerol kinase	A2AHJ7
Dhrs1	0.0000	-0.01	0.99	0.06	0.76	-0.06	0.52	0.04	0.75	Dehydrogenase/reductase SDR family member 1	Q99L04
Dhrs4	-0.0031	-0.07	0.62	0.24	0.13	0.28	0.0220	0.17	0.0360	Dehydrogenase/reductase SDR family member 4	Q99LB2
Dhrs7b	-0.1038	0.47	0.2	-0.51	0.24	-0.05	0.85	-0.04	0.91	Dehydrogenase/reductase SDR family member 7B	Z4YKT6
Dhx15	-0.7105	0.88	0.26	-1.38	0.1	0.32	0.7	-0.50	0.49	Pre-mRNA-splicing factor ATP-dependent RNA helicase DHX15	O35286
Dhx30	-4.9320	1.91	0.22	-2.72	0.0360	-0.81	0.41	-0.81	0.34	RNA helicase	A0A0G2JGL8
Dhx36	0.0045	0.41	0.76	0.95	0.8	-2.11	0.88	1.37	0.44	ATP-dependent DNA/RNA helicase DHX36	Q8VHK9
Dhx9	-0.0033	0.16	0.59	-0.43	0.62	-0.55	0.28	-0.27	0.94	DEAH box protein 9	A0A087WPL5
Dip2a	-0.0154	0.38	0.6	-0.59	0.49	-1.00	0.64	-0.21	0.83	Disco-interacting protein 2 homolog A	D3Z7D3
Dip2b	-0.9227	0.63	0.0010	-0.39	0.056	0.13	0.56	0.24	0.27	Disco-interacting protein 2 homolog B	Q3UH60
Diras2	-0.2782	0.61	0.0290	-0.36	0.15	0.27	0.54	0.24	0.48	GTP-binding protein Di-Ras2	Q5PR73
Dis3l2	-0.5469	0.52	0.12	-0.75	0.0300	0.43	0.12	-0.22	0.31	DIS3-like exonuclease 2	Q8CI75
Dlat	0.0000	0.00	0.77	-0.17	0.057	-0.37	0.072	-0.17	0.11	Dihydrolipoyllysine-residue acetyltransferase component of pyruvate dehydrogenase complex, mitochondrial	Q8BMF4
Dld	-0.0094	-0.19	0.0210	0.07	0.38	-0.08	0.097	-0.12	0.079	Dihydrolipoyl dehydrogenase, mitochondrial	O08749
Dlg1	-0.0005	0.07	0.69	-0.14	0.5	0.03	0.87	-0.07	0.57	Disks large homolog 1	E9Q9H0
Dlg2	-15.2308	1.24	0.0180	-1.76	0.0001	-0.35	0.33	-0.52	0.0310	Disks large homolog 2	A0A571BEK3
Dlg3	-0.0191	0.53	0.61	-0.59	0.52	-0.31	0.17	-0.06	0.87	Disks large homolog 3	A2BEE9
Dlg4	-18.8026	1.55	0.071	-2.64	0.0001	-0.55	0.066	-1.09	0.0001	Disks large homolog 4	A0A338P6E5
Dlgap1	-1.2602	3.36	0.67	-4.86	0.36	-1.73	0.32	-1.49	0.54	Disks large-associated protein 1	Q9D415
Dlgap2	-1.0300	0.73	0.35	-1.58	0.0110	-0.41	0.37	-0.85	0.072	Disks large-associated protein 2	Q0VF59
Dlgap3	-30.3718	2.28	0.076	-3.48	0.0004	-0.20	0.35	-1.20	0.0110	Disks large-associated protein 3	B1AS06
Dlgap4	-74.2828	9.38	0.2	-10.38	0.081	3.91	0.94	-1.01	0.51	Disks large-associated protein 4	B1AZP2

Table S2: Differentially expressed proteins in cerebral cortex *(continued)*

gene	$\pi_{TW/EW} \times \pi_{TF/TW}$	log2FC TW/EW	padj TW/EW	log2FC TF/TW	padj TF/TW	log2FC EF/EW	padj EF/EW	log2FC TF/EW	padj TF/EW	description	accession
Dlst	-2.0555	-0.34	0.0020	0.56	0.0001	0.11	0.86	0.22	0.0180	Dihydrolipoyllysine-residue succinyltransferase component of 2-oxoglutarate dehydrogenase complex, mitochondrial	Q9D2G2
Dmac2l	-0.1469	0.26	0.39	-0.89	0.0280	-0.15	0.72	-0.63	0.16	ATP synthase subunit s, mitochondrial	Q9CRA7
Dmtn	-0.5252	0.29	0.2	-0.96	0.0020	0.71	0.0440	-0.67	0.0410	Dematin	Q9WV69
Dmwd	-0.0001	0.06	0.71	-0.09	0.67	-0.03	0.7	-0.03	0.89	Dystrophia myotonica WD repeat-containing protein	E9Q6J8
Dmxl1	0.0000	0.00	0.49	0.29	0.48	-0.39	0.14	0.29	0.054	DmX-like protein 1	F8WGX5
Dmxl2	-0.0749	-0.27	0.63	0.77	0.0160	0.06	0.97	0.49	0.0250	DmX-like protein 2	B0V2P5
Dnah8	-0.0262	0.85	0.85	-1.63	0.54	-1.22	0.28	-0.79	0.74	Dynein heavy chain 8, axonemal	Q91XQ0
Dnaja1	-0.0036	0.55	0.53	-0.22	0.78	-1.03	0.32	0.33	0.64	DnaJ homolog subfamily A member 1	P63037
Dnaja2	0.0000	0.12	1	-0.06	0.97	-0.39	0.11	0.06	0.49	DnaJ homolog subfamily A member 2	Q9QYJ0
Dnaja3	-4.8340	1.04	0.0190	-1.32	0.0090	-0.05	0.81	-0.29	0.93	DnaJ homolog subfamily A member 3, mitochondrial	Q99M87
Dnaja4	-0.0247	0.29	0.0400	-0.13	0.34	0.08	0.57	0.16	0.16	DnaJ homolog subfamily A member 4	Q9JMC3
Dnajb1	-0.1740	-0.44	0.63	1.33	0.0330	-0.06	0.91	0.89	0.11	DnaJ homolog subfamily B member 1	Q3TYL7
Dnajb11	-0.0216	-0.10	0.43	0.46	0.052	0.23	0.88	0.36	0.075	DnaJ homolog subfamily B member 11	Q99KV1
Dnajb14	-0.2976	0.61	0.19	-0.85	0.16	-0.58	0.18	-0.24	0.65	DnaJ homolog subfamily B member 14	Q149L6
Dnajb2	0.0022	-0.41	0.37	-0.12	0.79	-0.05	0.97	-0.53	0.052	DnaJ homolog subfamily B member 2	Q9QYI5
Dnajb4	-1.1734	2.53	0.065	-1.82	0.61	2.03	0.081	0.71	0.38	DnaJ homolog subfamily B member 4	Q9D832
Dnajb5	-0.1063	0.33	0.67	-1.82	0.096	-0.18	0.93	-1.49	0.094	DnaJ homolog subfamily B member 5	A2AG30
Dnajc10	-0.0099	0.61	0.64	-0.67	0.75	0.89	0.089	-0.06	0.68	DnaJ homolog subfamily C member 10	Q9DC23
Dnajc11	0.0811	0.29	0.2	0.40	0.1	0.17	1	0.70	0.0080	DnaJ homolog subfamily C member 11	Q5U458
Dnajc13	-0.4381	0.35	0.0440	-0.55	0.0210	0.14	0.83	-0.20	0.38	DnaJ heat shock protein family (Hsp40) member C13	D4AFX7
Dnajc19	0.0004	0.24	0.055	0.03	0.9	0.20	0.51	0.27	0.47	Mitochondrial import inner membrane translocase subunit TIM14	D3Z5K6
Dnajc3	0.0000	0.00	0.48	0.51	0.0001	0.06	0.32	0.50	0.0001	DnaJ homolog subfamily C member 3	Q91YW3
Dnajc6	-0.1403	-0.12	0.44	0.82	0.0001	0.13	0.55	0.70	0.0060	Putative tyrosine-protein phosphatase auxilin	Q80TZ3
Dnajc8	-152.7034	9.65	0.0280	-8.09	0.055	0.23	0.66	1.57	0.22	DnaJ homolog subfamily C member 8	A2ALF3
Dner	0.0007	0.04	0.87	0.48	0.26	0.11	0.6	0.52	0.16	Delta and Notch-like epidermal growth factor-related receptor	Q8JZM4
Dnm1	0.0917	0.28	0.14	0.40	0.11	-0.13	0.87	0.68	0.0036	Dynamin GTPase	A0A0J9YUN4
Dnm1l	0.0281	0.37	0.19	0.18	0.26	0.20	0.47	0.55	0.088	Dynamin-1-like protein	Q8K1M6
Dnm3	0.0000	0.48	0.0002	0.13	1	-0.06	0.81	0.61	0.0001	Dynamin-3	Q8BZ98
Dock1	0.0055	-0.30	0.69	-0.21	0.29	0.01	0.76	-0.51	0.22	Dedicator of cytokinesis protein 1	Q8BUR4
Dock3	-0.4464	0.46	0.066	-0.49	0.0210	-0.10	0.8	-0.03	0.82	Dedicator of cytokinesis protein 3	Q8CIQ7

Table S2: Differentially expressed proteins in cerebral cortex *(continued)*

gene	$\pi_{TW/EW} \times \pi_{TF/TW}$	log2FC TW/EW	padj TW/EW	log2FC TF/TW	padj TF/TW	log2FC EF/EW	padj EF/EW	log2FC TF/EW	padj TF/EW	description	accession
Dock5	-0.0226	0.40	0.92	-2.16	0.19	-0.09	0.88	-1.76	0.12	Dedicator of cytokinesis protein 5	B2RY04
Dock9	0.0014	0.04	0.83	0.51	0.15	-0.16	0.81	0.55	0.15	Dedicator of cytokinesis protein 9	E9QMR2
Dpp10	-0.0393	-0.20	0.29	0.24	0.0300	0.09	0.81	0.04	0.5	Inactive dipeptidyl peptidase 10	A0A5F8MPK1
Dpp6	-0.0891	-0.53	0.23	0.45	0.26	-0.05	0.74	-0.08	0.77	Dipeptidyl aminopeptidase-like protein 6	Q5U4C2
Dpysl2	-0.0002	-0.03	0.54	0.08	0.49	0.08	0.69	0.04	0.98	Dihydropyrimidinase-related protein 2	O08553
Dpysl3	-0.0049	0.15	0.0280	-0.02	0.088	0.02	1	0.13	0.43	Dihydropyrimidinase-related protein 3	E9PWE8
Dpysl4	-0.0057	-0.06	0.31	0.33	0.27	-0.09	0.44	0.28	0.0053	Dihydropyrimidinase-related protein 4	O35098
Dpysl5	0.0016	0.26	0.2	0.02	0.36	0.07	0.77	0.28	0.52	Dihydropyrimidinase-related protein 5	Q9EQF6
Dsp	-0.0021	-1.39	0.0230	0.21	0.99	-0.63	0.25	-1.18	0.0200	Desmoplakin	E9Q557
Dtna	-1.9005	0.86	0.0260	-0.81	0.0190	0.10	0.93	0.05	1	Dystrobrevin	A0A1Y7VL34
Dtymk	0.0760	-0.20	0.8	-3.15	0.057	-0.53	0.68	-3.35	0.0220	Thymidylate kinase	P97930
Dync1h1	-0.0010	-0.01	0.0030	0.12	0.47	0.06	0.43	0.11	0.076	Cytoplasmic dynein 1 heavy chain 1	Q9JHU4
Dync1i1	0.0039	0.22	0.17	0.33	0.85	0.34	0.19	0.55	0.0310	Cytoplasmic dynein 1 intermediate chain 1	D3Z0M6
Dync1i2	-0.0125	-0.13	0.43	0.34	0.17	0.23	0.2	0.22	0.41	Cytoplasmic dynein 1 intermediate chain 2	A2BFF8
Dync1li1	-0.0017	-0.09	0.71	0.23	0.28	0.03	0.76	0.14	0.14	Cytoplasmic dynein 1 light intermediate chain 1	Q8R1Q8
Dync1li2	0.0000	-0.05	0.82	0.17	0.92	0.20	0.57	0.11	0.71	Cytoplasmic dynein 1 light intermediate chain 2	Q6PDL0
Dynlrb1	0.0007	-0.28	0.3	-0.02	0.57	-0.12	0.9	-0.31	0.17	Dynein light chain roadblock	A2AVR9
Dyrk1a	-0.0989	0.59	0.17	-0.44	0.32	0.13	0.48	0.15	0.5	Dual-specificity kinase	F6U6X3
Dzank1	-0.4138	0.97	0.4	-1.21	0.13	0.02	0.9	-0.25	0.58	Double zinc ribbon and ankyrin repeat-containing protein 1	E9Q4Z6
Ech1	0.0010	-0.09	0.55	-0.12	0.43	-0.28	0.079	-0.21	0.15	Delta(3,5)-Delta(2,4)-dienoyl-CoA isomerase, mitochondrial	O35459
Echs1	0.0000	0.06	0.44	0.16	0.97	-1.04	0.0001	0.22	1	Enoyl-CoA hydratase, mitochondrial	Q8BH95
Eci1	-0.1525	-0.55	0.081	0.33	0.17	0.26	0.26	-0.22	0.32	Enoyl-CoA delta isomerase 1, mitochondrial	P42125
Eci2	-0.0349	0.69	0.28	-0.19	0.33	-1.12	0.5	0.50	0.38	Enoyl-CoA delta isomerase 2	Q3TCD4
Ecpas	-0.1627	0.78	0.25	-0.70	0.32	-0.71	0.62	0.08	0.92	Proteasome adapter and scaffold protein ECM29	Q6PDI5
Edc4	-0.0031	0.83	0.2	-0.24	0.95	0.79	0.16	0.58	0.15	Enhancer of mRNA-decapping protein 4	A0A0R4J1Q0
Edf1	0.0662	0.21	0.16	0.26	0.0300	0.40	0.0470	0.47	0.0044	Endothelial differentiation-related factor 1	Q9JMG1
Eea1	-0.0062	-0.25	0.64	0.45	0.52	0.29	0.54	0.20	0.6	Early endosome antigen 1	Q8BL66
Eef1a2	0.0012	0.18	0.48	0.07	0.5	0.15	0.096	0.24	0.96	Elongation factor 1-alpha 2	P62631
Eef1b	-0.0304	-0.08	0.29	0.28	0.0030	0.04	0.87	0.20	0.0070	Elongation factor 1-beta	O70251
Eef1d	-1.4808	1.67	0.087	-1.31	0.23	-7.10	0.17	0.36	0.5	Elongation factor 1-delta	A0A0R4J1E2
Eef1g	0.0029	0.05	0.4	0.26	0.28	0.07	0.85	0.31	0.11	Elongation factor 1-gamma	Q9D8N0
Eef2	0.0019	-0.12	0.63	-0.10	0.17	-0.18	0.0100	-0.21	0.0018	Elongation factor 2	P58252

Table S2: Differentially expressed proteins in cerebral cortex *(continued)*

gene	$\pi_{\mathrm{TW/EW}}\times\pi_{\mathrm{TF/TW}}$	log2FC TW/EW	padj TW/EW	log2FC TF/TW	padj TF/TW	log2FC EF/EW	padj EF/EW	log2FC TF/EW	padj TF/EW	description	accession
Efhd2	0.0103	-0.06	0.39	-0.27	0.0280	0.51	0.17	-0.33	0.4	EF-hand domain-containing protein D2	Q8C845
Efl1	-0.0020	-0.74	0.25	0.11	0.91	-0.37	0.76	-0.63	0.37	Elongation factor-like GTPase 1	Q8C0D5
Efr3a	-0.5220	0.71	0.11	-0.80	0.11	0.32	0.44	-0.10	0.8	Protein EFR3 homolog A	A0A1D5RLL3
Efr3b	-0.3320	-0.50	0.0280	0.35	0.06	0.13	0.47	-0.15	0.49	Protein EFR3 homolog B	Q6ZQ18
Ehbp1	-0.4057	1.26	0.17	-0.49	0.14	0.72	0.76	0.77	0.75	EH domain-binding protein 1	Q69ZW3
Ehd1	-0.0071	-0.17	0.34	0.20	0.36	-0.15	0.3	0.03	0.91	EH domain-containing protein 1	Q9WVK4
Ehd2	-0.2395	0.98	0.12	-0.48	0.28	-0.16	0.62	0.50	0.26	EH domain-containing protein 2	Q8BH64
Ehd3	-0.0283	0.70	0.31	-0.37	0.61	0.63	0.26	0.33	0.49	EH domain-containing protein 3	Q9QXY6
Ehd4	0.1558	0.60	0.11	0.45	0.25	-0.16	0.93	1.04	0.0120	EH domain-containing protein 4	Q9EQP2
Eif2a	0.0168	0.38	0.48	0.33	0.38	0.55	0.19	0.71	0.11	Eukaryotic translation initiation factor 2A	Q8BJW6
Eif2s1	-0.1147	0.27	0.0300	-0.21	0.0470	0.05	0.55	0.06	0.77	Eukaryotic translation initiation factor 2 subunit 1	Q6ZWX6
Eif2s2	-0.0661	0.41	0.18	-0.30	0.19	-0.12	0.57	0.11	0.71	Eukaryotic translation initiation factor 2 subunit 2	Q99L45
Eif2s3x	-0.0148	0.57	0.87	-0.89	0.33	-0.25	0.86	-0.32	0.45	Eukaryotic translation initiation factor 2 subunit 3, X-linked	Q9Z0N1
Eif3a	0.0006	0.24	0.29	0.20	0.95	-0.47	0.44	0.44	0.34	Eukaryotic translation initiation factor 3 subunit A	P23116
Eif3b	-0.0005	-0.15	0.94	0.38	0.5	0.20	0.59	0.23	0.57	Eukaryotic translation initiation factor 3 subunit B	Q8JZQ9
Eif3c	-0.0008	-0.01	0.73	0.54	0.073	-0.95	0.0290	0.53	0.064	Eukaryotic translation initiation factor 3 subunit C	Q8R1B4
Eif3d	-0.9017	0.91	0.051	-0.62	0.058	-0.37	0.69	0.29	0.86	Eukaryotic translation initiation factor 3 subunit D	O70194
Eif3e	-0.2729	-0.28	0.16	0.73	0.0210	-0.09	0.64	0.45	0.19	Eukaryotic translation initiation factor 3 subunit E	P60229
Eif3f	-0.0339	0.82	0.19	-0.17	0.46	0.69	0.24	0.65	0.55	Eukaryotic translation initiation factor 3 subunit F	Q9DCH4
Eif3g	-0.1939	-0.40	0.093	0.42	0.076	-0.19	0.66	0.02	0.85	Eukaryotic translation initiation factor 3 subunit G	Q9Z1D1
Eif3h	-4.5465	-1.55	0.0160	1.23	0.0470	-0.61	0.37	-0.32	0.7	Eukaryotic translation initiation factor 3 subunit H	Q91WK2
Eif3i	-0.0799	-0.18	0.29	0.51	0.0240	-0.11	0.65	0.32	0.23	Eukaryotic translation initiation factor 3 subunit I	Q9QZD9
Eif3l	-1.5462	-0.32	0.0210	0.72	0.0001	-0.25	0.054	0.41	0.0010	Eukaryotic translation initiation factor 3 subunit L	Q8QZY1
Eif4a2	-0.0047	0.41	0.92	-1.14	0.53	-1.15	0.59	-0.73	0.63	Eukaryotic initiation factor 4A-II	P10630
Eif4a3	-0.0035	0.38	0.22	-0.13	0.78	-0.30	0.69	0.26	0.2	Eukaryotic initiation factor 4A-III	Q91VC3
Eif4b	-0.0695	-0.19	0.59	0.94	0.0200	-0.12	0.91	0.75	0.0330	Eukaryotic translation initiation factor 4B	Q8BGD9

Table S2: Differentially expressed proteins in cerebral cortex *(continued)*

gene	$\pi_{TW/EW}\times \pi_{TF/TW}$	log2FC TW/EW	padj TW/EW	log2FC TF/TW	padj TF/TW	log2FC EF/EW	padj EF/EW	log2FC TF/EW	padj TF/EW	description	accession
Eif4e	-0.0868	-0.29	0.11	0.30	0.091	-0.50	0.25	0.00	0.96	Eukaryotic translation initiation factor 4E	P63073
Eif4g1	0.0364	0.16	0.36	0.33	0.0280	0.45	0.17	0.50	0.095	Eukaryotic translation initiation factor 4 gamma 1	Q6NZJ6
Eif4g2	0.0000	0.02	0.99	0.15	0.59	0.22	0.71	0.17	0.69	Eukaryotic translation initiation factor 4 gamma 2	F7CBP1
Eif4g3	-0.0013	-0.84	0.78	0.07	0.62	0.63	0.28	-0.77	0.9	Eukaryotic translation initiation factor 4 gamma 3	A0A0N4SVL0
Eif4h	0.0000	-0.11	0.68	-0.04	0.94	-0.30	0.67	-0.15	0.64	Eukaryotic translation initiation factor 4H	Q9WUK2
Eif5	0.0000	0.04	0.66	-0.01	0.96	0.31	0.12	0.03	0.85	Eukaryotic translation initiation factor 5	Q8BVV6
Eif5a	-0.0010	-0.10	0.47	0.25	0.75	-0.28	0.19	0.15	0.92	Eukaryotic translation initiation factor 5A (Fragment)	A0A0A0MQM0
Eif6	-5.7090	-4.50	0.3	4.03	0.25	-0.22	0.97	-0.47	0.98	Eukaryotic translation initiation factor 6	O55135
Eipr1	0.0001	0.20	0.38	0.26	0.99	0.42	0.24	0.46	0.26	EARP and GARP complex-interacting protein 1	Q8K0G5
Elavl1	-0.0372	0.15	0.69	-0.94	0.0230	-0.60	0.22	-0.78	0.0180	ELAV-like protein 1	P70372
Elavl3	-0.0441	0.50	0.62	-1.01	0.38	-0.50	0.85	-0.51	0.72	ELAV-like protein 3	Q60900
Elfn1	0.0000	1.05	0.46	0.06	1	0.36	0.87	1.11	0.25	Protein ELFN1	Q8C8T7
Elfn2	-2.4443	1.12	0.079	-1.47	0.0450	-0.62	0.51	-0.35	0.63	Protein phosphatase 1 regulatory subunit 29	Q68FM6
Elmo1	-0.0123	-0.94	0.41	0.37	0.81	0.14	0.77	-0.57	0.66	Engulfment and cell motility protein 1	Q8BPU7
Elmo2	-0.0004	0.07	0.85	-0.30	0.57	0.40	0.27	-0.23	0.65	Engulfment and cell motility protein 2	Q8BHL5
Elob	0.0018	0.09	0.81	0.19	0.075	0.48	0.0110	0.29	0.16	Elongin-B	A0A3B2WBM3
Eloc	-0.0010	-0.10	0.55	0.13	0.52	-0.39	0.31	0.02	1	Elongin-C (Fragment)	A0A087WQE6
Elp2	0.0615	-0.44	0.34	-0.73	0.39	-0.05	0.98	-1.17	0.19	Elongator complex protein 2	Q91WG4
Emb	0.0609	0.52	0.4	0.37	0.16	0.71	0.19	0.89	0.081	Embigin	P21995
Emc1	-0.0026	-0.06	0.72	0.89	0.46	0.58	0.0019	0.83	0.47	ER membrane protein complex subunit 1	Q8C7X2
Emc10	-0.0084	1.17	0.058	-0.06	0.8	1.30	0.0060	1.11	0.066	ER membrane protein complex subunit 10	A0A0X1KG67
Emc4	-0.0513	-0.30	0.74	1.42	0.12	-0.31	0.78	1.12	0.16	ER membrane protein complex subunit 4	Q9CZX9
Emc7	-0.0001	-0.02	0.93	0.62	0.47	-0.21	0.68	0.60	0.43	ER membrane protein complex subunit 7	Q9EP72
Emd	0.0027	-0.09	0.82	-0.96	0.43	-1.23	0.0430	-1.04	0.2	Emerin	B7FAU5
Eml1	-7.8310	4.08	0.0270	-1.59	0.17	1.97	0.44	2.49	0.46	Echinoderm microtubule-associated protein-like 1	D3Z4J9
Eml2	0.0000	0.09	0.73	0.01	1	0.26	0.42	0.10	0.55	Echinoderm microtubule-associated protein-like 2	E9QK48
Eml5	0.0002	0.20	0.58	0.08	0.88	0.26	0.47	0.28	0.21	Echinoderm microtubule-associated protein-like 5	A0A217FL67
Eml6	-0.0319	-3.62	0.27	0.18	0.82	-3.13	0.58	-3.44	0.61	Echinoderm microtubule-associated protein-like 6	Q5SQM0
Enah	-0.0965	0.65	0.79	-2.48	0.26	-0.62	1	-1.83	0.3	Protein enabled homolog	E9QKR1

Table S2: Differentially expressed proteins in cerebral cortex *(continued)*

gene	$\pi_{TW/EW}\times$ $\pi_{TF/TW}$	log2FC TW/EW	padj TW/EW	log2FC TF/TW	padj TF/TW	log2FC EF/EW	padj EF/EW	log2FC TF/EW	padj TF/EW	description	accession
Endod1	-22.5662	7.67	0.0490	-6.30	0.44	0.23	0.66	1.37	0.4	Endonuclease domain-containing 1 protein	Q8C522
Endog	0.0005	0.14	0.64	0.08	0.57	0.19	0.67	0.23	0.17	Endonuclease G, mitochondrial	O08600
Eno1	-0.0439	-0.37	0.0001	0.06	0.32	-0.41	0.0001	-0.31	0.0001	Alpha-enolase	P17182
Eno2	-0.0021	-0.64	0.0050	0.16	0.98	-0.23	0.27	-0.48	0.0048	Gamma-enolase	P17183
Enpp6	-0.0027	0.08	0.78	-0.98	0.48	-0.61	0.19	-0.89	0.2	Glycerophosphocholine cholinephosphodiesterase ENPP6	Q8BGN3
Ensa	0.0133	-0.43	0.3	-0.18	0.47	-0.09	0.65	-0.60	0.053	Alpha-endosulfine	P60840
Eny2	0.0000	0.00	0.85	0.10	0.48	0.39	0.27	0.11	0.91	Transcription and mRNA export factor ENY2	A0A2I3BRK6
Epb41	-0.0052	0.20	0.59	-0.31	0.43	0.00	0.77	-0.11	0.77	Band 4.1	A2A841
Epb41l2	-0.0019	-0.08	0.69	0.25	0.25	0.30	0.093	0.17	0.2	Band 4.1-like protein 2	O70318
Epb41l3	-11.6063	1.96	0.23	-4.83	0.0120	0.32	0.9	-2.87	0.085	Band 4.1-like protein 3	A0A5F8MPE1
Epdr1	0.0317	0.87	0.0130	0.07	0.53	0.36	0.76	0.93	0.1	Mammalian ependymin-related protein 1	Q99M71
Epha4	-0.0001	-0.78	0.76	0.05	0.97	0.19	0.68	-0.73	0.69	Ephrin type-A receptor 4	Q03137
Epn2	0.0029	0.08	0.56	0.43	0.46	0.40	0.4	0.51	0.27	Epsin-2	J3QNT7
Eprs1	0.0001	0.11	0.87	0.11	0.85	-0.07	0.85	0.22	0.95	Bifunctional glutamate/proline–tRNA ligase	Q8CGC7
Eps15l1	0.0010	0.12	0.27	0.34	0.91	0.35	0.0200	0.46	0.0120	Epidermal growth factor receptor substrate 15-like 1	Q60902
Eps8	-0.6339	0.88	0.11	-0.88	0.14	0.34	0.89	0.00	0.75	Epidermal growth factor receptor kinase substrate 8	Q08509
Erbb4	-0.0078	-0.10	0.72	0.52	0.089	0.01	1	0.42	0.17	Receptor tyrosine-protein kinase erbB-4	Q61527
Erbin	-0.0064	0.23	0.5	-0.22	0.38	0.65	0.062	0.01	0.98	Erbb2ip protein	B2RUJ2
Erc1	-0.9740	0.97	0.083	-0.83	0.076	0.24	0.97	0.14	0.73	ELKS/Rab6-interacting/CAST family member 1	V9GXP8
Erc2	-2.9009	1.05	0.53	-3.34	0.0010	-1.65	0.0010	-2.29	0.0002	ERC protein 2	Q3UHT7
Ergic1	-0.6551	-0.70	0.34	1.44	0.0410	-1.14	0.19	0.74	0.0370	Endoplasmic reticulum-Golgi intermediate compartment protein 1	A0A494BAK4
Erlec1	-0.0612	-0.46	0.62	0.86	0.18	-1.00	0.46	0.40	0.65	Endoplasmic reticulum lectin 1	Q8VEH8
Erlin2	-0.0562	0.41	0.31	-0.35	0.17	0.03	0.86	0.06	0.85	Erlin-2	Q8BFZ9
Erp29	0.0243	-0.21	0.29	-0.28	0.17	-0.39	0.37	-0.49	0.0190	Endoplasmic reticulum resident protein 29	P57759
Erp44	-0.0015	-0.08	0.9	0.42	0.1	0.02	0.62	0.34	0.087	Endoplasmic reticulum resident protein 44	Q9D1Q6
Esyt2	0.0032	-0.08	0.75	-0.41	0.17	-0.68	0.19	-0.49	0.088	Extended synaptotagmin-2	Q3TZZ7
Etfa	-0.0609	0.29	0.38	-0.50	0.1	-0.09	0.79	-0.21	0.27	Electron transfer flavoprotein subunit alpha, mitochondrial	Q99LC5
Etfb	-0.1256	0.89	0.28	-0.53	0.33	0.57	0.57	0.36	0.84	Electron transfer flavoprotein subunit beta	Q9DCW4
Etfdh	-0.1190	0.24	0.29	-0.68	0.0440	-0.29	0.2	-0.43	0.12	Electron transfer flavoprotein-ubiquinone oxidoreductase, mitochondrial	Q921G7
Etl4	-16.2843	1.23	0.0270	-2.11	0.0001	-0.72	0.12	-0.88	0.0200	Enhancer trap locus 4	E9QAU4

Table S2: Differentially expressed proteins in cerebral cortex *(continued)*

gene	$\pi_{TW/EW} \times \pi_{TF/TW}$	log2FC TW/EW	padj TW/EW	log2FC TF/TW	padj TF/TW	log2FC EF/EW	padj EF/EW	log2FC TF/EW	padj TF/EW	description	accession
Eva1a	-2.4235	1.39	0.25	-4.95	0.26	-7.14	0.36	-3.56	0.64	Protein eva-1 homolog A	Q91WM6
Evl	-0.0247	0.17	0.46	-0.45	0.11	-0.45	0.75	-0.28	0.9	Ena/VASP-like protein (Fragment)	A0A1Y7VJA2
Exoc1	0.1826	0.22	0.2	0.44	0.0020	-0.18	0.42	0.66	0.0001	Exocyst complex component 1	Q5PPR2
Exoc2	0.3476	1.02	0.18	0.37	0.058	1.11	0.11	1.38	0.0010	Exocyst complex component 2	Q9D4H1
Exoc3	-0.0707	-0.14	0.52	0.58	0.0009	0.13	0.18	0.44	0.0050	Exocyst complex component 3	Q6KAR6
Exoc4	0.0000	0.07	0.79	0.23	1	0.05	0.92	0.30	0.0130	Exocyst complex component 4	O35382
Exoc5	0.0717	0.25	0.26	0.49	0.1	0.16	0.68	0.74	0.0200	Exocyst complex component 5	Q3TPX4
Exoc6b	0.0001	0.12	0.62	0.04	0.76	-0.02	0.96	0.15	0.54	Exocyst complex component 6B	A6H5Z3
Exoc7	0.0000	-0.06	0.34	0.33	1	0.13	0.2	0.27	1	Exocyst complex component 7	A2AAN0
Exoc8	0.0363	0.20	0.35	0.35	0.073	-0.25	0.18	0.55	0.0003	Exocyst complex component 8	Q6PGF7
Exog	0.0005	2.29	0.5	0.03	0.95	0.03	0.9	2.32	0.46	Nuclease EXOG, mitochondrial	Q8C163
Ezr	-0.0648	0.52	0.28	-0.19	0.065	0.44	0.26	0.33	0.87	Ezrin	P26040
F8a1	-0.0102	0.44	0.79	-0.90	0.56	1.41	0.0430	-0.46	0.81	40-kDa huntingtin-associated protein	Q00558
Fabp3	0.0002	-0.30	0.97	-0.25	0.64	-0.90	0.56	-0.55	0.49	Fatty acid-binding protein, heart	P11404
Fahd2a	-0.1292	0.33	0.15	-0.37	0.052	-0.44	0.33	-0.05	0.9	Fumarylacetoacetate hydrolase domain-containing 2A	A0A0R4J094
Fam117b	-0.1544	1.66	0.0150	-0.16	0.48	0.13	0.97	1.50	0.0200	Protein FAM117B	Q3U3E2
FAM120A	-0.0264	0.36	0.58	-0.53	0.26	-0.07	0.98	-0.17	0.47	Constitutive coactivator of PPAR-gamma-like protein 1	Q6A0A9
Fam120c	-0.1559	0.72	0.53	-1.23	0.23	0.31	0.4	-0.51	0.49	Constitutive coactivator of PPAR-gamma-like protein 2	Q8C3F2
Fam126b	0.0008	0.15	0.38	0.04	0.5	0.05	0.91	0.19	0.98	Protein FAM126B	Q8C729
Fam131b	0.0097	-0.10	0.71	-1.32	0.32	0.66	0.28	-1.42	0.26	Protein FAM131B	E9Q8P8
Fam171a1	-0.0877	0.22	0.28	-0.45	0.0250	-0.62	0.14	-0.23	0.05	Family with sequence similarity 171, member A1	A2ATK9
Fam171a2	0.0000	-0.17	0.5	0.12	1	0.20	0.07	-0.05	0.64	Protein FAM171A2	A2A699
Fam171b	-0.0069	0.36	0.59	-0.40	0.62	0.33	0.33	-0.04	0.93	Protein FAM171B	Q14CH0
Fam193a	0.0021	0.23	0.83	0.71	0.69	0.42	0.75	0.94	0.46	Protein FAM193A	M0QWZ1
Fam81a	-1.5417	1.28	0.46	-2.18	0.0230	-0.87	0.0460	-0.90	0.0210	Protein FAM81A	Q3UXZ6
Fam98b	-0.0313	0.39	0.39	-0.43	0.35	-0.07	0.94	-0.04	0.77	Protein FAM98B	Q80VD1
Farp1	-0.9271	0.35	0.13	-0.98	0.0009	-0.24	0.5	-0.63	0.0011	FERM, ARHGEF and pleckstrin domain-containing protein 1	F8VPU2
Farsa	-0.0499	0.07	0.41	-0.46	0.0001	-0.24	0.0210	-0.39	0.0020	Phenylalanine–tRNA ligase alpha subunit	Q8C0C7
Farsb	-0.0226	0.12	0.092	-0.16	0.073	-0.13	0.83	-0.04	0.77	Phenylalanine–tRNA ligase beta subunit	Q9WUA2
Fasn	-0.0005	-0.17	0.68	0.04	0.35	0.00	0.54	-0.14	0.14	Fatty acid synthase	P19096
Fbxl16	-0.0045	0.19	0.48	-0.22	0.46	0.21	0.57	-0.04	0.9	F-box/LRR-repeat protein 16	A2RT62
Fbxo2	-0.0254	0.25	0.51	-0.61	0.27	-0.40	0.17	-0.37	0.42	F-box only protein 2	Q80UW2
Fbxo41	-0.0063	0.30	0.97	-1.17	0.0430	-0.17	0.77	-0.87	0.0280	F-box only protein 41	Q6NS60

Table S2: Differentially expressed proteins in cerebral cortex *(continued)*

gene	$\pi_{TW/EW}\times$ $\pi_{TF/TW}$	log2FC TW/EW	padj TW/EW	log2FC TF/TW	padj TF/TW	log2FC EF/EW	padj EF/EW	log2FC TF/EW	padj TF/EW	description	accession
Fbxo6	-0.0232	-0.78	0.082	0.20	0.73	0.52	0.27	-0.58	0.23	F-box only protein 6	Q9QZN4
Fcho2	-0.0374	-0.19	0.8	0.78	0.0025	0.29	0.54	0.59	0.062	F-BAR domain only protein 2	Q3UQN2
Fdxr	-17.9045	8.26	0.089	-4.65	0.36	3.70	0.38	3.60	0.53	NADPH:adrenodoxin oxidoreductase, mitochondrial	Q61578
Fech	-0.2937	0.51	0.19	-0.32	0.0032	0.77	0.1	0.20	0.8	Ferrochelatase	A0A494BAI5
Fer	-0.1238	3.42	0.72	-3.35	0.84	-3.50	0.47	0.06	0.89	Tyrosine-protein kinase Fer	P70451
Fga	0.0000	0.09	0.5	0.02	0.9	0.16	0.49	0.11	0.65	Fibrinogen alpha chain	E9PV24
Fh	0.0010	-0.07	0.81	-0.23	0.2	-0.10	0.5	-0.30	0.2	Fumarate hydratase, mitochondrial	P97807
Fibp	0.0032	0.26	0.37	0.12	0.58	0.04	0.76	0.38	0.0280	Acidic fibroblast growth factor intracellular-binding protein	Q8K2D8
Fkbp3	-0.0054	-0.04	0.85	0.48	0.0001	0.21	0.0060	0.44	0.0001	Peptidyl-prolyl cis-trans isomerase FKBP3	Q62446
Fkbp8	0.0002	0.05	0.77	0.11	0.55	-0.02	0.92	0.16	0.27	Peptidyl-prolyl cis-trans isomerase FKBP8	O35465
Flii	-0.3251	1.11	0.23	-0.83	0.28	1.34	0.0012	0.28	0.75	Protein flightless-1 homolog	Q9JJ28
Flna	-1.2092	0.36	0.22	-1.45	0.0003	-0.19	0.91	-1.09	0.055	Filamin, alpha	B7FAU9
Flot1	-0.0024	-0.26	0.62	0.22	0.63	0.44	0.058	-0.04	0.93	Flotillin-1	O08917
Flot2	-0.0049	-0.48	0.19	0.28	0.89	0.38	0.064	-0.20	0.0430	Flotillin	Q5SS83
Flrt2	0.0000	0.63	0.0170	0.00	0.83	0.27	0.17	0.63	0.0320	Leucine-rich repeat transmembrane protein FLRT2	Q8BLU0
Fmn2	-0.0104	0.32	0.46	-0.33	0.51	0.48	0.47	0.00	0.88	Formin-2	Q9JL04
Fmnl2	-0.0973	0.43	0.37	-0.75	0.2	0.80	0.0360	-0.32	0.6	Formin-like protein 2	A2APV2
Fmr1	-223.2321	6.23	0.0410	-8.61	0.0010	-2.04	0.26	-2.38	0.12	Synaptic functional regulator FMR1	D3Z6U8
Fn3k	0.0155	-0.18	0.059	-0.13	0.29	0.23	0.0300	-0.32	0.0010	Fructosamine-3-kinase	Q9ER35
Foxred1	-0.0015	0.07	0.75	-0.39	0.37	0.43	0.0370	-0.32	0.41	FAD-dependent oxidoreductase domain-containing protein 1	Q3TQB2
Frmpd3	-0.0002	0.22	0.96	-0.76	0.88	0.14	0.85	-0.53	0.93	FERM and PDZ domain-containing 3	A0A140LIW3
Fry	-0.0362	0.31	0.46	-0.72	0.33	-0.35	0.71	-0.40	0.79	Protein furry homolog	A0A5F8MPN1
Fscn1	0.0066	-0.05	0.39	-0.20	0.0250	-0.04	0.94	-0.25	0.5	Fascin	Q61553
Fsd1	0.0001	0.08	0.8	0.06	0.67	-0.29	0.63	0.14	0.64	Fibronectin type III and SPRY domain-containing protein 1	Q7TPM6
Fsd1l	0.0000	-0.02	0.92	-0.02	0.84	0.33	0.17	-0.04	0.7	FSD1-like protein	J3QM95
Fsip2	-0.0074	0.23	0.32	-0.21	0.49	-0.06	0.85	0.03	0.87	Fibrous sheath-interacting protein 2	A2ARZ3
Fubp1	-0.0985	-0.36	0.47	0.45	0.0140	-0.10	0.85	0.09	0.56	Far upstream element-binding protein 1	Q3UUU2
Fubp3	-0.0011	-0.11	0.75	0.19	0.38	0.19	0.42	0.08	0.67	Far upstream element (FUSE)-binding protein 3	A2AJ72
Fxr1	-0.0066	0.28	0.27	-0.15	0.53	-0.06	0.84	0.13	0.48	Fragile X mental retardation syndrome-related protein 1	Q61584
Fyn	0.0000	-0.05	0.83	0.00	0.89	0.19	0.49	-0.05	0.93	Tyrosine-protein kinase Fyn	P39688
G3bp1	-0.0008	0.22	0.87	-0.38	0.71	0.85	0.54	-0.16	0.78	Ras GTPase-activating protein-binding protein 1	P97855

Table S2: Differentially expressed proteins in cerebral cortex *(continued)*

gene	$\pi_{TW/EW} \times \pi_{TF/TW}$	log2FC TW/EW	padj TW/EW	log2FC TF/TW	padj TF/TW	log2FC EF/EW	padj EF/EW	log2FC TF/EW	padj TF/EW	description	accession
G3bp2	0.0063	0.85	0.25	0.55	0.95	0.15	0.64	1.40	0.28	Ras GTPase-activating protein-binding protein 2	P97379
G6pdx	-0.0550	-0.28	0.0280	0.17	0.18	0.27	0.34	-0.11	0.51	Glucose-6-phosphate 1-dehydrogenase X	Q00612
Gabarapl2	-0.0108	0.10	0.53	-0.29	0.0450	0.16	0.34	-0.19	0.27	Gamma-aminobutyric acid receptor-associated protein-like 2	P60521
Gabbr1	0.0000	-0.11	0.7	0.01	0.86	-0.15	0.62	-0.11	0.76	Gamma-aminobutyric acid type B receptor subunit 1	Q9WV18
Gabbr2	-0.0068	-0.24	0.63	0.24	0.26	0.22	0.29	0.00	0.66	Gamma-aminobutyric acid type B receptor subunit 2	Q80T41
Gabra1	-0.0002	0.14	0.34	-0.06	0.88	0.08	0.44	0.08	0.26	Gamma-aminobutyric acid receptor subunit alpha-1	P62812
Gabrb2	-0.0444	0.59	0.33	-0.49	0.48	0.03	0.44	0.10	0.73	Gamma-aminobutyric acid receptor subunit beta-2	P63137
Gabrg2	-0.0111	0.13	0.077	-0.11	0.2	-0.05	0.77	0.02	0.52	Gamma-aminobutyric acid receptor subunit gamma-2	P22723
Gad1	-0.0228	0.28	0.15	-0.20	0.32	0.09	0.83	0.09	0.93	Glutamate decarboxylase 1	P48318
Gad2	0.0000	0.01	0.99	-0.26	0.057	0.00	0.94	-0.25	0.0300	Glutamate decarboxylase 2	P48320
Gak	-0.0040	-0.09	0.87	0.52	0.0390	-0.15	0.21	0.43	0.08	Cyclin-G-associated kinase	A0A0R4J0F6
Galnt2	0.0110	0.13	0.68	0.77	0.22	-0.18	0.95	0.91	0.12	Polypeptide N-acetylgalactosaminyltransferase 2	Q6PB93
Ganab	-0.0007	-0.14	0.32	0.06	0.67	0.02	0.93	-0.08	0.56	Neutral alpha-glucosidase AB	Q8BHN3
Gap43	0.0002	-0.04	0.83	-0.22	0.5	-0.02	0.51	-0.27	0.39	Neuromodulin	P06837
Gapdh	0.0667	-0.28	0.66	-0.44	0.0010	-0.09	0.88	-0.71	0.0060	Glyceraldehyde-3-phosphate dehydrogenase	A0A0A0MQF6
Gapvd1	-0.0142	1.05	0.67	-0.90	0.82	0.23	0.64	0.15	0.62	GTPase-activating protein and VPS9 domain-containing protein 1	Q6PAR5
Gars1	0.0010	-0.08	0.93	-0.38	0.09	-0.08	0.71	-0.45	0.09	Glycine–tRNA ligase	Q9CZD3
Gart	0.0146	0.23	0.0080	0.07	0.37	0.01	0.69	0.31	0.0020	Trifunctional purine biosynthetic protein adenosine-3	Q64737
Gas7	-0.0001	0.01	0.89	-0.33	0.21	-0.06	0.68	-0.32	0.068	Growth arrest-specific protein 7	F6QB26
Gatad2b	-0.0067	0.21	0.87	-1.90	0.53	-1.75	0.71	-1.69	0.7	Gatad2b protein	A1L3S7
Gatd1	0.0000	-0.13	0.88	0.03	0.72	0.14	0.82	-0.10	0.98	Glutamine amidotransferase-like class 1 domain-containing protein 1	Q8BFQ8
Gatd3a	-0.8435	0.29	0.0290	-0.57	0.0005	-0.24	0.23	-0.28	0.0180	Glutamine amidotransferase-like class 1 domain-containing protein 3A, mitochondrial	Q9D172
Gcdh	-0.0020	0.99	0.15	-0.09	0.94	-0.09	0.97	0.90	0.16	Glutaryl-CoA dehydrogenase, mitochondrial	Q60759
Gcn1	-0.0803	0.53	0.0260	-0.30	0.48	0.21	0.5	0.23	0.48	eIF-2-alpha kinase activator GCN1	E9PVA8

Table S2: Differentially expressed proteins in cerebral cortex *(continued)*

gene	$\pi_{TW/EW} \times \pi_{TF/TW}$	log2FC TW/EW	padj TW/EW	log2FC TF/TW	padj TF/TW	log2FC EF/EW	padj EF/EW	log2FC TF/EW	padj TF/EW	description	accession
Gda	-0.0066	-0.33	0.47	0.25	0.57	-0.36	0.14	-0.09	0.94	Guanine deaminase	Q9R111
Gdap1	-0.1222	1.01	0.068	-0.24	0.37	-1.33	0.41	0.76	0.2	Ganglioside-induced differentiation-associated protein 1	O88741
Gdap1l1	-0.0002	-0.17	0.79	0.10	0.76	-0.06	0.87	-0.06	0.99	Ganglioside-induced differentiation-associated protein 1-like 1	Q3USC7
Gdi1	0.0002	-0.21	0.48	-0.10	0.93	-0.21	0.23	-0.31	0.3	Rab GDP dissociation inhibitor alpha	P50396
Gemin5	-0.0060	0.19	0.38	-0.14	0.29	0.05	0.88	0.05	0.86	Gem-associated protein 5	A2AFQ9
Get3	-0.0002	0.01	0.53	-0.16	0.4	0.24	0.5	-0.16	0.98	ATPase GET3	O54984
Gfod1	0.0754	1.06	0.65	0.93	0.39	0.85	0.62	1.99	0.15	Glucose-fructose oxidoreductase domain-containing protein 1	Q3UHD2
Ggt7	0.1031	-0.30	0.73	-4.55	0.28	0.38	0.75	-4.85	0.25	Glutathione hydrolase 7	Q99JP7
Gigyf2	-1.4396	2.20	0.64	-6.28	0.29	0.25	0.9	-4.08	0.48	GRB10-interacting GYF protein 2	G3UYG6
Gipc1	-0.0140	0.50	0.35	-0.26	0.58	-0.05	0.5	0.24	0.54	PDZ domain-containing protein GIPC1	Q9Z0G0
Git1	-1.9683	0.78	0.0300	-0.81	0.0090	-0.08	0.59	-0.04	0.75	ARF GTPase-activating protein GIT1	Q5F258
Gja1	-0.0022	0.37	0.97	-0.74	0.24	0.10	0.88	-0.37	0.11	Gap junction alpha-1 protein	P23242
Gk	0.0070	0.24	0.26	0.25	0.63	-0.01	0.59	0.49	0.2	Glycerol kinase	Q64516
Glg1	-0.0103	-0.10	0.88	0.69	0.0020	0.33	0.52	0.59	0.0002	Golgi apparatus protein 1 (Fragment)	F8WHM5
Glo1	-0.0312	-0.48	0.0020	0.09	0.54	-0.60	0.0080	-0.39	0.067	Lactoylglutathione lyase	Q9CPU0
Gls	-0.0074	0.09	0.44	-0.23	0.1	-0.23	0.14	-0.14	0.25	Glutaminase kidney isoform, mitochondrial	D3Z7P3
Glud1	-0.0028	-0.02	0.87	0.78	0.0010	0.08	0.95	0.76	0.0020	Glutamate dehydrogenase 1, mitochondrial	P26443
Glul	-0.2474	-0.31	0.17	0.54	0.0120	-0.02	0.36	0.22	0.54	Glutamine synthetase	P15105
Gm49804	0.0000	7.02	0.45	-2.95	1	0.57	0.85	4.07	0.43	Predicted gene, 49804	A0A3B2W864
Gmfb	-0.0155	-0.15	0.23	0.21	0.17	-0.15	0.2	0.05	0.66	Glia maturation factor beta	Q9CQI3
Gmps	0.0000	-0.12	0.97	0.05	0.92	0.04	0.66	-0.07	0.64	GMP synthase [glutamine-hydrolyzing]	Q3THK7
Gna11	-0.0095	-0.30	0.15	0.22	0.67	0.15	0.32	-0.08	0.38	Guanine nucleotide-binding protein subunit alpha-11	P21278
Gna13	0.0035	-0.17	0.67	-0.24	0.32	-0.08	0.77	-0.41	0.17	Guanine nucleotide-binding protein subunit alpha-13	P27601
Gnao1	-0.0011	-0.25	0.83	0.13	0.37	0.33	0.12	-0.12	0.54	Guanine nucleotide-binding protein G(o) subunit alpha	P18872
Gnaq	-0.0002	-0.24	0.65	0.06	0.86	0.23	0.99	-0.18	0.58	Guanine nucleotide-binding protein G(q) subunit alpha	P21279
Gnaz	-0.0183	0.19	0.13	-0.16	0.21	0.27	0.56	0.03	0.83	Guanine nucleotide-binding protein G(z) subunit alpha	O70443
Gnb2	0.0000	-0.35	0.99	0.12	0.58	0.22	0.52	-0.22	0.99	Guanine nucleotide-binding protein G(I)/G(S)/G(T) subunit beta-2	D3YZX3

Table S2: Differentially expressed proteins in cerebral cortex *(continued)*

gene	$\pi_{TW/EW} \times \pi_{TF/TW}$	log2FC TW/EW	padj TW/EW	log2FC TF/TW	padj TF/TW	log2FC EF/EW	padj EF/EW	log2FC TF/EW	padj TF/EW	description	accession
Gnb5	-0.0026	0.13	0.71	-0.27	0.32	0.53	0.74	-0.14	0.65	Guanine nucleotide-binding protein subunit beta-5	P62881
Gng10	0.0000	0.02	1	0.28	0.41	0.30	0.46	0.30	0.14	Guanine nucleotide-binding protein G(I)/G(S)/G(O) subunit gamma-10	Q9CXP8
Gng12	-0.0371	-0.78	0.0001	0.29	0.91	0.31	0.051	-0.49	0.0270	Guanine nucleotide-binding protein subunit gamma (Fragment)	A0A0N4SVT3
Gng13	-0.0332	-0.65	0.0010	0.15	0.77	0.33	0.23	-0.50	0.0120	Guanine nucleotide-binding protein G(I)/G(S)/G(O) subunit gamma-13	Q9JMF3
Gng2	-0.0003	-0.40	0.52	0.11	0.95	0.02	0.82	-0.28	0.5	Guanine nucleotide-binding protein G(I)/G(S)/G(O) subunit gamma-2	P63213
Gng5	-0.3609	-0.83	0.11	0.57	0.16	0.45	0.23	-0.26	0.18	Guanine nucleotide-binding protein G(I)/G(S)/G(O) subunit gamma-5	Q80SZ7
Gnl1	0.0003	0.20	0.26	0.02	0.77	-0.35	0.13	0.22	0.0300	Guanine nucleotide-binding protein-like 1	P36916
Golga2	0.0405	0.17	0.22	0.30	0.062	0.31	0.061	0.47	0.0010	Golgin subfamily A member 2	A0A6I8MX07
Golga3	-0.0483	0.31	0.22	-0.36	0.22	0.35	0.065	-0.05	0.94	Golgin subfamily A member 3	A0A0R4J1H6
GOLGA7B	0.0000	-0.13	0.59	0.02	0.92	0.36	0.094	-0.11	0.67	Golgin subfamily A member 7B	Q9D428
Golgb1	-0.0898	-0.11	0.6	0.92	0.0001	0.49	0.36	0.81	0.0010	Golgi autoantigen, golgin subfamily b, macrogolgin 1	E9PVZ8
Got1	0.2686	-0.44	0.0001	-0.13	0.067	-0.21	0.0028	-0.56	0.0010	Aspartate aminotransferase, cytoplasmic	P05201
Got2	-0.3490	0.14	0.06	-0.51	0.0001	-0.16	0.45	-0.37	0.2	Aspartate aminotransferase, mitochondrial	P05202
Gpc1	-0.0116	0.45	0.23	-0.16	0.56	0.05	0.85	0.28	0.42	Glypican-1	Q9QZF2
Gpc4	-0.0033	0.35	0.4	-0.18	0.74	-0.31	0.61	0.17	0.74	Glypican-4	P51655
Gpd1	0.0000	-0.02	0.88	0.17	0.72	-0.89	0.13	0.16	0.76	Glycerol-3-phosphate dehydrogenase [NAD(+)]	E0CXN5
Gpd1l	0.0064	-0.32	0.17	-0.11	0.58	-0.57	0.0190	-0.43	0.14	Glycerol-3-phosphate dehydrogenase 1-like protein	Q3ULJ0
Gpd2	-0.0002	0.01	0.22	-0.03	0.1	-0.01	0.46	-0.02	0.96	Glycerol-3-phosphate dehydrogenase, mitochondrial	Q64521
Gphn	-0.0590	0.41	0.15	-0.29	0.25	0.01	0.37	0.12	0.21	Gephyrin	Q8BUV3
Gpi	0.0002	-0.34	0.49	-0.03	0.89	-0.32	0.49	-0.37	0.49	Glucose-6-phosphate isomerase	P06745
Gpm6a	-0.9876	1.18	0.17	-1.32	0.15	-1.59	0.47	-0.14	0.87	Neuronal membrane glycoprotein M6-a	P35802
Gpm6b	-0.0003	-0.18	0.65	0.08	0.78	0.00	1	-0.10	0.56	Neuronal membrane glycoprotein M6-b	A2AEG6
Gpr158	-0.0334	-0.20	0.63	0.33	0.0030	0.02	0.89	0.13	0.0430	Probable G-protein coupled receptor 158	Q8C419
Gpr162	-0.0423	0.42	0.17	-0.25	0.3	0.28	0.49	0.18	0.5	Probable G-protein coupled receptor 162	Q3UN16
Gprasp1	-0.9802	1.90	0.41	-2.41	0.28	0.99	0.63	-0.52	0.51	G-protein coupled receptor-associated sorting protein 1	Q5U4C1
Gprc5b	-0.0897	-0.46	0.0200	0.47	0.57	0.20	0.88	0.01	0.38	G protein-coupled receptor family C group 5 member B variant 2	D2DFA9

Table S2: Differentially expressed proteins in cerebral cortex *(continued)*

gene	$\pi_{TW/EW} \times \pi_{TF/TW}$	log2FC TW/EW	padj TW/EW	log2FC TF/TW	padj TF/TW	log2FC EF/EW	padj EF/EW	log2FC TF/EW	padj TF/EW	description	accession
Gprin1	-0.0094	-0.50	0.38	0.30	0.71	0.18	0.65	-0.20	0.26	G protein-regulated inducer of neurite outgrowth 1	Q3UNH4
Gprin3	-0.1925	-0.40	0.29	0.66	0.0440	0.62	0.12	0.26	0.36	G protein-regulated inducer of neurite outgrowth 3	Q8BWS5
Gps1	-0.0532	-0.32	0.063	0.28	0.32	-0.20	0.2	-0.04	0.69	COP9 signalosome complex subunit 1	A0A140LJB7
Gpsm1	-5.5338	0.91	0.3	-7.78	0.0320	-0.84	0.87	-6.87	0.2	G-protein-signaling modulator 1 (Fragment)	F6SX81
Gpx1	0.1571	-0.91	0.25	-1.04	0.53	-0.03	0.92	-1.95	0.0470	Glutathione peroxidase 1	P11352
Grb2	0.0220	-0.24	0.16	-0.26	0.36	-0.62	0.0040	-0.50	0.095	Growth factor receptor-bound protein 2	B1AT92
Grcc10	0.0000	0.03	0.98	-0.28	0.36	0.39	0.22	-0.24	0.33	Protein C10	O35127
Gria1	0.0000	0.00	0.75	0.06	0.89	0.02	0.47	0.06	0.93	Glutamate receptor	F6YNQ1
Gria2	0.0000	-0.16	0.96	0.02	0.25	0.22	0.7	-0.15	0.2	Glutamate receptor	E9QKC0
Gria4	-0.0619	-0.27	0.0070	0.12	0.13	0.29	0.0010	-0.15	0.1	Glutamate receptor	G5E863
Grid2	0.0246	-0.63	0.6	-0.48	0.43	-0.46	0.56	-1.11	0.087	Glutamate receptor ionotropic, delta-2	Q61625
Grin1	-0.0395	0.34	0.95	-1.74	0.0010	-0.19	0.48	-1.41	0.0001	Glutamate receptor	A2AI21
Grin2a	-15.5557	1.47	0.0290	-2.23	0.0008	0.11	0.98	-0.75	0.0170	Glutamate receptor ionotropic, NMDA 2A	P35436
Grin2b	-0.2257	0.27	0.29	-0.96	0.0240	-0.14	0.28	-0.68	0.12	Glutamate receptor	G3X9V4
Grip1	-0.7186	1.35	0.44	-1.94	0.17	-1.16	0.44	-0.59	0.59	Glutamate receptor-interacting protein 1	D3YWY4
Grip2	-17.4458	3.82	0.27	-5.12	0.0270	-0.57	0.65	-1.30	0.22	Glutamate receptor-interacting protein 2	E0CXE0
Gripap1	-0.3263	0.91	0.12	-0.61	0.23	0.37	0.41	0.30	0.47	GRIP1-associated protein 1	A2AEW8
Grk2	0.0000	0.14	0.36	0.00	0.56	0.00	0.59	0.14	0.78	Beta-adrenergic receptor kinase 1	Q99MK8
Grm1	0.0000	-0.40	0.62	0.00	0.8	0.16	0.9	-0.40	0.77	Metabotropic glutamate receptor 1	P97772
Grm2	-0.1767	-0.77	0.0001	0.18	0.48	0.12	0.32	-0.58	0.0001	Metabotropic glutamate receptor 2	Q14BI2
Grm3	-0.0016	-0.39	0.092	0.07	0.88	0.15	0.27	-0.32	0.45	Metabotropic glutamate receptor 3	Q9QYS2
Grm5	-0.0023	-0.37	0.55	0.12	0.63	-0.07	0.76	-0.24	0.78	Metabotropic glutamate receptor 5	Q3UVX5
Grm7	0.0005	-0.05	0.93	-0.39	0.18	0.04	0.97	-0.44	0.0150	Metabotropic glutamate receptor 7	Q68ED2
Grpel1	-0.0001	0.03	0.79	-0.20	0.73	-0.26	0.051	-0.17	0.41	GrpE protein homolog 1, mitochondrial	Q99LP6
Gsk3b	-0.0001	-0.15	0.95	0.21	0.73	-0.05	0.56	0.05	0.74	Glycogen synthase kinase-3 beta	E9QAQ5
Gsn	0.0000	-0.02	0.85	-0.02	0.55	0.32	0.2	-0.04	0.47	Gelsolin	P13020
Gsta4	-0.4967	-0.63	0.0002	0.29	0.19	-0.75	0.0001	-0.35	0.0280	Glutathione S-transferase A4	P24472
Gstm1	-0.0005	0.03	0.85	-0.47	0.34	-0.76	0.38	-0.44	0.43	Glutathione transferase	A2AE89
Gstp1	-0.1279	-0.31	0.082	0.37	0.094	-0.38	0.065	0.06	0.7	Glutathione S-transferase P 1	P19157
Gstz1	0.0001	-0.07	0.61	-0.09	0.81	-0.29	0.53	-0.16	0.52	Maleylacetoacetate isomerase	A0A1Y7VNC1
Gtf2i	0.5874	4.24	0.35	1.07	0.52	5.35	0.084	5.31	0.16	General transcription factor II-I	Q3UHU8
Gtpbp1	-0.0970	0.41	0.11	-0.31	0.16	-0.24	0.21	0.10	0.66	GTP-binding protein 1	O08582
Gucy1a2	0.0012	-0.17	0.3	-0.09	0.71	-0.01	0.42	-0.26	0.29	Guanylate cyclase	F8VQK3
H1-0	-0.4741	0.93	0.39	-1.62	0.17	-0.26	0.65	-0.69	0.73	Histone H1.0	P10922
H1-4	-0.5940	1.03	0.13	-0.79	0.15	-0.03	0.97	0.23	0.87	Histone H1.4	P43274

Table S2: Differentially expressed proteins in cerebral cortex *(continued)*

gene	$\pi_{TW/EW} \times \pi_{TF/TW}$	log2FC TW/EW	padj TW/EW	log2FC TF/TW	padj TF/TW	log2FC EF/EW	padj EF/EW	log2FC TF/EW	padj TF/EW	description	accession
H2ax	-0.1928	1.09	0.45	-1.60	0.48	-0.27	0.87	-0.51	0.93	Histone H2AX	P27661
H2az2	-0.0001	0.05	0.71	-0.27	0.9	0.28	0.61	-0.22	0.87	Histone H2A.V	Q3THW5
H3f3a	-0.2981	0.85	0.33	-1.01	0.19	-0.19	0.68	-0.16	0.9	Histone H3	F8WI35
H4c1	-2.5667	0.71	0.13	-1.36	0.0010	0.12	0.53	-0.65	0.2	Histone H4	P62806
Hacd3	-0.1049	0.60	0.25	-0.54	0.29	0.00	0.63	0.06	0.87	Very-long-chain (3R)-3-hydroxyacyl-CoA dehydratase 3	Q8K2C9
Hadh	-0.0519	-0.20	0.19	0.32	0.075	-0.23	0.3	0.13	0.58	Hydroxyacyl-coenzyme A dehydrogenase, mitochondrial	Q61425
Hadha	0.0024	0.09	0.47	0.08	0.1	0.07	0.24	0.17	0.0001	Trifunctional enzyme subunit alpha, mitochondrial	Q8BMS1
Hadhb	-0.3029	0.35	0.078	-0.55	0.0380	0.02	0.88	-0.20	0.61	Trifunctional enzyme subunit beta, mitochondrial	Q99JY0
Hagh	0.0003	0.02	0.66	0.17	0.27	-0.07	0.44	0.19	0.078	Hydroxyacylglutathione hydrolase, mitochondrial	A0A0R4J052
Hapln1	0.0061	-0.21	0.11	-0.07	0.37	-0.20	0.086	-0.28	0.0020	Hyaluronan and proteoglycan link protein 1	Q9QUP5
Hapln4	0.0004	0.06	0.84	0.26	0.46	0.19	0.65	0.32	0.41	Hyaluronan and proteoglycan link protein 4	Q80WM4
Hars1	0.0004	-0.32	0.68	-0.04	0.66	-0.01	0.73	-0.36	0.39	Histidine–tRNA ligase, cytoplasmic	Q61035
Hba	-2.0843	-0.57	0.0006	0.38	0.0010	-0.56	0.0010	-0.19	0.24	Hemoglobin subunit alpha	P01942
Hbb-bs	0.0003	0.02	0.41	0.17	0.64	-0.14	0.92	0.19	0.16	Beta-globin	A8DUK4
Hcfc1	-0.0002	-0.15	0.97	0.24	0.36	0.16	0.57	0.09	0.4	Host cell factor 1	B1AUX2
Hcn2	-0.0010	0.15	0.6	-0.19	0.69	0.17	0.59	-0.04	0.84	Potassium/sodium hyperpolarization-activated cyclic nucleotide-gated channel 2	O88703
Hdac6	0.0006	0.14	0.69	0.10	0.52	0.21	0.47	0.24	0.5	Histone deacetylase 6 (Fragment)	A0A1B0GX25
Hdgfl3	-5.8693	1.15	0.0180	-1.29	0.0054	-0.20	0.6	-0.14	0.98	Hepatoma-derived growth factor-related protein 3	Q9JMG7
Hdhd3	-0.0524	0.34	0.28	-0.45	0.24	-0.66	0.66	-0.11	0.91	Haloacid dehalogenase-like hydrolase domain-containing protein 3	Q9CYW4
Hdlbp	-0.0579	-0.10	0.52	0.51	0.0001	0.14	0.83	0.42	0.0001	Vigilin	Q8VDJ3
Heatr1	0.0554	1.21	0.44	0.36	0.44	-3.80	0.44	1.57	0.21	HEAT repeat-containing protein 1	G3X9B1
Hectd1	0.0031	-0.10	0.54	-0.19	0.25	0.04	0.95	-0.29	0.12	HECT-type E3 ubiquitin transferase	F8WIE5
Hectd3	0.0116	0.38	0.4	0.24	0.48	0.29	0.42	0.62	0.2	E3 ubiquitin-protein ligase HECTD3	Q3U487
Hectd4	0.0000	0.23	0.19	0.00	0.36	0.12	0.56	0.22	0.76	HECT domain E3 ubiquitin protein ligase 4	E9Q2E4
Hecw1	-0.0029	0.18	0.62	-0.30	0.55	0.16	0.66	-0.12	0.8	E3 ubiquitin-protein ligase HECW1	Q8K4P8
Hecw2	-0.0004	0.02	0.74	-0.20	0.2	0.21	0.37	-0.18	0.33	E3 ubiquitin-protein ligase HECW2	Q6I6G8
Hepacam	0.0004	0.14	0.3	0.08	0.86	-0.25	0.2	0.22	0.11	Hepatocyte cell adhesion molecule	Q640R3

Table S2: Differentially expressed proteins in cerebral cortex *(continued)*

gene	$\pi_{TW/EW} \times \pi_{TF/TW}$	log2FC TW/EW	padj TW/EW	log2FC TF/TW	padj TF/TW	log2FC EF/EW	padj EF/EW	log2FC TF/EW	padj TF/EW	description	accession
Herc1	-1.2933	2.14	0.14	-1.47	0.33	1.52	0.17	0.67	0.46	HECT and RLD domain-containing E3 ubiquitin protein ligase family member 1	E9PZP8
Hexa	-0.1102	-1.14	0.45	0.90	0.49	0.91	0.12	-0.23	0.93	Beta-hexosaminidase subunit alpha	P29416
Hexb	0.0000	-0.07	0.55	0.22	1	0.42	0.24	0.15	0.54	Beta-hexosaminidase subunit beta	P20060
Hibch	-0.0268	0.40	0.59	-0.56	0.3	-0.49	0.86	-0.16	0.99	3-hydroxyisobutyryl-CoA hydrolase, mitochondrial	Q8QZS1
Hid1	-0.0039	-0.20	0.5	0.22	0.51	0.12	0.51	0.01	0.83	Protein HID1	Q8R1F6
Hint2	0.0171	-0.22	0.62	-0.57	0.22	0.05	0.8	-0.78	0.19	Histidine triad nucleotide-binding protein 2, mitochondrial	Q9D0S9
Hip1	-0.0943	-0.26	0.78	0.84	0.0001	0.23	0.18	0.58	0.0003	Huntingtin-interacting protein 1	Q8VD75
Hip1r	0.1112	0.35	0.2	0.63	0.19	0.35	0.22	0.98	0.0270	Huntingtin-interacting protein 1-related protein	Q9JKY5
Hist2h2bb	-1.0808	1.48	0.33	-2.17	0.2	-10.20	0.17	-0.69	0.57	Histone H2B type 2-B	Q64525
Hk1	0.0003	0.02	0.37	0.11	0.54	-0.14	0.0040	0.13	0.052	Hexokinase	G3UVV4
Hmgb1	0.0000	-0.05	1	0.01	0.92	-0.09	0.77	-0.04	0.91	High mobility group protein B1	P63158
Hmgcl	0.0676	0.36	0.063	0.21	0.18	-0.65	0.29	0.57	0.0130	Hydroxymethylglutaryl-CoA lyase, mitochondrial	P38060
Hnrnpa0	-0.0006	0.61	0.91	-0.58	0.91	-0.63	0.19	0.03	0.94	Heterogeneous nuclear ribonucleoprotein A0	Q9CX86
Hnrnpa1	-0.0010	0.21	0.75	-0.26	0.72	-1.04	0.0410	-0.05	0.98	Heterogeneous nuclear ribonucleoprotein A1	P49312
Hnrnpa2b1	-0.0078	0.16	0.66	-0.46	0.26	-0.74	0.0310	-0.30	0.26	Heterogeneous nuclear ribonucleoproteins A2/B1	O88569
Hnrnpa3	-3.1680	2.60	0.3	-2.63	0.13	-0.53	0.4	-0.03	0.8	Heterogeneous nuclear ribonucleoprotein A3	A2AL12
Hnrnpab	0.0000	-0.13	0.96	-0.07	0.59	-0.36	0.7	-0.20	0.98	Heterogeneous nuclear ribonucleoprotein A/B	Q99020
Hnrnpc	-0.0012	0.24	0.74	-0.43	0.82	-1.68	0.0120	-0.19	0.86	Heterogeneous nuclear ribonucleoproteins C1/C2	Q9Z204
Hnrnpd	0.0013	-0.18	0.71	-0.08	0.25	-0.56	0.0440	-0.26	0.093	Heterogeneous nuclear ribonucleoprotein D0 (Fragment)	F6ZV59
Hnrnpdl	0.0003	0.25	0.72	0.06	0.76	-0.19	0.75	0.30	0.38	Heterogeneous nuclear ribonucleoprotein D-like	D3YTQ3
Hnrnpf	-0.0016	0.19	0.79	-0.36	0.59	-0.43	0.089	-0.17	0.52	Heterogeneous nuclear ribonucleoprotein F	Q9Z2X1
Hnrnph1	0.0000	0.00	1	-0.57	0.24	-0.30	0.16	-0.57	0.091	Heterogeneous nuclear ribonucleoprotein H	O35737
Hnrnph3	-0.1600	0.35	0.32	-0.80	0.07	-0.20	0.21	-0.44	0.089	Heterogeneous nuclear ribonucleoprotein H3	D3YWT1
Hnrnpk	0.0000	0.00	0.88	-0.04	0.85	-0.17	0.59	-0.03	0.93	Heterogeneous nuclear ribonucleoprotein K	P61979

Table S2: Differentially expressed proteins in cerebral cortex *(continued)*

gene	$\pi_{TW/EW} \times \pi_{TF/TW}$	log2FC TW/EW	padj TW/EW	log2FC TF/TW	padj TF/TW	log2FC EF/EW	padj EF/EW	log2FC TF/EW	padj TF/EW	description	accession
Hnrnpl	-0.1948	0.67	0.24	-0.63	0.18	-0.51	0.25	0.04	0.96	Heterogeneous nuclear ribonucleoprotein L (Fragment)	G5E924
Hnrnpll	-0.0722	0.55	0.097	-0.30	0.37	0.28	0.44	0.25	0.45	Heterogeneous nuclear ribonucleoprotein L-like	Q921F4
Hnrnpm	-0.0006	0.41	0.95	-1.11	0.88	-1.59	0.0022	-0.70	0.069	Heterogeneous nuclear ribonucleoprotein M	Q9D0E1
Hnrnpu	-0.0042	0.11	0.38	-0.28	0.47	-0.47	0.54	-0.17	0.8	Heterogeneous nuclear ribonucleoprotein U	Q8VEK3
Hnrnpul2	-0.0130	-0.26	0.55	0.40	0.33	-0.02	0.63	0.14	0.55	Heterogeneous nuclear ribonucleoprotein U-like protein 2	Q00PI9
Homer1	-16.0534	1.23	0.0130	-1.73	0.0001	0.10	0.6	-0.50	0.068	Homer protein homolog 1	Q9Z2Y3
Homer2	-2.5984	0.60	0.073	-1.27	0.0010	-0.39	0.5	-0.67	0.13	Homer protein homolog 2	Q9QWW1
Homer3	-0.0009	0.59	0.68	-0.43	0.95	-1.50	0.49	0.16	0.92	Homer protein homolog 3	Q99JP6
Hook3	-0.0431	-0.36	0.58	0.68	0.18	0.18	0.58	0.32	0.39	Protein Hook homolog 3	Q8BUK6
Hprt1	-0.1389	-0.54	0.14	0.30	0.099	-0.29	0.86	-0.24	0.68	Hypoxanthine-guanine phosphoribosyltransferase	P00493
Hsd17b10	-0.0031	0.13	0.43	-0.14	0.34	0.53	0.16	-0.01	0.83	3-hydroxyacyl-CoA dehydrogenase type-2	A2AFQ2
Hsd17b11	-0.0421	0.11	0.73	-2.18	0.052	-0.29	0.69	-2.07	0.093	Estradiol 17-beta-dehydrogenase 11	Q9EQ06
Hsd17b4	0.0205	0.19	0.0290	0.15	0.34	0.43	0.16	0.34	0.16	Peroxisomal multifunctional enzyme type 2	P51660
Hsd17b8	-0.0010	0.10	0.95	-0.73	0.26	-0.78	0.17	-0.62	0.29	Estradiol 17-beta-dehydrogenase 8	P50171
Hsdl1	-0.0002	0.06	0.83	-0.12	0.48	0.25	0.2	-0.06	0.58	Inactive hydroxysteroid dehydrogenase-like protein 1	A0A1D5RLG0
Hsdl2	-0.2519	0.37	0.0310	-0.31	0.0350	0.40	0.77	0.06	0.63	Hydroxysteroid dehydrogenase-like protein 2	Q2TPA8
Hsp90aa1	0.0010	0.05	0.39	0.08	0.25	0.12	0.15	0.13	0.0200	Heat shock protein HSP 90-alpha	P07901
Hsp90ab1	0.0006	0.08	0.35	0.03	0.26	0.06	0.76	0.10	0.98	Heat shock protein HSP 90-beta	P11499
Hsp90b1	-0.0236	-0.19	0.0180	0.14	0.31	-0.08	0.17	-0.05	0.11	Endoplasmin	P08113
Hspa12a	-0.0106	0.02	0.56	-0.70	0.0010	0.06	0.77	-0.69	0.0230	Heat shock 70 kDa protein 12A	Q8K0U4
Hspa12b	-0.9831	0.53	0.35	-1.15	0.0003	-0.04	0.91	-0.63	0.47	Heat shock 70 kDa protein 12B	Q9CZJ2
Hspa1b	-0.0070	0.26	0.0250	-0.04	0.38	0.10	0.96	0.22	0.28	Heat shock 70 kDa protein 1B	P17879
Hspa4	-0.0027	-0.18	0.44	0.13	0.48	0.03	0.66	-0.05	1	Heat shock 70 kDa protein 4	Q3U2G2
Hspa4l	-0.0003	-0.07	0.54	0.05	0.5	0.09	0.62	-0.03	0.96	Heat shock 70 kDa protein 4L	P48722
Hspa5	0.0352	0.13	0.25	0.45	0.1	0.06	0.95	0.58	0.0008	Endoplasmic reticulum chaperone BiP	P20029
Hspa8	0.0005	0.07	0.33	0.08	0.63	-0.04	0.72	0.16	0.15	Heat shock cognate 71 kDa protein	P63017
Hspa9	0.0051	-0.12	0.38	-0.14	0.19	-0.02	0.68	-0.25	0.0290	Stress-70 protein, mitochondrial	P38647
Hspb1	-0.0139	0.08	0.84	-1.77	0.051	-0.90	0.56	-1.69	0.0280	Heat shock protein beta-1	D3YZ06
Hspd1	-0.4295	0.20	0.0300	-0.47	0.0010	-0.12	0.33	-0.27	0.32	60 kDa heat shock protein, mitochondrial	P63038
Hsph1	-0.0001	-0.02	0.62	0.13	0.57	0.00	0.67	0.10	0.31	Heat shock protein 105 kDa	E9Q0U7
Htra2	0.7001	1.00	0.45	1.30	0.0280	1.60	0.0490	2.31	0.0280	Serine protease HTRA2, mitochondrial	Q9JIY5

Table S2: Differentially expressed proteins in cerebral cortex *(continued)*

gene	$\pi_{TW/EW} \times \pi_{TF/TW}$	log2FC TW/EW	padj TW/EW	log2FC TF/TW	padj TF/TW	log2FC EF/EW	padj EF/EW	log2FC TF/EW	padj TF/EW	description	accession
Htt	-0.0019	0.10	0.91	-0.91	0.31	-0.80	0.37	-0.81	0.31	Huntingtin	G3X9H5
Huwe1	0.0004	-0.11	0.93	-0.26	0.34	0.00	0.9	-0.37	0.37	HECT-type E3 ubiquitin transferase	A2AFQ0
Hyou1	-0.0035	-0.13	0.25	0.11	0.39	-0.01	0.71	-0.02	0.7	Hypoxia up-regulated protein 1	Q9JKR6
Iap	0.0000	0.28	0.96	-0.68	0.98	0.09	0.79	-0.40	0.16	IgE-binding protein	P03975
Iars1	-0.3731	1.05	0.0160	-0.31	0.23	0.52	0.29	0.73	0.34	Isoleucine–tRNA ligase, cytoplasmic	Q8BU30
Iars2	-0.0849	0.38	0.57	-1.52	0.25	-1.03	0.57	-1.13	0.49	Isoleucine–tRNA ligase, mitochondrial	Q8BIJ6
Icam5	-0.0027	-0.21	0.31	0.05	0.31	0.18	1	-0.16	0.1	Intercellular adhesion molecule 5	Q60625
Idh1	0.8646	-0.43	0.0470	-1.04	0.0350	-0.53	0.16	-1.47	0.0110	Isocitrate dehydrogenase [NADP] cytoplasmic	O88844
Idh2	-0.0021	0.09	0.68	-0.47	0.5	-0.70	0.18	-0.37	0.81	Isocitrate dehydrogenase [NADP], mitochondrial	P54071
Idh3a	-0.0079	0.06	0.47	-0.29	0.0420	-0.13	0.33	-0.23	0.31	Isocitrate dehydrogenase [NAD] subunit, mitochondrial	A0A1L1STE6
Idh3b	-3.5528	0.64	0.0008	-0.50	0.0003	0.08	0.89	0.14	0.6	Isocitrate dehydrogenase [NAD] subunit, mitochondrial	Q91VA7
Idh3g	-0.0049	0.08	0.69	-0.29	0.0480	-0.08	0.9	-0.22	0.1	Isocitrate dehydrogenase [NAD] subunit gamma 1, mitochondrial	P70404
Igdcc4	-0.1690	-0.89	0.098	0.36	0.3	-1.74	0.15	-0.52	0.44	Immunoglobulin superfamily DCC subclass member 4	Q9EQS9
Ighm	0.0031	-0.56	0.76	-0.11	0.38	-0.35	0.54	-0.68	0.2	Immunoglobulin heavy constant mu (Fragment)	A0A075B5P6
Iglon5	3.9016	-4.52	0.44	-4.63	0.3	-1.68	0.056	-9.15	0.0280	IgLON family member 5	Q8HW98
Igsf21	-0.4110	0.58	0.1	-0.86	0.15	0.56	0.41	-0.28	0.98	Immunoglobulin superfamily member 21	Q7TNR6
Igsf8	-0.0020	-0.16	0.79	0.31	0.4	0.28	0.15	0.15	0.28	Immunoglobulin superfamily member 8	Q8R366
Igsf9b	-0.7633	0.87	0.16	-1.15	0.11	-0.58	0.67	-0.28	0.36	Protein turtle homolog B	A0A1L1SR84
Ik	-0.5857	1.31	0.31	-1.46	0.25	0.23	0.48	-0.15	0.47	Protein Red	Q9Z1M8
Il1rapl1	-1.1915	3.29	0.74	-4.60	0.25	-1.24	0.57	-1.31	0.52	Interleukin-1 receptor accessory protein-like 1	B1ASU0
Ildr2	-0.0001	0.32	0.53	-0.27	0.99	0.18	0.35	0.06	0.76	Immunoglobulin-like domain-containing receptor 2	A0A0A6YVY9
Ilf2	-0.0043	-0.20	0.73	0.44	0.44	-4.17	0.059	0.24	0.61	Interleukin enhancer-binding factor 2	Q9CXY6
Ilf3	-0.0301	0.50	0.65	-0.52	0.24	-0.55	0.083	-0.02	0.32	Interleukin enhancer-binding factor 3	A0A1L1STE4
Ilk	-1.5951	0.42	0.12	-1.72	0.0040	-0.97	0.072	-1.30	0.0470	Integrin-linked protein kinase (Fragment)	D3YZA5
Immt	-0.0005	0.51	0.5	-0.13	0.94	-0.04	0.7	0.38	0.48	MICOS complex subunit Mic60	Q8CAQ8
Impa1	0.0000	-0.26	0.83	0.12	0.97	-0.54	0.35	-0.14	0.83	Inositol monophosphatase 1	O55023
Ina	-48.6318	1.80	0.0060	-3.04	0.0001	-0.50	0.09	-1.24	0.0001	Alpha-internexin	P46660
Inpp4a	0.0008	0.20	0.1	0.06	0.86	-0.04	0.7	0.26	0.36	Phosphatidylinositol-3,4-bisphosphate 4-phosphatase	D3YUL8
Inpp5a	0.0000	-0.04	0.96	0.47	1	0.31	0.51	0.43	0.49	Inositol-polyphosphate 5-phosphatase	Q3TZT4

Table S2: Differentially expressed proteins in cerebral cortex *(continued)*

gene	$\pi_{TW/EW}\times$ $\pi_{TF/TW}$	log2FC TW/EW	padj TW/EW	log2FC TF/TW	padj TF/TW	log2FC EF/EW	padj EF/EW	log2FC TF/EW	padj TF/EW	description	accession
Inpp5j	0.0004	0.15	0.43	0.07	0.8	-0.21	0.26	0.22	0.66	Phosphatidylinositol 4,5-bisphosphate 5-phosphatase A	P59644
Ipo7	0.0138	-0.34	0.0150	-0.07	0.48	-0.03	0.46	-0.41	0.18	Importin-7	Q9EPL8
Iqgap2	-15.3717	4.24	0.38	-9.00	0.11	-4.68	0.17	-4.76	0.077	Ras GTPase-activating-like protein IQGAP2	Q3UQ44
Iqsec1	-77.0556	4.09	0.1	-6.28	0.0010	0.54	0.99	-2.19	0.092	IQ motif and SEC7 domain-containing protein 1	A0A1D5RM83
Iqsec2	-9.7040	1.73	0.13	-2.64	0.0040	0.19	0.9	-0.91	0.066	ARF6 guanine nucleotide exchange factor IQArfGEF	A4GZ26
Iqsec3	-0.0760	0.72	0.74	-1.38	0.26	-0.09	0.81	-0.65	0.28	IQ motif and SEC7 domain-containing protein 3	Q3TES0
Irgq	-0.2074	-0.84	0.05	0.28	0.21	-0.23	0.8	-0.56	0.18	Immunity-related GTPase family Q protein	Q8VIM9
Isca2	-1.8343	1.50	0.36	-2.45	0.075	1.22	0.23	-0.94	0.32	Iron-sulfur cluster assembly 2 homolog, mitochondrial (Fragment)	A0A1Y7VK19
Ist1	-0.0035	0.11	0.26	-0.09	0.25	0.05	0.54	0.02	0.91	IST1 homolog	Q9CX00
Isyna1	0.0000	-0.02	0.99	-0.30	0.69	-0.03	0.89	-0.32	0.59	Inositol-3-phosphate synthase 1	Q9JHU9
Itch	-0.1890	0.64	0.26	-0.70	0.19	0.05	0.75	-0.06	0.78	E3 ubiquitin-protein ligase Itchy	Q8C863
Itpka	-0.5374	1.17	0.23	-1.23	0.26	0.98	0.37	-0.06	0.95	Inositol-trisphosphate 3-kinase A	Q8R071
Itpr1	0.0039	0.24	0.68	0.36	0.54	-0.39	0.83	0.60	0.34	Inositol 1,4,5-trisphosphate receptor type 1	P11881
Itsn1	0.0006	0.24	0.0008	0.20	0.99	0.30	0.0180	0.44	0.0001	Intersectin-1	E9Q0N0
Ivd	0.1457	-0.58	0.051	-0.45	0.37	-0.58	0.11	-1.03	0.0010	Isovaleryl-CoA dehydrogenase, mitochondrial	Q9JHI5
Jak1	-9.9356	1.89	0.093	-2.88	0.0170	-1.14	0.42	-1.00	0.45	Tyrosine-protein kinase	B1ASP2
Jakmip2	-0.0088	0.43	0.61	-0.43	0.6	0.07	0.94	0.00	1	Janus kinase and microtubule-interacting protein 2	D3YXK0
Jam3	-0.0371	-0.36	0.83	1.49	0.14	0.36	0.87	1.13	0.4	Junctional adhesion molecule C	Q9D8B7
Jph4	-0.0913	0.69	0.64	-1.07	0.23	-0.82	0.0280	-0.37	0.36	Junctophilin-4	Q80WT0
Jup	0.0023	-0.26	0.92	-0.35	0.2	-0.15	0.93	-0.61	0.21	Junction plakoglobin	Q02257
Kars	-0.0800	-0.11	0.35	0.42	0.0002	0.07	0.41	0.30	0.0010	Lysine–tRNA ligase	Q8R2P8
Kbtbd11	-0.0002	-0.21	0.45	0.02	0.7	0.18	0.8	-0.19	0.19	Kelch repeat and BTB domain-containing protein 11	Q8BNW9
Kcna1	0.0375	1.30	0.41	0.57	0.74	0.46	0.84	1.87	0.19	Potassium voltage-gated channel subfamily A member 1	P16388
Kcnab2	-0.1138	0.28	0.13	-0.38	0.062	-0.36	0.17	-0.09	0.6	K(+) channel subunit beta-2	A0A571BGH0
Kcnb1	-13.5061	1.54	0.0450	-2.83	0.0050	1.02	0.16	-1.29	0.47	Potassium voltage-gated channel subfamily B member 1	Q03717
Kcnd2	-0.0776	0.46	0.28	-0.45	0.21	1.05	0.25	0.00	0.91	Potassium voltage-gated channel subfamily D member 2	Q9Z0V2

Table S2: Differentially expressed proteins in cerebral cortex *(continued)*

gene	$\pi_{TW/EW} \times \pi_{TF/TW}$	log2FC TW/EW	padj TW/EW	log2FC TF/TW	padj TF/TW	log2FC EF/EW	padj EF/EW	log2FC TF/EW	padj TF/EW	description	accession
Kcnip4	-0.0008	-0.12	0.92	0.61	0.49	1.98	0.0020	0.49	0.19	Kv channel-interacting protein 4	Q3UFC0
Kcnq2	-0.0001	-0.37	0.94	0.39	0.92	0.33	0.79	0.02	0.99	Potassium voltage-gated channel subfamily KQT member 2	A0A0G2JFQ2
Kcnq3	-70.5264	6.66	0.093	-7.17	0.0370	1.70	0.4	-0.52	0.82	Potassium voltage-gated channel subfamily KQT member 3	Q8K3F6
Kctd12	-0.0413	-0.34	0.0140	0.14	0.34	0.15	0.16	-0.20	0.12	BTB/POZ domain-containing protein KCTD12	A0A0R4J2B2
Kctd16	-0.0577	0.39	0.19	-0.53	0.41	0.16	0.81	-0.14	0.85	BTB/POZ domain-containing protein KCTD16	Q5DTY9
Kctd8	0.0023	-0.13	0.77	-0.72	0.61	0.37	0.76	-0.85	0.28	BTB/POZ domain-containing protein KCTD8	Q50H33
Khdrbs1	-0.1279	0.75	0.18	-0.56	0.39	-0.17	0.64	0.19	0.38	KH domain-containing, RNA-binding, signal transduction-associated protein 1	Q60749
Khdrbs3	-0.2200	0.63	0.19	-0.65	0.18	-0.12	0.72	-0.02	0.98	KH domain-containing, RNA-binding, signal transduction-associated protein 3	Q9R226
Khsrp	-0.0835	-0.46	0.26	0.43	0.19	-0.46	0.081	-0.03	0.97	Far upstream element-binding protein 2	A0A3B2WCD8
Kiaa1109	0.0180	-0.13	0.56	-0.94	0.26	-0.67	0.12	-1.07	0.13	Transmembrane protein KIAA1109	A2AAE1
Kif1a	-0.0035	-0.08	0.67	0.34	0.18	-0.07	0.97	0.26	0.27	Kinesin-like protein KIF1A	A0A087WQE8
Kif21a	-6.3559	2.07	0.11	-2.20	0.0350	0.97	0.24	-0.13	0.78	Kinesin-like protein KIF21A	F8WGN6
Kif21b	0.0043	-3.73	0.82	-0.24	0.88	-3.75	0.47	-3.98	0.51	Kinesin-like protein KIF21B	E9Q0A4
Kif2a	-2.3818	0.71	0.29	-1.56	0.0001	-0.37	0.095	-0.85	0.0007	Kinesin-like protein	E0CZ72
Kif3a	-0.0020	0.13	0.49	-0.14	0.45	-0.22	0.64	-0.01	0.98	Kinesin-like protein	B1AQZ2
Kif5c	-1.0853	1.02	0.0010	-0.37	0.11	0.16	0.63	0.65	0.0210	Kinesin heavy chain isoform 5C	P28738
Klc1	-0.1041	0.53	0.17	-0.40	0.23	0.07	0.99	0.13	0.94	Kinesin light chain	Q7TNF4
Klc2	0.0059	0.19	0.18	0.06	0.2	-0.50	0.08	0.25	0.82	Kinesin light chain	D3YXZ3
Kpnb1	-0.0439	-0.35	0.0020	0.12	0.41	-0.11	0.23	-0.23	0.0120	Importin subunit beta-1	P70168
Kras	0.0011	-0.31	0.98	-0.28	0.0380	0.07	0.5	-0.59	0.98	GTPase KRas	P32883
Ksr1	-0.1664	-0.63	0.11	0.37	0.18	0.26	0.57	-0.25	0.41	Kinase suppressor of Ras 1	Q61097
Ktn1	0.0004	-0.16	0.87	-0.23	0.68	-0.13	0.6	-0.39	0.69	Kinectin	Q61595
L1cam	-0.0001	0.08	0.31	-0.08	0.93	0.48	0.17	0.00	0.34	Neural cell adhesion molecule L1	A2AFG7
Lactb	-1.0841	0.52	0.0006	-0.47	0.0420	0.40	0.0020	0.05	1	Serine beta-lactamase-like protein LACTB, mitochondrial	Q9EP89
Lamb2	0.0045	-0.18	0.83	-0.51	0.25	0.25	0.57	-0.69	0.49	Laminin subunit beta-2	Q61292
Lamc1	-0.8249	0.72	0.75	-9.01	0.096	1.19	0.35	-8.29	0.23	Laminin subunit gamma-1	P02468
Lamtor1	-0.0118	0.55	0.83	-1.09	0.57	-0.87	0.9	-0.54	0.97	Ragulator complex protein LAMTOR1	Q9CQ22
Lancl1	-0.0315	-0.08	0.69	0.61	0.0001	0.33	0.0020	0.53	0.0002	Glutathione S-transferase LANCL1	O89112
Lancl2	0.0000	-0.03	0.75	0.11	0.8	0.44	0.065	0.08	0.71	LanC-like protein 2 (Fragment)	F6RJV6
Lap3	-0.0002	0.11	0.75	-0.19	0.81	0.15	0.64	-0.08	1	Cytosol aminopeptidase	Q9CPY7
Larp4b	-0.6215	-0.80	0.079	0.62	0.073	0.16	0.75	-0.18	0.5	La-related protein 4B	Q6A0A2

Table S2: Differentially expressed proteins in cerebral cortex *(continued)*

gene	$\pi_{TW/EW}\times \pi_{TF/TW}$	log2FC TW/EW	padj TW/EW	log2FC TF/TW	padj TF/TW	log2FC EF/EW	padj EF/EW	log2FC TF/EW	padj TF/EW	description	accession
Larp6	0.0000	0.83	0.46	-0.14	1	-0.37	0.98	0.68	0.91	La-related protein 6	Q8BN59
Lars1	0.0000	-0.01	0.94	0.03	0.83	-0.18	0.34	0.02	0.89	Leucine–tRNA ligase, cytoplasmic	Q8BMJ2
Lars2	0.0376	0.31	0.42	0.49	0.22	-0.33	0.38	0.80	0.056	Probable leucine–tRNA ligase, mitochondrial	Q8VDC0
Lasp1	-2.3290	0.60	0.057	-1.04	0.0010	0.04	0.93	-0.44	0.22	LIM and SH3 domain protein 1	Q61792
Ldha	-0.0058	-0.52	0.0017	0.05	0.83	-0.08	0.14	-0.47	0.0001	L-lactate dehydrogenase	A0A1B0GSX0
Ldhb	-0.0451	-0.41	0.0003	0.06	0.31	-0.20	0.19	-0.35	0.0180	L-lactate dehydrogenase	A0A6I8MX27
Lemd3	-0.0005	-0.13	0.88	0.43	0.68	0.37	0.51	0.31	0.6	Inner nuclear membrane protein Man1	D3YU56
Letm1	0.0003	0.54	0.2	0.19	0.99	-0.02	0.77	0.73	0.07	Mitochondrial proton/calcium exchanger protein	Q9Z2I0
Letmd1	-0.3994	0.46	0.14	-0.89	0.072	0.00	1	-0.43	0.41	LETM1 domain-containing protein 1	Q924L1
Lgals1	0.0885	0.80	0.11	0.26	0.36	-0.30	0.9	1.06	0.0016	Galectin-1	P16045
Lgals8	0.0000	0.02	0.83	-0.06	0.41	0.27	0.14	-0.04	0.53	Galectin	A8DIL0
Lgi1	0.0011	0.21	0.22	0.01	0.17	0.18	0.32	0.22	0.72	Leucine-rich glioma-inactivated protein 1	A0A0G2JGE8
Lgi2	-0.0021	0.13	0.62	-0.20	0.41	0.30	0.26	-0.07	0.67	Leucine-rich repeat LGI family member 2	Q8K4Z0
Lgi3	-0.0867	0.59	0.52	-0.91	0.27	0.53	0.43	-0.32	0.56	Leucine-rich repeat LGI family member 3	Q8K406
Lima1	-2.1769	4.72	0.13	-3.23	0.69	5.62	0.088	1.49	0.26	LIM domain and actin-binding protein 1	Q9ERG0
Lin7a	-0.0043	-0.20	0.86	0.44	0.18	0.06	0.98	0.24	0.37	Protein lin-7 homolog A	Q8JZS0
Lin7b	-0.0088	0.08	0.38	-0.21	0.057	0.08	0.97	-0.13	0.6	Protein lin-7 homolog B	O88951
Lingo1	-0.0909	-0.31	0.51	0.70	0.0370	-0.02	0.88	0.39	0.27	Leucine-rich repeat and immunoglobulin-like domain-containing nogo receptor-interacting protein 1	A9DA50
Llgl1	-0.0068	-0.29	0.45	0.27	0.56	-0.39	0.41	-0.02	0.75	Lethal(2) giant larvae protein homolog 1	A0A0R4J0S4
Lman1	0.0000	0.02	1	0.11	0.56	0.16	0.55	0.13	0.66	Protein ERGIC-53	Q9D0F3
Lmna	-0.0425	0.14	0.8	-1.22	0.0027	-0.21	0.51	-1.08	0.0018	Prelamin-A/C	P48678
Lmnb1	-0.1343	0.34	0.67	-1.37	0.0220	-0.36	0.34	-1.02	0.0064	Lamin-B1	P14733
Lmnb2	-3.6479	1.01	0.33	-3.26	0.0050	0.41	0.42	-2.24	0.0330	Lamin-B2	A0A0R4J0Q5
Lmo7	-0.1402	-0.79	0.14	0.42	0.32	0.31	0.59	-0.37	0.17	LIM domain only 7	E9PYF4
Lmtk2	0.0002	-0.20	0.49	-0.03	0.81	0.31	0.18	-0.24	0.52	Serine/threonine-protein kinase LMTK2	Q3TYD6
Lmtk3	-3.5248	1.24	0.15	-2.13	0.0240	0.48	0.4	-0.89	0.41	Serine/threonine-protein kinase LMTK3	A0A1B0GSR5
Lonp1	-0.0002	-0.04	0.95	0.51	0.46	-0.67	0.89	0.47	0.28	Lon protease homolog, mitochondrial	Q8CGK3
Lrfn4	-0.0058	2.23	0.12	-0.32	0.98	1.47	0.44	1.91	0.45	Leucine-rich repeat and fibronectin type-III domain-containing protein 4	Q80XU8
Lrfn5	-17.1799	5.92	0.0280	-4.57	0.39	3.07	0.26	1.34	0.76	Leucine-rich repeat and fibronectin type-III domain-containing protein 5	Q8BXA0
Lrp1	-0.0019	-0.02	0.48	0.44	0.21	0.15	0.4	0.41	0.068	Prolow-density lipoprotein receptor-related protein 1	A0A0R4J0I9
Lrp1b	-5.4040	4.78	0.08	-2.59	0.4	6.29	0.074	2.18	0.32	Low-density lipoprotein receptor-related protein 1B	A2API5

Table S2: Differentially expressed proteins in cerebral cortex *(continued)*

gene	$\pi_{TW/EW} \times \pi_{TF/TW}$	log2FC TW/EW	padj TW/EW	log2FC TF/TW	padj TF/TW	log2FC EF/EW	padj EF/EW	log2FC TF/EW	padj TF/EW	description	accession
Lrpap1	0.0449	0.08	0.65	0.75	0.0001	0.13	0.94	0.83	0.0006	Alpha-2-macroglobulin receptor-associated protein	P55302
Lrpprc	-0.0028	0.14	0.39	-0.11	0.36	-0.17	0.47	0.03	0.97	Leucine-rich PPR motif-containing protein, mitochondrial	Q6PB66
Lrrc40	-0.0001	0.04	0.79	-0.07	0.63	-0.25	0.39	-0.04	0.85	Leucine-rich repeat-containing protein 40	Q9CRC8
Lrrc47	0.0004	0.12	0.39	0.23	0.92	-0.33	0.42	0.35	0.41	Leucine-rich repeat-containing protein 47	E9PV22
Lrrc4b	0.0000	0.06	0.88	-0.10	0.84	-0.53	0.25	-0.04	0.81	Leucine-rich repeat-containing protein 4B	P0C192
Lrrc4c	-0.0064	0.46	0.36	-0.11	0.52	0.43	0.4	0.35	0.56	Leucine-rich repeat-containing protein 4C	Q8C031
Lrrc57	-0.1721	-0.48	0.0004	0.15	0.2	0.03	0.54	-0.32	0.0010	Leucine rich repeat containing 57	Q8JZX5
Lrrc59	-2.6859	0.82	0.0001	-0.38	0.0070	0.24	0.8	0.45	0.069	Leucine-rich repeat-containing protein 59	Q922Q8
Lrrc7	-5.5127	1.44	0.24	-2.54	0.0037	-0.76	0.0410	-1.10	0.0005	Leucine-rich repeat-containing protein 7 (Fragment)	A0A0G2JFZ5
Lrrc73	-0.6074	0.32	0.33	-1.41	0.0016	-0.17	0.4	-1.09	0.0005	Gm88 protein	B2RWC4
Lrrc8a	-0.5715	0.70	0.093	-0.70	0.074	-0.24	0.97	0.00	0.86	Volume-regulated anion channel subunit LRRC8A	Q80WG5
Lrrc8c	-0.0014	0.23	0.36	-0.09	0.71	-0.15	0.42	0.13	0.47	Volume-regulated anion channel subunit LRRC8C	Q8R502
Lrrc8d	0.0004	0.06	0.7	0.15	0.56	0.33	0.22	0.22	0.35	Volume-regulated anion channel subunit LRRC8D	Q8BGR2
Lrriq1	-0.0027	0.28	0.0270	-0.05	0.75	0.00	0.98	0.23	0.19	Leucine-rich repeat and IQ domain-containing protein 1	Q0P5X1
Lrrtm1	-16.3379	1.97	0.0230	-2.20	0.0050	-0.11	0.57	-0.23	0.25	Leucine-rich repeat transmembrane neuronal protein 1	Q8K377
Lrrtm4	0.0067	-0.33	0.77	-0.58	0.49	-0.66	0.19	-0.90	0.05	Leucine-rich repeat transmembrane neuronal protein 4	A0A0R4J1T4
Lsamp	-0.0079	0.23	0.93	-0.81	0.0460	-0.24	0.15	-0.58	0.0020	Limbic system-associated membrane protein	Q8BLK3
Lsm12	0.0003	-0.02	0.72	-0.26	0.41	0.57	0.25	-0.28	0.66	Protein LSM12 homolog	Q9D0R8
Lsm14b	0.0000	0.07	0.64	0.00	1	0.06	0.31	0.07	0.53	Protein LSM14 homolog B	Q8BM41
Lsm3	-0.1282	-0.52	0.18	0.55	0.25	-0.55	0.33	0.04	0.49	U6 snRNA-associated Sm-like protein LSm3	P62311
Lsm6	-0.0029	-0.20	0.97	0.50	0.0070	0.01	0.91	0.30	0.35	U6 snRNA-associated Sm-like protein LSm6	P62313
Lsm7	-0.1611	-0.41	0.19	0.54	0.098	-0.33	0.22	0.14	0.67	U6 snRNA-associated Sm-like protein LSm7	A0A1W2P6N1
Lum	0.0101	0.96	0.62	0.19	0.54	1.47	0.061	1.15	0.27	Lumican	P51885
Luzp1	-0.0372	0.50	0.15	-0.24	0.42	0.17	0.97	0.26	0.35	Leucine zipper protein 1	Q8R4U7
Ly6h	0.0000	-0.25	0.13	0.01	0.98	-0.06	0.47	-0.24	0.078	Lymphocyte antigen 6 complex, locus H	Q8K356
Lypla1	-0.0067	0.18	0.19	-0.14	0.43	0.12	0.42	0.05	0.66	Acyl-protein thioesterase 1	D3Z111

Table S2: Differentially expressed proteins in cerebral cortex *(continued)*

gene	$\pi_{TW/EW} \times \pi_{TF/TW}$	log2FC TW/EW	padj TW/EW	log2FC TF/TW	padj TF/TW	log2FC EF/EW	padj EF/EW	log2FC TF/EW	padj TF/EW	description	accession
Lysmd1	0.0001	0.09	0.86	0.16	0.88	-0.76	0.44	0.25	0.91	LysM and putative peptidoglycan-binding domain-containing protein 1	Q9D0E3
Lyz1	-2.1148	-2.04	0.0280	0.53	0.055	-0.91	0.29	-1.51	0.0280	Lysozyme C-1	P17897
Lzts1	-28.6424	2.21	0.0320	-2.89	0.0010	-0.07	0.88	-0.68	0.12	Leucine zipper putative tumor suppressor 1	P60853
Lzts3	-0.0141	0.44	0.95	-2.12	0.21	-0.06	0.97	-1.68	0.23	Leucine zipper putative tumor suppressor 3	A2AHG0
Macroh2a1	-0.5292	1.01	0.34	-1.60	0.2	-0.11	0.94	-0.59	0.53	Core histone macro-H2A.1	Q9QZQ8
Macroh2a2	-4.7109	0.88	0.0290	-1.38	0.0030	-0.13	0.75	-0.49	0.17	Core histone macro-H2A.2	Q8CCK0
Madd	-0.0037	0.13	0.33	-0.15	0.4	-0.05	0.61	-0.02	0.91	MAP kinase-activating death domain protein	A0A0R4J1F4
Mag	-0.0001	-0.08	0.6	0.05	0.82	0.19	0.58	-0.04	0.83	Myelin-associated glycoprotein	P20917
Magi2	-1.7365	0.43	0.44	-9.27	0.06	-1.09	0.39	-8.84	0.055	Membrane-associated guanylate kinase, WW and PDZ domain-containing protein 2	A0A0G2JE00
Magi3	-0.1430	0.82	0.58	-1.41	0.3	-1.37	0.32	-0.59	0.55	Membrane-associated guanylate kinase inverted 3	G5E8T6
Maip1	0.1683	-3.97	0.25	-0.93	0.84	-3.94	0.8	-4.90	0.24	m-AAA protease-interacting protein 1, mitochondrial	Q8BHE8
Man2b1	0.0015	0.46	0.75	0.27	0.8	0.69	0.5	0.73	0.62	Lysosomal alpha-mannosidase	O09159
Manf	0.0116	-0.14	0.56	-0.53	0.24	0.36	0.17	-0.67	0.11	Mesencephalic astrocyte-derived neurotrophic factor	Q3TMX5
Maoa	-0.9085	0.85	0.0270	-0.74	0.12	0.67	0.16	0.11	0.47	Amine oxidase [flavin-containing] A	Q64133
Maob	0.0003	0.05	0.76	0.15	0.41	0.11	0.76	0.21	0.14	Amine oxidase [flavin-containing] B	Q8BW75
Map1a	-0.3692	0.32	0.0005	-0.19	0.0140	0.03	0.0180	0.13	0.0220	Microtubule-associated protein 1A	A2ARP8
Map1b	-0.4224	0.35	0.1	-0.56	0.0070	-0.11	0.49	-0.21	0.28	Microtubule-associated protein 1B	P14873
Map1lc3b	-0.0002	-0.03	0.95	0.49	0.23	0.54	0.11	0.46	0.22	Microtubule-associated proteins 1A/1B light chain 3B	Q9CQV6
Map1s	-0.1386	0.52	0.11	-0.61	0.35	0.12	0.13	-0.09	0.87	Microtubule-associated protein 1S	Q8C052
Map2	-0.0875	0.42	0.0280	-0.14	0.11	0.01	0.63	0.28	0.62	Microtubule-associated protein	A0A668KLC6
Map2k1	0.0008	-0.06	0.28	-0.06	0.38	-0.13	0.0390	-0.12	0.082	Dual specificity mitogen-activated protein kinase kinase 1	P31938
Map2k4	0.0000	-0.04	0.95	-0.07	0.91	-0.26	0.68	-0.11	0.87	Dual specificity mitogen-activated protein kinase kinase 4	P47809
Map2k6	-1.1151	0.78	0.28	-2.04	0.054	-0.29	0.69	-1.26	0.11	Dual specificity mitogen-activated protein kinase kinase 6	P70236
Map4	-0.0006	0.11	0.62	-0.12	0.6	-0.11	0.67	0.00	1	Microtubule-associated protein	A0A0G2JDN7
Map4k3	0.0000	0.17	0.95	-0.07	0.97	0.18	0.62	0.10	0.88	Mitogen-activated protein kinase kinase kinase kinase 3	A0A3Q4EGQ9

Table S2: Differentially expressed proteins in cerebral cortex *(continued)*

gene	$\pi_{TW/EW} \times \pi_{TF/TW}$	log2FC TW/EW	padj TW/EW	log2FC TF/TW	padj TF/TW	log2FC EF/EW	padj EF/EW	log2FC TF/EW	padj TF/EW	description	accession
Map4k5	-45.6741	7.11	0.16	-8.42	0.11	3.81	0.31	-1.30	0.5	Mitogen-activated protein kinase kinase kinase kinase 5	E9Q1T3
Map6	-1.2421	0.82	0.0004	-0.36	0.06	0.21	0.86	0.46	0.26	Microtubule-associated protein 6	Q7TSJ2
Map6d1	-0.7202	0.44	0.12	-0.80	0.0060	-0.17	0.2	-0.36	0.0010	MAP6 domain-containing protein 1	Q14BB9
Map7	-0.0048	0.04	0.69	-0.59	0.056	0.26	0.26	-0.55	0.0450	Ensconsin	D3YWN7
Map7d1	-0.0771	0.32	0.58	-0.74	0.0420	0.41	0.75	-0.42	0.34	MAP7 domain-containing protein 1	A2AJI0
Map7d2	0.0001	0.12	0.46	0.13	0.94	-0.04	0.27	0.25	0.36	MAP7 domain-containing protein 2	A2AG50
Mapk1	-0.0085	-0.07	0.7	0.40	0.0110	-0.27	0.064	0.32	0.0090	Mitogen-activated protein kinase 1	P63085
Mapk10	0.0000	0.04	1	-0.71	0.33	-0.33	0.6	-0.67	0.25	Mitogen-activated protein kinase	A0A5F8MP75
Mapk3	-0.0148	-0.30	0.5	0.46	0.44	-0.19	0.7	0.16	0.85	Mitogen-activated protein kinase 3	Q63844
Mapk8ip3	-0.0048	0.33	0.18	-0.12	0.69	0.20	0.52	0.21	0.45	C-Jun-amino-terminal kinase-interacting protein 3	E9Q6B6
Mapre2	-0.3639	1.12	0.0380	-0.38	0.25	-0.33	0.75	0.73	0.11	Microtubule-associated protein RP/EB family member 2 (Fragment)	D3YYK8
Mapre3	0.0292	0.38	0.17	0.28	0.44	0.08	0.74	0.66	0.0310	Microtubule-associated protein RP/EB family member 3	Q6PER3
Mapt	-0.0079	0.17	0.11	-0.09	0.29	0.14	0.41	0.08	0.34	Microtubule-associated protein	A0A0A0MQC7
Marcks	0.0005	-0.04	0.77	-0.17	0.26	0.08	0.88	-0.21	0.31	Myristoylated alanine-rich C-kinase substrate	P26645
Mark2	-0.8489	0.92	0.0460	-0.69	0.1	-0.04	0.96	0.24	0.47	Non-specific serine/threonine protein kinase	A0A0R4J0B1
Mark3	-0.0060	0.89	0.97	-1.55	0.47	-0.44	0.37	-0.66	0.21	Non-specific serine/threonine protein kinase	A0A1Y7VNZ6
Mars1	0.0000	0.47	0.0120	0.22	1	0.40	0.071	0.69	0.22	Methionine–tRNA ligase, cytoplasmic	Q68FL6
Mast1	-3.3661	2.43	0.44	-4.55	0.14	2.60	0.39	-2.12	0.28	Microtubule-associated serine/threonine-protein kinase 1	Q9R1L5
Mat2a	-0.0865	0.44	0.75	-1.91	0.15	0.27	0.96	-1.47	0.15	S-adenosylmethionine synthase	A0A0U1RNK6
Matn4	-0.0258	-0.95	0.0001	0.07	0.8	-0.23	0.059	-0.87	0.0001	Matrilin-4	F2Z3U4
Matr3	-0.1564	0.29	0.14	-0.52	0.061	-0.91	0.0020	-0.23	0.48	Matrin-3	Q8K310
Mb21d2	-0.2364	-0.29	0.15	0.43	0.0050	0.36	0.098	0.15	0.3	Protein MB21D2	D3Z742
Mblac2	-9.3934	4.93	0.11	-4.73	0.38	5.36	0.055	0.20	0.76	Metallo-beta-lactamase domain-containing protein 2	Q8BL86
Mbp	0.0083	-0.20	0.82	-1.00	0.33	-0.57	0.16	-1.20	0.0280	Myelin basic protein	A0A498WGS3
Mcat	-0.6904	-1.01	0.087	0.70	0.12	0.23	0.57	-0.31	0.23	Malonyl-CoA-acyl carrier protein transacylase, mitochondrial (Fragment)	A0A2R8VHT7
Mccc1	0.0020	0.05	0.87	0.82	0.15	-0.12	0.65	0.86	0.0360	Methylcrotonoyl-CoA carboxylase subunit alpha, mitochondrial	Q99MR8
Mccc2	0.0132	0.13	0.45	0.66	0.36	0.13	0.27	0.79	0.074	Methylcrotonoyl-CoA carboxylase beta chain, mitochondrial	Q3ULD5

Table S2: Differentially expressed proteins in cerebral cortex *(continued)*

gene	$\pi_{TW/EW} \times \pi_{TF/TW}$	log2FC TW/EW	padj TW/EW	log2FC TF/TW	padj TF/TW	log2FC EF/EW	padj EF/EW	log2FC TF/EW	padj TF/EW	description	accession
Mcee	-0.0057	0.02	0.65	-0.86	0.0170	0.15	0.6	-0.84	0.0140	Methylmalonyl-CoA epimerase, mitochondrial	A0A0U1RQ27
Mcts1	0.0061	-0.22	0.44	-0.19	0.39	0.05	0.74	-0.40	0.0180	Malignant T-cell-amplified sequence 1	Q9DB27
Mcu	0.0308	0.45	0.43	0.47	0.4	0.36	0.9	0.92	0.14	Calcium uniporter protein, mitochondrial	Q3UMR5
Mdga2	0.0000	-0.39	0.26	-0.01	1	0.10	0.71	-0.41	0.07	MAM domain-containing glycosylphosphatidylinositol anchor protein 2	Q3UV20
Mdh1	0.0007	-0.35	0.0028	-0.06	0.97	-0.27	0.0190	-0.40	0.0005	Malate dehydrogenase, cytoplasmic	P14152
Mdh2	-0.0417	0.04	0.38	-0.62	0.0001	-0.07	0.16	-0.58	0.0006	Malate dehydrogenase, mitochondrial	P08249
Mdn1	-0.0083	1.29	0.44	-0.50	0.92	-0.70	0.66	0.79	0.41	Midasin	J3QMC5
Me1	-0.0011	-0.01	0.36	0.40	0.25	0.36	0.33	0.39	0.19	Malic enzyme	Q3TQP6
Me2	0.0487	0.61	0.52	0.60	0.34	0.67	0.47	1.21	0.0190	NAD-dependent malic enzyme, mitochondrial	Q99KE1
Me3	-0.0227	-0.34	0.94	0.83	0.0010	-0.29	0.85	0.49	0.0360	NADP-dependent malic enzyme, mitochondrial	Q8BMF3
Mecp2	-0.8042	2.22	0.35	-1.84	0.37	0.69	0.56	0.38	0.74	Methyl-CpG-binding protein 2	Q9Z2D6
Mecr	0.0172	0.22	0.49	0.42	0.25	0.28	0.12	0.64	0.0280	Enoyl-[acyl-carrier-protein] reductase, mitochondrial	Q9DCS3
Mef2d	-0.0457	1.27	0.34	-0.37	0.62	0.32	0.96	0.90	0.61	Myocyte-specific enhancer factor 2D	E9Q5E0
Metap1	0.0560	0.57	0.63	0.49	0.1	0.02	0.79	1.06	0.055	Methionine aminopeptidase 1	Q8BP48
Metap2	-0.0019	-0.02	0.44	0.21	0.058	0.03	0.95	0.20	0.0100	Methionine aminopeptidase 2	O08663
Mfge8	-3.8005	-0.58	0.0004	0.55	0.0003	0.49	0.0003	-0.03	0.79	Lactadherin	P21956
Mia2	0.0486	0.19	0.26	0.42	0.091	0.13	0.52	0.61	0.0110	Melanoma inhibitory activity protein 2	A0A1W2P711
Mia3	0.0013	0.63	0.25	0.11	0.93	-0.37	0.93	0.74	0.51	Transport and Golgi organization protein 1 homolog	Q8BI84
Mib2	-0.1705	0.53	0.73	-1.88	0.056	-0.77	0.31	-1.35	0.058	E3 ubiquitin-protein ligase MIB2	Q8R516
Mical3	-0.6771	1.28	0.0270	-0.76	0.36	0.52	0.35	0.52	0.13	F-actin monooxygenase	A0A5F8MPF3
Micu1	-1.2603	1.07	0.0007	-0.47	0.16	0.28	0.3	0.59	0.59	Calcium uptake protein 1, mitochondrial	Q8VCX5
Micu3	-0.0863	0.90	0.051	-0.15	0.32	-0.05	0.72	0.76	0.35	Calcium uptake protein 3, mitochondrial	Q9CTY5
Mink1	-6.4775	1.04	0.0010	-1.06	0.0110	0.39	0.12	-0.02	0.85	Misshapen-like kinase 1	G3X9G2
Mios	0.0003	0.36	0.71	0.41	0.97	0.14	0.63	0.77	0.79	GATOR complex protein MIOS	Q8VE19
Mipep	-0.0025	0.32	0.26	-0.13	0.79	-0.69	0.87	0.20	0.25	Mitochondrial intermediate peptidase	A0A286YDC5
Mlec	0.0343	0.34	0.65	0.82	0.22	1.09	0.0070	1.16	0.1	Malectin	Q6ZQI3
Mlf2	-0.1996	0.26	0.45	-1.13	0.0110	0.23	0.31	-0.87	0.0047	Myeloid leukemia factor 2	Q99KX1
Mlycd	-0.0556	-0.43	0.12	0.22	0.23	-0.04	0.91	-0.21	0.32	Malonyl-CoA decarboxylase, mitochondrial	Q99J39
Mmaa	-0.1384	0.38	0.14	-0.23	0.0140	-0.02	0.96	0.15	0.55	Methylmalonic aciduria type A homolog, mitochondrial	Q8C7H1
Mmab	-0.0156	0.29	0.34	-0.28	0.39	0.05	0.85	0.02	0.87	Corrinoid adenosyltransferase	Q9D273

Table S2: Differentially expressed proteins in cerebral cortex *(continued)*

gene	$\pi_{TW/EW} \times \pi_{TF/TW}$	log2FC TW/EW	padj TW/EW	log2FC TF/TW	padj TF/TW	log2FC EF/EW	padj EF/EW	log2FC TF/EW	padj TF/EW	description	accession
Mmut	0.0011	-0.04	0.85	-0.49	0.15	-0.81	0.069	-0.53	0.062	Methylmalonyl-CoA mutase, mitochondrial	P16332
Mobp	-3.3975	2.35	0.5	-5.01	0.11	0.01	0.76	-2.66	0.42	Myelin-associated oligodendrocyte basic protein	Q9D2P8
Mog	-0.0001	-0.37	0.87	0.11	0.92	-0.27	0.0120	-0.26	0.0010	Myelin-oligodendrocyte glycoprotein	Q29ZQ5
Mogs	-0.0430	0.36	0.31	-0.39	0.25	0.01	0.63	-0.03	0.71	Mannosyl-oligosaccharide glucosidase	Q80UM7
Mon2	0.1603	-0.32	0.67	-6.67	0.37	0.24	0.49	-6.99	0.19	Protein MON2 homolog	B9EKJ3
Mpc1	-0.0003	-0.08	0.85	0.18	0.54	0.37	0.18	0.10	0.65	Mitochondrial pyruvate carrier 1	P63030
Mpdz	-0.0099	0.26	0.54	-0.40	0.44	-0.13	0.69	-0.14	0.67	Mpdz protein	B2RQR2
Mpp1	2.7978	-1.40	0.075	-1.30	0.0430	-0.69	0.36	-2.69	0.0020	55 kDa erythrocyte membrane protein	A2AN84
Mpp2	-0.0001	-0.21	0.76	0.20	0.94	-0.12	0.31	-0.02	0.74	MAGUK p55 subfamily member 2	Q9WV34
Mpp3	0.0000	0.34	0.094	0.28	1	0.40	0.082	0.62	0.0010	MAGUK p55 subfamily member 3	Q6XE40
Mpp6	0.0000	-0.02	0.91	-0.07	0.45	-0.03	0.61	-0.09	0.43	MAGUK p55 subfamily member 6	Q9JLB0
Mprip	-9.1973	2.12	0.1	-3.03	0.0370	0.20	0.75	-0.91	0.34	Myosin phosphatase Rho-interacting protein	Q5SWZ5
Mpst	-0.0066	-0.38	0.66	0.35	0.53	-0.62	0.38	-0.03	0.72	3-mercaptopyruvate sulfurtransferase	Q99J99
Mras	-0.0311	0.28	0.18	-0.22	0.21	-0.08	0.77	0.06	0.79	Ras-related protein M-Ras	O08989
Mrpl1	-1.5951	1.78	0.0440	-0.83	0.16	1.86	0.07	0.95	0.56	39S ribosomal protein L1, mitochondrial	Q99N96
Mrpl11	-0.2642	0.92	0.0110	-0.46	0.48	0.01	0.47	0.46	0.37	39S ribosomal protein L11, mitochondrial (Fragment)	A0A494B9H5
Mrpl12	-0.0038	0.35	0.54	-0.27	0.71	0.27	0.31	0.09	0.8	39S ribosomal protein L12, mitochondrial	Q9DB15
Mrpl19	0.0001	-0.17	0.84	-0.09	0.83	-0.64	0.71	-0.26	0.63	39S ribosomal protein L19, mitochondrial	Q9D338
Mrpl2	-0.4075	0.41	0.1	-0.64	0.0280	-0.17	0.51	-0.23	0.0280	39S ribosomal protein L2, mitochondrial	B1B1D8
Mrpl27	0.0046	0.26	0.18	0.10	0.58	0.31	0.45	0.36	0.089	39S ribosomal protein L27, mitochondrial	Q99N92
Mrpl37	-0.0760	0.43	0.17	-0.36	0.23	-0.21	0.78	0.07	0.96	39S ribosomal protein L37, mitochondrial	Q921S7
Mrpl39	0.0000	0.19	0.94	-0.20	0.98	-0.21	0.97	-0.01	0.89	39S ribosomal protein L39, mitochondrial	Q9JKF7
Mrpl43	-0.6108	0.72	0.0100	-0.40	0.087	-0.39	0.47	0.33	0.23	39S ribosomal protein L43, mitochondrial	Q5RL20
Mrpl45	-0.0036	0.10	0.56	-0.31	0.35	0.16	0.81	-0.21	0.52	39S ribosomal protein L45, mitochondrial	Q9D0Q7
Mrpl53	-0.0872	0.85	0.26	-0.55	0.48	0.43	0.58	0.29	0.7	39S ribosomal protein L53, mitochondrial	Q9D1H8
Mrps28	-0.0748	0.52	0.43	-0.69	0.27	1.26	0.2	-0.18	0.8	28S ribosomal protein S28, mitochondrial	Q9CY16
Mrps36	-0.0071	0.18	0.18	-0.05	0.086	-0.31	0.48	0.14	0.99	28S ribosomal protein S36, mitochondrial	Q9CQX8
Mrrf	-0.0262	0.62	0.23	-0.28	0.58	0.33	0.99	0.35	0.79	Ribosome-recycling factor, mitochondrial	Q9D6S7
Mrtfa	-0.0174	0.22	0.5	-0.40	0.22	0.34	0.31	-0.19	0.35	Myocardin-related transcription factor A	A0A5F8MQ33
Msn	-0.0162	-0.11	0.56	0.34	0.0190	0.42	0.0060	0.23	0.11	Moesin	P26041
Msrb2	-0.4210	0.60	0.0440	-0.37	0.0400	0.34	0.52	0.23	0.72	Methionine-R-sulfoxide reductase B2, mitochondrial	Q78J03
Mtarc2	-0.0008	0.09	0.13	-0.03	0.47	-0.14	0.17	0.06	0.97	Mitochondrial amidoxime reducing component 2	Q922Q1

Table S2: Differentially expressed proteins in cerebral cortex *(continued)*

gene	$\pi_{TW/EW}\times$ $\pi_{TF/TW}$	log2FC TW/EW	padj TW/EW	log2FC TF/TW	padj TF/TW	log2FC EF/EW	padj EF/EW	log2FC TF/EW	padj TF/EW	description	accession
Mtatp8	-0.0003	0.01	0.75	-0.32	0.17	0.11	0.91	-0.31	0.096	ATP synthase protein 8	P03930
Mtch1	-0.0331	0.90	0.44	-0.48	0.61	1.47	0.0320	0.42	0.62	Mitochondrial carrier homolog 1	Q791T5
Mtch2	-0.0006	0.22	0.54	-0.04	0.53	0.17	0.82	0.18	0.98	Mitochondrial carrier homolog 2	A2AFW6
Mtco2	-0.7628	0.92	0.17	-1.17	0.12	1.03	0.095	-0.25	0.83	Cytochrome c oxidase subunit 2	P00405
Mtdh	1.1084	3.77	0.47	3.79	0.58	3.86	0.41	7.56	0.22	Protein LYRIC	Q80WJ7
Mtfr1l	-0.0652	0.48	0.64	-0.91	0.17	-0.12	0.75	-0.43	0.4	Mitochondrial fission regulator 1-like	Q9CWE0
Mthfd1	0.0028	-0.23	0.65	-0.07	0.12	0.15	0.69	-0.30	0.061	C-1-tetrahydrofolate synthase, cytoplasmic	Q922D8
Mthfd1l	0.0484	-0.09	0.64	-1.10	0.0030	0.27	0.19	-1.19	0.0007	Monofunctional C1-tetrahydrofolate synthase, mitochondrial	Q3V3R1
Mtmr1	-2.7887	1.74	0.0010	-0.47	0.073	-0.06	0.9	1.27	0.0320	Phosphatidylinositol-3,5-bisphosphate 3-phosphatase	I7HJQ9
Mtmr12	-0.0004	1.29	0.92	-1.06	0.98	-0.20	0.95	0.22	0.62	Myotubularin-related protein 12	Q80TA6
Mtor	-1.3641	1.13	0.0160	-0.73	0.12	-0.06	0.93	0.40	0.61	Serine/threonine-protein kinase mTOR	Q9JLN9
Mtpap	-1.9642	1.05	0.051	-1.26	0.071	0.90	0.0470	-0.21	0.53	Poly(A) RNA polymerase, mitochondrial	Q9D0D3
Mtus2	0.0001	0.81	0.15	0.01	0.95	0.62	0.47	0.82	0.15	Microtubule-associated tumor suppressor candidate 2 homolog	Q3UHD3
Mtx1	-0.2070	0.52	0.12	-0.40	0.083	0.28	0.88	0.12	0.86	Metaxin-1	A0A6I8MWZ8
Mtx2	0.0000	-0.02	0.86	0.30	1	0.12	0.9	0.28	0.97	Metaxin-2	O88441
Mvp	0.0773	-0.24	0.1	-0.35	0.12	0.09	0.52	-0.59	0.0017	Major vault protein	E9Q3X0
Mycbp2	-0.0026	0.15	0.94	-1.18	0.28	-0.61	0.2	-1.03	0.0010	E3 ubiquitin-protein ligase MYCBP2	Q7TPH6
Myef2	-0.2321	0.58	0.4	-0.88	0.072	-0.68	0.19	-0.30	0.45	Myelin expression factor 2 (Fragment)	G8JL68
Myh10	-3.2086	0.97	0.33	-2.29	0.0010	0.05	0.72	-1.31	0.0001	Myosin-10	Q5SV64
Myh14	-4.7014	1.59	0.097	-1.60	0.0150	1.22	0.2	-0.01	0.76	Myosin-14	K3W4R2
Myh9	-3.6517	0.81	0.45	-3.25	0.0001	-0.11	0.93	-2.44	0.0001	Myosin-9	Q8VDD5
Myl12b	-0.0732	1.01	0.39	-1.10	0.69	0.47	0.45	-0.09	0.82	Myosin regulatory light chain 12B	Q3THE2
Myl6	0.0004	-0.20	0.59	-0.09	0.8	0.45	0.0340	-0.29	0.67	Myosin light polypeptide 6	A0A1W2P6F6
Mylk	-0.4344	0.71	0.5	-2.12	0.11	0.20	0.63	-1.41	0.3	Myosin light chain kinase, smooth muscle	B1B1A8
Myo1b	0.0072	-0.06	0.48	-0.44	0.14	0.26	0.54	-0.50	0.45	Myo1b protein	Q7TQD7
Myo1c	-0.0680	0.55	0.67	-1.18	0.25	0.29	0.96	-0.63	0.6	Unconventional myosin-Ic	Q9WTI7
Myo1d	-0.6315	0.48	0.29	-1.56	0.0270	0.60	0.0170	-1.08	0.21	Unconventional myosin-Id	Q5SYD0
Myo5a	-18.6516	0.99	0.0010	-1.57	0.0001	0.45	0.069	-0.58	0.0070	Unconventional myosin-Va	Q99104
Myo6	-0.3965	0.83	0.56	-1.58	0.063	1.21	0.29	-0.75	0.35	Unconventional myosin-6	E9Q175
Nap1l1	-0.0009	-0.12	0.54	0.12	0.6	-0.01	0.97	0.00	0.96	Nucleosome assembly protein 1-like 1	Q3TF41
Napa	-0.0012	-0.45	0.082	0.09	0.94	-0.31	0.18	-0.37	0.088	Alpha-soluble NSF attachment protein	Q9DB05
Napb	-0.3701	0.44	0.069	-0.51	0.0380	-0.55	0.24	-0.07	0.75	Beta-soluble NSF attachment protein	P28663
Napg	0.0000	-0.06	1	0.16	0.83	-0.14	0.81	0.10	0.83	Gamma-soluble NSF attachment protein (Fragment)	D3Z4B2
Nars	-0.0007	-0.25	0.05	0.24	0.98	0.06	0.51	-0.01	0.8	Asparagine–tRNA ligase	A0A498WGK2

Table S2: Differentially expressed proteins in cerebral cortex *(continued)*

gene	$\pi_{TW/EW}\times$ $\pi_{TF/TW}$	log2FC TW/EW	padj TW/EW	log2FC TF/TW	padj TF/TW	log2FC EF/EW	padj EF/EW	log2FC TF/EW	padj TF/EW	description	accession
Nasp	-0.0218	-0.38	0.35	0.50	0.56	-0.19	0.54	0.12	0.82	Nuclear autoantigenic sperm protein	B1AU75
Nav1	-1.0974	1.26	0.18	-1.22	0.11	-0.43	0.38	0.04	0.65	Neuron navigator 1	Q8CH77
Naxd	0.1349	0.14	0.5	0.80	0.0001	-0.14	0.6	0.95	0.0030	ATP-dependent (S)-NAD(P)H-hydrate dehydratase	J3QMM7
Nbea	-0.0001	0.04	0.62	-0.04	0.58	-0.02	0.94	-0.01	0.9	Neurobeachin	Q9EPN1
Ncam1	0.0006	-0.15	0.47	-0.06	0.64	0.08	0.69	-0.21	0.3	Neural cell adhesion molecule 1 (Fragment)	A0A0A6YY91
Ncam2	-0.0082	-0.45	0.44	0.23	0.6	0.19	0.74	-0.23	0.33	Neural cell adhesion molecule 2	O35136
Ncan	-0.0025	-0.29	0.15	0.08	0.74	-0.26	0.0040	-0.21	0.16	Neurocan core protein	A0A0R4IZX5
Ncdn	0.0062	-0.33	0.37	-0.19	0.59	-0.39	0.057	-0.52	0.063	Neurochondrin	Q9Z0E0
Nceh1	0.0000	0.05	0.86	0.02	0.92	-0.15	0.9	0.07	0.66	Neutral cholesterol ester hydrolase 1	Q8BLF1
Nck1	0.0036	0.05	0.88	1.64	0.16	1.70	0.12	1.69	0.16	Cytoplasmic protein NCK1	Q99M51
Nck2	-0.0034	0.35	0.4	-0.15	0.69	-0.35	0.39	0.21	0.38	Cytoplasmic protein NCK2	O55033
Nckap1	0.0000	0.00	0.63	0.23	0.83	0.10	0.54	0.22	0.55	Nck-associated protein 1	A2AS98
Nckipsd	-0.0003	-0.12	0.61	0.08	0.74	-0.08	0.84	-0.04	0.99	NCK-interacting protein with SH3 domain	Q9ESJ4
Ncl	-1.4811	0.58	0.0005	-0.32	0.0040	-0.11	0.74	0.26	0.22	Nucleolin	P09405
Ncln	-0.0251	0.40	0.62	-0.78	0.41	0.59	0.4	-0.38	0.78	Nicalin	D3YU17
Ncoa7	0.0000	0.02	0.89	0.28	0.93	0.36	0.19	0.30	0.95	Nuclear receptor coactivator 7	A0A1L1SSS4
Ncstn	-22.9110	8.17	0.083	-6.70	0.41	8.46	0.0280	1.47	0.3	Nicastrin	P57716
Ndel1	-0.1820	0.89	0.22	-0.74	0.38	0.96	0.23	0.16	0.68	Nuclear distribution protein nudE-like 1	Q9ERR1
Ndrg1	-0.5551	1.79	0.054	-0.35	0.2	0.91	0.47	1.43	0.13	Protein NDRG1	Q62433
Ndrg2	0.0003	-0.05	0.73	-0.13	0.51	-0.08	0.5	-0.18	0.41	Protein NDRG2	Q9QYG0
Ndrg3	-0.0266	-0.26	0.54	0.48	0.16	-0.19	0.41	0.22	0.32	N-myc downstream-regulated gene 3 protein	Q8VCV2
Ndufa10	-0.0427	0.15	0.49	-0.77	0.064	-0.26	0.76	-0.62	0.13	NADH dehydrogenase [ubiquinone] 1 alpha subcomplex subunit 10, mitochondrial	Q99LC3
Ndufa12	-1.4049	0.43	0.28	-1.97	0.0010	-1.23	0.14	-1.55	0.0480	NADH dehydrogenase [ubiquinone] 1 alpha subcomplex subunit 12	A0A0R4J275
Ndufa13	-1.4536	0.61	0.0240	-0.78	0.0130	0.08	0.65	-0.17	0.59	NADH dehydrogenase [ubiquinone] 1 alpha subcomplex subunit 13	Q9ERS2
Ndufa2	-0.0023	0.16	0.67	-0.27	0.49	0.22	0.69	-0.11	0.65	NADH dehydrogenase [ubiquinone] 1 alpha subcomplex subunit 2	Q9CQ75
Ndufa4	-1.0978	0.51	0.0110	-0.51	0.0070	0.28	0.31	0.00	0.92	Cytochrome c oxidase subunit NDUFA4	Q62425
Ndufa5	-0.0839	0.31	0.36	-0.57	0.085	-0.36	0.22	-0.26	0.44	NADH dehydrogenase [ubiquinone] 1 alpha subcomplex subunit 5	Q9CPP6
Ndufa6	-0.1352	0.33	0.17	-0.42	0.054	0.04	0.59	-0.08	0.37	NADH dehydrogenase [ubiquinone] 1 alpha subcomplex subunit 6	Q9CQZ5

Table S2: Differentially expressed proteins in cerebral cortex *(continued)*

gene	$\pi_{TW/EW} \times \pi_{TF/TW}$	log2FC TW/EW	padj TW/EW	log2FC TF/TW	padj TF/TW	log2FC EF/EW	padj EF/EW	log2FC TF/EW	padj TF/EW	description	accession
Ndufa7	-0.0010	0.03	0.9	-0.32	0.0050	0.08	0.73	-0.29	0.0400	NADH dehydrogenase [ubiquinone] 1 alpha subcomplex subunit 7	Q9Z1P6
Ndufa8	-0.0469	0.31	0.57	-0.70	0.13	-0.12	0.8	-0.39	0.3	NADH dehydrogenase [ubiquinone] 1 alpha subcomplex subunit 8	Q9DCJ5
Ndufa9	-3.0458	0.65	0.055	-0.93	0.0001	-0.03	0.85	-0.28	0.1	NADH dehydrogenase [ubiquinone] 1 alpha subcomplex subunit 9, mitochondrial	A0A0R3P9C8
Ndufaf3	-0.0426	0.29	0.22	-0.35	0.23	0.18	0.23	-0.05	0.82	NADH dehydrogenase [ubiquinone] 1 alpha subcomplex assembly factor 3	Q9JKL4
Ndufaf4	-0.0037	-0.46	0.45	0.15	0.7	-0.12	0.55	-0.31	0.32	NADH dehydrogenase [ubiquinone] 1 alpha subcomplex assembly factor 4	Q9D1H6
Ndufb1	-0.0018	0.28	0.59	-0.17	0.69	0.40	0.34	0.10	0.74	NADH dehydrogenase [ubiquinone] 1 beta subcomplex subunit 1	P0DN34
Ndufb10	-0.0158	0.11	0.6	-0.40	0.0240	-0.04	0.72	-0.29	0.19	NADH dehydrogenase [ubiquinone] 1 beta subcomplex subunit 10	Q9DCS9
Ndufb11	0.0000	-0.18	0.61	0.02	0.96	-0.04	0.73	-0.16	0.38	NADH dehydrogenase [ubiquinone] 1 beta subcomplex subunit 11, mitochondrial	O09111
Ndufb3	-0.0001	0.03	0.77	-0.10	0.58	0.33	0.59	-0.07	0.76	NADH dehydrogenase [ubiquinone] 1 beta subcomplex subunit 3	Q9CQZ6
Ndufb4	-0.6011	0.83	0.1	-0.91	0.16	0.08	0.78	-0.08	0.8	NADH dehydrogenase [ubiquinone] 1 beta subcomplex subunit 4	Q9CQC7
Ndufb5	-0.0016	0.35	0.51	-0.08	0.64	0.49	0.38	0.27	0.62	Complex I-SGDH (Fragment)	D3YX99
Ndufb6	-0.0016	0.25	0.72	-0.24	0.65	0.13	0.96	0.01	0.93	Complex I-B17	A2AP32
Ndufb8	-0.0004	0.01	0.8	-0.34	0.078	0.15	0.96	-0.33	0.075	NADH dehydrogenase [ubiquinone] 1 beta subcomplex subunit 8, mitochondrial	Q9D6J5
Ndufb9	-0.0715	0.36	0.19	-0.37	0.18	0.30	0.25	0.00	0.98	NADH dehydrogenase [ubiquinone] 1 beta subcomplex subunit 9	Q9CQJ8
Ndufc2	-0.0118	0.18	0.65	-0.50	0.2	0.32	0.59	-0.32	0.23	NADH dehydrogenase [ubiquinone] 1 subunit C2	Q9CQ54
Ndufs1	-0.2399	0.32	0.14	-0.51	0.0190	0.35	0.19	-0.18	0.42	NADH-ubiquinone oxidoreductase 75 kDa subunit, mitochondrial	Q91VD9
Ndufs2	-0.0919	0.39	0.61	-0.86	0.053	-0.91	0.0460	-0.47	0.18	Complex I-49kD	D3YXT0
Ndufs3	-3.1315	0.84	0.0320	-1.04	0.0040	-0.13	0.79	-0.20	0.8	NADH dehydrogenase [ubiquinone] iron-sulfur protein 3, mitochondrial	Q9DCT2
Ndufs4	-0.0038	0.08	0.87	-0.59	0.0470	0.04	0.67	-0.52	0.051	NADH dehydrogenase [ubiquinone] iron-sulfur protein 4, mitochondrial	E9QPX3
Ndufs7	-2.0258	0.53	0.0330	-0.86	0.0010	-0.77	0.0430	-0.33	0.23	NADH dehydrogenase [ubiquinone] iron-sulfur protein 7, mitochondrial	Q9DC70
Ndufs8	-1.7125	0.86	0.13	-1.17	0.0120	0.25	0.67	-0.31	0.71	NADH dehydrogenase [ubiquinone] iron-sulfur protein 8, mitochondrial	Q8K3J1

Table S2: Differentially expressed proteins in cerebral cortex *(continued)*

gene	$\pi_{TW/EW} \times \pi_{TF/TW}$	log2FC TW/EW	padj TW/EW	log2FC TF/TW	padj TF/TW	log2FC EF/EW	padj EF/EW	log2FC TF/EW	padj TF/EW	description	accession
Ndufv1	-0.0010	0.12	0.7	-0.21	0.54	0.02	0.81	-0.09	0.74	NADH dehydrogenase [ubiquinone] flavoprotein 1, mitochondrial	D3YUM1
Ndufv2	-0.0667	0.28	0.41	-0.46	0.0460	0.16	0.66	-0.19	0.17	NADH dehydrogenase [ubiquinone] flavoprotein 2, mitochondrial	Q9D6J6
Ndufv3	-0.0011	0.11	0.43	-0.09	0.5	0.33	0.091	0.02	0.79	Complex I-9kD	Q3U422
Nebl	-4.2480	0.49	0.0020	-0.84	0.0001	-0.01	0.89	-0.35	0.0200	LIM zinc-binding domain-containing Nebulette	Q9DC07
Necap1	-0.0008	-0.10	0.93	0.78	0.47	-0.27	0.53	0.68	0.48	Adaptin ear-binding coat-associated protein 1	Q9CR95
Nectin1	0.0000	0.01	1	0.34	0.14	0.23	0.089	0.35	0.059	Nectin-1	Q9JKF6
Nedd4	-0.0001	-0.03	0.93	0.19	0.51	0.11	0.76	0.16	0.62	E3 ubiquitin-protein ligase NEDD4	P46935
Nefh	-0.9549	1.02	0.7	-3.26	0.0140	-0.24	0.82	-2.24	0.0290	Neurofilament heavy polypeptide	P19246
Nefl	-47.1671	1.97	0.0046	-2.83	0.0002	0.16	0.67	-0.85	0.0130	Neurofilament light polypeptide	P08551
Nefm	-10.6520	1.20	0.12	-2.41	0.0001	0.04	0.81	-1.21	0.0001	160 kDa neurofilament protein	A0A0R4J036
Negr1	-0.1388	0.63	0.58	-1.17	0.16	-0.01	0.74	-0.54	0.46	Neuronal growth regulator 1	A0A4W9
Nell2	0.3622	0.53	0.24	0.77	0.0370	0.09	0.74	1.30	0.0160	Protein kinase C-binding protein NELL2	Q8BM06
Nf1	-0.0352	0.55	0.0230	-0.16	0.57	0.24	0.61	0.39	0.48	Neurofibromin	Q04690
Nfasc	0.0023	-0.12	0.44	-0.07	0.17	0.42	0.096	-0.19	0.66	Neurofascin	A0A087WPX3
Nfs1	-0.3636	0.59	0.0008	-0.35	0.27	-0.08	0.96	0.24	0.58	Cysteine desulfurase, mitochondrial	Q9Z1J3
Ngef	-0.0048	-0.32	0.2	0.12	0.66	-0.40	0.075	-0.20	0.55	Ephexin-1	E9QK62
Nhsl2	-132.9397	5.70	0.0480	-7.01	0.0030	0.99	0.63	-1.31	0.32	NHS-like protein 2	B1AXH1
Niban2	0.0044	-0.22	0.42	-0.23	0.59	0.03	0.83	-0.45	0.11	Protein Niban 2	Q8R1F1
Nipsnap1	0.0499	0.80	0.0066	0.07	0.39	0.09	0.88	0.87	0.077	Protein NipSnap homolog 1	O55125
Nipsnap2	0.2199	0.25	0.0230	0.56	0.11	0.32	0.0160	0.81	0.0001	Protein NipSnap homolog 2	O55126
Nlgn2	-0.0102	-0.17	0.43	0.25	0.22	-0.55	0.32	0.09	0.59	Neuroligin-2	Q69ZK9
Nlgn3	-0.7815	-3.16	0.2	1.21	0.51	-1.53	0.54	-1.95	0.49	Neuroligin-3	A2AGI3
Nln	-0.0039	-0.38	0.077	0.06	0.7	-0.07	0.67	-0.31	0.14	Neurolysin, mitochondrial	Q91YP2
Nme1	-0.0004	-0.29	0.82	0.05	0.49	-0.04	0.99	-0.24	0.39	Nucleoside diphosphate kinase A	P15532
Nme3	-0.0024	-1.10	0.09	0.02	0.79	-0.95	0.086	-1.08	0.13	Nucleoside diphosphate kinase 3	Q9WV85
Nme7	0.0000	0.05	0.98	0.06	0.86	0.15	0.47	0.11	0.5	Nucleoside diphosphate kinase 7	Q3UMG6
Nmral1	0.2160	-0.35	0.0220	-0.50	0.18	-0.51	0.063	-0.85	0.0014	NmrA-like family domain-containing protein 1	D3YU12
Nmt1	-0.0028	-0.07	0.67	0.36	0.23	0.25	0.4	0.29	0.073	Glycylpeptide N-tetradecanoyltransferase 1	O70310
Nmt2	-0.0083	-0.22	0.74	0.58	0.32	-0.35	0.79	0.36	0.47	Glycylpeptide N-tetradecanoyltransferase	A2AJH3
Nnt	-0.0428	0.15	0.5	-0.64	0.0330	-0.44	0.06	-0.48	0.089	NAD(P) transhydrogenase, mitochondrial	Q61941
Nolc1	0.0002	-0.35	0.7	-0.20	0.96	-0.21	0.78	-0.55	0.55	Nucleolar and coiled-body phosphoprotein 1	A0A286YDA2
Nomo1	0.0001	0.11	0.51	0.38	0.98	-0.03	0.54	0.49	0.52	Nodal modulator 1	Q6GQT9

Table S2: Differentially expressed proteins in cerebral cortex *(continued)*

gene	$\pi_{TW/EW} \times \pi_{TF/TW}$	log2FC TW/EW	padj TW/EW	log2FC TF/TW	padj TF/TW	log2FC EF/EW	padj EF/EW	log2FC TF/EW	padj TF/EW	description	accession
Nono	-0.0026	0.80	0.82	-0.75	0.89	-0.13	0.29	0.05	0.64	Non-POU domain-containing octamer-binding protein	Q99K48
Nop56	-54.5788	7.56	0.14	-8.82	0.11	-1.18	0.59	-1.26	0.54	Nucleolar protein 56	Q9D6Z1
Nos1	-0.0034	-0.16	0.39	0.14	0.43	-0.04	0.94	-0.01	0.98	Constitutive NOS	F8WGF2
Nova1	0.0199	-0.32	0.64	-0.43	0.18	-0.40	0.48	-0.75	0.0210	RNA-binding protein Nova-1	Q9JKN6
Nova2	-0.1194	-0.33	0.0190	0.20	0.089	0.09	0.85	-0.13	0.22	NOVA alternative-splicing regulator 2	A0A1W2P872
Npepps	0.0165	-0.13	0.33	-0.22	0.063	-0.03	0.61	-0.35	0.43	Puromycin-sensitive aminopeptidase	Q11011
Npm1	0.0000	0.35	0.0300	0.02	0.99	-0.21	0.2	0.37	0.0025	Nucleophosmin	Q61937
Nptn	-2.5515	-0.63	0.0010	0.45	0.0010	-0.19	0.081	-0.18	0.13	Neuroplastin	P97300
Nptx1	0.0000	-0.02	0.76	-0.05	0.45	-0.19	0.58	-0.07	0.68	Neuronal pentraxin-1	Q62443
Nptxr	-0.0006	-0.04	0.82	0.29	0.25	-0.25	0.34	0.25	0.28	Neuronal pentraxin receptor	E9PZM8
Nras	0.0000	0.00	0.77	-0.61	0.11	-0.27	0.98	-0.61	0.18	GTPase NRas	P08556
Nrcam	-0.3404	-0.33	0.0030	0.31	0.0480	0.00	0.65	-0.02	0.3	Neuronal cell adhesion molecule	Q810U4
Nrd1	-0.4154	1.69	0.2	-0.86	0.39	-6.23	0.16	0.83	0.56	Nardilysin, N-arginine dibasic convertase, NRD convertase 1	A2A9Q2
Nrp1	-0.0008	0.88	0.84	-0.53	0.95	-0.55	0.69	0.35	0.78	Neuropilin-1	P97333
Nrxn1	-1.2303	-0.71	0.0430	0.90	0.0390	0.67	0.15	0.19	0.63	Neurexin-1	Q9CS84
Nrxn2	-0.0068	0.36	0.6	-0.79	0.78	2.75	0.29	-0.43	0.65	Neurexin-2	E9PUM9
Nrxn3	-0.1037	0.62	0.0079	-0.10	0.16	0.12	0.68	0.52	0.35	Neurexin-3	Q6P9K9
Nsf	-0.0286	0.08	0.13	-0.16	0.0030	-0.12	0.061	-0.08	0.099	Vesicle-fusing ATPase	P46460
Nsfl1c	0.0013	-0.22	0.51	-0.16	0.74	-0.13	0.61	-0.38	0.31	NSFL1 cofactor p47	Q9CZ44
Nsmf	-9.9579	2.25	0.0200	-1.33	0.0110	0.90	0.51	0.92	0.52	NMDA receptor synaptonuclear-signaling and neuronal migration factor	A2AJ92
Nt5dc3	-0.1433	0.50	0.43	-0.72	0.082	-0.28	0.89	-0.23	0.81	5'-nucleotidase domain-containing protein 3	Q3UHB1
Ntm	-1.9063	0.57	0.0170	-0.63	0.0010	0.12	0.66	-0.06	0.95	Neurotrimin	D3Z396
Ntrk3	-0.0873	0.87	0.099	-0.62	0.69	0.20	0.45	0.25	0.52	Tyrosine-protein kinase receptor	A0A0A6YWF9
Nudc	-0.0196	-0.28	0.0470	0.14	0.42	0.15	0.25	-0.14	0.42	Nuclear migration protein nudC	O35685
Nudcd3	0.0000	-0.01	1	-0.23	0.73	-0.27	0.47	-0.25	0.61	NudC domain-containing protein 3	Q8R1N4
Nudt16	-0.0020	0.01	0.64	-0.75	0.0430	0.24	0.6	-0.74	0.12	U8 snoRNA-decapping enzyme (Fragment)	A0A087WRE5
Nudt2	-1.0738	4.34	0.14	-2.99	0.8	5.07	0.28	1.35	0.47	Bis(5'-nucleosyl)-tetraphosphatase [asymmetrical]	P56380
Nudt5	-1.1209	3.33	0.74	-4.40	0.26	0.51	0.8	-1.07	0.8	ADP-sugar pyrophosphatase	Q9JKX6
Numb	0.0001	-0.13	0.69	-0.26	0.95	0.29	0.49	-0.39	0.93	Protein numb homolog	Q9QZS3
Numbl	0.0000	0.01	0.98	-0.20	0.2	0.05	0.94	-0.20	0.19	Numb-like protein	O08919
Nup93	-0.0001	-0.44	0.98	0.13	0.58	-0.38	0.25	-0.31	0.6	Nuclear pore complex protein Nup93	Q8BJ71
Nup98	-0.0038	-0.13	0.93	0.54	0.0200	-0.03	0.98	0.41	0.21	Nuclear pore complex protein Nup96	A0A1B0GSX7

Table S2: Differentially expressed proteins in cerebral cortex *(continued)*

gene	$\pi_{TW/EW} \times \pi_{TF/TW}$	log2FC TW/EW	padj TW/EW	log2FC TF/TW	padj TF/TW	log2FC EF/EW	padj EF/EW	log2FC TF/EW	padj TF/EW	description	accession
Nutf2	0.0108	-1.07	0.0280	-0.06	0.78	-0.22	0.27	-1.13	0.0280	Nuclear transport factor 2	P61971
Nwd2	0.0000	-0.06	0.85	-0.06	0.69	0.00	0.92	-0.12	0.53	NACHT and WD repeat domain-containing protein 2	Q6P5U7
Oat	-0.0047	-0.93	0.1	0.03	0.68	-0.50	0.33	-0.91	0.12	Ornithine aminotransferase, mitochondrial	P29758
Ociad1	-0.0011	-0.30	0.59	0.14	0.77	0.32	0.28	-0.16	0.73	OCIA domain-containing protein 1	Q9CRD0
Ociad2	-0.3337	-0.54	0.37	1.10	0.05	0.35	0.22	0.56	0.0300	OCIA domain-containing protein 2	Q9D8W7
Ocrl	-0.0420	-0.10	0.53	0.98	0.0280	0.09	0.62	0.89	0.0280	Inositol polyphosphate 5-phosphatase OCRL	Q6NVF0
Oga	0.0007	0.56	0.0450	0.10	0.98	-0.06	0.93	0.66	0.0120	Protein O-GlcNAcase	Q9EQQ9
Ogdh	-0.0360	-0.14	0.26	0.28	0.0270	0.13	0.66	0.13	0.26	2-oxoglutarate dehydrogenase, mitochondrial	Q60597
Ogdhl	-0.0004	-0.05	0.89	0.20	0.2	-0.07	0.25	0.16	0.25	Ogdhl protein	B2RXT3
Ogt	-0.3321	0.22	0.0120	-0.30	0.0024	0.01	0.64	-0.08	0.87	UDP-N-acetylglucosamine–peptide N-acetylglucosaminyltransferase 110 kDa subunit	Q8CGY8
Ola1	-0.0004	-0.07	0.89	0.15	0.2	-0.16	0.24	0.08	0.35	Obg-like ATPase 1	Q9CZ30
Olfm1	0.0000	-0.37	0.0008	0.00	0.8	0.23	1	-0.37	0.0010	Noelin	Q8R357
Olfm2	-0.0920	-0.38	0.0050	0.16	0.22	0.42	0.0001	-0.22	0.095	Noelin-2	A0A1L1SV46
Olfm3	-1.7573	0.63	0.0280	-0.72	0.0032	0.51	0.17	-0.10	0.65	Noelin-3	P63056
Omg	-0.0009	0.18	0.95	-0.59	0.4	-0.18	0.56	-0.41	0.26	Oligodendrocyte-myelin glycoprotein	G3XA53
Opa1	0.0094	0.17	0.58	0.50	0.34	-0.01	0.88	0.67	0.079	Dynamin-like 120 kDa protein, form S1	H7BX01
Opa3	0.2215	1.05	0.0120	0.21	0.3	0.21	0.87	1.26	0.0070	Optic atrophy 3 protein homolog	Q505D7
Opcml	-0.8103	0.39	0.0200	-0.51	0.0040	-0.24	0.28	-0.12	0.93	Opioid-binding protein/cell adhesion molecule-like	G5E8G3
Osbp	-0.1355	0.23	0.24	-1.36	0.2	-1.10	0.5	-1.14	0.46	Oxysterol-binding protein 1	Q3B7Z2
Osbpl6	0.0015	-0.07	0.79	-0.40	0.29	-0.05	0.92	-0.47	0.19	Oxysterol-binding protein (Fragment)	V9GXX3
Osbpl8	-0.0001	0.05	0.97	-0.94	0.64	-2.25	0.34	-0.89	0.68	Oxysterol-binding protein	A0A0R4J150
Oxct1	0.0081	-0.11	0.68	-0.34	0.05	0.06	0.83	-0.45	0.0070	Succinyl-CoA:3-ketoacid coenzyme A transferase 1, mitochondrial	Q9D0K2
Oxr1	0.0004	-0.03	0.91	-0.24	0.0460	0.24	0.82	-0.27	0.0190	Oxidation resistance protein 1	Q4KMM3
Oxsm	0.0266	-0.12	0.59	-1.05	0.12	-0.26	0.41	-1.17	0.0067	3-oxoacyl-[acyl-carrier-protein] synthase, mitochondrial	Q9D404
Oxsr1	-0.8930	0.26	0.32	-5.51	0.055	-4.91	0.1	-5.25	0.11	Serine/threonine-protein kinase OSR1	Q6P9R2
P4hb	-0.0799	-0.49	0.26	0.49	0.27	0.25	0.54	0.00	1	Protein disulfide-isomerase	P09103
Pa2g4	-0.0127	0.17	0.19	-0.13	0.16	0.04	0.91	0.04	0.68	Proliferation-associated protein 2G4	P50580
Pabpc1	-0.0583	0.80	0.0054	-0.12	0.54	0.07	0.82	0.68	0.11	Polyadenylate-binding protein 1	P29341
Pacs1	0.0004	0.12	0.86	0.18	0.52	0.21	0.5	0.30	0.33	Phosphofurin acidic cluster sorting protein 1	Q8K212

Table S2: Differentially expressed proteins in cerebral cortex *(continued)*

gene	$\pi_{TW/EW}\times$ $\pi_{TF/TW}$	log2FC TW/EW	padj TW/EW	log2FC TF/TW	padj TF/TW	log2FC EF/EW	padj EF/EW	log2FC TF/EW	padj TF/EW	description	accession
Pacsin1	-0.0090	-0.01	0.34	0.63	0.0009	-0.12	0.33	0.62	0.0010	Protein kinase C and casein kinase substrate in neurons protein 1	Q61644
Pacsin2	0.0650	0.13	0.089	0.66	0.19	-0.29	0.17	0.78	0.0001	Protein kinase C and casein kinase substrate in neurons protein 2	Q9WVE8
Pacsin3	-0.0001	-0.06	0.7	0.06	0.6	-0.46	0.56	0.00	1	Protein kinase C and casein kinase II substrate protein 3	Q99JB8
Padi2	-0.0436	-0.74	0.089	0.27	0.62	-1.20	0.082	-0.47	0.12	Protein-arginine deiminase type-2	Q08642
Pafah1b1	-0.0001	-0.05	0.82	0.08	0.66	-0.32	0.15	0.03	0.81	Platelet-activating factor acetylhydrolase IB subunit beta	P63005
Pafah1b2	-0.0171	-0.75	0.0280	0.06	0.57	-0.04	0.93	-0.68	0.0280	Platelet-activating factor acetylhydrolase IB subunit alpha2 (Fragment)	A0A1L1SVK0
Pafah1b3	-0.0141	1.08	0.67	-0.93	0.83	0.23	0.66	0.15	0.64	Platelet-activating factor acetylhydrolase IB subunit alpha1	Q61205
Paics	0.1991	-0.63	0.67	-1.64	0.078	-0.84	0.67	-2.28	0.0060	Multifunctional protein ADE2	Q9DCL9
Pak1	-0.0004	-0.24	0.095	0.18	0.98	-0.09	0.42	-0.06	0.37	Serine/threonine-protein kinase PAK 1	O88643
Pak5	-0.0051	0.26	0.73	-0.48	0.5	0.51	0.32	-0.22	0.45	Serine/threonine-protein kinase PAK 5	Q8C015
Palm	0.0002	-0.03	0.21	-0.07	0.72	0.44	0.11	-0.10	0.44	Paralemmin-1	Q9Z0P4
Palm2	-2.2082	0.62	0.089	-1.13	0.0010	0.50	0.95	-0.50	0.14	Paralemmin-2	Q8BR92
Pam16	0.0000	0.01	1	0.06	0.6	0.50	0.0280	0.08	0.78	Mitochondrial import inner membrane translocase subunit TIM16	Q9CQV1
Park7	-0.2772	-0.46	0.0001	0.17	0.13	-0.43	0.0002	-0.29	0.0008	Parkinson disease protein 7 homolog	Q99LX0
Pcbd2	-0.0055	0.39	0.81	-0.82	0.65	-0.45	0.97	-0.42	0.66	4a-hydroxytetrahydrobiopterin dehydratase	A0A0R4J1R7
Pcbp1	-0.0005	-0.12	0.9	0.39	0.57	-0.21	0.59	0.27	0.57	Poly(rC)-binding protein 1	P60335
Pcca	0.0000	0.03	0.48	0.14	0.94	-0.21	0.22	0.17	0.97	Propionyl-CoA carboxylase alpha chain, mitochondrial	Q91ZA3
Pccb	0.0015	0.29	0.51	0.14	0.75	-0.80	0.4	0.43	0.32	Propionyl-CoA carboxylase beta chain, mitochondrial	E9Q1J7
Pcdh1	0.0000	-0.06	0.97	0.17	0.62	-0.08	0.89	0.10	0.61	Protocadherin 1	Q8CFX3
Pcdh10	0.0019	-0.18	0.35	-0.11	0.62	0.28	0.52	-0.29	0.16	Protocadherin 10	E9PX28
Pcdh19	-12.5675	1.50	0.0080	-2.04	0.0110	0.74	0.39	-0.53	0.68	Protocadherin-19	E9Q5E1
Pcdh7	-0.0004	0.19	0.64	-0.03	0.45	0.51	0.49	0.16	0.95	Protocadherin 7	A0A0A6YY83
Pcdh8	0.0046	-0.19	0.67	-3.06	0.9	-2.99	0.83	-3.25	0.77	Protocadherin-8	Q7TSK3
Pcdh9	0.0000	0.01	0.96	0.14	0.18	0.07	0.36	0.15	0.0130	Protocadherin 9	A0A0A6YWY8
Pcdhgb5	-4.0490	1.46	0.13	-5.35	0.26	0.08	0.73	-3.90	0.7	Protocadherin gamma B5	Q91XX5
Pcdhgc5	-0.0006	0.37	0.77	-0.37	0.92	-0.43	0.48	0.00	0.75	Pcdhgc5 protein	Q91XW9
Pclo	-21.2808	1.12	0.0020	-1.76	0.0001	-0.17	0.0260	-0.64	0.0001	Protein piccolo	Q9QYX7
Pcmt1	-0.0041	-0.47	0.0020	0.04	0.83	-0.22	0.26	-0.43	0.0005	Protein-L-isoaspartate O-methyltransferase	E0CYV0

Table S2: Differentially expressed proteins in cerebral cortex *(continued)*

gene	$\pi_{TW/EW}\times$ $\pi_{TF/TW}$	log2FC TW/EW	padj TW/EW	log2FC TF/TW	padj TF/TW	log2FC EF/EW	padj EF/EW	log2FC TF/EW	padj TF/EW	description	accession
Pcnp	-0.0078	0.91	0.71	-0.76	0.84	1.82	0.23	0.15	0.64	PEST proteolytic signal-containing nuclear protein	F8WI88
Pcsk1	-0.0089	0.12	0.73	-0.52	0.091	-0.69	0.22	-0.40	0.25	Neuroendocrine convertase 1	P63239
Pcsk1n	-0.0004	-0.40	0.88	0.04	0.36	-0.15	0.91	-0.36	0.48	ProSAAS	Q9QXV0
Pcx	-0.0051	0.09	0.44	-0.24	0.22	0.19	0.56	-0.15	0.72	Pyruvate carboxylase	G5E8R3
Pcyt2	0.0345	4.83	0.39	0.14	0.75	5.22	0.37	4.97	0.38	Ethanolamine-phosphate cytidylyltransferase	A2ABY3
Pdap1	-0.1654	-1.02	0.0056	0.43	0.68	-0.37	0.45	-0.59	0.25	28 kDa heat- and acid-stable phosphoprotein	Q3UHX2
Pdcd10	-0.0125	0.30	0.46	-0.27	0.35	0.19	0.63	0.03	0.76	Programmed cell death protein 10	Q8VE70
Pdcd5	0.0091	-0.11	0.54	-0.39	0.16	-0.01	0.74	-0.50	0.49	Programmed cell death protein 5	P56812
Pdcd6ip	-0.0001	0.02	0.82	-0.08	0.4	-0.03	0.49	-0.06	0.53	Programmed cell death 6-interacting protein	Q9WU78
Pdcl	-0.0374	-0.73	0.43	0.63	0.6	-1.60	0.38	-0.10	0.89	Phosducin-like protein	Q9DBX2
Pde10a	0.0050	0.92	0.38	0.14	0.81	0.94	0.11	1.06	0.29	Phosphodiesterase	A0A384DV92
Pde1a	0.0076	0.19	0.34	0.22	0.41	0.15	0.44	0.41	0.27	Phosphodiesterase	A2ASF9
Pde4d	-0.0064	0.22	0.74	-0.59	0.42	-0.29	0.14	-0.38	0.055	Phosphodiesterase (Fragment)	F6QFD1
Pde9a	0.0000	-0.49	0.11	0.27	1	-0.17	0.55	-0.22	0.38	High affinity cGMP-specific 3',5'-cyclic phosphodiesterase 9A	A0A494BAI4
Pdha1	-0.0017	-0.06	0.17	0.07	0.29	-0.05	1	0.01	0.89	Pyruvate dehydrogenase E1 component subunit alpha, somatic form, mitochondrial	P35486
Pdhb	-0.0011	-0.10	0.55	0.12	0.43	-0.17	0.0030	0.02	0.27	Pyruvate dehydrogenase E1 component subunit beta, mitochondrial	Q9D051
Pdhx	0.0003	-0.14	0.93	-0.09	0.2	-0.37	0.081	-0.23	0.31	Pyruvate dehydrogenase protein X component, mitochondrial	Q8BKZ9
Pdia3	0.0000	0.00	0.99	0.13	0.15	-0.17	0.0120	0.13	0.12	Protein disulfide-isomerase A3	P27773
Pdia4	0.0000	-0.10	0.38	0.02	0.94	-0.12	0.49	-0.08	0.66	Protein disulfide-isomerase A4	A0A0R4J0Z1
Pdia6	0.1832	0.17	0.21	0.53	0.0010	-0.05	0.48	0.69	0.0030	Protein disulfide-isomerase A6	F7DBQ0
Pdk1	0.0067	0.60	0.35	0.14	0.67	0.22	0.91	0.74	0.24	[Pyruvate dehydrogenase (acetyl-transferring)] kinase isozyme 1, mitochondrial	Q8BFP9
Pdk2	0.0006	0.06	0.65	0.16	0.44	-0.21	0.31	0.21	0.27	[Pyruvate dehydrogenase (acetyl-transferring)] kinase isozyme 2, mitochondrial	Q9JK42
Pdk3	-0.0106	0.51	0.27	-0.15	0.57	0.00	0.79	0.35	0.66	[Pyruvate dehydrogenase (acetyl-transferring)] kinase isozyme 3, mitochondrial	Q922H2
Pdlim5	-7.1519	6.04	0.0280	-0.59	0.051	5.39	0.12	5.45	0.11	ENH isoform 1d	D9J301

Table S2: Differentially expressed proteins in cerebral cortex *(continued)*

gene	$\pi_{TW/EW} \times \pi_{TF/TW}$	log2FC TW/EW	padj TW/EW	log2FC TF/TW	padj TF/TW	log2FC EF/EW	padj EF/EW	log2FC TF/EW	padj TF/EW	description	accession
Pdpk1	0.0000	-0.12	0.98	0.09	0.93	0.10	0.27	-0.03	0.88	3-phosphoinositide-dependent protein kinase 1	F2Z3X6
Pdxdc1	0.0034	-0.33	0.42	-0.34	0.83	0.01	0.86	-0.67	0.23	Pyridoxal-dependent decarboxylase domain-containing protein 1	A0A0R4J034
Pdxk	-0.0035	-0.41	0.0270	0.09	0.87	-0.22	0.26	-0.31	0.065	Pyridoxal kinase	Q8K183
Pdxp	0.0000	-0.16	0.41	0.00	1	-0.88	0.65	-0.16	0.51	Pyridoxal phosphate phosphatase	P60487
Pebp1	-0.0157	0.14	0.61	-0.68	0.17	-0.15	0.94	-0.53	0.17	Phosphatidylethanolamine-binding protein 1	P70296
Pelo	0.0000	-0.20	0.63	-0.03	0.98	0.26	0.67	-0.23	0.57	Protein pelota homolog	Q80X73
Pfdn1	-0.0035	-0.20	0.35	0.14	0.53	-0.15	0.37	-0.06	0.69	Prefoldin 1	Q9CQF7
Pfdn2	-0.0033	-0.45	0.0040	0.17	0.96	-0.07	0.92	-0.28	0.14	Prefoldin subunit 2	O70591
Pfdn4	-0.1808	-0.53	0.0280	0.17	0.051	0.13	0.43	-0.36	0.055	Prefoldin 4	Q6P0X1
Pfkl	0.0021	0.04	0.78	0.38	0.056	0.13	0.76	0.42	0.068	ATP-dependent 6-phosphofructokinase, liver type	P12382
Pfkm	0.0092	0.53	0.0170	0.44	0.95	0.46	0.14	0.97	0.0005	ATP-dependent 6-phosphofructokinase, muscle type	P47857
Pfkp	-0.1094	0.37	0.0001	-0.13	0.27	0.10	0.28	0.25	0.0004	ATP-dependent 6-phosphofructokinase	Q8C605
Pfn1	0.0203	-0.23	0.071	-0.21	0.43	-0.58	0.0390	-0.44	0.0270	Profilin-1	P62962
Pfn2	-1.0014	0.44	0.083	-0.67	0.0007	0.16	0.56	-0.23	0.32	Profilin-2	Q9JJV2
Pgam1	-0.0002	-0.30	0.99	0.28	0.32	0.22	0.73	-0.02	0.99	Phosphoglycerate mutase 1	Q9DBJ1
Pgam5	-0.0066	0.42	0.0029	-0.04	0.7	0.39	0.15	0.37	0.23	Serine/threonine-protein phosphatase PGAM5, mitochondrial	Q8BX10
Pgap1	-0.0660	1.18	0.14	-0.35	0.65	0.61	0.14	0.83	0.24	GPI inositol-deacylase	Q3UUQ7
Pgbd5	-0.9667	0.66	0.075	-0.83	0.0270	-0.09	0.94	-0.17	0.67	PiggyBac transposable element-derived protein 5	D3YZI9
Pgd	-0.0364	-0.22	0.62	1.25	0.23	0.47	0.7	1.04	0.39	6-phosphogluconate dehydrogenase, decarboxylating	Q9DCD0
Pgk1	0.0253	-0.50	0.0003	-0.12	0.76	-0.36	0.08	-0.62	0.0003	Phosphoglycerate kinase 1	P09411
Pgls	-0.0081	0.25	0.69	-0.65	0.49	-0.70	0.46	-0.39	0.66	6-phosphogluconolactonase	Q9CQ60
Pgm1	-0.0153	-0.58	0.0280	0.11	0.7	-0.48	0.055	-0.47	0.0280	Phosphoglucomutase-1	Q9D0F9
Pgm2l1	0.0022	-0.10	0.65	-0.15	0.17	-0.12	0.34	-0.25	0.12	Glucose 1,6-bisphosphate synthase	Q8CAA7
Pgp	0.0121	-0.27	0.0190	-0.10	0.55	-0.43	0.0150	-0.37	0.0200	Glycerol-3-phosphate phosphatase	Q8CHP8
Pgrmc1	0.0001	-0.26	0.7	-0.11	0.96	0.03	0.48	-0.37	0.68	Membrane-associated progesterone receptor component 1	O55022
Pgrmc2	-0.0150	-0.45	0.0010	0.05	0.6	-0.37	0.13	-0.40	0.0010	Membrane-associated progesterone receptor component 2	Q80UU9
Phactr1	-0.1444	0.65	0.14	-0.75	0.45	0.06	0.67	-0.10	0.23	Phosphatase and actin regulator 1	Q2M3X8
Phb	-0.0001	-0.02	0.7	0.28	0.75	-0.18	0.22	0.26	0.96	Prohibitin	P67778
Phb2	0.0009	0.04	0.65	0.17	0.18	0.03	0.88	0.21	0.38	Prohibitin-2	O35129

Table S2: Differentially expressed proteins in cerebral cortex *(continued)*

gene	$\pi_{TW/EW} \times \pi_{TF/TW}$	log2FC TW/EW	padj TW/EW	log2FC TF/TW	padj TF/TW	log2FC EF/EW	padj EF/EW	log2FC TF/EW	padj TF/EW	description	accession
Phf24	-0.0041	0.10	0.76	-0.39	0.13	0.08	0.73	-0.29	0.2	PHD finger protein 24	Q80TL4
Phgdh	-0.0044	-0.06	0.9	0.45	0.0003	-0.12	0.53	0.40	0.0008	D-3-phosphoglycerate dehydrogenase	Q61753
Phyhip	-0.0010	-0.03	0.83	0.33	0.054	-0.90	0.24	0.30	0.053	Phytanoyl-CoA hydroxylase-interacting protein	Q8K0S0
Pi4ka	-0.0188	0.36	0.0010	-0.01	0.0180	-0.03	0.67	0.35	0.53	Phosphatidylinositol 4-kinase alpha	E9Q3L2
Picalm	0.0554	0.15	0.18	0.56	0.13	-0.04	0.91	0.71	0.0070	Phosphatidylinositol-binding clathrin assembly protein	Q7M6Y3
Pick1	0.0077	0.55	0.88	0.51	0.32	0.85	0.59	1.05	0.41	PRKCA-binding protein	E9PUZ5
Pigs	0.0630	0.16	0.43	0.82	0.0490	-0.29	0.74	0.98	0.0280	GPI transamidase component PIG-S	Q6PD26
Pik3c3	0.0091	0.50	0.54	0.35	0.64	0.03	0.95	0.85	0.16	Phosphatidylinositol 3-kinase catalytic subunit type 3	E9QLS6
Pik3r1	-0.0001	0.08	0.86	-0.17	0.75	-0.13	0.92	-0.10	0.83	Phosphatidylinositol 3-kinase regulatory subunit alpha	P26450
Pik3r4	-0.3432	1.32	0.0010	-0.18	0.33	-0.50	0.66	1.15	0.0025	Phosphoinositide 3-kinase regulatory subunit 4	Q8VD65
Pin1	0.0001	-0.10	0.37	-0.08	0.93	-0.08	0.95	-0.17	0.15	Peptidyl-prolyl cis-trans isomerase NIMA-interacting 1	Q9QUR7
Pip4k2b	-0.0009	-0.09	0.44	0.05	0.27	0.21	0.75	-0.04	0.68	Phosphatidylinositol 5-phosphate 4-kinase type-2 beta	Q80XI4
Pip4k2c	-0.0022	-0.05	0.39	0.36	0.5	-0.08	0.66	0.31	0.12	Phosphatidylinositol 5-phosphate 4-kinase type-2 gamma	Q91XU3
Pip5k1c	-0.2701	0.55	0.14	-0.60	0.11	0.07	0.67	-0.05	0.9	Phosphatidylinositol 4-phosphate 5-kinase type-1 gamma	F8WHW6
Pisd	-0.7485	0.67	0.097	-0.71	0.0280	-0.23	0.36	-0.05	0.69	Phosphatidylserine decarboxylase proenzyme, mitochondrial	E9PX91
Pithd1	0.0001	0.27	0.58	0.04	0.93	-0.02	0.8	0.31	0.53	PITH domain-containing protein 1	Q8BWR2
Pitpnm1	-0.2051	0.50	0.22	-0.58	0.084	-0.54	0.13	-0.08	0.4	Membrane-associated phosphatidylinositol transfer protein 1	O35954
Pitpnm2	-1.8536	1.61	0.0430	-1.32	0.23	-0.01	0.5	0.29	0.26	Membrane-associated phosphatidylinositol transfer protein 2	Q6ZPQ6
Pitrm1	-0.1881	0.42	0.0190	-0.25	0.091	0.16	0.53	0.17	0.44	Presequence protease, mitochondrial	Q8K411
Pkm	-0.0009	-0.24	0.6	0.02	0.14	-0.13	0.25	-0.22	0.38	Pyruvate kinase PKM	P52480
Pkp2	-0.1003	0.88	0.64	-1.40	0.38	-0.39	0.58	-0.53	0.44	Plakophilin 2	Q9CQ73
Pkp4	-37.0053	2.31	0.0120	-2.78	0.0010	0.15	0.94	-0.47	0.23	Plakophilin-4	Q68FH0
Plaa	-0.0015	0.11	0.81	-0.33	0.36	-0.14	0.83	-0.21	0.69	Phospholipase A-2-activating protein	P27612
Plcb1	0.0000	-0.02	0.91	-0.01	0.93	0.08	0.55	-0.03	0.9	1-phosphatidylinositol 4,5-bisphosphate phosphodiesterase beta-1	Q9Z1B3
Plcg1	0.0003	0.59	0.39	0.01	0.72	0.25	0.35	0.60	0.0440	1-phosphatidylinositol 4,5-bisphosphate phosphodiesterase gamma-1	Q62077

Table S2: Differentially expressed proteins in cerebral cortex *(continued)*

gene	$\pi_{TW/EW} \times \pi_{TF/TW}$	log2FC TW/EW	padj TW/EW	log2FC TF/TW	padj TF/TW	log2FC EF/EW	padj EF/EW	log2FC TF/EW	padj TF/EW	description	accession
Plch2	0.0001	-0.03	0.79	-0.34	0.88	-0.13	0.93	-0.37	0.76	1-phosphatidylinositol 4,5-bisphosphate phosphodiesterase eta-2	A2AP18
Plcl2	-0.0066	0.18	0.7	-0.37	0.23	0.02	0.89	-0.20	0.28	Inactive phospholipase C-like protein 2	Q8K394
Plcxd2	-0.8156	-3.62	0.5	3.73	0.63	0.89	0.73	0.11	1	PI-PLC X domain-containing protein 2	B2RXA1
Pld3	0.0096	0.13	0.5	0.71	0.45	0.29	0.66	0.83	0.19	5'-3' exonuclease PLD3	O35405
Plec	-36.1200	1.29	0.0001	-1.75	0.0001	0.57	0.0001	-0.46	0.0016	Plectin	E9Q3W4
Plekha5	-2.3325	1.88	0.45	-4.19	0.14	-1.52	0.33	-2.30	0.21	Pleckstrin homology domain-containing, family A member 5 (Fragment)	A0A0N4SWD7
Plekha6	-13.5100	4.97	0.34	-9.09	0.23	4.03	0.24	-4.12	0.43	Pleckstrin homology domain-containing family A member 6	A0A087WRB7
Plekhm1	0.0000	0.34	0.84	-0.05	0.94	0.09	0.64	0.29	0.72	Pleckstrin homology domain-containing family M member 1	Q7TSI1
Plg	0.0007	-0.67	0.55	-0.02	0.65	0.42	0.65	-0.69	0.53	Plasminogen	P20918
Plin4	-0.0001	0.05	0.96	-0.30	0.41	-0.22	0.66	-0.25	0.42	Perilipin-4	A0A571BEC9
Plp1	0.3467	-0.28	0.27	-0.55	0.0001	-0.34	0.0410	-0.82	0.0001	Myelin proteolipid protein	P60202
Plpbp	-0.0011	-0.04	0.48	0.12	0.18	-0.08	0.27	0.07	0.45	Pyridoxal phosphate homeostasis protein (Fragment)	A0A1B0GRP7
Plpp3	0.0000	-0.18	1	0.06	0.78	0.11	0.94	-0.12	0.77	Phospholipid phosphatase 3	Q99JY8
Plppr3	0.0000	0.01	0.96	-0.25	0.71	-0.17	0.88	-0.24	0.71	Phospholipid phosphatase-related protein type 3	A0A0R3P9D0
Plppr4	-0.4023	0.42	0.052	-0.45	0.0220	-0.09	0.9	-0.03	0.94	2-lysophosphatidate phosphatase PLPPR4 (Fragment)	A0A0G2JGY9
Pls3	0.1383	-0.65	0.079	-0.33	0.26	-0.94	0.084	-0.99	0.0280	Plastin-3	B1AX58
Plxna1	-0.0055	-0.04	0.83	0.63	0.0020	-0.30	0.24	0.59	0.0007	Plexin-A1	P70206
Plxna4	-0.0385	-0.26	0.15	0.44	0.39	-0.25	0.23	0.18	0.64	Plexin-A4	Q80UG2
Plxnb1	-0.0388	-0.20	0.4	0.44	0.078	0.25	0.31	0.25	0.33	Plexin-B1	Q8CJH3
Plxnb2	0.1308	-0.61	0.36	-0.85	0.27	-0.20	0.61	-1.45	0.12	Plexin-B2	B2RXS4
Plxnc1	-0.0881	-0.23	0.14	0.34	0.0480	-0.41	0.25	0.11	0.33	Plexin-C1	Q9QZC2
Plxnd1	-0.0259	-0.37	0.16	0.31	0.52	-0.15	0.32	-0.06	0.88	Plexin-D1	Q3UH93
Pmm1	0.1088	-0.35	0.78	-4.65	0.24	-3.54	0.57	-5.00	0.25	Phosphomannomutase 1	O35621
Pmpca	-0.0045	-0.49	0.17	0.11	0.78	-0.08	0.98	-0.39	0.072	Mitochondrial-processing peptidase subunit alpha	Q9DC61
Pmpcb	0.0000	-0.01	0.95	0.03	0.83	-0.21	0.8	0.02	0.83	Mitochondrial-processing peptidase subunit beta	Q9CXT8
Pnn	-0.0023	-0.33	0.81	0.50	0.7	-0.41	0.37	0.17	0.63	Pinin	Q3TUQ5
Pnpla8	0.0123	0.09	0.46	0.25	0.0240	0.19	0.97	0.34	0.0008	Calcium-independent phospholipase A2-gamma	Q8K1N1
Pnpt1	0.0523	0.12	0.15	0.39	0.0440	0.29	0.12	0.50	0.0007	Polyribonucleotide nucleotidyltransferase 1, mitochondrial	Q8K1R3

Table S2: Differentially expressed proteins in cerebral cortex *(continued)*

gene	$\pi_{TW/EW} \times \pi_{TF/TW}$	log2FC TW/EW	padj TW/EW	log2FC TF/TW	padj TF/TW	log2FC EF/EW	padj EF/EW	log2FC TF/EW	padj TF/EW	description	accession
Podn	0.0115	-0.19	0.72	-1.33	0.48	-1.49	0.054	-1.51	0.16	Podn protein (Fragment)	Q6P3D8
Poglut1	0.0001	0.05	0.34	0.03	0.68	0.29	0.3	0.08	0.71	Protein O-glucosyltransferase 1	Q8BYB9
Poldip3	-1.0026	1.09	0.0390	-0.63	0.092	0.41	0.51	0.45	0.42	Polymerase delta-interacting protein 3	Q8BG81
Polr2a	-0.0026	-0.29	0.42	0.14	0.68	0.10	0.85	-0.15	0.57	DNA-directed RNA polymerase subunit	A0A0R4J0V5
Pomgnt2	-6.6063	1.13	0.0010	-0.71	0.0018	-0.37	0.71	0.43	0.5	Protein O-linked-mannose beta-1,4-N-acetylglucosaminyltransferase 2	Q8BW41
Por	-0.4020	0.64	0.17	-0.66	0.058	-4.54	0.0330	-0.02	0.88	NADPH–hemoprotein reductase	Q05DV1
Ppa1	-0.0002	-0.19	0.71	0.13	0.91	0.00	0.84	-0.06	0.8	Inorganic pyrophosphatase	Q9D819
Ppfia1	-3.4624	3.36	0.41	-4.68	0.27	-1.24	0.56	-1.32	0.5	Ppfia1 protein	B2RXQ2
Ppfia2	-2.3608	0.90	0.34	-2.67	0.0080	-0.65	0.45	-1.77	0.0200	Liprin-alpha-2	B8QI34
Ppfia3	-0.1592	0.38	0.24	-0.61	0.078	-0.20	0.52	-0.23	0.69	Liprin-alpha-3	P60469
Ppfia4	0.0000	0.12	0.89	-0.02	0.88	-0.34	0.51	0.09	0.83	Liprin-alpha 4	B8QI36
Ppia	-0.0191	0.05	0.45	-0.46	0.0040	-0.30	0.086	-0.41	0.0250	Peptidyl-prolyl cis-trans isomerase A	P17742
Ppid	0.0006	-0.23	0.92	-0.14	0.29	-0.06	0.67	-0.37	0.26	Peptidyl-prolyl cis-trans isomerase D	Q9CR16
Ppif	0.0027	-0.22	0.93	-0.60	0.22	-0.19	0.76	-0.83	0.24	Peptidyl-prolyl cis-trans isomerase F, mitochondrial	Q99KR7
Ppip5k1	0.0003	0.27	0.45	0.05	0.87	0.04	0.92	0.32	0.28	Inositol hexakisphosphate and diphosphoinositol-pentakisphosphate kinase 1	A2ARP1
Ppm1a	0.0000	0.17	0.9	-0.07	0.81	0.59	0.53	0.10	0.7	Protein phosphatase 1A	P49443
Ppm1b	0.0003	-0.20	0.33	-0.06	0.87	0.16	0.63	-0.26	0.35	Protein-serine/threonine phosphatase	Q99NF7
Ppm1e	0.0340	-0.72	0.13	-0.24	0.6	-0.36	0.21	-0.96	0.055	Protein phosphatase 1E	Q80TL0
Ppm1f	-0.0141	-0.21	0.21	0.23	0.37	-0.21	0.21	0.02	0.91	Protein phosphatase 1F	Q8CGA0
Ppm1g	-0.1793	-0.24	0.32	0.72	0.0080	-0.12	0.84	0.48	0.051	Protein phosphatase 1G	A0A0J9YVG0
Ppm1h	-0.0183	0.17	0.41	-0.29	0.11	0.31	0.2	-0.12	0.61	Protein phosphatase 1H	Q3UYC0
Ppme1	0.0007	-0.26	0.94	-0.21	0.31	-0.28	0.091	-0.47	0.21	Protein phosphatase methylesterase 1	Q8BVQ5
Ppp1ca	-0.0469	0.31	0.44	-0.57	0.18	-0.01	0.7	-0.26	0.77	Serine/threonine-protein phosphatase PP1-alpha catalytic subunit	P62137
Ppp1cc	0.0099	-0.72	0.2	-0.15	0.74	0.01	0.96	-0.87	0.061	Serine/threonine-protein phosphatase (Fragment)	A0A0G2JFF1
Ppp1r12a	-0.0754	0.73	0.31	-0.47	0.37	0.61	0.28	0.26	0.88	Protein phosphatase 1 regulatory subunit 12A	Q9DBR7
Ppp1r12c	-0.0829	1.13	0.092	-0.21	0.46	0.70	0.28	0.93	0.16	Protein phosphatase 1 regulatory subunit 12C	Q3UMT1
Ppp1r13b	-0.4346	0.69	0.0280	-0.32	0.054	-0.11	0.67	0.37	0.15	Apoptosis-stimulating of p53 protein 1 (Fragment)	A0A1Y7VJH3
Ppp1r1b	-0.0147	-0.66	0.75	0.73	0.57	0.30	0.73	0.07	0.93	Protein phosphatase 1 regulatory subunit 1B	Q60829

Table S2: Differentially expressed proteins in cerebral cortex *(continued)*

gene	$\pi_{TW/EW} \times \pi_{TF/TW}$	log2FC TW/EW	padj TW/EW	log2FC TF/TW	padj TF/TW	log2FC EF/EW	padj EF/EW	log2FC TF/EW	padj TF/EW	description	accession
Ppp1r21	-0.0001	-0.14	0.42	0.10	0.97	-0.05	0.86	-0.03	0.56	Protein phosphatase 1 regulatory subunit 21	Q3TDD9
Ppp1r7	-0.0109	-0.40	0.0030	0.06	0.66	-0.09	0.42	-0.34	0.0200	Protein phosphatase 1 regulatory subunit 7	Q3UM45
Ppp1r9a	-1.9345	0.59	0.14	-1.28	0.0010	0.81	0.1	-0.70	0.085	Protein phosphatase 1, regulatory subunit 9A	H3BJD6
Ppp1r9b	-9.3231	1.11	0.0130	-1.65	0.0020	0.96	0.0390	-0.54	0.72	Neurabin-2	Q6R891
Ppp2ca	-0.0897	-0.37	0.0140	0.17	0.17	0.02	0.95	-0.20	0.087	Serine/threonine-protein phosphatase 2A catalytic subunit alpha isoform	P63330
Ppp2r1a	-0.1118	-0.30	0.059	0.23	0.0480	0.16	0.95	-0.07	0.82	Serine/threonine-protein phosphatase 2A 65 kDa regulatory subunit A alpha isoform	Q76MZ3
Ppp2r2a	-0.0436	0.49	0.069	-0.27	0.52	-0.12	0.99	0.22	0.59	Serine/threonine-protein phosphatase 2A 55 kDa regulatory subunit B alpha isoform	Q6P1F6
Ppp2r5a	0.0560	-0.28	0.41	-1.23	0.38	0.04	0.98	-1.51	0.11	Serine/threonine-protein phosphatase 2A 56 kDa regulatory subunit alpha isoform	Q6PD03
Ppp2r5b	-0.0008	0.05	0.88	-0.51	0.28	0.08	0.81	-0.46	0.29	Serine/threonine-protein phosphatase 2A 56 kDa regulatory subunit beta isoform	Q6PD28
Ppp2r5c	0.0000	0.17	1	-0.14	0.98	-0.91	0.12	0.03	0.89	Serine/threonine-protein phosphatase 2A 56 kDa regulatory subunit gamma isoform	Q60996
Ppp2r5d	0.0429	-0.66	0.41	-0.33	0.31	-0.12	0.66	-0.99	0.088	Serine/threonine-protein phosphatase 2A 56 kDa regulatory subunit	Q7TNL5
Ppp2r5e	0.0220	-0.09	0.47	-0.46	0.0240	-0.34	0.0310	-0.55	0.0011	Serine/threonine-protein phosphatase 2A 56 kDa regulatory subunit	Q3UZJ4
Ppp3ca	0.0000	-0.15	0.95	0.03	0.57	0.03	0.65	-0.13	0.56	Serine/threonine-protein phosphatase 2B catalytic subunit alpha isoform	P63328
Ppp5c	-0.5650	-0.66	0.0011	0.44	0.22	-0.42	0.16	-0.22	0.54	Serine/threonine-protein phosphatase (Fragment)	F7BX26
Ppp6c	0.0001	0.15	0.77	0.10	0.93	0.61	0.23	0.25	0.65	Serine/threonine-protein phosphatase	A0A0N4SVL9
Ppt1	0.0000	-0.07	0.72	0.01	1	0.17	0.17	-0.06	0.73	Palmitoyl-protein thioesterase 1	O88531
Praf2	0.0007	0.14	0.49	0.08	0.63	-0.84	0.24	0.23	0.22	PRA1 family protein 2	Q9JIG8
Prdx1	0.0001	0.03	0.54	0.12	0.77	0.27	0.33	0.15	0.68	Peroxiredoxin-1 (Fragment)	B1AXW5
Prdx2	0.0000	0.07	0.42	0.08	0.97	0.19	0.38	0.15	0.41	Peroxiredoxin-2	Q61171
Prdx3	0.0008	-0.15	0.7	-0.08	0.38	-0.03	0.74	-0.23	0.24	Thioredoxin-dependent peroxide reductase, mitochondrial	P20108
Prdx5	-0.0153	0.10	0.26	-0.18	0.0350	-0.07	0.089	-0.09	0.23	Peroxiredoxin-5	H3BJQ7
Prdx6	-0.0491	-0.51	0.0010	0.09	0.44	-1.07	0.0004	-0.43	0.062	Peroxiredoxin-6	D3Z0Y2
Preb	-3.0469	2.20	0.094	-1.87	0.19	0.14	0.84	0.33	0.56	Prolactin regulatory element-binding protein	D3Z3S1
Prepl	-0.0207	0.24	0.61	-0.79	0.31	-1.40	0.34	-0.55	0.6	Prolyl endopeptidase-like	Q8C167

Table S2: Differentially expressed proteins in cerebral cortex *(continued)*

gene	$\pi_{TW/EW} \times \pi_{TF/TW}$	log2FC TW/EW	padj TW/EW	log2FC TF/TW	padj TF/TW	log2FC EF/EW	padj EF/EW	log2FC TF/EW	padj TF/EW	description	accession
Prex1	-0.0001	0.14	0.64	-0.05	0.87	-0.22	0.56	0.08	0.83	Phosphatidylinositol 3,4,5-trisphosphate-dependent Rac exchanger 1 protein	Q69ZK0
Prickle2	-0.4340	1.01	0.38	-1.65	0.24	-0.87	0.24	-0.64	0.6	Prickle-like 2 (Drosophila)	A7YQ68
Prkaa1	0.0119	-0.30	0.65	-3.25	0.86	-3.37	0.56	-3.55	0.69	Acetyl-CoA carboxylase kinase	Q3TUQ7
Prkacb	0.0034	-0.12	0.38	-0.16	0.38	0.45	0.11	-0.28	0.17	cAMP-dependent protein kinase catalytic subunit beta	P68181
Prkar1a	0.0017	-0.08	0.7	-0.21	0.22	0.17	0.29	-0.29	0.0490	cAMP-dependent protein kinase type I-alpha regulatory subunit	Q9DBC7
Prkar2a	0.0001	0.03	0.48	0.13	0.9	-0.14	0.48	0.15	0.6	Protein kinase, cAMP dependent regulatory, type II alpha	Q8K1M3
Prkar2b	0.0000	-0.21	0.52	0.15	0.99	0.21	0.98	-0.06	0.64	cAMP-dependent protein kinase type II-beta regulatory subunit	P31324
Prkca	-0.1633	0.81	0.35	-0.97	0.35	-0.35	0.23	-0.17	0.52	Protein kinase C alpha type	P20444
Prkcb	-0.3128	0.22	0.2	-0.67	0.0009	-0.40	0.0080	-0.45	0.0004	Protein kinase C beta type	P68404
Prkcg	-0.4657	0.21	0.31	-1.09	0.0001	-0.49	0.0001	-0.88	0.0001	Protein kinase C gamma type	P63318
Prkcsh	0.0000	-0.09	0.71	0.02	0.98	0.51	0.11	-0.07	0.8	Glucosidase 2 subunit beta	O08795
Prkg2	0.0007	-0.02	0.9	-0.45	0.0210	-0.13	0.44	-0.46	0.0050	cGMP-dependent protein kinase	E9QPH0
Prmt1	-0.0007	-0.25	0.45	0.05	0.7	0.12	0.94	-0.20	0.87	Protein arginine N-methyltransferase 1	A0A171KXD3
Prnp	-0.4128	1.17	0.27	-1.22	0.31	-0.46	0.33	-0.04	0.9	Major prion protein	P04925
Prpf19	-0.2999	0.51	0.058	-0.44	0.083	0.01	0.86	0.07	1	Pre-mRNA-processing factor 19	Q99KP6
Prpf8	-0.0032	0.27	0.2	-0.10	0.68	-0.37	0.54	0.17	0.22	Pre-mRNA-processing-splicing factor 8	Q99PV0
Prps1l3	-0.0006	-0.01	0.93	0.71	0.0030	-0.88	0.0030	0.69	0.0010	Ribose-phosphate diphosphokinase	G3UXL2
Prpsap1	0.0005	0.64	0.28	0.17	0.98	0.04	0.73	0.81	0.086	Phosphoribosyl pyrophosphate synthase-associated protein 1	B1AT82
Prpsap2	-0.1599	-0.37	0.17	0.61	0.12	-0.34	0.14	0.24	0.42	Phosphoribosyl pyrophosphate synthase-associated protein 2	Q8R574
Prr36	0.0005	0.45	0.52	0.03	0.72	0.47	0.3	0.48	0.23	Proline-rich 36	E9PV26
Prrc2a	0.0109	0.18	0.42	0.23	0.2	-0.06	0.87	0.42	0.15	Protein PRRC2A	Q7TSC1
Prrc2c	-0.5833	0.93	0.0490	-0.52	0.12	1.08	0.0140	0.40	0.52	Protein PRRC2C	A0A0A0MQ79
Prrt1	-0.0001	-0.05	0.94	0.41	0.54	-0.76	0.62	0.36	0.51	Proline-rich transmembrane protein 1	O35449
Prrt2	0.1379	0.86	0.14	0.22	0.14	1.45	0.0050	1.08	0.064	Proline-rich transmembrane protein 2	E9PUL5
Prrt3	-3.6121	4.79	0.052	-2.13	0.53	5.20	0.0280	2.65	0.16	Proline-rich transmembrane protein 3	A0A0N4SVB5
Prss1	-0.0432	-0.38	0.77	1.13	0.13	0.02	0.91	0.75	0.26	Protease, serine 1 (trypsin 1)	Q9Z1R9
Prune1	-6.7153	2.45	0.0390	-1.92	0.097	0.56	0.49	0.53	0.66	Exopolyphosphatase PRUNE1	Q8BIW1
Psat1	0.0057	-0.30	0.0190	-0.04	0.53	-0.18	0.11	-0.34	0.0010	Phosphoserine aminotransferase	Q99K85
Psd	-0.0097	0.52	0.87	-1.02	0.5	0.06	0.27	-0.50	0.18	PH and SEC7 domain-containing protein 1	Q5DTT2
Psd3	-0.6051	0.65	0.17	-0.99	0.06	0.16	0.88	-0.34	0.55	PH and SEC7 domain-containing protein 3	E9PUC5
Psma1	-0.0005	-0.11	0.74	0.23	0.73	0.18	0.36	0.12	0.58	Proteasome subunit alpha type-1	Q9R1P4

Table S2: Differentially expressed proteins in cerebral cortex *(continued)*

gene	$\pi_{\mathrm{TW/EW}} \times \pi_{\mathrm{TF/TW}}$	log2FC TW/EW	padj TW/EW	log2FC TF/TW	padj TF/TW	log2FC EF/EW	padj EF/EW	log2FC TF/EW	padj TF/EW	description	accession
Psma2	0.0000	0.13	0.42	0.32	1	0.38	0.6	0.45	0.38	Proteasome subunit alpha type-2	P49722
Psma3	-0.0001	-0.23	0.96	0.27	0.84	0.07	1	0.03	0.75	Proteasome subunit alpha type-3	O70435
Psma4	-0.0056	-0.23	0.29	0.11	0.39	0.17	0.0430	-0.12	0.7	Proteasome subunit alpha type-4	Q9R1P0
Psma5	-0.0075	-0.27	0.12	0.10	0.5	0.13	0.43	-0.17	0.54	Proteasome subunit alpha type-5	Q9Z2U1
Psma6	-0.7112	-0.38	0.0004	0.35	0.0270	-0.01	0.65	-0.03	0.45	Proteasome subunit alpha type-6	Q9QUM9
Psma7	-0.0060	-0.14	0.47	0.20	0.22	-0.17	0.089	0.06	0.52	Proteasome subunit alpha type	A0A338P7D7
Psmb1	0.0009	-0.49	0.57	-0.10	0.84	-1.09	0.29	-0.59	0.56	Proteasome subunit beta type-1	O09061
Psmb2	-0.0004	-0.65	0.0280	0.03	0.97	-0.14	0.72	-0.63	0.14	Proteasome subunit beta type-2	Q9R1P3
Psmb4	-0.2486	-0.31	0.0140	0.27	0.0250	0.14	0.22	-0.04	0.61	Proteasome subunit beta type-4	P99026
Psmb5	-0.1327	-0.56	0.0110	0.33	0.43	-0.06	0.41	-0.23	0.076	Proteasome subunit beta type-5	O55234
Psmb6	0.0000	-0.23	1	0.02	0.52	0.22	0.68	-0.21	0.066	Proteasome subunit beta type-6	Q60692
Psmc1	-0.0024	-0.28	0.0030	0.04	0.82	0.04	0.69	-0.24	0.087	26S proteasome regulatory subunit 4	P62192
Psmc2	-0.0251	0.39	0.63	-0.58	0.28	-0.29	0.56	-0.19	0.52	26S proteasome regulatory subunit 7	P46471
Psmc3	0.0000	0.01	0.85	0.05	0.71	-0.06	0.61	0.06	0.52	26S proteasome regulatory subunit 6A	A2AGN7
Psmc4	-0.0004	0.10	0.32	-0.02	0.42	-0.24	0.12	0.08	0.77	26S proteasome regulatory subunit 6B	P54775
Psmc5	-0.0060	0.05	0.78	-0.68	0.0230	-0.39	0.48	-0.63	0.11	26S proteasome regulatory subunit 8	Q8K1K2
Psmc6	0.0010	-0.02	0.62	-0.40	0.25	-0.12	0.52	-0.41	0.34	26S proteasome regulatory subunit 10B	P62334
Psmd1	0.0000	-0.01	0.46	0.09	0.85	0.11	0.58	0.08	0.5	26S proteasome non-ATPase regulatory subunit 1	Q3TXS7
Psmd11	-0.0475	-0.38	0.48	0.38	0.093	0.00	0.67	0.00	0.81	26S proteasome non-ATPase regulatory subunit 11	Q8BG32
Psmd13	0.0040	-0.34	0.52	-0.15	0.53	-0.03	0.99	-0.49	0.13	26S proteasome non-ATPase regulatory subunit 13	Q9WVJ2
Psmd14	-0.0125	-0.26	0.58	0.30	0.21	-0.05	0.59	0.04	0.6	26S proteasome non-ATPase regulatory subunit 14	O35593
Psmd2	-0.0005	0.06	0.46	-0.09	0.51	-0.08	0.57	-0.03	1	26S proteasome non-ATPase regulatory subunit 2	Q8VDM4
Psmd3	-2.4447	0.46	0.0190	-0.88	0.0003	0.15	0.59	-0.42	0.52	26S proteasome non-ATPase regulatory subunit 3	P14685
Psmd4	-0.0267	-0.35	0.0280	0.15	0.47	0.05	0.66	-0.20	0.24	26S proteasome non-ATPase regulatory subunit 4	O35226
Psmd5	-0.0140	0.14	0.68	-0.65	0.12	0.15	0.78	-0.51	0.19	26S proteasome non-ATPase regulatory subunit 5	Q8BJY1
Psmd6	0.5132	3.84	0.38	0.97	0.47	5.31	0.056	4.81	0.087	26S proteasome non-ATPase regulatory subunit 6	Q99JI4
Psmd7	0.0000	0.04	0.5	0.02	0.82	-0.27	0.23	0.05	0.65	26S proteasome non-ATPase regulatory subunit 7	P26516
Psmd8	-0.0652	-0.31	0.21	0.39	0.16	0.08	0.8	0.08	0.67	26S proteasome non-ATPase regulatory subunit 8	Q9CX56

Table S2: Differentially expressed proteins in cerebral cortex *(continued)*

gene	$\pi_{TW/EW}\times$ $\pi_{TF/TW}$	log2FC TW/EW	padj TW/EW	log2FC TF/TW	padj TF/TW	log2FC EF/EW	padj EF/EW	log2FC TF/EW	padj TF/EW	description	accession
Psmd9	0.0000	-0.32	1	-0.28	0.94	0.02	0.85	-0.61	0.48	26S proteasome non-ATPase regulatory subunit 9	Q9CR00
Pspc1	-0.6366	0.33	0.058	-0.52	0.0010	-0.02	0.55	-0.19	0.0410	Paraspeckle component 1	Q8R326
Ptbp2	-0.0975	0.38	0.27	-0.49	0.12	-0.26	0.32	-0.11	0.68	Polypyrimidine tract-binding protein 2	Q91Z31
Ptgds	-0.1952	-0.79	0.0010	0.29	0.52	-0.87	0.0011	-0.51	0.0310	Prostaglandin-H2 D-isomerase	O09114
Ptges3	-0.0031	-0.06	0.65	0.26	0.088	-0.17	0.88	0.20	0.059	Prostaglandin E synthase 3	D3Z7C6
Ptk2	0.0007	0.07	0.78	0.18	0.33	-0.10	0.68	0.25	0.13	Non-specific protein-tyrosine kinase	A0A5F8MPY3
Ptk2b	-0.0882	1.03	0.0240	-0.21	0.56	0.20	0.41	0.82	0.097	Non-specific protein-tyrosine kinase	E9Q2A6
Ptk7	0.0007	3.79	0.44	0.06	0.98	0.43	0.69	3.85	0.49	Inactive tyrosine-protein kinase 7	Q8BKG3
Ptms	-1.3375	-0.60	0.0020	0.43	0.0120	-0.19	0.56	-0.17	0.16	Parathymosin	Q9D0J8
Ptp4a2	0.0000	0.06	0.9	-0.19	0.93	0.05	0.9	-0.12	0.62	Protein tyrosine phosphatase type IVA 2	O70274
Ptpa	-1.2182	-0.91	0.0390	0.74	0.052	-0.20	0.69	-0.18	0.88	Serine/threonine-protein phosphatase 2A activator	A2AWE9
Ptpmt1	-0.1779	0.61	0.0230	-0.38	0.34	0.15	0.51	0.23	0.65	Phosphatidylglycerophosphatase and protein-tyrosine phosphatase 1	A0A5F8MPX4
Ptpn11	0.0000	-0.01	0.88	0.21	0.5	-0.27	0.51	0.20	0.99	Tyrosine-protein phosphatase non-receptor type 11	P35235
Ptpn23	-0.1585	0.69	0.081	-0.32	0.22	0.58	0.28	0.36	0.073	Tyrosine-protein phosphatase non-receptor type 23	Q6PB44
Ptpra	0.0038	-0.13	0.73	-3.25	0.86	0.92	0.45	-3.38	0.8	Receptor-type tyrosine-protein phosphatase alpha	Q91V35
Ptprd	-0.0243	0.15	0.59	-0.54	0.0490	-0.03	0.69	-0.40	0.2	Receptor-type tyrosine-protein phosphatase delta	Q64487
Ptprn2	-0.1489	0.42	0.077	-0.40	0.16	-0.10	0.71	0.03	0.84	Receptor-type tyrosine-protein phosphatase N2	P80560
Ptprs	-0.0079	0.13	0.42	-0.23	0.2	0.25	0.7	-0.10	0.61	Receptor-type tyrosine-protein phosphatase S	B0V2N1
Ptprz1	0.0159	0.12	0.31	0.57	0.35	-0.14	0.38	0.68	0.0420	Receptor-type tyrosine-protein phosphatase zeta	B9EKR1
Ptrh2	-0.0145	1.06	0.67	-0.91	0.82	0.23	0.68	0.15	0.62	Peptidyl-tRNA hydrolase 2, mitochondrial	Q8R2Y8
Pum1	0.0001	0.13	0.61	0.06	0.84	0.26	0.31	0.19	0.37	Pumilio homolog 1	Q80U78
Pum2	-0.0009	-0.22	0.4	0.10	0.79	0.14	0.74	-0.13	0.48	Pumilio homolog 2	Q3TQ29
Pura	-0.6428	0.50	0.0003	-0.28	0.0480	-0.35	0.0260	0.23	0.0020	Transcriptional activator protein Pur-alpha	P42669
Purb	-0.3488	0.78	0.26	-0.83	0.12	0.02	0.7	-0.05	0.67	Transcriptional activator protein Pur-beta	O35295
Purg	-1.5429	0.79	0.065	-0.84	0.0110	-0.37	0.97	-0.05	0.92	Purine-rich element-binding protein gamma	Q8R4E6
Pycr2	0.0197	-0.25	0.6	-0.28	0.054	0.01	0.81	-0.53	0.19	Pyrroline-5-carboxylate reductase 2	Q922Q4
Pycr3	0.1288	0.14	0.39	0.75	0.0010	-0.04	0.66	0.89	0.0020	Pyrroline-5-carboxylate reductase 3	Q9DCC4

Table S2: Differentially expressed proteins in cerebral cortex *(continued)*

gene	$\pi_{TW/EW} \times \pi_{TF/TW}$	log2FC TW/EW	padj TW/EW	log2FC TF/TW	padj TF/TW	log2FC EF/EW	padj EF/EW	log2FC TF/EW	padj TF/EW	description	accession
Pygb	0.0039	-0.25	0.45	-0.09	0.32	-0.12	0.94	-0.35	0.11	Glycogen phosphorylase, brain form	Q8CI94
Pzp	-0.0794	-0.62	0.0140	0.16	0.37	-0.13	0.98	-0.46	0.15	Pregnancy zone protein	Q61838
Q8K207	-0.0001	-0.02	0.94	0.19	0.23	-0.18	0.95	0.16	0.29	Uncharacterized protein C1orf21 homolog	Q8K207
Qars1	0.0668	0.53	0.27	0.25	0.13	0.42	0.5	0.79	0.0230	Glutamine–tRNA ligase	Q8BML9
Qdpr	0.0030	-0.35	0.0190	-0.03	0.68	-0.01	0.58	-0.38	0.0320	Dihydropteridine reductase	A0A0G2JGJ1
Qki	-0.2342	-0.58	0.23	0.53	0.064	-0.31	0.57	-0.05	0.88	Protein quaking	Q9QYS9
Rab11b	-0.0048	-0.36	0.12	0.18	0.83	-0.09	0.56	-0.18	0.47	Ras-related protein Rab-11B	G3UY29
Rab11fip2	-0.4562	1.34	0.3	-1.28	0.31	-1.00	0.23	0.06	0.79	Rab11 family-interacting protein 2	G3XA57
Rab11fip5	-0.9556	0.93	0.055	-0.78	0.09	0.04	0.68	0.16	0.88	Rab11 family-interacting protein 5	A0A0N4SW73
Rab13	0.0000	-0.01	0.97	-0.05	1	0.24	0.21	-0.06	0.93	Ras-related protein Rab-13	Q9DD03
Rab18	0.0000	-0.01	0.72	0.01	0.75	-0.81	0.12	0.00	0.96	Ras-related protein Rab-18	A0A452J8C1
Rab23	1.6828	-0.68	0.12	-2.45	0.08	0.01	0.97	-3.14	0.0280	Ras-related protein Rab-23	P35288
Rab27b	0.0044	-0.10	0.76	-0.60	0.24	-0.46	0.43	-0.70	0.17	Ras-related protein Rab-27B	Q99P58
Rab2a	0.0042	-0.32	0.51	-0.21	0.61	-0.25	0.69	-0.53	0.22	Ras-related protein Rab-2A	P53994
Rab33b	0.0001	0.60	0.89	0.06	0.92	0.02	0.68	0.66	0.91	Ras-related protein Rab-33B	O35963
Rab3gap2	0.0006	-0.47	0.88	-0.11	0.6	-0.47	0.4	-0.58	0.53	Rab3 GTPase-activating protein non-catalytic subunit	E9QKE4
Rab5b	-4.3040	0.69	0.0110	-1.18	0.0020	-0.32	0.71	-0.49	0.37	Ras-related protein Rab-5B	P61021
Rab6a	-0.0182	0.35	0.26	-0.23	0.41	-0.32	0.59	0.13	0.75	Ras-related protein Rab-6A	P35279
Rab7a	0.0022	-0.01	0.55	-1.23	0.21	0.51	0.27	-1.23	0.31	Ras-related protein Rab-7a	P51150
Rab8b	-0.0033	0.05	0.32	-0.18	0.18	0.11	0.88	-0.13	0.78	Ras-related protein Rab-8B	P61028
Rabep1	-0.0185	0.39	0.22	-0.22	0.47	0.10	0.89	0.16	0.61	Rab GTPase-binding effector protein 1	O35551
Rabgap1	-0.0005	-0.34	0.28	0.21	0.97	0.43	0.16	-0.14	0.61	Rab GTPase-activating protein 1	A2AWA9
Rabggta	-0.0091	-0.17	0.41	0.21	0.22	-0.10	0.73	0.05	0.75	Geranylgeranyl transferase type-2 subunit alpha	Q9JHK4
Rac1	0.0034	-0.25	0.36	-0.07	0.36	-0.05	0.61	-0.32	0.068	Ras-related C3 botulinum toxin substrate 1	P63001
Rack1	0.0000	0.21	0.21	0.02	0.99	0.04	0.4	0.24	0.12	Receptor of activated protein C kinase 1	P68040
Rala	0.0006	-0.33	0.47	-0.06	0.81	0.31	0.28	-0.39	0.48	Ras-related protein Ral-A	P63321
Ralb	-0.0042	0.20	0.57	-0.22	0.41	0.09	0.72	-0.02	0.89	Ras-related protein Ral-B	Q9JIW9
Ralgapa1	-0.0024	-0.15	0.71	0.17	0.23	1.05	0.0430	0.02	0.25	Ral GTPase-activating protein subunit alpha-1	A0A2I3BRX9
Ralgapb	0.0005	0.10	0.82	0.27	0.62	-0.04	0.94	0.37	0.62	Ral GTPase-activating protein subunit beta	A2ACC6
Raly	0.0002	-0.04	0.97	-1.17	0.43	-2.41	0.25	-1.21	0.44	RNA-binding protein Raly (Fragment)	A2AU61
Ran	0.0365	-0.34	0.15	-0.21	0.24	-0.38	0.078	-0.55	0.0010	GTP-binding nuclear protein Ran	P62827
Ranbp1	0.0015	-0.28	0.0250	-0.02	0.68	-0.28	0.062	-0.31	0.0170	Ran-specific GTPase-activating protein	H7BX22
Ranbp10	-0.3240	3.59	0.59	-3.65	0.78	-1.21	0.57	-0.06	0.83	Ran-binding protein 10	A0A0R4J0G4

Table S2: Differentially expressed proteins in cerebral cortex *(continued)*

gene	$\pi_{TW/EW} \times \pi_{TF/TW}$	log2FC TW/EW	padj TW/EW	log2FC TF/TW	padj TF/TW	log2FC EF/EW	padj EF/EW	log2FC TF/EW	padj TF/EW	description	accession
Ranbp2	-32.0944	2.29	0.0050	-2.54	0.0040	0.23	0.35	-0.26	0.78	E3 SUMO-protein ligase RanBP2	Q9ERU9
Rangap1	0.0000	0.02	0.69	0.04	0.92	-0.54	0.12	0.06	0.55	Ran GTPase-activating protein 1	P46061
Rap1gap	0.0002	0.34	0.25	0.06	0.96	-0.14	0.97	0.40	0.14	Rap1 GTPase-activating protein 1	A2ALS5
Rapgef2	-0.1630	0.58	0.15	-0.60	0.27	-0.04	0.92	-0.02	0.95	Cyclic nucleotide ras GEF	A0A0A6YWG7
Rapgef4	-7.4794	1.00	0.066	-2.06	0.0008	-0.04	0.78	-1.06	0.11	Rap guanine nucleotide exchange factor 4	Q9EQZ6
Raph1	-0.0065	-0.22	0.78	0.51	0.29	0.19	0.54	0.28	0.48	Ras association (RalGDS/AF-6) and pleckstrin homology domains 1	F2Z3U3
Rars2	0.0566	-0.41	0.54	-1.37	0.42	-0.83	0.49	-1.78	0.25	Probable arginine–tRNA ligase, mitochondrial	Q3U186
Rasa3	0.0001	0.05	0.34	0.13	0.94	-0.09	0.8	0.18	0.67	Ras GTPase-activating protein 3	Q60790
Rasal1	0.0002	0.09	0.61	0.07	0.77	-0.10	0.64	0.16	0.42	RasGAP-activating-like protein 1	Q9Z268
Rasal2	-7.0964	1.26	0.085	-2.12	0.0033	-0.35	0.6	-0.87	0.35	RAS protein activator-like 2	D3YUS5
Rasgef1a	-0.0197	0.94	0.8	-1.08	0.63	0.12	0.6	-0.14	0.77	RasGEF domain family, member 1A	A0A0N4SVF2
Rasgrf1	-2.8265	3.67	0.6	-7.21	0.33	-3.41	0.54	-3.55	0.66	Ras-specific guanine nucleotide-releasing factor 1	P27671
Rasgrf2	-1.4244	1.20	0.32	-1.59	0.0310	-0.47	0.66	-0.39	0.6	Ras-specific guanine nucleotide-releasing factor 2	D3Z6K8
Rbbp7	-0.3898	1.01	0.3	-0.77	0.11	0.64	0.53	0.24	0.88	Histone-binding protein RBBP7	A2AFI9
Rbfox3	0.0003	-0.07	0.85	-0.26	0.59	-0.42	0.58	-0.33	0.37	RNA binding protein fox-1 homolog 3	Q8BIF2
Rbm14	-4.3743	1.53	0.0250	-1.57	0.073	-0.41	0.51	-0.05	0.52	RNA-binding protein 14	Q8C2Q3
Rbm26	0.0002	1.00	0.091	0.01	0.95	-0.33	0.8	1.01	0.0330	RNA-binding protein 26	E9PYZ7
Rbsn	0.0208	0.45	0.4	0.21	0.28	-0.10	0.68	0.66	0.16	Rabenosyn-5	Q80Y56
Rcan1	-1.1573	-1.15	0.085	0.94	0.1	-0.05	0.92	-0.22	0.47	Calcipressin-1	Q9JHG6
Rcc2	-0.0001	0.05	0.92	-0.25	0.5	0.03	0.82	-0.20	0.097	Protein RCC2	Q8BK67
Rdh14	0.0004	0.21	0.47	0.08	0.86	0.33	0.14	0.29	0.3	Retinol dehydrogenase 14	Q9ERI6
Rdx	0.0167	0.38	0.37	0.38	0.54	0.94	0.069	0.76	0.15	Radixin	P26043
Reep1	-0.0164	0.96	0.0340	-0.23	0.89	0.28	0.4	0.73	0.12	Receptor expression-enhancing protein 1	Q8BGH4
Reep2	0.0011	0.24	0.063	0.05	0.84	0.14	0.66	0.28	0.0250	Receptor expression-enhancing protein 2	Q8VCD6
Reep5	0.0001	0.06	0.88	0.15	0.65	0.19	0.68	0.21	0.51	Receptor expression-enhancing protein 5 (Fragment)	A0A494BBE3
Relch	-0.2118	1.32	0.62	-1.89	0.39	-0.33	0.85	-0.57	0.86	RAB11-binding protein RELCH	E9QM90
Rem2	-0.0021	0.11	0.65	-0.32	0.48	0.49	0.082	-0.21	0.65	GTP-binding protein REM 2	E9Q4D5
Reps1	-0.0020	-0.03	0.86	0.57	0.0180	0.27	0.26	0.53	0.0190	RalBP1-associated Eps domain-containing protein 1	E9Q632
Reps2	-0.0493	0.54	0.3	-0.29	0.25	0.07	0.92	0.26	0.98	RalBP1-associated Eps domain-containing protein 2	A2AFI8
Rgs14	-0.0115	0.23	0.35	-0.26	0.38	0.09	0.84	-0.02	0.94	Regulator of G-protein signaling 14	P97492
Rgs17	-0.0003	0.06	0.79	-0.20	0.58	-0.15	0.65	-0.14	0.69	Regulator of G-protein-signaling 17	G5E8E0

Table S2: Differentially expressed proteins in cerebral cortex *(continued)*

gene	$\pi_{TW/EW} \times \pi_{TF/TW}$	log2FC TW/EW	padj TW/EW	log2FC TF/TW	padj TF/TW	log2FC EF/EW	padj EF/EW	log2FC TF/EW	padj TF/EW	description	accession
Rgs6	0.0000	-0.35	0.96	0.35	0.99	0.12	0.48	0.00	0.81	Regulator of G-protein-signaling 6 (Fragment)	A0A087WPC4
Rgs7	-0.0002	-0.69	0.64	0.31	0.99	0.49	0.73	-0.39	0.86	Regulator of G-protein-signaling 7	Q80XD3
Rheb	0.0000	-0.02	0.98	-0.37	0.33	0.11	0.72	-0.39	0.24	GTP-binding protein Rheb	Q921J2
Rhob	0.0000	-0.21	0.23	0.01	0.92	0.26	0.19	-0.20	0.15	Rho-related GTP-binding protein RhoB	P62746
Rhog	0.0000	0.03	0.97	0.06	0.63	0.16	0.27	0.09	0.4	Rho-related GTP-binding protein RhoG	P84096
Rhot1	-0.0145	0.31	0.45	-0.33	0.39	0.14	0.68	-0.01	0.9	Mitochondrial Rho GTPase 1	Q8BG51
Ric8a	0.0509	-0.34	0.24	-0.99	0.57	-0.84	0.81	-1.33	0.34	Synembryn-A	Q3TIR3
Rida	-0.0257	-0.57	0.0010	0.07	0.61	-0.42	0.0270	-0.50	0.0020	2-iminobutanoate/2-iminopropanoate deaminase	P52760
Rimbp2	-0.9259	1.13	0.26	-1.70	0.15	-0.44	0.25	-0.57	0.23	RIMS-binding protein 2	D3YXR8
Rims1	-2.2306	1.53	0.53	-3.19	0.0220	-1.58	0.063	-1.65	0.0350	Regulating synaptic membrane exocytosis protein 1	Q99NE5
Rims2	-0.1399	5.31	0.51	-1.78	0.89	4.58	0.27	3.53	0.51	Regulating synaptic membrane exocytosis 2	D9HP81
Rims3	-0.0554	0.50	0.43	-0.70	0.37	-0.08	0.5	-0.19	0.22	Regulating synaptic membrane exocytosis protein 3	Q80U57
Rln3	0.0444	-0.36	0.97	-8.66	0.084	-4.46	0.44	-9.03	0.0280	Relaxin-3	Q8CHK2
Rmc1	-0.0436	-0.39	0.46	0.60	0.28	0.04	0.98	0.21	0.67	Regulator of MON1-CCZ1 complex	Q8VC42
Rmdn3	-0.0019	-0.13	0.89	0.56	0.3	0.50	0.16	0.43	0.18	Regulator of microtubule dynamics protein 3	Q3UJU9
Rnf214	-0.0942	0.42	0.0240	-0.23	0.25	-0.10	0.79	0.19	0.2	RING finger protein 214	Q8BFU3
Robo2	-1.3516	0.94	0.0010	-0.50	0.11	0.01	0.81	0.45	0.0100	Roundabout homolog 2	E9Q6A0
Rogdi	0.0000	-0.27	1	0.80	0.0001	0.09	0.53	0.54	0.0001	Protein rogdi homolog	Q3TDK6
Rph3a	-1.1134	1.36	0.0230	-0.83	0.25	-0.16	0.61	0.53	0.2	Rabphilin-3A	P47708
Rpl10a	-0.1087	0.78	0.76	-1.27	0.12	-0.23	0.48	-0.49	0.0380	Ribosomal protein	A0A3B2WDD2
Rpl11	-6.7472	0.75	0.0010	-0.79	0.0002	-0.06	0.84	-0.03	0.9	60S ribosomal protein L11	Q9CXW4
Rpl12	-0.3103	0.41	0.2	-0.75	0.0360	0.29	0.37	-0.33	0.38	60S ribosomal protein L12	P35979
Rpl13	-11.6640	1.51	0.087	-2.38	0.0009	-0.77	0.13	-0.87	0.05	60S ribosomal protein L13	P47963
Rpl13a	-4.5345	0.80	0.072	-1.39	0.0003	-0.34	0.57	-0.59	0.053	60S ribosomal protein L13a (Fragment)	A0A1B0GTA1
Rpl14	-1.6556	0.95	0.14	-1.26	0.0240	-0.71	0.0210	-0.31	0.28	60S ribosomal protein L14	Q9CR57
Rpl15	-0.1493	0.52	0.84	-2.56	0.0330	-1.38	0.1	-2.04	0.0270	60S ribosomal protein L15	Q9CZM2
Rpl17	-1.1568	1.38	0.0290	-0.78	0.2	0.00	0.8	0.59	0.0380	60S ribosomal protein L17	Q9CPR4
Rpl18	-0.0752	1.08	0.7	-1.37	0.47	-0.51	0.41	-0.29	0.59	60S ribosomal protein L18	P35980
Rpl18a	-4.1600	0.78	0.0330	-1.20	0.0010	-0.32	0.16	-0.41	0.0270	60S ribosomal protein L18a	P62717
Rpl19	-0.0072	0.68	0.91	-1.06	0.57	-0.47	0.0310	-0.38	0.14	Ribosomal protein L19	A2A547
Rpl21	-3.4179	0.89	0.082	-1.31	0.0020	-0.37	0.0020	-0.42	0.0110	60S ribosomal protein L21	Q9CQM8
Rpl22	-0.3138	0.79	0.32	-0.94	0.14	-0.49	0.33	-0.16	0.52	60S ribosomal protein L22	P67984
Rpl22l1	-0.0117	0.55	0.43	-0.27	0.61	0.00	0.9	0.28	0.26	60S ribosomal protein L22-like 1	Q9D7S7

Table S2: Differentially expressed proteins in cerebral cortex *(continued)*

gene	$\pi_{TW/EW} \times \pi_{TF/TW}$	log2FC TW/EW	padj TW/EW	log2FC TF/TW	padj TF/TW	log2FC EF/EW	padj EF/EW	log2FC TF/EW	padj TF/EW	description	accession
Rpl23	-2.2063	0.82	0.0120	-0.78	0.0160	-0.26	0.064	0.04	0.54	60S ribosomal protein L23	P62830
Rpl23a	-14.6212	1.10	0.0001	-1.12	0.0009	-0.52	0.24	-0.02	0.71	60S ribosomal protein L23a	P62751
Rpl24	-2.2905	1.30	0.3	-2.59	0.05	-3.34	0.0220	-1.30	0.21	60S ribosomal protein L24	Q8BP67
Rpl26	-0.0084	0.64	0.93	-0.97	0.37	-0.40	0.16	-0.33	0.053	60S ribosomal protein L26	P61255
Rpl27	-0.2490	0.39	0.093	-0.60	0.093	-0.26	0.52	-0.21	0.81	60S ribosomal protein L27	P61358
Rpl27a	-1.0301	0.84	0.38	-1.49	0.0110	-0.93	0.0350	-0.65	0.12	60S ribosomal protein L27a	P14115
Rpl28	-0.8368	1.24	0.26	-1.40	0.15	-0.46	0.39	-0.17	0.65	60S ribosomal protein L28	P41105
Rpl29	-0.1829	0.96	0.39	-1.05	0.36	-0.13	0.71	-0.08	0.99	60S ribosomal protein L29	P47915
Rpl3	-12.4391	1.10	0.0150	-1.55	0.0001	-0.44	0.16	-0.45	0.13	60S ribosomal protein L3	P27659
Rpl30	-2.9101	0.75	0.0250	-0.96	0.0030	-0.20	0.72	-0.20	0.78	60S ribosomal protein L30	P62889
Rpl31	-0.0032	0.24	0.92	-1.52	0.57	-0.22	0.78	-1.29	0.51	60S ribosomal protein L31	P62900
Rpl34	-2.8151	1.52	0.087	-1.60	0.081	-0.84	0.19	-0.08	0.91	60S ribosomal protein L34	Q9D1R9
Rpl35	-0.1086	0.92	0.34	-0.97	0.55	-0.46	0.5	-0.05	0.7	60S ribosomal protein L35	Q6ZWV7
Rpl35a	-0.4748	0.70	0.072	-0.67	0.13	0.19	0.26	0.03	0.56	60S ribosomal protein L35a	O55142
Rpl37	-0.0004	0.80	0.98	-1.48	0.91	-0.55	0.0100	-0.68	0.0160	60S ribosomal protein L37	Q9D823
Rpl37a	-0.7867	0.66	0.12	-0.85	0.0300	-0.27	0.39	-0.19	0.37	60S ribosomal protein L37a	P61514
Rpl4	-0.5741	0.83	0.39	-1.30	0.05	-0.46	0.0440	-0.47	0.069	60S ribosomal protein L4	Q9D8E6
Rpl5	0.0219	0.48	0.0020	0.28	0.87	0.33	0.0020	0.76	0.0001	60S ribosomal protein L5	P47962
Rpl6	-7.7386	1.17	0.0040	-1.28	0.0070	-0.19	0.89	-0.10	0.89	60S ribosomal protein L6	P47911
Rpl7	-3.7294	1.20	0.23	-1.93	0.0030	-0.59	0.0270	-0.73	0.0040	60S ribosomal protein L7	P14148
Rpl7a	-3.1340	1.06	0.12	-1.49	0.0070	-0.36	0.56	-0.43	0.17	60S ribosomal protein L7a	P12970
Rpl8	-0.1484	1.01	0.58	-1.29	0.33	-0.52	0.19	-0.29	0.072	60S ribosomal protein L8	P62918
Rplp0	0.0029	0.10	0.052	0.11	0.62	0.33	0.61	0.21	0.83	60S acidic ribosomal protein P0	P14869
Rplp2	-0.0016	-0.07	0.86	0.67	0.3	0.21	0.98	0.60	0.19	60S acidic ribosomal protein P2	P99027
Rpn1	0.2745	0.48	0.0010	0.52	0.43	0.25	0.54	0.99	0.0001	Dolichyl-diphosphooligosaccharide–protein glycosyltransferase subunit 1	Q91YQ5
Rps11	-1.9346	0.89	0.0450	-0.95	0.0200	-0.40	0.56	-0.07	0.87	40S ribosomal protein S11	P62281
Rps13	-0.0556	1.55	0.33	-0.92	0.83	-0.71	0.7	0.63	0.38	40S ribosomal protein S13	P62301
Rps14	-0.2303	0.63	0.32	-0.96	0.17	-1.03	0.0430	-0.33	0.64	40S ribosomal protein S14	P62264
Rps15a	-0.4876	0.76	0.0310	-0.48	0.13	-0.34	0.21	0.29	0.14	40S ribosomal protein S15a (Fragment)	F8WJ41
Rps16	-0.5313	0.81	0.0400	-0.63	0.18	-0.21	0.22	0.18	0.51	40S ribosomal protein S16	P14131
Rps17	0.0000	-0.03	0.94	0.07	0.7	0.10	0.65	0.03	0.65	40S ribosomal protein S17	P63276
Rps18-ps5	-5.8191	0.72	0.0019	-0.99	0.0010	-0.33	0.29	-0.27	0.47	40S ribosomal protein S18	A0A1Y7VKY1
Rps19	-2.6054	1.27	0.0010	-0.83	0.15	-0.04	0.96	0.44	0.056	40S ribosomal protein S19	Q9CZX8
Rps2	-0.0038	0.79	0.61	-0.62	0.92	-0.36	0.23	0.17	0.28	40S ribosomal protein S2	P25444
Rps21	-5.6964	-0.47	0.0010	1.01	0.0001	-0.05	0.63	0.54	0.0001	40S ribosomal protein S21	Q9CQR2
Rps23	-0.5604	0.45	0.067	-0.64	0.0220	-0.01	0.97	-0.19	0.23	40S ribosomal protein S23	P62267
Rps25	-6.0143	1.53	0.0010	-1.11	0.066	-0.18	0.74	0.42	0.2	40S ribosomal protein S25	P62852

Table S2: Differentially expressed proteins in cerebral cortex *(continued)*

gene	$\pi_{TW/EW} \times \pi_{TF/TW}$	log2FC TW/EW	padj TW/EW	log2FC TF/TW	padj TF/TW	log2FC EF/EW	padj EF/EW	log2FC TF/EW	padj TF/EW	description	accession
Rps27a	-0.0557	0.75	0.17	-0.35	0.53	0.44	0.27	0.41	0.16	Ubiquitin-40S ribosomal protein S27a	P62983
Rps28	-2.0419	-0.73	0.0280	1.16	0.0280	-0.41	0.3	0.42	0.17	40S ribosomal protein S28	P62858
Rps3	-0.6713	0.50	0.0010	-0.27	0.0220	-0.02	0.79	0.23	0.39	40S ribosomal protein S3	P62908
Rps3a	-0.7542	2.00	0.0170	-0.93	0.59	-0.83	0.45	1.07	0.051	40S ribosomal protein S3a	P97351
Rps4x	-0.3833	0.76	0.17	-0.88	0.18	-0.22	0.92	-0.12	1	40S ribosomal protein S4, X isoform	P62702
Rps5	0.0000	0.01	0.68	-0.03	0.68	0.13	0.51	-0.03	0.96	40S ribosomal protein S5	P97461
Rps6	-1.1092	0.86	0.21	-1.12	0.0200	-0.52	0.0450	-0.25	0.19	40S ribosomal protein S6	P62754
Rps7	-0.0065	0.62	0.52	-0.49	0.84	-0.11	0.78	0.13	0.15	40S ribosomal protein S7	P62082
Rps8	-1.2713	1.48	0.0010	-0.76	0.42	0.00	0.46	0.73	0.0440	40S ribosomal protein S8	P62242
Rps9	-0.1315	0.70	0.59	-0.96	0.14	-0.55	0.076	-0.26	0.23	40S ribosomal protein S9	Q6ZWN5
Rpsa	-3.0511	-0.33	0.0057	1.03	0.0001	-0.12	0.38	0.69	0.0001	40S ribosomal protein SA	P14206
Rptor	-0.0077	0.22	0.85	-0.85	0.26	-0.43	0.54	-0.63	0.28	Regulatory-associated protein of mTOR	A2ACM0
Rrbp1	0.0099	0.36	0.0049	0.26	0.9	0.03	0.98	0.62	0.0020	Ribosome-binding protein 1	A2AVJ7
Rtcb	-0.0565	0.67	0.41	-0.44	0.32	-0.33	0.64	0.23	0.91	RNA-splicing ligase RtcB homolog	Q99LF4
Rtn1	0.0000	0.02	0.89	-0.05	0.87	0.09	0.66	-0.03	0.87	Reticulon-1	Q8K0T0
Rtn3	-0.0421	0.09	0.21	-0.32	0.0070	0.07	0.5	-0.23	0.36	Reticulon-3	Q9ES97
Rtn4	0.0008	-0.15	0.16	-0.04	0.69	0.24	0.0410	-0.19	0.06	Reticulon-4	Q99P72
RTRAF	-0.1679	0.87	0.0330	-0.31	0.38	-0.48	0.63	0.55	0.53	RNA transcription, translation and transport factor protein	Q9CQE8
Rufy3	-0.0040	-0.29	0.25	0.11	0.62	-0.21	0.4	-0.18	0.44	Protein RUFY3	Q9D394
Rundc3a	0.0463	0.47	0.12	0.30	0.44	-0.10	0.85	0.78	0.0160	RUN domain-containing protein 3A	O08576
Ruvbl1	0.0145	0.41	0.073	0.41	0.84	0.17	0.55	0.81	0.0020	RuvB-like 1	P60122
Ruvbl2	-0.0739	-0.28	0.34	0.48	0.067	-0.02	0.61	0.20	0.85	RuvB-like 2	Q9WTM5
Ryr2	0.0003	0.04	0.6	0.22	0.67	-0.02	0.81	0.25	0.26	Ryanodine receptor 2	E9Q401
Ryr3	-0.0648	-0.54	0.062	0.23	0.37	-0.13	0.63	-0.31	0.18	Ryanodine receptor 3	A0A140LJK7
S100a13	-0.2999	-0.48	0.0280	0.39	0.093	0.05	0.86	-0.09	0.75	Protein S100-A13	A0A0A0MQ90
Sacm1l	0.0044	0.10	0.75	0.27	0.0480	-0.37	0.0180	0.37	0.0140	Phosphatidylinositol-3-phosphatase SAC1	A0A5F8MPK9
Sacs	-19.4157	1.41	0.0010	-1.53	0.0010	-0.04	0.81	-0.12	0.87	Sacsin	Q9JLC8
Safb	-0.1657	0.55	0.38	-0.87	0.15	-1.26	0.0290	-0.32	0.47	Scaffold attachment factor B1	D3YXK2
Samm50	-0.0025	0.36	0.0280	-0.01	0.35	0.29	0.4	0.35	0.0450	Sorting and assembly machinery component 50 homolog	Q8BGH2
Sar1a	-0.0010	0.13	0.77	-0.24	0.53	-0.41	0.34	-0.10	0.65	GTP-binding protein SAR1a	P36536
Sarm1	-2.5541	0.85	0.0380	-1.16	0.0150	0.68	0.25	-0.31	0.37	NAD(+) hydrolase SARM1	Q6PDS3
Sarnp	0.0000	-0.20	0.54	0.00	0.99	-0.50	0.0100	-0.19	0.37	SAP domain-containing ribonucleoprotein	Q9D1J3
Sars	0.0005	-0.04	0.77	-0.15	0.17	0.00	0.96	-0.19	0.99	Seryl-tRNA synthetase	Q8C483
Sbds	-16.1666	1.81	0.0060	-2.01	0.0100	-0.41	0.64	-0.20	0.58	Ribosome maturation protein SBDS	P70122
Sbf1	-0.0428	0.53	0.18	-0.18	0.25	0.13	0.51	0.36	0.62	Myotubularin-related protein 5	Q6ZPE2
Sbf2	-78.9369	5.28	0.0280	-6.20	0.0280	-0.76	0.22	-0.92	0.094	Myotubularin-related protein 13	E9PXF8

Table S2: Differentially expressed proteins in cerebral cortex *(continued)*

gene	$\pi_{TW/EW} \times \pi_{TF/TW}$	log2FC TW/EW	padj TW/EW	log2FC TF/TW	padj TF/TW	log2FC EF/EW	padj EF/EW	log2FC TF/EW	padj TF/EW	description	accession
Scai	-1.1492	-0.49	0.0006	0.40	0.0150	0.35	0.25	-0.09	0.24	Protein SCAI	Q8C8N2
Scamp5	0.0000	-0.02	0.9	0.05	0.92	-0.08	0.78	0.02	0.91	Secretory carrier-associated membrane protein 5	Q9JKD3
Sccpdh	0.0021	0.32	0.0130	0.03	0.77	0.20	0.69	0.35	0.0001	Saccharopine dehydrogenase-like oxidoreductase	Q8R127
Scfd1	0.0500	1.12	0.35	0.26	0.42	-0.64	0.77	1.38	0.14	Sec1 family domain-containing protein 1	Q8BRF7
Scg2	-0.0628	-0.21	0.11	0.23	0.0440	0.02	0.79	0.02	0.9	Secretogranin-2	Q03517
Scn2a	-0.0137	-0.37	0.0040	0.05	0.49	0.12	0.24	-0.32	0.0220	Sodium channel protein	A0A5H1ZRM8
Scn2b	-0.0003	-0.19	0.41	0.06	0.85	0.23	0.2	-0.14	0.41	Sodium channel subunit beta-2	Q56A07
Sco2	0.0261	0.52	0.091	0.17	0.52	0.51	0.0017	0.69	0.0030	Protein SCO2 homolog, mitochondrial	Q8VCL2
Scp2	-0.0209	0.15	0.92	-1.67	0.0050	-0.74	0.27	-1.52	0.0100	Sterol carrier protein 2	P32020
Scrn1	0.0000	-0.21	0.062	-0.01	0.98	-0.02	0.68	-0.22	0.0490	Secernin-1	Q9CZC8
Scyl2	-0.3427	-0.26	0.15	0.40	0.0001	-0.27	0.12	0.13	0.0420	SCY1-like protein 2	G5E8J9
Sdcbp	-0.0471	0.53	0.053	-0.19	0.43	0.25	0.45	0.34	0.0400	Syntenin-1	O08992
Sdha	-0.6282	0.25	0.0300	-0.55	0.0010	0.09	1	-0.30	0.36	Succinate dehydrogenase [ubiquinone] flavoprotein subunit, mitochondrial	Q8K2B3
Sdhb	-0.0705	0.13	0.42	-0.48	0.0010	0.05	0.87	-0.35	0.0010	Succinate dehydrogenase [ubiquinone] iron-sulfur subunit, mitochondrial	Q9CQA3
Sdk2	0.0000	-0.04	0.98	0.12	0.99	0.26	0.59	0.08	0.83	Protein sidekick-2	Q6V4S5
Sec16a	-0.0005	0.22	0.95	-0.59	0.67	-0.54	0.66	-0.37	0.68	Protein transport protein Sec16A	E9QAT4
Sec22b	0.0085	0.31	0.14	0.12	0.54	0.38	0.11	0.43	0.054	Vesicle-trafficking protein SEC22b	O08547
Sec23a	0.0009	0.07	0.6	0.26	0.61	0.12	0.78	0.33	0.67	Protein transport protein SEC23	E9Q1S3
Sec24b	0.0074	0.04	0.68	0.46	0.0040	-0.01	0.99	0.50	0.0060	Sec24-related gene family, member B (S. cerevisiae)	Q80ZX0
Sec24c	0.0064	0.43	0.22	0.20	0.77	-0.09	0.68	0.62	0.46	Sec24-related gene family, member C (S. cerevisiae)	A0A286YDI8
Sec31a	0.0000	-0.04	0.96	0.16	0.5	-0.26	0.068	0.12	0.31	Protein transport protein Sec31A	Q3UPL0
Sec62	0.0243	1.57	0.14	0.16	0.77	0.05	0.86	1.74	0.14	Translocation protein SEC62	Q8BU14
Sel1l	0.0015	0.15	0.76	0.27	0.48	0.12	0.45	0.42	0.22	Protein sel-1 homolog 1	J3QJX3
Selenbp2	0.0166	-0.64	0.0090	-0.18	0.85	-0.40	0.44	-0.82	0.0450	Selenium-binding protein 2 (Fragment)	G3UWK0
Sema4a	0.0001	0.03	0.97	0.54	0.23	-0.51	0.34	0.57	0.0280	Semaphorin-4A	Q62178
Sema7a	0.0000	0.05	0.96	-0.48	0.78	-1.09	0.56	-0.43	0.87	Semaphorin-7A	Q9QUR8
Septin11	-0.0063	0.03	0.13	-0.17	0.0410	0.04	0.71	-0.14	0.48	Septin	A0A0J9YTY0
Septin2	0.0035	-0.11	0.83	-0.30	0.0480	-0.27	0.26	-0.41	0.0260	Septin-2	E9Q3V6
Septin3	0.0009	0.10	0.49	0.22	0.74	0.06	0.88	0.31	0.32	Neuronal-specific septin-3	A0A5F8MPL6
Septin4	0.0003	-0.02	0.83	-0.13	0.0370	-0.11	0.19	-0.16	0.11	Septin-4	A0A5F8MP96
Septin5	0.0046	0.17	0.0190	0.03	0.3	-0.03	0.69	0.20	0.16	Septin	A0A338P769
Septin6	-0.0103	-0.15	0.65	0.21	0.0180	0.08	0.76	0.06	0.32	Septin-6	Q9R1T4

Table S2: Differentially expressed proteins in cerebral cortex *(continued)*

gene	$\pi_{TW/EW}\times$ $\pi_{TF/TW}$	log2FC TW/EW	padj TW/EW	log2FC TF/TW	padj TF/TW	log2FC EF/EW	padj EF/EW	log2FC TF/EW	padj TF/EW	description	accession
Septin8	-0.0005	-0.06	0.88	0.23	0.24	0.10	0.74	0.17	0.52	Septin-8	B1AQZ0
Septin9	0.0003	0.08	0.16	0.14	0.93	0.09	0.78	0.22	0.23	Septin-9	A2A6U3
Serbp1	0.0074	1.76	0.0170	0.18	0.97	-0.49	0.71	1.94	0.0130	Plasminogen activator inhibitor 1 RNA-binding protein	A0A0N4SV32
Serpina1c	-0.0079	-0.50	0.0180	0.08	0.77	-0.99	0.0330	-0.42	0.095	Alpha-1-antitrypsin 1-3	A0A0R4J0X5
Serpinb1a	-0.0194	0.41	0.9	-2.33	0.36	-1.01	0.32	-1.93	0.25	Leukocyte elastase inhibitor A	Q9D154
Serpine1	-0.7973	0.85	0.2	-1.40	0.11	0.21	0.54	-0.54	0.51	Plasminogen activator inhibitor 1	G5E899
Serpine2	0.0048	0.55	0.21	0.08	0.69	0.61	0.41	0.63	0.25	Glia-derived nexin	Q07235
Serpinh1	0.0000	-0.02	0.96	-0.10	0.59	0.05	0.58	-0.12	0.45	Serpin H1	P19324
Sestd1	-0.1813	0.23	0.58	-1.50	0.0060	-0.54	0.3	-1.27	0.07	SEC14 domain and spectrin repeat-containing protein 1	Q80UK0
Set	0.0052	0.05	0.82	0.58	0.0080	0.30	0.17	0.63	0.0180	Protein SET (Fragment)	A2BE93
Sez6l	0.0000	-0.29	0.98	-0.22	0.83	1.18	0.0460	-0.51	0.68	Seizure 6-like protein	A0A0G2JG23
Sez6l2	0.0002	-0.24	0.48	-0.08	0.94	0.24	0.39	-0.32	0.69	Seizure 6-like protein 2	Q4V9Z5
Sf1	0.0082	0.32	0.58	0.35	0.49	-0.16	0.72	0.67	0.052	Splicing factor 1 (Fragment)	D3YVH4
Sf3a1	-1.1800	-0.67	0.081	0.84	0.0120	-0.12	0.83	0.17	0.52	Splicing factor 3A subunit 1	Q8K4Z5
Sf3a3	-0.2599	-0.73	0.38	0.92	0.12	-0.50	0.56	0.19	0.55	Splicing factor 3A subunit 3	Q9D554
Sf3b1	0.0002	0.04	0.88	0.26	0.49	-0.17	0.34	0.31	0.14	Splicing factor 3B subunit 1	Q99NB9
Sf3b2	-0.0026	0.53	0.54	-0.17	0.78	0.18	0.88	0.36	0.74	Splicing factor 3b, subunit 2	Q3UJB0
Sf3b3	-0.2642	-0.49	0.22	0.71	0.07	-0.10	0.64	0.21	0.48	Splicing factor 3B subunit 3	Q921M3
Sfpq	-0.3985	0.81	0.096	-0.78	0.24	0.02	0.31	0.03	0.35	Splicing factor, proline- and glutamine-rich	Q8VIJ6
Sfxn1	0.1251	-0.41	0.67	-1.98	0.13	0.30	0.81	-2.39	0.068	Sideroflexin-1	Q99JR1
Sfxn3	-10.2316	0.80	0.0029	-1.26	0.0001	0.17	0.56	-0.46	0.087	Sideroflexin-3	Q91V61
Sfxn5	-0.8769	0.31	0.071	-0.69	0.0003	0.07	0.28	-0.38	0.0010	Sideroflexin-5	Q925N0
Sgip1	-0.0393	0.50	0.3	-0.33	0.35	0.36	0.61	0.18	0.74	SH3-containing GRB2-like protein 3-interacting protein 1	Q8VD37
Sh3bgrl3	0.0002	-0.47	0.99	-0.28	0.47	0.21	0.56	-0.75	0.6	SH3 domain-binding glutamic acid-rich-like protein 3	Q91VW3
Sh3bp1	-0.2724	-0.59	0.054	0.38	0.11	-0.14	0.56	-0.21	0.4	SH3 domain-binding protein 1	P55194
Sh3d19	-0.5646	0.72	0.0490	-0.49	0.06	0.52	0.58	0.23	0.71	SH3 domain-containing protein 19	A0A571BEF4
Sh3gl2	-0.3482	-0.10	0.092	0.84	0.0001	0.13	0.37	0.75	0.0001	Endophilin-A1	A2ALV3
Sh3gl3	0.0380	0.13	0.35	0.40	0.0250	0.05	0.79	0.53	0.0001	Endophilin-A3	A0A0R4J0B8
Sh3glb1	0.0000	-0.09	0.8	-0.10	0.89	0.49	0.47	-0.20	0.77	Endophilin-B1	A0A0G2JEC4
Sh3glb2	-0.0060	0.20	0.54	-0.26	0.37	-0.17	0.31	-0.06	0.75	Endophilin-B2	A2AWI7
Sh3kbp1	-0.2605	0.70	0.22	-0.76	0.18	-0.01	0.85	-0.07	0.9	SH3 domain-containing kinase-binding protein 1	Q8R550
Shank1	-28.4520	1.44	0.0220	-2.98	0.0001	-0.86	0.0070	-1.54	0.0001	SH3 and multiple ankyrin repeat domains protein 1	D3YZU1

Table S2: Differentially expressed proteins in cerebral cortex *(continued)*

gene	$\pi_{TW/EW} \times \pi_{TF/TW}$	log2FC TW/EW	padj TW/EW	log2FC TF/TW	padj TF/TW	log2FC EF/EW	padj EF/EW	log2FC TF/EW	padj TF/EW	description	accession
Shank2	-1.1629	0.90	0.56	-2.23	0.0050	-0.67	0.54	-1.33	0.0050	SH3 and multiple ankyrin repeat domains protein 2	D3Z5K8
Shank3	-6.0793	1.10	0.23	-2.54	0.0004	-0.70	0.11	-1.44	0.0010	SH3 and multiple ankyrin repeat domains protein 3	Q4ACU6
Shf	-0.0130	0.25	0.29	-0.19	0.31	-0.08	0.82	0.06	0.75	SH2 domain-containing adapter protein F (Fragment)	A2AQ89
Shfl	-0.0077	0.35	0.43	-0.39	0.7	-0.17	0.77	-0.05	0.64	Shiftless antiviral inhibitor of ribosomal frameshifting protein homolog	Q8CAK3
Shisa7	-2.3003	0.91	0.34	-2.25	0.0040	-0.09	0.84	-1.34	0.0050	Protein shisa-7 (Fragment)	F6T9T6
Shisa9	-0.3895	0.60	0.17	-0.88	0.11	0.63	0.41	-0.29	0.85	Protein shisa-9	Q9CZN4
Shmt2	-0.0125	-0.20	0.44	0.25	0.2	0.06	0.96	0.06	0.67	Serine hydroxymethyltransferase, mitochondrial	Q9CZN7
Shoc2	-0.2992	1.28	0.37	-1.22	0.36	-3.67	0.47	0.06	0.75	Leucine-rich repeat protein SHOC-2	O88520
Sik3	-0.1544	0.34	0.44	-0.82	0.0280	0.33	0.0280	-0.48	0.0280	Non-specific serine/threonine protein kinase	E9PU87
Sipa1l1	-12.7888	2.18	0.068	-2.88	0.0180	1.28	0.0260	-0.70	0.48	Signal-induced proliferation-associated 1-like protein 1	Q4VBF8
Sipa1l2	-13.7901	5.61	0.17	-5.46	0.26	0.27	0.68	0.15	0.62	Signal-induced proliferation-associated 1-like protein 2	A0A1D5RLY9
Sipa1l3	-0.0438	0.36	0.7	-1.09	0.19	0.37	0.28	-0.73	0.093	Signal-induced proliferation-associated 1-like protein 3	G3X9J0
Sirpa	0.0000	0.06	0.54	-0.06	0.89	0.60	0.0400	0.00	0.57	Tyrosine-protein phosphatase non-receptor type substrate 1	P97797
Sirt2	0.5782	-0.67	0.0006	-0.47	0.27	-0.42	0.0009	-1.14	0.0001	NAD-dependent protein deacetylase	A0A140LHL5
Slc12a2	-0.0032	-0.30	0.33	0.12	0.65	-0.04	0.74	-0.18	0.74	Solute carrier family 12 member 2	E9QM38
Slc12a5	0.0007	-0.14	0.49	-0.04	0.41	-0.17	0.96	-0.18	0.91	KCC2a-S25 variant 1	A0A076FR46
Slc17a7	0.0020	-0.18	0.79	-0.19	0.26	-0.22	0.3	-0.37	0.24	Vesicular glutamate transporter 1	Q3TXX4
Slc1a2	0.0000	-0.05	0.74	0.03	0.87	-0.11	0.46	-0.01	0.68	Excitatory amino acid transporter 2	P43006
Slc1a3	-0.0002	-0.06	0.69	0.07	0.55	-0.58	0.21	0.00	0.94	Excitatory amino acid transporter 1	P56564
Slc25a1	-0.0113	0.08	0.64	-0.47	0.0280	0.39	0.36	-0.40	0.0280	Tricarboxylate transport protein, mitochondrial	Q8JZU2
Slc25a10	-0.8041	0.40	0.53	-2.89	0.0030	0.10	0.58	-2.49	0.0130	Mitochondrial dicarboxylate carrier	Q9QZD8
Slc25a11	-4.3031	0.74	0.0280	-1.00	0.0002	0.31	0.24	-0.27	0.25	Mitochondrial 2-oxoglutarate/malate carrier protein	Q9CR62
Slc25a12	-7.2647	0.73	0.0001	-0.66	0.0002	0.21	0.25	0.06	0.42	Calcium-binding mitochondrial carrier protein Aralar1	Q8BH59
Slc25a18	-2.3382	0.32	0.0250	-1.26	0.0002	-0.31	0.2	-0.94	0.0010	Mitochondrial glutamate carrier 2	Q9DB41
Slc25a19	-0.3489	3.32	0.5	-3.41	0.79	3.81	0.34	-0.09	0.78	Mitochondrial thiamine pyrophosphate carrier	Q9DAM5

Table S2: Differentially expressed proteins in cerebral cortex *(continued)*

gene	$\pi_{TW/EW} \times \pi_{TF/TW}$	log2FC TW/EW	padj TW/EW	log2FC TF/TW	padj TF/TW	log2FC EF/EW	padj EF/EW	log2FC TF/EW	padj TF/EW	description	accession
Slc25a20	-0.1387	0.39	0.29	-0.69	0.11	-0.09	0.7	-0.30	0.35	Mitochondrial carnitine/acylcarnitine carrier protein	Q9Z2Z6
Slc25a22	-0.1696	0.44	0.23	-0.63	0.11	0.30	0.28	-0.19	0.6	Mitochondrial glutamate carrier 1	Q9D6M3
Slc25a23	-0.0010	0.15	0.81	-0.20	0.45	0.16	1	-0.05	0.64	Calcium-binding mitochondrial carrier protein SCaMC-3	Q6GQS1
Slc25a3	-0.5023	0.31	0.0430	-0.47	0.0030	0.36	0.0300	-0.17	0.6	Phosphate carrier protein, mitochondrial	G5E902
Slc25a4	-4.7523	0.62	0.0020	-0.71	0.0001	0.00	0.46	-0.09	0.34	ADP/ATP translocase 1	P48962
Slc25a46	-0.0001	0.39	0.93	-0.19	0.89	0.38	0.26	0.19	0.92	Solute carrier family 25 member 46	A0A3Q4L2U5
Slc25a51	-0.2402	0.65	0.2	-0.71	0.18	0.40	0.51	-0.07	0.86	Mitochondrial nicotinamide adenine dinucleotide transporter SLC25A51 (Fragment)	G3UYJ3
Slc27a4	0.0014	0.28	0.16	0.06	0.79	0.17	0.5	0.34	0.062	Long-chain fatty acid transport protein 4	Q91VE0
Slc2a1	-0.0001	0.03	0.84	-0.13	0.67	0.38	0.06	-0.11	0.73	Solute carrier family 2, facilitated glucose transporter member 1	P17809
Slc2a3	0.0272	-0.64	0.055	-0.10	0.46	-0.20	0.7	-0.74	0.0280	Solute carrier family 2, facilitated glucose transporter member 3	P32037
Slc32a1	-0.1725	0.52	0.095	-0.45	0.19	0.59	0.28	0.08	0.91	Vesicular inhibitory amino acid transporter	O35633
Slc3a2	0.0821	-0.11	0.2	-0.37	0.0013	-0.24	0.0440	-0.47	0.0009	4F2 cell-surface antigen heavy chain	P10852
Slc4a1	0.0714	-0.65	0.0020	-0.08	0.31	-0.69	0.0016	-0.73	0.0009	Band 3 anion transport protein	P04919
Slc4a10	-0.0013	-0.10	0.82	0.42	0.44	0.42	0.26	0.32	0.15	Sodium-driven chloride bicarbonate exchanger	Q5DTL9
Slc4a3	0.0314	1.90	0.49	0.47	0.77	1.29	0.33	2.38	0.14	Anion exchange protein 3	P16283
Slc4a4	0.0001	0.04	0.91	0.24	0.61	-0.50	0.0280	0.28	0.31	Anion exchange protein	E9Q8N8
Slc4a7	-1.5308	-0.77	0.054	1.01	0.0280	0.58	0.32	0.24	0.42	Anion exchange protein	J9RV59
Slc6a11	-0.0806	0.25	0.42	-0.70	0.06	0.06	0.97	-0.45	0.0490	Sodium- and chloride-dependent GABA transporter 3	P31650
Slc8a1	0.0000	0.03	0.85	0.01	0.98	-0.21	0.28	0.04	0.7	Na(+)/Ca(2+)-exchange protein 1	G3X9J1
Slc8a2	0.0000	-0.12	0.58	0.06	0.95	-0.03	0.81	-0.06	0.51	Sodium/calcium exchanger 2	Q8K596
Slc9a3r1	0.0514	0.60	0.1	0.32	0.54	0.36	0.32	0.92	0.0390	Na(+)/H(+) exchange regulatory cofactor NHE-RF1	P70441
Slk	-0.1264	0.67	0.0110	-0.16	0.25	0.50	0.07	0.51	0.08	STE20-like serine/threonine-protein kinase	O54988
Slmap	0.0000	-0.02	0.9	-0.11	0.54	0.08	0.8	-0.13	0.5	Sarcolemmal membrane-associated protein	H7BX64
Smarca5	-0.0301	0.38	0.79	-1.36	0.27	-1.42	0.28	-0.98	0.37	SWI/SNF-related matrix-associated actin-dependent regulator of chromatin subfamily A member 5	Q91ZW3
Smarcc2	0.0000	-0.04	0.97	-0.16	0.87	-0.32	0.39	-0.20	0.57	SWI/SNF complex subunit SMARCC2	Q3UID0
Smc1a	0.0004	0.19	0.92	0.69	0.83	-3.64	0.57	0.88	0.83	Structural maintenance of chromosomes protein 1A	Q9CU62

Table S2: Differentially expressed proteins in cerebral cortex *(continued)*

gene	$\pi_{TW/EW} \times \pi_{TF/TW}$	log2FC TW/EW	padj TW/EW	log2FC TF/TW	padj TF/TW	log2FC EF/EW	padj EF/EW	log2FC TF/EW	padj TF/EW	description	accession
Smc3	0.0000	0.24	0.28	0.02	0.99	0.06	0.9	0.26	0.25	Structural maintenance of chromosomes protein 3	Q9CW03
Smdt1	0.0315	0.29	0.65	0.83	0.2	0.11	0.87	1.12	0.056	Essential MCU regulator, mitochondrial	Q9DB10
Snap25	-0.0040	-0.19	0.53	0.26	0.51	0.04	0.61	0.07	0.71	Synaptosomal-associated protein 25	P60879
Snap47	-0.0047	0.42	0.39	-0.24	0.77	-0.27	0.81	0.17	0.51	Synaptosomal-associated protein 47	Q8R570
Snap91	-0.0297	-0.04	0.66	1.03	0.0001	0.00	0.64	0.99	0.0001	Clathrin coat assembly protein AP180	A0A5F8MPL3
Snca	0.0330	-0.43	0.39	-0.22	0.14	-0.40	0.11	-0.66	0.0230	Alpha-synuclein	O55042
Sncb	0.0424	-0.36	0.064	-0.15	0.22	-0.35	0.064	-0.50	0.0080	Beta-synuclein	Q91ZZ3
Snd1	0.5091	0.33	0.11	0.44	0.0002	-0.07	0.63	0.77	0.0003	Staphylococcal nuclease domain-containing protein 1	Q78PY7
Snph	-0.0141	0.13	0.94	-2.25	0.0160	-1.88	0.14	-2.12	0.0100	Syntaphilin	Q80U23
Snrk	-1.7482	0.98	0.14	-1.75	0.064	-0.61	0.35	-0.77	0.41	SNF-related serine/threonine-protein kinase	Q8VDU5
Snrnp70	-0.4984	-0.51	0.0320	0.71	0.12	-0.47	0.0340	0.20	0.98	U1 small nuclear ribonucleoprotein 70 kDa	Q62376
Snrpa	0.1513	-0.84	0.65	-2.70	0.44	-1.36	0.52	-3.54	0.22	U1 small nuclear ribonucleoprotein A (Fragment)	D3Z0S6
Snrpa1	-0.7507	-0.70	0.0260	0.57	0.065	-0.50	0.0410	-0.13	0.63	U2 small nuclear ribonucleoprotein A'	P57784
Snrpd1	-2.1251	-0.88	0.0420	0.93	0.0130	-0.09	0.68	0.05	0.81	Small nuclear ribonucleoprotein Sm D1	P62315
Snrpd3	-0.0034	-0.38	0.72	0.41	0.7	-0.29	0.86	0.03	0.96	Small nuclear ribonucleoprotein Sm D3	P62320
Snrpe	-0.0014	0.78	0.69	-0.36	0.93	-1.18	0.35	0.42	0.71	Small nuclear ribonucleoprotein E	P62305
Snta1	0.0124	0.08	0.53	0.28	0.0100	0.16	0.064	0.36	0.0001	Alpha-1-syntrophin	A2AKD7
Sntb1	0.0035	-0.12	0.6	-0.33	0.4	-0.06	0.88	-0.45	0.26	Beta-1-syntrophin	Q99L88
Snu13	0.0000	-0.06	0.98	0.16	1	0.30	0.22	0.10	0.63	NHP2-like protein 1	Q9D0T1
Snx1	-0.0823	0.65	0.46	-0.97	0.41	0.34	0.72	-0.32	0.91	Sorting nexin-1	Q6NZD2
Snx12	0.3263	-0.26	0.0180	-0.30	0.0040	-0.15	0.17	-0.56	0.0001	Sorting nexin-12	Q3TGS7
Snx15	0.1923	-0.41	0.67	-4.35	0.24	-3.13	0.87	-4.76	0.43	Sorting nexin-15	D3Z479
Snx2	-0.0127	0.22	0.54	-0.30	0.19	0.30	0.75	-0.08	0.7	Sorting nexin-2	Q9CWK8
Snx27	-0.0017	0.13	0.8	-0.27	0.32	-0.35	0.29	-0.14	0.47	Sorting nexin-27	Q3UHD6
Snx3	0.1992	-0.25	0.079	-0.42	0.0190	-0.37	0.098	-0.67	0.0001	Sorting nexin-3	Q78ZM0
Snx5	0.0001	0.07	0.85	0.11	0.57	0.05	0.72	0.18	0.69	Sorting nexin-5	Q9D8U8
Snx6	0.0002	0.02	0.9	0.24	0.12	0.03	0.67	0.26	0.094	Sorting nexin-6	A0A1W2P701
Snx9	-0.9590	5.02	0.0280	-0.52	0.58	2.37	0.54	4.50	0.21	Sorting nexin-9	Q91VH2
Sod1	0.0002	0.02	0.89	0.28	0.19	0.12	0.42	0.30	0.17	Superoxide dismutase [Cu-Zn]	P08228
Soga1	-0.0028	0.10	0.92	-3.64	0.61	0.10	0.95	-3.54	0.68	Protein SOGA1	A2ACV6
Soga3	0.0000	-0.06	0.93	-0.18	1	-0.12	0.45	-0.24	0.0060	Protein SOGA3	Q6NZL0
Sorbs1	-4.9849	1.80	0.13	-2.45	0.053	0.49	0.56	-0.66	0.56	Sorbin and SH3 domain-containing protein 1	A0A286YCI8
Sorbs2	-8.2492	1.39	0.053	-1.94	0.0040	0.28	0.62	-0.55	0.27	Sorbin and SH3 domain-containing protein 2	B7ZWM6

Table S2: Differentially expressed proteins in cerebral cortex *(continued)*

gene	$\pi_{TW/EW} \times \pi_{TF/TW}$	log2FC TW/EW	padj TW/EW	log2FC TF/TW	padj TF/TW	log2FC EF/EW	padj EF/EW	log2FC TF/EW	padj TF/EW	description	accession
Sorcs1	0.0002	-0.30	0.65	-0.02	0.66	-0.40	0.6	-0.32	0.37	VPS10 domain-containing receptor SorCS1	A0A1B0GRB7
Sorcs2	0.0014	0.23	0.19	0.09	0.8	-0.12	0.67	0.33	0.16	VPS10 domain-containing receptor SorCS2	Q9EPR5
Sorl1	-8.0493	6.13	0.11	-1.78	0.17	3.64	0.75	4.35	0.71	Sortilin-related receptor	O88307
Sos1	0.3269	1.00	0.16	0.40	0.094	0.39	0.49	1.40	0.0190	Son of sevenless homolog 1	Q62245
Sowaha	0.0007	-0.03	0.94	-0.96	0.14	-0.84	0.43	-0.99	0.0280	Ankyrin repeat domain-containing protein SOWAHA	Q8BLS7
Spast	-7.5642	1.58	0.0220	-1.70	0.0200	0.73	0.47	-0.12	0.84	Spastin	A0A286YE25
Spata2l	-0.3943	0.85	0.48	-1.26	0.07	-0.05	0.46	-0.41	0.31	Spermatogenesis-associated protein 2-like protein	Q8BNN1
Spcs2	0.0343	0.42	0.23	0.39	0.47	-0.12	0.93	0.81	0.21	Microsomal signal peptidase 25 kDa subunit	A0A140LHG8
Specc1	-0.2431	0.50	0.062	-0.42	0.11	0.06	0.86	0.08	0.63	Cytospin-B	A0A0J9YTU3
Speg	-0.3456	0.28	0.56	-2.13	0.0050	-0.18	0.47	-1.85	0.0070	Striated muscle-specific serine/threonine-protein kinase	Q62407
Spg7	-0.0173	0.42	0.3	-0.27	0.51	-0.12	0.67	0.14	0.45	Paraplegin	D3YZN4
Sphk2	-0.0332	0.62	0.33	-0.47	0.58	0.30	0.37	0.15	0.56	Sphingosine kinase 2	Q9JIA7
Sphkap	-1.3082	1.23	0.32	-1.94	0.078	0.42	0.62	-0.71	0.2	A-kinase anchor protein SPHKAP	E9PUC4
Spire1	-0.1624	0.50	0.74	-2.32	0.085	0.71	0.14	-1.83	0.091	Protein spire homolog 1	A0A5H1ZRL1
Spon1	0.0010	0.04	0.91	1.27	0.34	-0.27	0.94	1.31	0.23	Spondin-1	Q8VCC9
Spr	0.0000	-0.12	0.93	0.04	0.72	-0.38	0.28	-0.08	0.86	Sepiapterin reductase	G3UXX3
Spta1	0.2655	-0.15	0.31	-0.87	0.0001	0.56	0.0450	-1.02	0.0010	Spectrin alpha chain, erythrocytic 1	P08032
Sptan1	-4.2336	0.42	0.0001	-0.63	0.0001	0.56	0.0004	-0.20	0.0054	Spectrin alpha chain, non-erythrocytic 1	A3KGU9
Sptb	0.0008	-0.10	0.99	-0.75	0.0030	0.59	0.05	-0.86	0.0010	Spectrin beta chain	Q3UGX2
Sptbn1	-4.0704	0.48	0.0001	-0.53	0.0001	0.65	0.0001	-0.05	0.8	Spectrin beta chain, non-erythrocytic 1	Q62261
Sptbn2	-8.5376	0.58	0.0001	-0.92	0.0001	0.49	0.0002	-0.34	0.0050	Spectrin beta chain	Q68FG2
Sptbn4	-0.4787	0.43	0.13	-0.71	0.0170	0.30	0.28	-0.28	0.37	Spectrin beta chain	E9PX29
Src	-0.0003	0.04	0.64	-0.11	0.39	0.00	0.91	-0.08	0.94	Neuronal proto-oncogene tyrosine-protein kinase Src	P05480
Srcin1	-3.5829	4.06	0.4	-4.36	0.31	1.88	0.44	-0.31	1	SRC kinase-signaling inhibitor 1	A0A571BDG0
Srgap2	0.0016	0.23	0.73	0.29	0.66	0.90	0.0440	0.53	0.56	SLIT-ROBO Rho GTPase-activating protein 2	Q91Z67
Srgap3	-18.2282	2.03	0.0001	-1.25	0.0160	0.38	0.57	0.77	0.085	SLIT-ROBO Rho GTPase-activating protein 3	E9QN14
Srm	0.1648	-1.02	0.58	-2.98	0.59	-0.05	0.69	-4.00	0.22	Spermidine synthase	Q64674
Srp54	-0.0254	-0.18	0.85	0.74	0.0020	0.24	0.18	0.56	0.0017	Signal recognition particle 54 kDa protein	P14576
Srp72	0.0027	0.94	0.0028	0.05	0.95	0.38	0.14	0.99	0.0035	Signal recognition particle subunit SRP72	E9Q740
Srpk2	-0.0308	0.35	0.34	-0.39	0.33	-0.16	0.69	-0.04	0.96	SRSF protein kinase 2	A0A0R4J124

Table S2: Differentially expressed proteins in cerebral cortex *(continued)*

gene	$\pi_{TW/EW}\times$ $\pi_{TF/TW}$	log2FC TW/EW	padj TW/EW	log2FC TF/TW	padj TF/TW	log2FC EF/EW	padj EF/EW	log2FC TF/EW	padj TF/EW	description	accession
Srrm2	0.3132	0.70	0.25	0.64	0.069	0.15	0.66	1.34	0.0040	Serine/arginine repetitive matrix protein 2	Q8BTI8
Srsf1	-0.1455	0.50	0.7	-1.60	0.067	-1.81	0.085	-1.11	0.14	Serine/arginine-rich splicing factor 1	H7BX95
Srsf2	0.0000	-0.26	0.72	-0.02	1	-0.74	0.13	-0.27	0.54	Serine/arginine-rich splicing factor 2	Q62093
Srsf3	-0.7034	0.92	0.19	-1.06	0.1	-1.54	0.0035	-0.14	0.41	Serine/arginine-rich splicing factor 3	P84104
Srsf4	-0.8034	8.40	0.18	-0.64	0.63	4.30	0.33	7.76	0.24	Serine/arginine-rich-splicing factor 4	Q542V3
Srsf7	-0.0221	0.64	0.76	-0.79	0.43	-1.07	0.05	-0.15	0.4	Serine/arginine-rich-splicing factor 7	A0A3Q4L393
Ssb	-0.0255	0.36	0.79	-0.96	0.19	-1.39	0.0110	-0.60	0.33	Lupus La protein homolog	P32067
Ssbp1	-0.2843	0.28	0.1	-0.77	0.0480	-0.01	0.87	-0.49	0.17	Single-stranded DNA-binding protein, mitochondrial	Q9CYR0
Ssr3	3.5064	5.54	0.0280	0.46	0.13	4.23	0.19	6.00	0.0280	Translocon-associated protein subunit gamma	Q9DCF9
Ssr4	0.0381	0.32	0.13	0.32	0.38	0.06	0.81	0.65	0.062	Translocon-associated protein subunit delta	Q62186
Sst	-0.0062	0.08	0.87	-0.75	0.0190	-0.41	0.1	-0.67	0.0053	Somatostatin	P60041
St13	0.0039	0.09	0.65	0.25	0.12	0.21	0.96	0.35	0.0310	Hsc70-interacting protein	F8WJK8
Stambp	-0.0093	0.31	0.26	-0.23	0.6	-0.50	0.059	0.08	0.57	STAM-binding protein	Q9CQ26
Stau2	-0.0022	0.17	0.65	-0.28	0.57	0.50	0.48	-0.11	0.81	Double-stranded RNA-binding protein Staufen homolog 2	E2QRQ3
Stim2	-4.1109	2.23	0.085	-1.87	0.12	-0.29	0.8	0.36	0.51	Stromal interaction molecule 2	I1E4X8
Stip1	-0.0098	0.18	0.21	-0.08	0.1	0.01	0.77	0.10	0.71	Stress-induced-phosphoprotein 1	Q60864
Stk24	-0.0140	1.06	0.23	-0.24	0.82	0.84	0.31	0.81	0.45	Serine/threonine-protein kinase 24	Q99KH8
Stmn1	-0.0013	-0.37	0.38	0.31	0.94	-0.09	0.71	-0.07	0.34	Stathmin	P54227
Stmn3	0.0000	-0.20	0.7	-0.13	0.99	0.00	0.77	-0.33	0.85	Stathmin-3	O70166
Stmn4	-0.0788	0.31	0.087	-0.25	0.11	-0.02	0.85	0.06	0.56	Stathmin-4	P63042
Stoml2	0.0006	0.18	0.88	0.27	0.58	0.04	0.83	0.45	0.51	Stomatin-like protein 2, mitochondrial	Q99JB2
Strap	-0.0018	-0.09	0.69	0.25	0.31	0.04	0.74	0.16	0.57	Serine-threonine kinase receptor-associated protein	Q9Z1Z2
Strbp	-0.0001	-0.03	0.93	0.83	0.69	0.23	0.74	0.80	0.31	Spermatid perinuclear RNA-binding protein	Q91WM1
Strip1	-0.0002	0.01	0.79	-0.40	0.26	-0.27	0.65	-0.39	0.49	Striatin-interacting protein 1	Q8C079
Strn	-0.3280	1.23	0.17	-0.76	0.35	0.77	0.25	0.48	0.57	Striatin	O55106
Strn3	-0.3552	0.29	0.28	-1.07	0.0085	0.25	0.62	-0.79	0.25	Striatin-3	B2RQS1
Strn4	0.0003	-0.02	0.85	-0.28	0.15	-0.26	0.26	-0.30	0.086	Striatin-4	P58404
Stub1	0.0001	-0.02	0.75	-0.09	0.32	0.20	0.092	-0.11	0.72	STIP1 homology and U box-containing protein 1	A0A494BBF6
Stum	-0.0854	-0.37	0.25	0.40	0.11	0.18	0.54	0.03	0.97	Protein stum homolog	J3QP43
Stx12	0.0022	0.04	0.39	0.29	0.34	0.62	0.22	0.33	0.4	Syntaxin-12	Q9ER00
Stx1b	0.0001	0.05	0.2	0.23	0.96	0.21	0.28	0.27	0.26	Syntaxin-1B	P61264
Stx6	0.0000	-0.01	0.91	0.19	0.78	0.49	0.66	0.18	0.86	Syntaxin-6	A0A0A6YXG0

Table S2: Differentially expressed proteins in cerebral cortex *(continued)*

gene	$\pi_{TW/EW} \times \pi_{TF/TW}$	log2FC TW/EW	padj TW/EW	log2FC TF/TW	padj TF/TW	log2FC EF/EW	padj EF/EW	log2FC TF/EW	padj TF/EW	description	accession
Stx7	0.0323	0.36	0.35	0.29	0.21	0.37	0.24	0.65	0.084	Syntaxin-7	O70439
Stxbp1	0.0004	0.01	0.55	0.29	0.32	-0.11	0.12	0.30	0.17	Syntaxin-binding protein 1	O08599
Stxbp3	0.0073	-0.27	0.47	-0.30	0.53	-0.89	0.075	-0.57	0.16	Syntaxin-binding protein 3	Q60770
Stxbp5	-0.0002	-0.02	0.88	0.55	0.48	0.71	0.0170	0.53	0.0380	Syntaxin-binding protein 5	Q8K400
Stxbp6	0.0000	0.18	0.9	-0.07	0.97	0.60	0.63	0.10	0.94	Syntaxin-binding protein 6	Q3TYA4
Sucla2	-1.4976	0.24	0.0010	-0.52	0.0001	0.09	0.93	-0.28	0.0001	Succinate–CoA ligase [ADP-forming] subunit beta, mitochondrial	Q9Z2I9
Suclg1	-1.9588	0.38	0.0060	-0.58	0.0001	0.33	0.0180	-0.20	0.13	Succinate–CoA ligase [ADP/GDP-forming] subunit alpha, mitochondrial	Q9WUM5
Sumo1	-0.0013	-0.38	0.45	0.08	0.76	-0.14	0.77	-0.29	0.55	Small ubiquitin-related modifier 1	P63166
Sumo3	-0.4156	-0.75	0.0010	0.24	0.17	-0.24	0.0410	-0.51	0.0010	Small ubiquitin-related modifier 3	G3UWI9
Susd2	0.0253	0.28	0.41	0.41	0.27	0.42	0.2	0.69	0.087	Sushi domain-containing protein 2	Q9DBX3
Sv2a	0.0000	0.01	0.81	-0.02	0.83	0.01	0.91	-0.01	0.98	Synaptic vesicle glycoprotein 2A	Q9JIS5
Sv2b	0.0000	-0.23	0.65	-0.03	0.92	-0.07	0.9	-0.26	0.43	Synaptic vesicle glycoprotein 2B	Q8BG39
Svip	-0.0471	-0.76	0.086	0.30	0.64	-0.98	0.11	-0.46	0.5	Small VCP/p97-interacting protein	Q3UZP4
Syn1	0.0000	0.00	0.0150	-0.08	0.0040	0.16	0.55	-0.08	0.98	Synapsin-1	O88935
Syn2	-0.0158	0.06	0.3	-0.28	0.0160	-0.21	0.39	-0.22	0.34	Synapsin-2	Q64332
Syn3	-21.4623	5.27	0.0150	-2.52	0.13	3.99	0.19	2.75	0.39	Synapsin-3	Q8JZP2
Syncrip	-0.4156	0.76	0.39	-1.00	0.0460	0.17	0.83	-0.24	0.52	Heterogeneous nuclear ribonucleoprotein Q	A0A0R4J259
Syne1	-0.4927	0.28	0.0410	-0.47	0.0020	0.11	0.18	-0.19	1	Nesprin-1	A0A1L1STC6
Syne2	-0.0120	-0.19	0.5	0.32	0.22	0.18	0.068	0.13	0.17	Nesprin-2	E9QP46
Syngap1	-35.8968	2.76	0.089	-5.38	0.0050	-2.18	0.47	-2.62	0.19	Ras/Rap GTPase-activating protein SynGAP	F6SEU4
Syngr1	0.0000	-0.48	0.56	0.00	0.86	0.09	0.7	-0.49	0.55	Synaptogyrin-1	O55100
Syngr3	0.0020	-0.15	0.15	-0.08	0.63	-0.06	0.69	-0.22	0.0290	Synaptogyrin-3	Q8R191
Synj1	-47.7789	7.06	0.0120	-5.52	0.23	1.70	0.085	1.54	0.084	Phosphoinositide 5-phosphatase	D3Z656
Synpo	-4.3430	0.95	0.0250	-1.19	0.0040	0.98	0.0080	-0.24	0.57	Synaptopodin	E9Q3E2
Syp	0.0015	-0.15	0.91	-0.32	0.17	-0.04	0.82	-0.48	0.24	Synaptophysin	Q62277
Syt1	-0.0266	0.55	0.3	-0.12	0.17	0.33	0.67	0.43	0.55	Synaptotagmin-1	P46096
Syt11	0.0029	-0.11	0.81	-0.50	0.27	0.10	0.47	-0.60	0.079	Synaptotagmin-11	Q9R0N3
Syt17	-0.0002	-0.14	0.74	0.10	0.8	0.58	0.21	-0.04	1	Synaptotagmin XVII (Fragment)	A0A140LJ89
Syt3	-0.1509	-0.29	0.17	0.54	0.056	0.44	0.062	0.24	0.27	Synaptotagmin-3	G3X9Y1
Syt7	-5.7090	2.27	0.16	-3.16	0.1	0.79	0.66	-0.89	0.61	Synaptotagmin-7	A0A494BB44
Tagln3	-0.1965	0.16	0.61	-1.43	0.0001	-0.09	0.53	-1.27	0.0010	Transgelin-3	Q9R1Q8
Taldo1	-0.0379	-0.42	0.0007	0.07	0.39	-0.22	0.07	-0.35	0.0010	Transaldolase	A0A1B0GR11
Tanc2	-19.9681	1.44	0.0270	-2.21	0.0001	-0.37	0.32	-0.77	0.0440	Protein TANC2	A2A690

Table S2: Differentially expressed proteins in cerebral cortex *(continued)*

gene	$\pi_{TW/EW} \times \pi_{TF/TW}$	log2FC TW/EW	padj TW/EW	log2FC TF/TW	padj TF/TW	log2FC EF/EW	padj EF/EW	log2FC TF/EW	padj TF/EW	description	accession
Taok2	-0.2081	0.87	0.53	-1.40	0.24	-0.19	0.97	-0.53	0.42	Serine/threonine-protein kinase TAO2	Q6ZQ29
Tardbp	0.0018	0.43	0.37	0.12	0.83	1.03	0.11	0.55	0.23	TAR DNA-binding protein 43	Q921F2
Tars1	-0.0166	0.18	0.22	-0.22	0.23	0.00	0.67	-0.04	0.74	Threonine–tRNA ligase 1, cytoplasmic	Q9D0R2
Tars2	-0.0927	0.33	0.52	-0.98	0.098	-0.04	0.87	-0.65	0.1	Threonine–tRNA ligase, mitochondrial	Q3UQ84
Tars3	-0.0933	0.65	0.55	-1.00	0.28	-0.08	0.96	-0.34	0.47	Threonine–tRNA ligase 2, cytoplasmic	Q8BLY2
Tbc1d10b	-0.0319	0.37	0.16	-0.18	0.25	-0.23	0.63	0.18	0.58	TBC1 domain family member 10B	Q8BHL3
Tbc1d24	-0.0006	0.03	0.94	-0.91	0.16	0.07	0.77	-0.88	0.22	TBC1 domain family member 24	Q3UUG6
Tbca	-2.2125	0.96	0.69	-9.21	0.0280	0.15	0.59	-8.24	0.0280	Tubulin-specific chaperone A	P48428
Tbcb	0.0390	0.89	0.54	0.56	0.51	0.95	0.36	1.45	0.21	Tubulin-folding cofactor B	Q9D1E6
Tbck	0.0000	0.13	0.96	-0.14	0.77	-0.08	0.84	-0.01	0.93	TBC domain-containing protein kinase-like protein	E9Q1W7
Tbl2	-0.0195	0.23	0.14	-0.19	0.3	-0.30	0.58	0.04	0.7	Transducin beta-like protein 2	Q9R099
Tcea1	0.0045	-0.19	0.66	-0.70	0.65	-0.34	0.6	-0.90	0.56	Transcription elongation factor A protein 1	P10711
Tceal5	0.0002	0.04	0.9	0.21	0.35	0.29	0.47	0.25	0.78	Transcription elongation factor A protein-like 5	Q8CCT4
Tcerg1	-0.0003	-0.21	0.87	0.31	0.83	0.09	0.6	0.10	0.96	Transcription elongation regulator 1	Q8CGF7
Tcf25	-0.4828	0.81	0.088	-0.54	0.09	-0.27	0.81	0.27	0.71	Nuclear localized protein-1 isoform d (Fragment)	B2ZAC8
Tcof1	0.0258	0.45	0.16	0.22	0.47	0.36	0.056	0.67	0.0040	Treacle protein	H3BL37
Tcp1	-0.0003	-0.14	0.93	0.11	0.28	0.00	0.9	-0.03	0.33	T-complex protein 1 subunit alpha	P11983
Tdrkh	-1.3181	4.41	0.0060	-0.46	0.51	0.36	0.72	3.95	0.0300	Tudor and KH domain-containing protein	A0A0G2JFB2
Tecpr1	-0.0216	-0.33	0.089	0.17	0.43	0.40	0.14	-0.15	0.5	Tectonin beta-propeller repeat-containing protein 1	Q80VP0
Tecr	-0.5221	0.52	0.0100	-0.43	0.068	-0.09	0.62	0.09	0.47	Very-long-chain enoyl-CoA reductase	A0A5F8MQC8
Tenm1	-0.1577	1.07	0.41	-1.01	0.42	-0.68	0.68	0.06	0.9	Teneurin-1	A2ANL9
Tenm2	-0.0396	0.35	0.26	-0.37	0.3	-0.08	0.84	-0.02	0.91	Teneurin-2	A0A0A0MQ92
Tenm3	0.0000	0.21	0.28	-0.02	0.97	-0.39	0.47	0.19	0.26	Odz3 protein	B7ZNJ5
Tenm4	-0.6852	0.65	0.0280	-0.45	0.0310	0.03	0.75	0.20	0.62	Teneurin-4	Q3UHK6
Tf	-0.1183	-0.52	0.0001	0.10	0.27	-0.68	0.0007	-0.42	0.0010	Serotransferrin	Q921I1
Tfam	-2.4956	1.07	0.0010	-0.74	0.089	0.22	0.23	0.33	0.0016	Transcription factor A, mitochondrial	P40630
Tfg	-0.0896	0.53	0.13	-0.29	0.22	0.39	0.47	0.24	0.5	Trk-fused gene (Fragment)	B8JJG8
Tfrc	-0.0016	-1.20	0.51	0.08	0.88	-4.49	0.21	-1.11	0.69	Transferrin receptor protein 1	Q62351
Them4	2.2694	-0.86	0.13	-3.87	0.17	-1.67	0.16	-4.73	0.0240	Acyl-coenzyme A thioesterase THEM4	Q3UUI3
Them6	-0.0145	1.02	0.073	-0.11	0.77	0.76	0.27	0.91	0.07	Protein THEM6	Q80ZW2
Thop1	-0.0845	-0.63	0.0023	0.19	0.54	-0.13	0.59	-0.44	0.06	Thimet oligopeptidase	A0A0R4IZY0
Thrap3	-0.0646	0.39	0.27	-0.43	0.21	-0.09	0.69	-0.04	0.83	Thyroid hormone receptor-associated protein 3	Q569Z6

Table S2: Differentially expressed proteins in cerebral cortex *(continued)*

gene	$\pi_{TW/EW} \times \pi_{TF/TW}$	log2FC TW/EW	padj TW/EW	log2FC TF/TW	padj TF/TW	log2FC EF/EW	padj EF/EW	log2FC TF/EW	padj TF/EW	description	accession
Thsd7a	0.0002	0.01	0.79	0.21	0.2	0.31	0.0030	0.23	0.15	Thrombospondin type-1 domain-containing protein 7A	E9PWD2
Thy1	-0.0442	0.34	0.18	-0.29	0.25	-0.05	0.73	0.06	0.38	Thy-1 membrane glycoprotein	P01831
Tial1	-2.0240	3.64	0.62	-4.71	0.27	3.94	0.52	-1.07	0.78	Nucleolysin TIAR	P70318
Timm10	-0.0407	-0.27	0.35	0.43	0.17	0.12	0.98	0.16	0.42	Mitochondrial import inner membrane translocase subunit Tim10	P62073
Timm44	-0.0509	0.94	0.32	-0.24	0.35	-0.21	0.89	0.70	0.65	Mitochondrial import inner membrane translocase subunit TIM44	O35857
Timm8a1	0.0390	-0.29	0.85	-2.81	0.21	-0.84	0.35	-3.10	0.19	Mitochondrial import inner membrane translocase subunit Tim8 A	Q9WVA2
Timm8b	-0.0009	0.03	0.72	-0.31	0.22	0.50	0.17	-0.28	0.34	Mitochondrial import inner membrane translocase subunit Tim8 B	P62077
Timm9	-0.0017	-0.04	0.81	0.44	0.083	0.26	0.59	0.39	0.0440	Mitochondrial import inner membrane translocase subunit Tim9	Q9WV98
Tjp1	-9.3886	1.89	0.059	-2.18	0.0140	0.24	0.53	-0.29	0.27	Tight junction protein ZO-1	B9EHJ3
Tjp2	-19.4769	1.69	0.18	-7.38	0.0080	0.63	0.43	-5.69	0.085	Tight junction protein ZO-2	A0A3B2WBH9
Tkfc	-0.0133	1.05	0.67	-0.90	0.83	0.23	0.66	0.15	0.62	Triokinase/FMN cyclase	Q8VC30
Tkt	0.0262	-0.35	0.0001	-0.07	0.54	-0.52	0.0003	-0.42	0.0001	Transketolase	P40142
Tln1	-0.0420	0.32	0.2	-0.34	0.28	0.15	0.96	-0.02	0.62	Talin-1	P26039
Tln2	0.0055	0.18	0.092	0.17	0.67	0.11	0.55	0.36	0.22	Talin-2	A0A1L1SQ51
Tmcc1	-0.2984	0.86	0.24	-0.93	0.25	0.70	0.19	-0.07	0.93	Transmembrane and coiled-coil domains protein 1 (Fragment)	F8WJ98
Tmed10	-0.0021	0.27	0.34	-0.09	0.65	0.21	0.28	0.18	0.65	Transmembrane emp24 domain-containing protein 10	Q9D1D4
Tmem11	-1.6412	2.01	0.0130	-0.74	0.26	1.99	0.08	1.27	0.23	Transmembrane protein 11, mitochondrial	Q8BK08
Tmem121b	-0.0085	0.87	0.71	-0.72	0.81	1.78	0.24	0.15	0.64	Transmembrane protein 121B	Q99MX7
Tmem126a	0.0002	-0.37	0.38	-0.03	0.91	0.20	1	-0.40	0.56	Transmembrane protein 126A	Q9D8Y1
Tmem132a	0.0000	-0.01	1	-0.03	0.65	-0.03	0.92	-0.04	0.83	Transmembrane protein 132A	D3Z4S6
Tmem132b	-0.0001	0.75	0.49	-0.03	0.96	1.46	0.13	0.73	0.5	Transmembrane protein 132B	F7BAB2
Tmem214	0.4878	1.49	0.3	0.76	0.15	-0.08	0.86	2.25	0.0050	Transmembrane protein 214	D3Z6S1
Tmem263	-0.2053	0.58	0.2	-0.68	0.18	-0.39	0.76	-0.10	0.77	Transmembrane protein 263	Q9DAM7
Tmem65	-0.0001	0.19	0.99	-0.43	0.6	-0.53	0.23	-0.24	0.4	Transmembrane protein 65	Q4VAE3
Tmem94	-0.0029	0.39	0.0180	-0.05	0.82	0.10	0.44	0.34	0.0230	Transmembrane protein 94	A0A668KL57
Tmod1	-1.5022	0.81	0.059	-0.80	0.0130	1.62	0.062	0.01	0.75	Tropomodulin-1	P49813
Tmod2	-0.0113	0.20	0.52	-0.33	0.25	1.20	0.0010	-0.14	0.62	Tropomodulin-2	Q9JKK7
Tmod3	-0.1002	1.67	0.41	-0.40	0.41	2.41	0.23	1.26	0.46	Tropomodulin-3	Q9JHJ0
Tmpo	-0.0019	-0.10	0.7	0.27	0.35	0.25	0.34	0.18	0.41	Lamina-associated polypeptide 2, isoforms beta/delta/epsilon/gamma	Q61029
Tmx2	0.0000	0.01	0.95	-0.36	0.75	-0.60	0.29	-0.36	0.66	Thioredoxin domain-containing protein 14	D3Z2J6

Table S2: Differentially expressed proteins in cerebral cortex *(continued)*

gene	$\pi_{TW/EW} \times \pi_{TF/TW}$	log2FC TW/EW	padj TW/EW	log2FC TF/TW	padj TF/TW	log2FC EF/EW	padj EF/EW	log2FC TF/EW	padj TF/EW	description	accession
Tnc	-0.4936	-0.67	0.0001	0.20	0.12	-0.03	0.55	-0.47	0.0001	Tenascin	Q80YX1
Tnik	-2.6682	0.90	0.0420	-1.04	0.0085	-0.42	0.54	-0.14	0.92	Tnik protein	B2RQ80
Tnks1bp1	-0.0030	0.16	0.52	-0.20	0.47	0.29	0.68	-0.04	0.96	182 kDa tankyrase-1-binding protein	P58871
Tnpo1	-0.2738	-0.40	0.061	0.37	0.0300	0.34	0.19	-0.03	0.91	Transportin-1	Q8BFY9
Tnr	-0.0067	-0.22	0.68	0.39	0.34	0.06	0.77	0.17	0.0480	Tenascin-R	Q8BYI9
Tom1l2	0.0000	-0.32	0.0010	0.00	0.42	0.09	0.97	-0.31	0.0040	TOM1-like protein 2	Q5SRX1
Tomm20	-0.2784	0.66	0.19	-0.61	0.11	0.44	0.07	0.05	0.62	Mitochondrial import receptor subunit TOM20 homolog	A0A1D5RLZ6
Tomm34	0.0000	0.01	0.97	-0.11	0.87	0.43	0.0390	-0.10	0.84	Mitochondrial import receptor subunit TOM34	Q9CYG7
Tomm40	-0.0323	0.44	0.13	-0.15	0.28	0.39	0.29	0.29	0.67	Mitochondrial import receptor subunit TOM40 homolog	Q9QYA2
Tomm70	0.0018	0.44	0.074	0.09	0.91	0.46	0.12	0.54	0.0350	Mitochondrial import receptor subunit TOM70	Q9CZW5
Top1	0.0085	-0.36	0.56	-0.92	0.79	0.52	0.55	-1.27	0.29	DNA topoisomerase 1	Q04750
Top2a	-0.0977	-0.65	0.061	0.25	0.32	0.11	0.79	-0.40	0.085	DNA topoisomerase 2-alpha	Q01320
Top2b	-1.4597	3.03	0.88	-7.95	0.081	-3.44	0.57	-4.92	0.34	DNA topoisomerase 2-beta	Q64511
Tpd52l2	-2.1478	1.03	0.091	-1.29	0.0280	0.04	0.68	-0.26	0.48	Tumor protein D54	A2AUD5
Tpgs1	-0.1827	0.57	0.38	-1.38	0.28	-2.26	0.23	-0.81	0.62	Tubulin polyglutamylase complex subunit 1	A0A6Q6PKE1
Tpi1	-0.0195	-0.43	0.075	0.11	0.43	-0.36	0.0140	-0.32	0.05	Triosephosphate isomerase	P17751
Tpm3	-0.2908	0.43	0.082	-0.38	0.0230	0.82	0.0160	0.06	0.77	Tropomyosin alpha-3 chain	D3Z6I8
Tpm4	0.0049	0.84	0.21	0.17	0.89	0.58	0.6	1.01	0.2	Tropomyosin alpha-4 chain	Q6IRU2
Tpp2	-0.0865	-0.13	0.59	0.84	0.0003	0.18	0.085	0.72	0.0001	Tripeptidyl-peptidase 2	Q64514
Tppp	-0.0002	0.02	0.52	-0.07	0.3	0.13	0.72	-0.06	0.77	Tubulin polymerization-promoting protein	Q7TQD2
Tppp3	0.1341	-0.22	0.57	-0.99	0.0030	-0.55	0.2	-1.20	0.0010	Tubulin polymerization-promoting protein family member 3	Q9CRB6
Tpr	0.0000	-0.05	0.61	0.18	0.99	-0.06	0.83	0.14	0.75	Nucleoprotein TPR	F6ZDS4
Tpt1	0.0022	-0.17	0.47	-0.13	0.5	-0.29	0.0320	-0.31	0.059	Translationally-controlled tumor protein	D3YU75
Tra2a	-0.0012	0.39	0.84	-0.44	0.81	-0.72	0.2	-0.05	0.96	Transformer-2 protein homolog alpha	A0A0N4SVC2
Traf3	-66.0288	5.35	0.0250	-5.20	0.0330	0.59	0.42	0.15	0.47	TNF receptor-associated factor	Q3UHJ1
Trap1	-0.3962	0.35	0.35	-0.92	0.0020	-0.64	0.14	-0.58	0.17	Heat shock protein 75 kDa, mitochondrial	Q9CQN1
Trappc1	-0.0007	-0.06	0.85	0.33	0.34	0.20	0.26	0.26	0.27	Trafficking protein particle complex subunit	B1ASW5
Trappc10	-2.1037	2.47	0.35	-2.59	0.19	-2.16	0.57	-0.12	0.86	Trafficking protein particle complex subunit 10	Q3TLI0
Trappc11	0.0015	0.13	0.82	0.59	0.6	0.24	0.74	0.73	0.21	Trafficking protein particle complex subunit 11	B2RXC1

Table S2: Differentially expressed proteins in cerebral cortex *(continued)*

gene	$\pi_{TW/EW} \times \pi_{TF/TW}$	log2FC TW/EW	padj TW/EW	log2FC TF/TW	padj TF/TW	log2FC EF/EW	padj EF/EW	log2FC TF/EW	padj TF/EW	description	accession
Trappc14	-0.1280	0.69	0.12	-0.43	0.34	0.15	0.37	0.27	0.2	Trafficking protein particle complex subunit 14	A0A0G2JDC3
Trappc3	0.1304	0.24	0.25	0.47	0.0120	0.40	0.0300	0.72	0.0040	Trafficking protein particle complex subunit 3	O55013
Trappc4	-0.0430	-0.21	0.51	0.66	0.087	0.12	0.57	0.45	0.0280	Trafficking protein particle complex subunit 4	Q9ES56
Trappc5	-0.0381	0.45	0.58	-0.48	0.18	0.50	0.5	-0.03	0.51	Trafficking protein particle complex subunit 5	Q9CQA1
Trappc6b	-0.0244	-0.29	0.23	0.68	0.64	0.38	0.12	0.39	0.96	Trafficking protein particle complex subunit 6B	Q9D289
Trappc9	-0.0314	0.92	0.3	-0.39	0.68	1.08	0.18	0.53	0.66	Trafficking protein particle complex subunit 9	Q3U0M1
Trim2	0.0002	-0.01	0.46	-0.10	0.34	-0.12	0.12	-0.12	0.058	Tripartite motif-containing protein 2	E9QKC6
Trim28	-0.4096	0.34	0.22	-1.05	0.0180	-0.64	0.0080	-0.71	0.0260	Transcription intermediary factor 1-beta	Q62318
Trim3	-11.5676	1.03	0.0007	-1.15	0.0008	-0.29	0.6	-0.12	0.71	Tripartite motif-containing protein 3	Q3TDT0
Trim46	-15.9138	1.80	0.0110	-2.35	0.0120	-0.02	0.91	-0.55	0.73	Tripartite motif-containing protein 46	D3YXA6
Trim9	-0.1471	0.87	0.31	-0.77	0.37	0.06	0.71	0.10	0.87	E3 ubiquitin-protein ligase TRIM9	A0A6I8MWZ7
Trp53bp1	0.0015	0.09	0.82	0.41	0.34	0.28	0.79	0.50	0.28	Transformation-related protein 53-binding protein 1	A0A5H1ZRL7
Trp53i11	0.0009	0.41	0.27	0.08	0.89	-0.15	0.68	0.48	0.19	Tumor protein p53-inducible protein 11 (Fragment)	A2AGS6
Trpv2	-0.0573	-0.33	0.25	0.40	0.19	0.35	0.058	0.06	0.83	Transient receptor potential cation channel subfamily V member 2	Q9WTR1
Tsc2	0.0000	0.01	0.96	0.08	0.8	0.43	0.42	0.09	0.81	Tuberin	A0A2I3BPP1
Tsc22d4	-0.0972	0.35	0.6	-1.36	0.12	0.49	0.62	-1.01	0.46	TSC22 domain family protein 4	D3YZZ4
Tsn	-0.0939	-0.36	0.13	0.37	0.16	0.41	0.0290	0.01	0.94	Translin	Q62348
Tst	0.0000	-0.07	0.81	0.01	0.88	-0.25	0.23	-0.06	0.83	Thiosulfate sulfurtransferase	P52196
Ttc37	0.0000	-0.10	0.93	0.30	1	-0.31	0.37	0.20	0.58	Tetratricopeptide repeat domain 37	F8VPK0
Ttc7b	-0.0140	0.24	0.11	-0.09	0.21	0.19	0.41	0.16	0.75	Tetratricopeptide repeat protein 7B	A0A1Y7VL44
Ttc9b	-0.0068	-0.20	0.53	0.40	0.49	-0.11	0.62	0.20	0.9	Tetratricopeptide repeat protein 9B	Q9D6E4
Ttyh1	0.0302	-0.31	0.5	-0.77	0.38	-0.47	0.5	-1.07	0.23	Protein tweety homolog	A0A0U1RPU8
Ttyh3	-0.0188	-0.22	0.081	0.13	0.25	0.02	1	-0.08	0.54	Protein tweety homolog 3	Q6P5F7
Tuba1b	-4.9471	0.77	0.0020	-0.77	0.0008	0.04	0.45	0.00	0.97	Tubulin alpha-1B chain	P05213
Tubb4b	-0.5788	0.47	0.0340	-0.54	0.0280	-0.23	0.45	-0.07	0.88	Tubulin beta-4B chain	P68372
Tubg2	0.0069	0.30	0.29	0.11	0.41	0.21	0.43	0.42	0.073	Tubulin gamma-2 chain	Q8VCK3
Tubgcp2	0.0044	-0.40	0.7	-0.93	0.84	-1.25	0.58	-1.33	0.52	Gamma-tubulin complex component 2	Q921G8
Tubgcp3	-1.1919	7.07	0.27	-3.44	0.82	0.40	0.66	3.64	0.39	Gamma-tubulin complex component 3	P58854
Tufm	-2.3371	0.73	0.0010	-0.62	0.0190	0.00	0.72	0.11	0.69	Elongation factor Tu, mitochondrial	Q8BFR5
Twf1	0.0000	-0.26	1	-0.39	0.084	-0.11	0.78	-0.65	0.5	Twinfilin-1	Q91YR1

Table S2: Differentially expressed proteins in cerebral cortex *(continued)*

gene	$\pi_{TW/EW} \times \pi_{TF/TW}$	log2FC TW/EW	padj TW/EW	log2FC TF/TW	padj TF/TW	log2FC EF/EW	padj EF/EW	log2FC TF/EW	padj TF/EW	description	accession
Twf2	-3.1203	0.44	0.0130	-0.94	0.0001	0.25	0.22	-0.50	0.062	Twinfilin-2	Q9Z0P5
Txndc12	0.0000	0.08	0.36	-0.02	0.91	-0.48	0.093	0.06	0.5	Thioredoxin domain-containing protein 12	Q9CQU0
Txnl1	-0.4378	-0.39	0.0190	0.42	0.0280	-0.05	0.58	0.03	0.72	Thioredoxin-like protein 1	A0A494B955
Txnrd1	-0.0043	-0.54	0.0230	0.05	0.8	-0.75	0.05	-0.49	0.089	Thioredoxin reductase 1, cytoplasmic	Q9JMH6
Txnrd2	0.0000	0.00	0.99	0.16	0.34	0.26	0.5	0.16	0.25	Thioredoxin-disulfide reductase	J3QMN4
Uba1	-0.0001	-0.22	0.58	0.05	0.89	0.14	0.92	-0.17	0.31	Ubiquitin-like modifier-activating enzyme 1	Q02053
Uba2	-0.4315	-0.55	0.11	0.59	0.0410	-0.12	0.83	0.03	0.93	SUMO-activating enzyme subunit 2	Q9Z1F9
Uba5	0.0013	-0.44	0.075	-0.03	0.82	-0.16	0.73	-0.47	0.054	Ubiquitin-like modifier-activating enzyme 5	Q8VE47
Ubap2l	0.0004	0.07	0.96	0.68	0.33	0.41	0.33	0.75	0.29	Ubiquitin-associated protein 2-like	Q80X50
Ube2i	-0.0019	0.17	0.61	-0.22	0.58	0.40	0.087	-0.04	0.92	SUMO-conjugating enzyme UBC9 (Fragment)	G3UYP0
Ube2m	0.0030	-0.08	0.77	-0.35	0.11	0.08	0.33	-0.44	0.098	NEDD8-conjugating enzyme Ubc12	P61082
Ube2n	0.0002	-0.12	0.81	-0.25	0.83	-0.37	0.31	-0.37	0.68	Ubiquitin-conjugating enzyme E2 N	P61089
Ube2o	-0.0013	0.12	0.64	-0.16	0.44	0.01	0.77	-0.04	0.63	E2 ubiquitin-conjugating enzyme UBE2O	UBE2O
Ube2v2	0.0919	-0.28	0.059	-0.23	0.069	-0.23	0.1	-0.51	0.0003	Ubiquitin-conjugating enzyme E2 variant 2	A6X925
Ube3a	-20.5864	3.11	0.32	-8.07	0.0220	0.50	0.88	-4.96	0.098	Ubiquitin-protein ligase E3A	O08759
Ubl4a	0.0000	-0.07	0.96	0.16	0.6	0.49	0.0220	0.09	0.53	Ubiquitin-like protein 4A	P21126
Ubr4	-0.0001	0.08	0.78	-0.05	0.51	0.25	0.97	0.03	0.76	E3 ubiquitin-protein ligase UBR4	A2AN08
Ubxn6	0.0000	0.12	0.58	-0.01	1	0.18	0.59	0.11	0.61	UBX domain-containing protein 6	Q99PL6
Uchl1	-0.0152	-0.63	0.0001	0.03	0.63	-0.63	0.0006	-0.59	0.0020	Ubiquitin carboxyl-terminal hydrolase isozyme L1	Q9R0P9
Uchl5	0.0071	-0.09	0.76	-1.10	0.25	-1.95	0.0190	-1.19	0.0330	Ubiquitin carboxyl-terminal hydrolase isozyme L5	Q9WUP7
Uck1	-0.0006	0.12	0.9	-0.35	0.48	0.55	0.16	-0.24	0.69	Uridine-cytidine kinase	A2AN37
Ufc1	-0.0002	-0.22	0.76	0.08	0.83	-0.30	0.34	-0.13	0.94	Ubiquitin-fold modifier-conjugating enzyme 1	M0QWS4
Ufd1	-0.5191	0.60	0.41	-1.74	0.052	0.00	0.91	-1.14	0.41	Ubiquitin recognition factor in ER-associated degradation protein 1	P70362
Ufl1	-0.0418	3.44	0.53	-3.33	0.97	3.73	0.4	0.11	0.65	E3 UFM1-protein ligase 1	Q8CCJ3
Uggt1	-0.0149	-0.17	0.5	0.39	0.18	0.07	0.78	0.22	0.32	UDP-glucose:glycoprotein glucosyltransferase 1	Q6P5E4
Ugp2	0.0000	-0.14	0.95	0.27	0.97	0.03	0.49	0.13	0.57	UTP–glucose-1-phosphate uridylyltransferase	Q91ZJ5
Uhrf1bp1l	0.0237	0.33	0.73	0.47	0.076	-0.97	0.55	0.81	0.092	UHRF1-binding protein 1-like	A2RSJ4
Unc13a	-0.9333	1.15	0.35	-1.78	0.1	0.66	0.25	-0.63	0.5	Protein unc-13 homolog A	H3BJZ7
Unc5a	-1.3892	0.89	0.36	-4.12	0.14	-0.19	0.85	-3.23	0.36	Netrin receptor UNC5A	Q8K1S4

Table S2: Differentially expressed proteins in cerebral cortex *(continued)*

gene	$\pi_{\mathrm{TW/EW}}\times$ $\pi_{\mathrm{TF/TW}}$	log2FC TW/EW	padj TW/EW	log2FC TF/TW	padj TF/TW	log2FC EF/EW	padj EF/EW	log2FC TF/EW	padj TF/EW	description	accession
Unc5c	0.0001	-0.27	0.53	-0.03	0.92	-0.79	0.65	-0.31	0.45	Netrin receptor UNC5C	O08747
Upf1	-0.0517	0.56	0.06	-0.33	0.59	-0.04	0.87	0.24	0.43	Regulator of nonsense transcripts 1	Q9EPU0
Uqcc2	0.0002	0.02	0.94	0.36	0.15	0.43	0.13	0.38	0.1	Mitochondrial nucleoid factor 1	D3Z4C9
Uqcrb	-0.0129	0.10	0.33	-0.26	0.094	0.27	0.79	-0.16	0.41	Cytochrome b-c1 complex subunit 7	Q9CQB4
Uqcrc1	-0.9546	0.25	0.0110	-0.56	0.0003	0.21	0.86	-0.31	0.27	Cytochrome b-c1 complex subunit 1, mitochondrial	Q9CZ13
Uqcrc2	-3.9632	0.55	0.0010	-0.67	0.0003	0.28	0.058	-0.13	0.078	Cytochrome b-c1 complex subunit 2, mitochondrial	Q9DB77
Uqcrfs1	-0.1324	0.48	0.22	-0.60	0.2	0.25	0.97	-0.12	0.66	Cytochrome b-c1 complex subunit Rieske, mitochondrial	Q9CR68
Uqcrq	-0.4010	0.76	0.5	-1.59	0.079	-0.09	0.65	-0.83	0.096	Cytochrome b-c1 complex subunit 8	Q9CQ69
Uso1	0.0020	0.03	0.26	0.29	0.4	-0.11	0.91	0.33	0.0030	General vesicular transport factor p115	Q9Z1Z0
Usp10	0.0007	0.66	0.32	0.17	0.97	0.04	0.86	0.82	0.28	Ubiquitin carboxyl-terminal hydrolase 10	P52479
Usp14	-0.0030	-0.45	0.0070	0.14	0.95	-0.31	0.19	-0.32	0.19	Ubiquitin carboxyl-terminal hydrolase	E9PYI8
Usp15	-0.0488	-0.21	0.44	0.60	0.082	0.43	0.15	0.40	0.19	Ubiquitin carboxyl-terminal hydrolase 15	Q8R5H1
Usp5	0.0039	-0.15	0.48	-0.07	0.069	0.00	0.68	-0.22	0.3	Ubiquitin carboxyl-terminal hydrolase 5	P56399
Usp7	-0.0103	0.40	0.38	-0.23	0.54	0.19	0.8	0.17	0.58	Ubiquitin carboxyl-terminal hydrolase 7	E9PXY8
Usp8	-0.0100	1.44	0.21	-0.10	0.79	0.79	0.52	1.35	0.24	Ubiquitin carboxyl-terminal hydrolase 8	Q80U87
Usp9x	0.0001	0.49	0.54	0.04	0.95	0.58	0.59	0.53	0.58	Probable ubiquitin carboxyl-terminal hydrolase FAF-X	P70398
Uvrag	-0.0527	0.36	0.0420	-0.29	0.43	-0.32	0.44	0.07	0.91	UV radiation resistance-associated protein	Q8K245
Vamp2	-0.0073	-0.18	0.18	0.11	0.32	-0.13	0.25	-0.07	0.6	Synaptobrevin-2	B0QZN5
Vamp7	-0.6216	3.76	0.53	-3.58	0.68	0.36	0.78	0.18	0.8	Vesicle-associated membrane protein 7	P70280
Vapb	-0.0334	0.44	0.0140	-0.07	0.26	0.16	0.7	0.37	0.43	Vesicle-associated membrane protein, associated protein B and C	Q8BH80
Vars1	0.0000	0.04	0.95	0.03	0.52	0.08	0.71	0.07	0.4	Valine–tRNA ligase	Q9Z1Q9
Vasp	0.0036	0.20	0.42	0.10	0.33	0.98	0.0170	0.30	0.33	Vasodilator-stimulated phosphoprotein	P70460
Vat1	-0.0030	0.06	0.52	-0.23	0.17	-0.51	0.25	-0.17	0.52	Synaptic vesicle membrane protein VAT-1 homolog	Q62465
Vat1l	0.0003	-0.01	0.76	-0.47	0.28	0.34	0.17	-0.48	0.32	Synaptic vesicle membrane protein VAT-1 homolog-like	Q80TB8
Vbp1	0.0000	-0.14	1	0.69	0.11	-0.57	0.65	0.55	0.31	Prefoldin subunit 3	P61759
Vcan	-0.0129	0.27	0.57	-0.88	0.6	-0.93	0.61	-0.61	0.83	Versican core protein	E9PYH0
Vcl	-0.1434	1.48	0.22	-0.76	0.64	1.09	0.3	0.72	0.52	Vinculin	Q64727
Vcp	-0.0172	-0.21	0.75	0.49	0.0460	0.26	0.7	0.29	0.085	Transitional endoplasmic reticulum ATPase	Q01853
Vcpip1	-0.0019	0.02	0.62	-0.83	0.28	-0.69	0.075	-0.80	0.075	Ubiquitinyl hydrolase 1	A0A0R4J0M9
Vdac1	0.0002	0.01	0.13	0.18	0.71	0.00	0.31	0.20	0.085	Voltage-dependent anion-selective channel protein 1	Q60932

Table S2: Differentially expressed proteins in cerebral cortex *(continued)*

gene	$\pi_{\mathrm{TW/EW}}\times \pi_{\mathrm{TF/TW}}$	log2FC TW/EW	padj TW/EW	log2FC TF/TW	padj TF/TW	log2FC EF/EW	padj EF/EW	log2FC TF/EW	padj TF/EW	description	accession
Vdac2	0.0005	0.11	0.12	0.15	0.93	-0.01	0.21	0.26	0.17	Outer mitochondrial membrane protein porin 2	G3UX26
Vdac3	0.0011	0.08	0.35	0.21	0.71	0.07	0.58	0.30	0.13	Voltage-dependent anion-selective channel protein 3	J3QMG3
Vgf	-0.0263	0.38	0.28	-0.26	0.33	0.02	0.36	0.12	0.99	Neurosecretory protein VGF	Q0VGU4
Vps11	0.0005	0.06	0.61	0.20	0.66	0.02	0.65	0.26	0.42	Vacuolar protein sorting-associated protein 11 homolog	Q91W86
Vps13a	-0.4651	0.39	0.81	-6.37	0.0090	-0.99	0.7	-5.98	0.0150	Vacuolar protein sorting-associated protein 13A	Q5H8C4
Vps13c	-0.0964	1.16	0.29	-0.72	0.61	0.05	0.89	0.44	0.53	Vacuolar protein sorting-associated protein 13C	Q8BX70
Vps16	0.0290	0.75	0.0010	0.08	0.69	-0.03	0.78	0.83	0.0008	Vacuolar protein sorting-associated protein 16 homolog	G3X8X7
Vps18	0.0084	0.46	0.32	0.13	0.52	0.05	0.68	0.60	0.077	Vacuolar protein sorting-associated protein 18 homolog	Q8R307
Vps26a	-0.2066	2.44	0.17	-0.53	0.62	-4.13	0.052	1.91	0.63	Vacuolar protein sorting-associated protein 26A	P40336
Vps26b	-0.7512	-0.60	0.35	2.18	0.055	0.23	0.65	1.57	0.22	Vacuolar protein sorting-associated protein 26B	Q8C0E2
Vps29	0.5549	0.32	0.18	0.64	0.0002	0.48	0.17	0.96	0.0010	Vacuolar protein sorting-associated protein 29	Q9QZ88
Vps33a	0.0001	0.07	0.99	0.30	0.12	0.01	0.89	0.37	0.0080	Vacuolar protein sorting-associated protein 33A	Q9D2N9
Vps33b	0.1291	0.50	0.059	0.31	0.21	-0.20	0.71	0.81	0.0280	Vacuolar protein sorting-associated protein 33B	P59016
Vps35	0.0000	0.33	0.15	0.43	1	-0.60	0.27	0.76	0.23	Vacuolar protein sorting-associated protein 35	Q9EQH3
Vps39	0.0010	0.20	0.93	0.23	0.19	-0.75	0.55	0.43	0.0240	Vam6/Vps39-like protein	Q8R5L3
Vps41	0.0038	0.34	0.51	0.14	0.53	-0.17	0.87	0.48	0.14	Vacuolar protein sorting-associated protein 41 homolog	Q5KU39
Vps45	-0.0027	-0.02	0.85	0.49	0.0001	-0.17	0.31	0.46	0.0001	Vacuolar protein sorting-associated protein 45	P97390
Vps4b	-0.0050	0.38	0.55	-0.28	0.66	-0.06	0.83	0.10	0.79	Vacuolar protein sorting-associated protein 4B	P46467
Vps50	0.0048	0.13	0.77	0.44	0.18	-0.05	0.8	0.57	0.0280	Syndetin	Q8CI71
Vps51	-0.0002	-0.11	0.78	0.51	0.91	-0.04	0.7	0.40	0.7	Vacuolar protein sorting-associated protein 51 homolog	Q3UVL4
Vps52	-0.4606	-0.98	0.0170	0.81	0.47	-0.41	0.58	-0.18	0.88	Vacuolar protein sorting-associated protein 52 homolog	Q8C754

Table S2: Differentially expressed proteins in cerebral cortex *(continued)*

gene	$\pi_{TW/EW} \times \pi_{TF/TW}$	log2FC TW/EW	padj TW/EW	log2FC TF/TW	padj TF/TW	log2FC EF/EW	padj EF/EW	log2FC TF/EW	padj TF/EW	description	accession
Vps53	-0.0723	-0.10	0.42	0.64	0.0010	0.26	0.056	0.54	0.0009	Vacuolar protein sorting-associated protein 53 homolog	Q8CCB4
Vps8	0.0686	0.87	0.1	0.38	0.62	-0.08	0.87	1.25	0.073	Vacuolar protein sorting-associated protein 8 homolog	F8WI64
Vps9d1	-1.1608	1.03	0.18	-1.40	0.083	0.49	0.47	-0.37	0.86	VPS9 domain-containing protein 1	Q3U280
Vsnl1	-9.4887	4.31	0.14	-4.16	0.24	0.23	0.66	0.15	0.63	Visinin-like protein 1	P62761
Vti1b	0.2627	0.54	0.067	0.45	0.12	0.59	0.46	0.99	0.0010	Vesicle transport through interaction with t-SNAREs homolog 1B	O88384
Vwa5a	-0.0017	0.10	0.77	-0.26	0.26	-0.54	0.88	-0.16	0.34	von Willebrand factor A domain-containing protein 5A	Q99KC8
Vwa8	-1.3202	1.07	0.13	-1.35	0.093	0.60	0.53	-0.27	0.66	von Willebrand factor A domain-containing protein 8	Q8CC88
Wars2	-0.0094	-0.20	0.96	0.66	0.0001	0.06	0.73	0.46	0.0022	Tryptophan–tRNA ligase, mitochondrial	Q9CYK1
Wasf1	-0.0077	0.40	0.23	-0.08	0.42	-0.11	0.84	0.32	0.23	Wiskott-Aldrich syndrome protein family member 1	Q8R5H6
Wasf2	0.0069	0.19	0.34	0.20	0.41	-0.08	0.5	0.39	0.0330	Wiskott-Aldrich syndrome protein family member 2	Q8BH43
Wasf3	0.0243	-0.63	0.26	-0.58	0.77	-0.78	0.17	-1.20	0.12	Wiskott-Aldrich syndrome protein family member 3	Q8VHI6
Washc1	0.2794	0.44	0.58	1.07	0.0031	0.77	0.21	1.50	0.0010	WASH complex subunit 1	Q8VDD8
Washc4	0.0002	0.02	0.85	0.30	0.32	-0.53	0.3	0.33	0.28	WASH complex subunit 4	Q3UMB9
Washc5	-0.0024	-0.02	0.84	1.00	0.0280	0.02	0.91	0.98	0.0280	WASH complex subunit 5	Q8C2E7
Wasl	0.0011	0.10	0.19	0.05	0.48	0.37	0.36	0.15	0.4	Neural Wiskott-Aldrich syndrome protein	Q91YD9
Wbp11	-0.4712	1.86	0.2	-1.17	0.49	0.21	0.97	0.68	0.76	WW domain-binding protein 11	Q923D5
Wdfy3	-2.5263	1.08	0.0390	-1.15	0.0360	0.13	0.62	-0.07	0.96	WD repeat and FYVE domain-containing protein 3	A0A1D5RLV7
Wdr1	-0.0343	0.06	0.082	-0.19	0.0017	0.19	0.31	-0.14	0.99	WD repeat-containing protein 1	O88342
Wdr13	0.0000	0.07	0.85	-0.04	0.77	-0.31	0.43	0.03	0.88	WD repeat-containing protein 13	Q91V09
Wdr20	-0.8779	1.56	0.42	-2.41	0.24	1.71	0.37	-0.85	0.83	WD repeat-containing protein 20	Q3UWE6
Wdr26	-0.1806	1.49	0.67	-1.85	0.42	1.46	0.44	-0.36	1	WD repeat-containing protein 26	A0A494BB75
Wdr37	-0.0029	0.23	0.94	-0.71	0.22	0.00	0.48	-0.47	0.17	WD repeat-containing protein 37	Q8CBE3
Wdr47	-0.0142	1.05	0.67	-0.90	0.82	0.23	0.67	0.15	0.64	WD repeat-containing protein 47	Q8CGF6
Wdr48	-0.0001	0.07	0.45	-0.03	0.85	0.22	0.058	0.05	0.57	WD repeat-containing protein 48	Q8BH57
Wdr7	0.0023	0.03	0.13	0.20	0.37	-0.15	0.88	0.23	0.53	WD repeat-containing protein 7	Q920I9
Wdr81	-0.8401	1.05	0.26	-1.66	0.15	0.61	0.38	-0.61	0.7	WD repeat-containing protein 81 (Fragment)	K4DI77
Wdr91	-0.0019	0.32	0.67	-0.29	0.76	-0.77	0.59	0.03	0.96	WD repeat-containing protein 91	Q7TMQ7
Wfs1	0.0025	-0.33	0.35	-0.12	0.73	0.30	0.0280	-0.46	0.0490	Wolframin	Q3UN10

Table S2: Differentially expressed proteins in cerebral cortex *(continued)*

gene	$\pi_{TW/EW} \times \pi_{TF/TW}$	log2FC TW/EW	padj TW/EW	log2FC TF/TW	padj TF/TW	log2FC EF/EW	padj EF/EW	log2FC TF/EW	padj TF/EW	description	accession
Wipf2	0.0000	-0.23	0.75	0.16	0.98	-0.17	0.55	-0.07	0.79	WAS/WASL-interacting protein family member 2	Q6PEV3
Wipf3	-0.0002	0.18	0.57	-0.06	0.86	-0.27	0.48	0.12	0.86	WAS/WASL-interacting protein family member 3	D3Z5R4
Wnk1	-0.0103	0.61	0.98	-4.21	0.35	-0.51	0.82	-3.60	0.43	Serine/threonine-protein kinase WNK1	P83741
Wnk2	-0.0155	0.11	0.83	-1.55	0.075	-0.34	0.68	-1.43	0.069	Serine/threonine-protein kinase WNK2	Q3UH66
Wwp1	-0.5267	-0.62	0.0130	0.39	0.07	0.19	0.56	-0.23	0.087	NEDD4-like E3 ubiquitin-protein ligase WWP1	Q8BZZ3
Xpnpep3	0.0000	0.14	0.67	0.25	1	0.79	0.078	0.39	0.0110	Xaa-Pro aminopeptidase 3	B7ZMP1
Xpo1	-0.0014	0.17	0.53	-0.15	0.63	0.15	0.45	0.02	1	Exportin-1	Q6P5F9
Xrn2	-0.0783	2.64	0.44	-2.64	0.93	-1.50	0.36	0.00	1	5’-3’ exoribonuclease 2	Q9DBR1
Yars	-0.0011	0.37	0.44	-0.10	0.82	-0.04	0.88	0.27	0.55	Tyrosine–tRNA ligase	A2A7S7
Yars2	-0.2729	0.57	0.0006	-0.20	0.18	0.31	0.11	0.37	0.0300	Tyrosine–tRNA ligase, mitochondrial	Q8BYL4
Yme1l1	-0.0204	0.95	0.25	-0.23	0.7	0.37	0.62	0.72	0.26	ATP-dependent zinc metalloprotease YME1L1	O88967
Ythdf1	0.0154	0.23	0.42	0.33	0.29	0.02	1	0.56	0.084	YTH domain-containing family protein 1	P59326
Ywhab	0.0018	0.12	0.16	0.09	0.61	0.09	0.63	0.21	0.4	14-3-3 protein beta/alpha	Q9CQV8
Ywhae	0.0000	-0.23	1	0.09	0.59	-0.04	0.9	-0.14	0.49	14-3-3 protein epsilon	P62259
Ywhag	-0.0390	-0.17	0.55	0.34	0.0025	-0.09	0.61	0.18	0.0410	14-3-3 protein gamma	P61982
Ywhah	-0.0002	-0.08	0.63	0.04	0.44	-0.09	0.51	-0.04	0.87	14-3-3 protein eta	P68510
Ywhaz	0.0000	-0.13	0.96	0.13	0.83	0.00	0.64	0.00	0.69	14-3-3 protein zeta/delta	P63101
Zc2hc1a	-1.8307	0.76	0.0032	-0.64	0.0310	0.15	0.47	0.12	0.44	Zinc finger C2HC domain-containing protein 1A	Q8BJH1
Zc3h15	-0.1801	0.65	0.0440	-0.32	0.23	-0.17	0.39	0.32	0.0300	Zinc finger CCCH domain-containing protein 15	Q3TIV5
Zdhhc5	0.0010	-0.29	0.6	-0.12	0.75	-1.06	0.12	-0.41	0.38	Palmitoyltransferase ZDHHC5	Q8VDZ4
Zfr	-0.1810	0.58	0.088	-0.41	0.19	-0.20	0.6	0.18	0.49	Zinc finger RNA-binding protein	O88532
Zmat2	0.0002	0.07	0.52	0.08	0.78	0.19	0.24	0.15	0.45	Zinc finger matrin-type protein 2	Q9CPW7
Zfp365	-0.0886	-0.66	0.0070	0.19	0.47	0.16	0.66	-0.47	0.0110	Protein ZNF365	Q8BG89
Zranb2	0.0006	0.01	0.93	0.59	0.0010	-0.41	0.14	0.60	0.0010	Zinc finger Ran-binding domain-containing protein 2	D3Z4U0
Zwint	-0.0045	0.36	0.1	-0.07	0.66	0.34	0.0310	0.29	0.072	ZW10 interactor	Q9CQU5

Table S3: Proteins from dendrogram cut 1

gene	interaction p-value	trisomy p-value	fluoxetine p-value	TW - TF p-value	TW $\log_2$(quantity) mean ±SE	TF $\log_2$(quantity) mean ±SE	EW $\log_2$(quantity) mean ±SE	EF $\log_2$(quantity) mean ±SE	UniProt accession
Abcf1	0.0370	0.6638	0.0442	0.0400	11.36 ±0.33	10.17 ±0.32	10.64 ±0.17	10.66 ±0.18	Q6P542
Acaa2	0.0096	0.0837	0.0016	0.0002	12.36 ±0.03	11.71 ±0.07	12.25 ±0.06	12.16 ±0.15	Q8BWT1
Actg1	0.0343	0.2057	0.7290	0.1099	12.17 ±0.15	11.53 ±0.31	11.84 ±0.48	12.71 ±0.23	P63260
Adap1	0.0375	0.1159	0.0295	0.0183	12.19 ±0.17	11.45 ±0.15	12.09 ±0.11	12.07 ±0.17	E9PY16
Aifm3	0.0182	0.5913	0.0115	0.0135	12.17 ±0.18	11.43 ±0.12	11.75 ±0.08	11.72 ±0.12	Q3TY86
Ank2	0.0235	0.9726	0.3229	0.0743	10.94 ±0.53	9.62 ±0.30	10.01 ±0.35	10.58 ±0.18	S4R2F3
Atp5c1	0.0149	0.1041	0.0058	0.0124	12.01 ±0.39	10.51 ±0.16	10.89 ±0.21	10.77 ±0.10	A2AKU9
Bckdk	0.0150	0.2180	0.4050	0.0213	11.59 ±0.22	10.89 ±0.03	11.30 ±0.17	11.67 ±0.25	A0A0U1RNL7 A0A0U1RPT4 O55028
Cadps	0.0124	0.1774	0.2059	0.0279	6.86 ±2.42	-0.13 ±0.16	-0.28 ±0.28	2.34 ±2.17	A0A286YDH6
Cc2d1a	0.0220	0.5280	0.6402	0.0958	10.37 ±0.33	9.19 ±0.50	9.62 ±0.46	10.43 ±0.10	Q8K1A6
Cdh6	0.0147	0.2203	0.2534	0.0274	6.64 ±2.33	-0.13 ±0.16	-0.28 ±0.28	2.47 ±2.38	P97326
Cntnap1	0.0325	0.2353	0.2666	0.0464	11.04 ±0.20	10.44 ±0.13	10.84 ±0.23	11.06 ±0.07	O54991
Dars2	0.0390	0.3213	0.1704	0.6347	6.68 ±2.26	4.90 ±2.76	-0.28 ±0.28	7.56 ±2.12	Q8BIP0
Dis3l2	0.0242	0.6867	0.5548	0.1067	11.96 ±0.41	11.15 ±0.12	11.41 ±0.09	11.91 ±0.26	Q8CI75
Dnajc8	0.0093	0.0003	0.0139	0.0221	9.36 ±0.21	2.30 ±2.29	-0.28 ±0.28	-0.03 ±0.45	A2ALF3
Eif2s3x	0.0031	0.0149	0.0544	0.0209	10.68 ±0.32	9.37 ±0.28	9.21 ±0.09	9.56 ±0.13	Q9Z0N1
Epb41l3	0.0180	0.3120	0.0809	0.0257	7.83 ±2.23	1.20 ±0.24	5.43 ±1.52	6.61 ±0.88	A0A3B2WBE1
Fech	0.0151	0.8541	0.2584	0.2074	11.51 ±0.20	11.17 ±0.13	10.97 ±0.30	11.79 ±0.15	A0A494BAI5 Q544X6
Flii	0.0444	0.7729	0.3638	0.3428	10.33 ±0.62	9.62 ±0.31	8.94 ±0.82	10.68 ±0.21	Q9JJ28
Golga3	0.0263	0.7855	0.9985	0.1678	11.41 ±0.16	11.07 ±0.14	11.11 ±0.10	11.44 ±0.12	A0A0R4J1H6 E9QP99 P55937
Lrrc73	0.0334	0.1811	0.0097	0.0230	11.91 ±0.39	10.51 ±0.24	11.65 ±0.03	11.48 ±0.22	B2RWC4
Map4k5	0.0372	0.9988	0.3190	0.0270	7.11 ±2.48	-0.13 ±0.16	2.10 ±2.22	4.88 ±2.68	E9Q1T3
Mink1	0.0278	0.1598	0.2484	0.1000	11.49 ±0.53	10.41 ±0.18	10.32 ±0.12	10.70 ±0.12	G3X9G2 Q9JM52
Mlf2	0.0228	0.1289	0.0814	0.0467	11.54 ±0.48	10.18 ±0.25	11.25 ±0.18	11.46 ±0.19	Q99KX1
Mthfd1l	0.0257	0.0147	0.1623	0.0793	11.24 ±0.18	10.11 ±0.50	11.33 ±0.12	11.62 ±0.11	Q3V3R1
Mtpap	0.0062	0.9423	0.7115	0.0391	10.02 ±0.35	8.90 ±0.25	9.00 ±0.38	9.89 ±0.21	Q9D0D3
Myo1b	0.0469	0.0885	0.3921	0.0778	10.24 ±0.13	9.52 ±0.31	10.16 ±0.08	10.47 ±0.31	Q7TQD7
Myo1d	0.0150	0.1431	0.3201	0.0512	11.09 ±0.59	9.52 ±0.26	10.57 ±0.44	11.30 ±0.20	Q5SYD0
Myo5a	0.0420	0.9763	0.2288	0.0614	12.16 ±0.68	10.48 ±0.28	11.06 ±0.51	11.54 ±0.32	Q99104
Ncstn	0.0109	0.6160	0.4305	0.2484	6.81 ±2.38	2.40 ±2.50	-0.28 ±0.28	7.42 ±2.06	P57716
Ndufa4	0.0423	0.6417	0.6545	0.1513	12.10 ±0.28	11.60 ±0.13	11.60 ±0.17	11.93 ±0.12	Q62425
Nebl	0.0107	0.6920	0.0075	0.0067	11.82 ±0.18	10.96 ±0.12	11.34 ±0.14	11.32 ±0.11	Q9DC07

Table S3: Proteins from dendrogram cut 1 *(continued)*

gene	interaction p-value	trisomy p-value	fluoxetine p-value	TW - TF p-value	TW $\log_2$(quantity) mean ±SE	TF $\log_2$(quantity) mean ±SE	EW $\log_2$(quantity) mean ±SE	EF $\log_2$(quantity) mean ±SE	UniProt accession
Nudt16	0.0482	0.0682	0.2180	0.0188	8.69 ±0.16	7.85 ±0.21	8.64 ±0.36	8.86 ±0.19	A0A087WRE5 Q6P3D0
Olfm3	0.0292	0.9479	0.6129	0.0971	10.71 ±0.29	9.89 ±0.30	10.05 ±0.18	10.58 ±0.30	P63056
Plec	0.0330	0.9447	0.2876	0.0865	11.55 ±0.81	9.75 ±0.34	10.28 ±0.51	10.95 ±0.12	E9Q3W4
Ppm1h	0.0365	0.4025	0.9077	0.1361	13.30 ±0.16	13.01 ±0.06	13.11 ±0.18	13.43 ±0.08	Q3UYC0
Prkacb	0.0445	0.0084	0.3131	0.3826	11.53 ±0.09	11.37 ±0.14	11.66 ±0.07	12.10 ±0.20	P68181
Prrc2c	0.0179	0.6073	0.3257	0.2475	10.61 ±0.31	10.05 ±0.32	9.53 ±0.48	10.77 ±0.10	A0A0A0MQ79 S4R2J9
Prrt2	0.0116	0.1438	0.0025	0.2896	12.70 ±0.18	12.91 ±0.05	11.52 ±0.47	13.28 ±0.11	E9PUL5
Prrt3	0.0225	0.4973	0.2796	0.3804	4.38 ±1.70	2.32 ±1.35	-0.23 ±0.28	4.98 ±1.70	A0A0N4SVB5 Q6PE13
Psmd3	0.0065	0.7992	0.0869	0.0094	11.66 ±0.10	10.64 ±0.25	11.06 ±0.23	11.34 ±0.18	P14685
Ralgapa1	0.0341	0.0214	0.0166	0.7457	11.91 ±0.28	12.01 ±0.06	11.97 ±0.14	13.25 ±0.38	A0A2I3BRX9
Sbf2	0.0136	0.0182	0.0022	0.0106	5.02 ±1.76	-1.63 ±0.45	-0.51 ±0.42	-1.48 ±0.63	E9PXF8 E9Q0D4
Sfxn3	0.0207	0.5806	0.0477	0.0322	11.23 ±0.42	9.89 ±0.24	10.34 ±0.26	10.47 ±0.04	Q91V61
Sfxn5	0.0414	0.6251	0.0793	0.0296	12.05 ±0.21	11.38 ±0.11	11.77 ±0.15	11.82 ±0.15	Q925N0
Sik3	0.0050	0.2010	0.1577	0.0457	11.79 ±0.30	10.95 ±0.14	11.43 ±0.03	11.76 ±0.06	E9PU87 F6U6U5 Q6P4S6
Slc12a6	0.0399	0.3428	0.7834	0.2378	6.38 ±2.14	2.28 ±2.27	-0.28 ±0.28	4.95 ±2.56	Q924N4
Slc25a11	0.0227	0.7721	0.1887	0.0299	11.46 ±0.34	10.45 ±0.12	10.73 ±0.25	11.04 ±0.25	Q9CR62
Slc25a22	0.0361	0.9262	0.2826	0.0486	11.90 ±0.24	11.25 ±0.10	11.44 ±0.23	11.67 ±0.14	Q9D6M3
Sptan1	0.0331	0.4210	0.9734	0.1357	11.61 ±0.28	11.00 ±0.22	11.21 ±0.23	11.81 ±0.27	A3KGU9
Sptbn1	0.0372	0.7803	0.9376	0.1645	11.56 ±0.30	10.95 ±0.25	11.01 ±0.28	11.66 ±0.26	Q62261
Sptbn2	0.0358	0.5459	0.4718	0.1095	11.70 ±0.42	10.74 ±0.30	11.17 ±0.28	11.67 ±0.20	Q68FG2
Suclg1	0.0003	0.3738	0.1756	0.0039	12.42 ±0.08	11.83 ±0.10	12.05 ±0.06	12.37 ±0.12	Q9WUM5
Tbca	0.0095	0.0945	0.0144	0.0000	9.10 ±0.44	-0.13 ±0.16	7.14 ±2.26	7.48 ±2.08	P48428
Timm8b	0.0322	0.0502	0.6582	0.2737	11.35 ±0.14	11.05 ±0.20	11.31 ±0.06	11.74 ±0.16	P62077
Tpd52l2	0.0137	0.1220	0.0202	0.0301	13.91 ±0.51	12.45 ±0.09	12.72 ±0.08	12.77 ±0.05	A2AUD5
Tubb2b	0.0181	0.2137	0.0468	0.0187	11.14 ±0.17	10.54 ±0.08	10.65 ±0.15	10.71 ±0.02	Q9CWF2
Ube2m	0.0205	0.0044	0.1197	0.0161	11.31 ±0.06	10.96 ±0.09	11.38 ±0.03	11.46 ±0.12	P61082
Wdfy3	0.0367	0.1259	0.0853	0.0210	13.28 ±0.27	12.15 ±0.25	12.21 ±0.24	12.34 ±0.31	A0A1D5RLV7 G3UYW1 Q6VNB8

Table S4: Proteins from dendrogram cut 2

gene	interaction p-value	trisomy p-value	fluoxetine p-value	TW - TF p-value	TW $\log_2$(quantity) mean ±SE	TF $\log_2$(quantity) mean ±SE	EW $\log_2$(quantity) mean ±SE	EF $\log_2$(quantity) mean ±SE	UniProt accession
Akr1b3	0.0093	0.4390	0.2794	0.2834	12.28 ±0.18	12.55 ±0.14	12.81 ±0.08	12.23 ±0.13	P45376
Ap2a1	0.0060	0.1708	0.0001	0.0010	10.92 ±0.08	11.55 ±0.07	11.04 ±0.08	11.23 ±0.03	P17426
Ap2a2	0.0478	0.0100	0.0001	0.0013	11.03 ±0.14	11.89 ±0.07	10.93 ±0.11	11.30 ±0.12	P17427
Ap2b1	0.0440	0.5340	0.0045	0.0114	10.97 ±0.24	11.87 ±0.05	11.22 ±0.16	11.42 ±0.10	H3BKM0 Q9DBG3
Armc1	0.0468	0.6161	0.5349	0.1749	10.61 ±0.34	11.16 ±0.11	11.21 ±0.23	10.21 ±0.55	Q9D7A8
Clta	0.0092	0.0146	0.0113	0.0002	12.08 ±0.11	13.24 ±0.09	12.13 ±0.22	12.11 ±0.28	B1AWD8 B1AWD9 O08585
Cltb	0.0027	0.0059	0.0005	0.0003	12.21 ±0.16	13.49 ±0.06	12.28 ±0.24	12.43 ±0.07	Q6IRU5
Cltc	0.0061	0.0028	0.0055	0.0004	11.26 ±0.16	12.52 ±0.08	11.17 ±0.27	11.19 ±0.19	Q68FD5
Commd2	0.0347	0.0370	0.0511	0.0206	12.53 ±0.16	13.41 ±0.23	12.54 ±0.16	12.50 ±0.21	Q8BXC6
Commd9	0.0430	0.0082	0.0023	0.0082	11.27 ±0.18	12.29 ±0.19	11.12 ±0.18	11.39 ±0.10	Q8K2Q0
Dmxl2	0.0102	0.2529	0.0064	0.0088	11.17 ±0.10	11.94 ±0.18	11.39 ±0.13	11.42 ±0.01	B0V2P5 Q8BPN8
Echs1	0.0296	0.0198	0.0833	0.5588	12.69 ±0.18	12.84 ±0.16	12.64 ±0.10	11.52 ±0.44	Q8BH95
Eif3e	0.0426	0.5831	0.1786	0.0286	11.20 ±0.12	11.85 ±0.19	11.50 ±0.11	11.35 ±0.24	P60229
Eif3h	0.0442	0.3793	0.6343	0.1114	4.94 ±0.33	6.81 ±0.95	7.10 ±0.89	5.90 ±0.27	Q91WK2
Eif3i	0.0493	0.3484	0.2591	0.0014	11.87 ±0.05	12.41 ±0.08	12.06 ±0.07	11.90 ±0.30	Q9QZD9
Eif3l	0.0038	0.1941	0.1644	0.0052	11.32 ±0.16	12.04 ±0.06	11.63 ±0.15	11.33 ±0.18	Q8QZY1
Eno1	0.0272	0.2576	0.0966	0.6289	12.01 ±0.11	12.08 ±0.09	12.37 ±0.06	11.95 ±0.13	P17182
Exoc1	0.0147	0.0004	0.3166	0.0561	11.74 ±0.14	12.16 ±0.12	11.52 ±0.09	11.32 ±0.09	Q5PPR2
Exoc3	0.0407	0.3249	0.0011	0.0003	11.01 ±0.05	11.59 ±0.06	11.13 ±0.13	11.30 ±0.09	Q6KAR6
Exoc8	0.0252	0.0003	0.4531	0.0593	11.17 ±0.13	11.52 ±0.08	10.91 ±0.12	10.72 ±0.08	Q6PGF7
Gmfb	0.0356	0.7267	0.6840	0.1381	12.72 ±0.08	12.93 ±0.09	12.87 ±0.06	12.72 ±0.06	Q9CQI3
Gsta4	0.0086	0.6631	0.1377	0.1278	10.72 ±0.10	11.01 ±0.13	11.39 ±0.06	10.51 ±0.33	P24472
Gstp1	0.0036	0.5248	0.8687	0.0946	13.17 ±0.10	13.52 ±0.14	13.47 ±0.10	13.08 ±0.02	P19157
Hba	0.0063	0.6518	0.5808	0.1047	11.47 ±0.20	11.87 ±0.05	12.02 ±0.03	11.45 ±0.20	P01942 Q91VB8
Hip1	0.0453	0.5261	0.0017	0.0072	11.18 ±0.18	12.03 ±0.11	11.39 ±0.14	11.64 ±0.09	Q8VD75
Mapk1	0.0250	0.0641	0.4963	0.1033	12.04 ±0.07	12.45 ±0.20	12.10 ±0.05	11.87 ±0.12	P63085
Naxd	0.0232	0.0048	0.1028	0.0184	12.22 ±0.17	13.01 ±0.18	12.07 ±0.10	11.92 ±0.25	J3QMM7
Ocrl	0.0043	0.0204	0.0011	0.0007	11.06 ±0.11	12.06 ±0.11	11.17 ±0.13	11.27 ±0.16	Q6NVF0
Park7	0.0160	0.2062	0.1912	0.3566	12.09 ±0.12	12.24 ±0.10	12.53 ±0.06	12.08 ±0.14	Q99LX0
Pgm1	0.0482	0.0918	0.1586	0.5444	12.10 ±0.13	12.20 ±0.08	12.69 ±0.10	12.14 ±0.23	Q9D0F9
Ppp5c	0.0279	0.1846	0.9665	0.1884	10.88 ±0.18	11.34 ±0.25	11.62 ±0.12	11.14 ±0.17	F7BX26 Q60676
Prdx6	0.0079	0.6613	0.0205	0.7097	12.08 ±0.11	12.18 ±0.23	12.61 ±0.10	11.47 ±0.27	D3Z0Y2 O08709 Q6GT24
Prps1l3	0.0183	0.0127	0.3944	0.0909	12.02 ±0.24	12.78 ±0.29	11.94 ±0.21	10.45 ±0.70	G3UXL2

Table S4: Proteins from dendrogram cut 2 *(continued)*

gene	interaction p-value	trisomy p-value	fluoxetine p-value	TW - TF p-value	TW $\log_2$(quantity) mean ±SE	TF $\log_2$(quantity) mean ±SE	EW $\log_2$(quantity) mean ±SE	EF $\log_2$(quantity) mean ±SE	UniProt accession
Prpsap2	0.0203	0.5645	0.4195	0.0812	11.59 ±0.18	12.21 ±0.23	11.96 ±0.15	11.64 ±0.12	Q8R574
Ptms	0.0441	0.1141	0.6272	0.0347	10.88 ±0.13	11.29 ±0.07	11.47 ±0.09	11.21 ±0.24	Q9D0J8
Pycrl	0.0186	0.0027	0.0504	0.0021	11.61 ±0.14	12.35 ±0.03	11.45 ±0.21	11.37 ±0.16	Q9DCC4
Rogdi	0.0269	0.6330	0.0098	0.0111	10.82 ±0.05	11.63 ±0.22	11.11 ±0.15	11.19 ±0.11	Q3TDK6
Rps21	0.0493	0.6809	0.1145	0.0015	13.55 ±0.09	14.55 ±0.16	14.01 ±0.03	13.88 ±0.48	Q9CQR2
Rps28	0.0040	0.8229	0.1137	0.0061	11.98 ±0.14	13.12 ±0.24	12.70 ±0.11	12.30 ±0.32	P62858
Rpsa	0.0411	0.2868	0.2716	0.0003	11.27 ±0.04	12.29 ±0.13	11.62 ±0.04	11.28 ±0.58	P14206
Sacm1l	0.0233	0.0038	0.7015	0.1415	11.68 ±0.08	11.94 ±0.13	11.56 ±0.15	11.21 ±0.09	A0A5F8MPK9 Q9EP69
Scyl2	0.0057	0.3795	0.3490	0.0170	12.02 ±0.09	12.43 ±0.09	12.25 ±0.09	12.02 ±0.11	G5E8J9 Q8CFE4
Snap91	0.0002	0.0004	0.0002	0.0005	11.08 ±0.14	12.15 ±0.08	11.12 ±0.07	11.11 ±0.11	A0A5F8MPL3 E9Q9A3 E9QLK9 E9QQ05 Q3TWS4 Q61548
Sumo3	0.0469	0.0006	0.8844	0.3197	11.07 ±0.20	11.30 ±0.07	11.82 ±0.05	11.56 ±0.04	G3UWI9 G3UWX9 G3UZ60 G3UZA7 P61957 Q9Z172
Uchl1	0.0298	0.0526	0.0279	0.9825	11.47 ±0.10	11.46 ±0.17	12.04 ±0.08	11.42 ±0.12	Q9R0P9
Ywhag	0.0374	0.2824	0.2158	0.0036	11.81 ±0.07	12.16 ±0.04	11.93 ±0.14	11.83 ±0.10	P61982
Zranb2	0.0040	0.0031	0.4253	0.0069	11.25 ±0.14	11.86 ±0.06	11.23 ±0.13	10.85 ±0.20	D3Z4U0 Q9R020

Table S5: Selected synaptic proteins

gene	interaction p-value	trisomy p-value	fluoxetine p-value	TW - TF p-value	$\pi_{TW/EW} \times \pi_{TF/TW}$	TW $\log_2$(quantity) mean ±SE	TF $\log_2$(quantity) mean ±SE	EW $\log_2$(quantity) mean ±SE	EF $\log_2$(quantity) mean ±SE	UniProt accession
Aak1	0.0348	0.0370	0.1554	0.0009	0.0565	10.36 ±0.12	11.13 ±0.03	10.37 ±0.15	10.20 ±0.34	Q3UHJ0
Ap2a1	0.0060	0.1708	0.0001	0.0010	-0.0414	10.92 ±0.08	11.55 ±0.07	11.04 ±0.08	11.23 ±0.03	P17426
Ap2a2	0.0478	0.0100	0.0001	0.0013	0.2007	11.03 ±0.14	11.89 ±0.07	10.93 ±0.11	11.30 ±0.12	P17427
Ap2b1	0.0440	0.5340	0.0045	0.0114	-0.1565	10.97 ±0.24	11.87 ±0.05	11.22 ±0.16	11.42 ±0.10	H3BKM0 Q9DBG3
Armc1	0.0468	0.6161	0.5349	0.1749	-0.5358	10.61 ±0.34	11.16 ±0.11	11.21 ±0.23	10.21 ±0.55	Q9D7A8
Bsn	0.2690	0.4287	0.0766	0.1384	-40.2529	12.44 ±1.75	9.45 ±0.11	10.53 ±0.80	9.78 ±0.18	O88737
Clta	0.0092	0.0146	0.0113	0.0002	-0.0158	12.08 ±0.11	13.24 ±0.09	12.13 ±0.22	12.11 ±0.28	B1AWD8 B1AWD9 O08585
Cltb	0.0027	0.0059	0.0005	0.0003	-0.0345	12.21 ±0.16	13.49 ±0.06	12.28 ±0.24	12.43 ±0.07	Q6IRU5
Cltc	0.0061	0.0028	0.0055	0.0004	0.4466	11.26 ±0.16	12.52 ±0.08	11.17 ±0.27	11.19 ±0.19	Q68FD5
Cntnap1	0.0325	0.2353	0.2666	0.0464	-0.0533	11.04 ±0.20	10.44 ±0.13	10.84 ±0.23	11.06 ±0.07	O54991
Commd2	0.0347	0.0370	0.0511	0.0206	-0.0026	12.53 ±0.16	13.41 ±0.23	12.54 ±0.16	12.50 ±0.21	Q8BXC6
Dbn1	0.1661	0.5069	0.9288	0.2508	-4.6948	11.84 ±0.56	10.52 ±0.87	11.17 ±0.85	12.34 ±1.02	A0A0R4J1E3 Q9QXS6
Dmxl2	0.0102	0.2529	0.0064	0.0088	-0.0749	11.17 ±0.10	11.94 ±0.18	11.39 ±0.13	11.42 ±0.01	B0V2P5 Q8BPN8
Echs1	0.0296	0.0198	0.0833	0.5588	0.0000	12.69 ±0.18	12.84 ±0.16	12.64 ±0.10	11.52 ±0.44	Q8BH95
Eif3c	0.0327	0.0386	0.2932	0.0414	-0.0008	10.31 ±0.19	10.86 ±0.10	10.35 ±0.15	8.88 ±0.80	Q8R1B4
Eif3e	0.0426	0.5831	0.1786	0.0286	-0.2729	11.20 ±0.12	11.85 ±0.19	11.50 ±0.11	11.35 ±0.24	P60229
Eif3h	0.0442	0.3793	0.6343	0.1114	-4.5465	4.94 ±0.33	6.81 ±0.95	7.10 ±0.89	5.90 ±0.27	Q91WK2
Eif3i	0.0493	0.3484	0.2591	0.0014	-0.0799	11.87 ±0.05	12.41 ±0.08	12.06 ±0.07	11.90 ±0.30	Q9QZD9
Eif3l	0.0038	0.1941	0.1644	0.0052	-1.5462	11.32 ±0.16	12.04 ±0.06	11.63 ±0.15	11.33 ±0.18	Q8QZY1
Exoc1	0.0147	0.0004	0.3166	0.0561	0.1826	11.74 ±0.14	12.16 ±0.12	11.52 ±0.09	11.32 ±0.09	Q5PPR2
Exoc3	0.0407	0.3249	0.0011	0.0003	-0.0707	11.01 ±0.05	11.59 ±0.06	11.13 ±0.13	11.30 ±0.09	Q6KAR6
Exoc8	0.0252	0.0003	0.4531	0.0593	0.0363	11.17 ±0.13	11.52 ±0.08	10.91 ±0.12	10.72 ±0.08	Q6PGF7
Fmr1	0.3420	0.1851	0.0425	0.0814	-223.2321	7.63 ±2.08	1.68 ±1.94	3.26 ±2.22	0.92 ±0.57	D3Z6U8
Gmfb	0.0356	0.7267	0.6840	0.1381	-0.0155	12.72 ±0.08	12.93 ±0.09	12.87 ±0.06	12.72 ±0.06	Q9CQI3
Grin2a	0.1391	0.5906	0.0943	0.1062	-15.5557	11.21 ±1.38	8.58 ±0.16	9.56 ±0.60	9.38 ±0.34	P35436
Hip1	0.0453	0.5261	0.0017	0.0072	-0.0943	11.18 ±0.18	12.03 ±0.11	11.39 ±0.14	11.64 ±0.09	Q8VD75
Itsn1	0.7058	0.0133	0.0318	0.0952	0.0006	12.08 ±0.15	12.45 ±0.11	11.74 ±0.08	12.02 ±0.17	E9Q0N0 Q9Z0R4
Lmtk3	0.0499	0.6674	0.2051	0.0583	-3.5248	10.72 ±0.55	8.57 ±0.74	9.66 ±0.61	10.17 ±0.52	A0A1B0GSR5
Myo5a	0.0420	0.9763	0.2288	0.0614	-18.6516	12.16 ±0.68	10.48 ±0.28	11.06 ±0.51	11.54 ±0.32	Q99104
Naxd	0.0232	0.0048	0.1028	0.0184	0.1349	12.22 ±0.17	13.01 ±0.18	12.07 ±0.10	11.92 ±0.25	J3QMM7
Nefl	0.1864	0.4782	0.1119	0.1316	-47.1671	9.22 ±2.75	4.40 ±0.23	5.92 ±1.36	5.44 ±0.31	P08551
Nrp1	0.7706	0.3256	0.4452	0.3784	-0.0008	10.68 ±0.21	9.09 ±1.66	8.74 ±1.40	8.03 ±1.95	P97333
Nrxn1	0.8209	0.1704	0.0442	0.1158	-1.2303	5.74 ±0.39	6.81 ±0.43	6.47 ±0.22	7.34 ±0.59	Q9CS84

Table S5: Selected synaptic proteins *(continued)*

gene	interaction p-value	trisomy p-value	fluoxetine p-value	TW - TF p-value	$\pi_{TW/EW} \times \pi_{TF/TW}$	TW $\log_2$(quantity) mean ±SE	TF $\log_2$(quantity) mean ±SE	EW $\log_2$(quantity) mean ±SE	EF $\log_2$(quantity) mean ±SE	UniProt accession
Ocrl	0.0043	0.0204	0.0011	0.0007	-0.0420	11.06 ±0.11	12.06 ±0.11	11.17 ±0.13	11.27 ±0.16	Q6NVF0
Park7	0.0160	0.2062	0.1912	0.3566	-0.2772	12.09 ±0.12	12.24 ±0.10	12.53 ±0.06	12.08 ±0.14	Q99LX0
Pclo	0.2402	0.5226	0.1009	0.1494	-21.2808	2.77 ±1.44	0.39 ±0.08	1.27 ±0.62	0.84 ±0.18	Q9QYX7
Pycr2	0.5462	0.1177	0.5657	0.4420	0.0197	10.94 ±0.14	10.68 ±0.28	11.16 ±0.14	11.17 ±0.25	Q922Q4
Rab5b	0.0998	0.2186	0.0157	0.0076	-4.3040	11.70 ±0.10	10.29 ±0.34	10.76 ±0.29	10.44 ±0.40	P61021
Rps21	0.0493	0.6809	0.1145	0.0015	-5.6964	13.55 ±0.09	14.55 ±0.16	14.01 ±0.03	13.88 ±0.48	Q9CQR2
Rps28	0.0040	0.8229	0.1137	0.0061	-2.0419	11.98 ±0.14	13.12 ±0.24	12.70 ±0.11	12.30 ±0.32	P62858
Rpsa	0.0411	0.2868	0.2716	0.0003	-3.0511	11.27 ±0.04	12.29 ±0.13	11.62 ±0.04	11.28 ±0.58	P14206
Sacm1l	0.0233	0.0038	0.7015	0.1415	0.0044	11.68 ±0.08	11.94 ±0.13	11.56 ±0.15	11.21 ±0.09	A0A5F8MPK9 Q9EP69
Scyl2	0.0057	0.3795	0.3490	0.0170	-0.3427	12.02 ±0.09	12.43 ±0.09	12.25 ±0.09	12.02 ±0.11	G5E8J9 Q8CFE4
Snap91	0.0002	0.0004	0.0002	0.0005	-0.0297	11.08 ±0.14	12.15 ±0.08	11.12 ±0.07	11.11 ±0.11	A0A5F8MPL3 E9Q9A3 E9QLK9 E9QQ05 Q3TWS4 Q61548
Snx9	0.4009	0.0411	0.9968	0.3801	-0.9590	10.11 ±0.09	8.72 ±1.46	5.06 ±1.87	6.46 ±2.14	Q91VH2
Syn3	0.1362	0.3196	0.9931	0.1666	-21.4623	8.85 ±0.20	6.17 ±1.69	4.45 ±2.10	7.10 ±1.96	Q8JZP2
Ube3a	0.0608	0.5047	0.0774	0.0191	-20.5864	8.36 ±1.50	2.13 ±1.27	6.21 ±1.15	6.42 ±2.14	O08759
Ywhag	0.0374	0.2824	0.2158	0.0036	-0.0390	11.81 ±0.07	12.16 ±0.04	11.93 ±0.14	11.83 ±0.10	P61982
Zfp365	0.9497	0.0229	0.4640	0.4272	-0.0886	13.40 ±0.20	13.61 ±0.14	14.09 ±0.19	14.27 ±0.41	Q8BG89

Table S6: GSEA of proteins ranked by PC 3 loading

Enriched Reactome pathway	FDR-adjusted p-value	Normalized enrichment score	Number of genes
Gluconeogenesis	0.00012	2.19541	18
Antigen processing: Ubiquitination & Proteasome degradation	0.00112	1.93194	56
CTLA4 inhibitory signaling	0.01276	1.87857	11
TCF dependent signaling in response to WNT	0.00758	1.85435	42
GPCR ligand binding	0.01522	1.84806	18
Signaling by GPCR	0.00299	1.83411	72
Metabolism	0.00001	1.80392	363
EPHB-mediated forward signaling	0.04378	1.71313	17
Innate Immune System	0.03797	1.43950	214
Transport of Mature mRNA derived from an Intron-Containing Transcript	0.04546	-1.78972	11
Membrane Trafficking	0.00001	-1.91791	169
Recycling pathway of L1	0.00299	-2.08569	23
Lysosome Vesicle Biogenesis	0.00050	-2.24093	10
MHC class II antigen presentation	0.00016	-2.24586	41
trans-Golgi Network Vesicle Budding	0.00045	-2.25416	23
Clathrin-mediated endocytosis	0.00001	-2.29878	56
Translation	0.00000	-2.31899	94
mRNA Splicing	0.00004	-2.34428	41
Formation of the ternary complex, and subsequently, the 43S complex	0.00001	-2.52513	34

Table S7: Genes of enriched pathways

Enriched Reactome pathway	Genes
Gluconeogenesis	Aldoa, Aldoc, Eno1, Eno2, Eno3, Gapdh, Got1, Got2, Gpi1, Mdh1, Mdh2, Pgam1, Pgk1, Slc25a1, Slc25a10, Slc25a11, Slc25a12, Tpi1
Antigen processing: Ubiquitination & Proteasome degradation	Cdc23, Cul3, Cul5, Fbxl16, Fbxo2, Fbxo41, Fbxo6, Hectd3, Hecw2, Herc1, Huwe1, Itch, Lmo7, Mib2, Nedd4, Npepps, Psma1, Psma2, Psma3, Psma4, Psma5, Psma6, Psmb1, Psmb2, Psmb4, Psmb5, Psmb6, Psmc1, Psmc4, Psmc6, Psmd1, Psmd11, Psmd13, Psmd14, Psmd2, Psmd3, Psmd4, Psmd5, Psmd6, Psmd7, Psmd8, Psmd9, Rps27a, Thop1, Tpp2, Uba1, Uba5, Ube2m, Ube2n, Ube2o, Ube2v1, Ube2v2, Ube3a, Ubr4, Ufl1, Wwp1
CTLA4 inhibitory signaling	Fyn, Lyn, Ppp2ca, Ppp2cb, Ppp2r1a, Ppp2r5a, Ppp2r5b, Ppp2r5c, Ptpn11, Src, Yes1
TCF dependent signaling in response to WNT	Csnk1e, Ctbp1, Ctnnb1, Cul3, Gsk3b, Hecw1, Ppp2ca, Ppp2cb, Ppp2r1a, Ppp2r5a, Ppp2r5b, Ppp2r5c, Psma1, Psma2, Psma3, Psma4, Psma5, Psma6, Psmb1, Psmb2, Psmb4, Psmb5, Psmb6, Psmc1, Psmc4, Psmc6, Psmd1, Psmd11, Psmd13, Psmd14, Psmd2, Psmd3, Psmd4, Psmd5, Psmd6, Psmd7, Psmd8, Psmd9, Rps27a, Usp8, Xpo1, Ywhaz
GPCR ligand binding	Cck, Gabbr1, Gabbr2, Gnas, Gnb5, Gng10, Gng13, Gng2, Gng5, Grm1, Grm2, Grm3, Grm5, Grm7, Nln, Plppr3, Rln3, Sst
Signaling by GPCR	Abhd12, Abr, Adcy1, Adcy5, Adcy9, Arhgef26, Arhgef7, Calm1, Cck, Cdc42, Cdk5, Dagla, Dgkb, Dgkd, Dgki, Dgkz, Gabbr1, Gabbr2, Gna11, Gna13, Gnai1, Gnai2, Gnai3, Gnal, Gnaq, Gnas, Gnat3, Gnaz, Gnb5, Gng10, Gng13, Gng2, Gng5, Grk2, Grm1, Grm2, Grm3, Grm5, Grm7, Hras, Itpr1, Itsn1, Kras, Mapk1, Mapk3, Ngef, Nln, Pdpk1, Pik3r1, Plcb1, Plppr3, Plxnb1, Ppp1ca, Ppp1r1b, Ppp2ca, Ppp2cb, Ppp2r1a, Prex1, Prkacb, Prkar1a, Prkar2b, Prkca, Prkcg, Rgs14, Rgs17, Rgs19, Rgs7, Rhob, Rln3, Sos1, Src, Sst

Table S7: Genes of enriched pathways *(continued)*

Enriched Reactome pathway	Genes
Metabolism	Aacs, Abcg2, Acaa2, Acaca, Acad11, Acad8, Acad9, Acadl, Acadm, Acads, Acadsb, Acan, Acat1, Acly, Aco2, Acot11, Acot13, Acot7, Acot9, Acox1, Acsbg1, Acsl6, Acss1, Adcy5, Adh5, Ado, Adss, Agk, Agl, Ahcyl, Aip, Ak1, Ak2, Ak4, Ak5, Akr1a1, Akr1b3, Akr7a5, Alb, Aldh18a1, Aldh1a1, Aldh1b1, Aldh1l1, Aldh2, Aldh3b1, Aldh6a1, Aldh7a1, Aldoa, Aldoc, Ampd2, Apoa1, Apoe, Arsb, Asrgl1, Ass1, Atic, Atp5b, Atp5d, Atp5e, Atp5h, Atp5j2, Atp5k, Atp5l, Atp5o, Atp5pb, ATP8, Auh, Bcan, Bdh1, Blvrb, Bphl, Bpnt1, Cad, Calm1, Car2, Cbr1, Cbr3, Cbr4, Ckb, Ckmt1, Cmpk1, Cndp2, Coasy, COX2, Cox4i1, Cox5a, Cox6b1, Cox7a2l, Cox7c, Cpne1, Cpne6, Crat, Crym, Cs, Cspg4, Cspg5, Cyb5a, Cyb5b, Cyc1, Cycs, Cyp46a1, Dbt, Ddah1, Ddc, Decr1, Dlat, Dld, Dlst, Dmac2l, Dtymk, Echs1, Eci1, Eno1, Eno2, Eno3, Enpp6, Esyt2, Etfa, Etfb, Etfdh, Fabp3, Fasn, Fdxr, Fh1, G6pdx, Gapdh, Gart, Gcdh, Gda, Ggt7, Gk, Glo1, Gls, Glud1, Glul, Gmps, Gna11, Gnai1, Gnai2, Gnaq, Gnas, Gnb5, Gng10, Gng13, Gng2, Gng5, Got1, Got2, Gpc1, Gpc4, Gpd1l, Gpd2, Gphn, Gpi1, Gpx1, Gstm5, Gstp1, Gstz1, Hacd3, Hadh, Hadha, Hadhb, Hagh, Hexa, Hexb, Hibch, Hmgcl, Hprt, Hsd17b11, Hsd17b4, Hsd17b8, Hsp90aa1, Hsp90ab1, Idh1, Idh2, Idh3b, Idh3g, Inpp5j, Isyna1, Itpka, Itpr1, Ivd, Kcnb1, Kpnb1, Ldha, Lrp1, Lrpprc, Lum, Man2b1, Maoa, Maob, Marcks, Mccc1, Mccc2, Mdh1, Mdh2, Me1, Me2, Me3, Mecr, Mlycd, Mmaa, Mmab, Mmut, Mtarc2, Mthfd1, Mthfd1l, Mtmr12, Naxd, Ncan, Ndufa10, Ndufa12, Ndufa13, Ndufa2, Ndufa4, Ndufa5, Ndufa6, Ndufa7, Ndufa8, Ndufaf3, Ndufaf4, Ndufb10, Ndufb11, Ndufb3, Ndufb4, Ndufb8, Ndufb9, Ndufc2, Ndufs1, Ndufs3, Ndufs4, Ndufs7, Ndufs8, Ndufv2, Ndufv3, Nfs1, Nme1, Nme3, Nmral1, Nudt5, Nup93, Nup98, Oat, Ocrl, Ogdh, Osbp, Osbpl8, Oxct1, Paics, Pcca, Pccb, Pcyt2, Pdha1, Pdhb, Pdhx, Pdk1, Pdk2, Pdk3, Pdxk, Pfkl, Pfkm, Pfkp, Pgam1, Pgd, Pgk1, Pgls, Pgm1, Pgm2l1, Phgdh, Pi4ka, Pik3r1, Pik3r4, Pip4k2a, Pip4k2b, Pip4k2c, Pip5k1c, Pitpnm1, Pitpnm2, Pkm, Plcb1, Plcg1, Plch2, Plpp3, Pnpla8, Ppa1, Ppip5k1, Ppt1, Prkacb, Prkar1a, Prkar2b, Prkca, Prps2, Psat1, Psma1, Psma2, Psma3, Psma4, Psma5, Psma6, Psmb1, Psmb2, Psmb4, Psmb5, Psmb6, Psmc1, Psmc4, Psmc6, Psmd1, Psmd11, Psmd13, Psmd14, Psmd2, Psmd3, Psmd4, Psmd5, Psmd6, Psmd7, Psmd8, Psmd9, Ptgds, Pycr2, Pycrl, Pygm, Rab14, Rab4a, Ran, Ranbp2, Rapgef4, Rida, Sbf1, Sco2, Scp2, Sdha, Sdhb, Shmt2, Slc25a1, Slc25a10, Slc25a11, Slc25a12, Slc25a19, Slc25a20, Slc2a1, Slc2a3, Slc3a2, Slc6a11, Sphk2, Srm, Sucla2, Suclg1, Them4, Tkfc, Tkt, Tpi1, Tpr, Tst, Txnrd1, Uck1, Ugp2, Uqcrc1, Uqcrc2, Uqcrfs1, Uqcrq, Vdac1
EPHB-mediated forward signaling	Actg1, Actr2, Actr3, Arpc1a, Arpc2, Arpc3, Arpc4, Arpc5, Cdc42, Fyn, Grin2b, Hras, Itsn1, Lyn, Rac1, Src, Yes1
Innate Immune System	Abi1, Abi2, Acly, Actg1, Actr10, Actr1b, Actr2, Actr3, Adam10, Agl, Aldh3b1, Aldoa, Aldoc, Anpep, Ap1m1, Ap2a2, Arpc1a, Arpc2, Arpc3, Arpc4, Arpc5, Arsb, Atad3a, Atp6ap2, Atp6v0d1, Atp6v1a, Atp6v1b2, Atp6v1c1, Atp6v1d, Atp6v1e1, Atp6v1g1, Atp6v1g2, Atp6v1h, Atp8a1, Baiap2, Brk1, C1qc, Cab39, Calm1, Cand1, Cap1, Capza2, Cat, Cct2, Cct8, Cdc42, Ckap4, Clu, Commd3, Commd9, Cotl1, Cpne1, Crk, Cst3, Ctnnb1, Cyfip1, Cyfip2, Cyld, Ddost, Dhx36, Dhx9, Dnajc3, Dnm1, Dnm3, Dock1, Dsp, Dync1h1, Dync1li1, Eea1, Eef1a1, Eef2, Elmo1, Elmo2, Erp44, Fga, Frmpd3, Fyn, Gdi2, Gpi1, Gsn, Gstp1, Hbb-bs, Hck, Hexb, Hmgb1, Hras, Hsp90aa1, Hsp90ab1, Hsp90b1, Hspa1b, Hspa8, Huwe1, Idh1, Ilf2, Iqgap2, Ist1, Itch, Jup, Kpnb1, Kras, Lamtor1, Lyn, Man2b1, Map2k4, Map2k6, Mapk1, Mapk3, Mlec, Mvp, Myh9, Myo1c, Myo5a, Nck1, Nckap1, Nckipsd, Ncstn, Ndufc2, Pa2g4, Padi2, Pak2, Pak3, Pdap1, Pdpk1, Pdxk, Pfkl, Pgam1, Pgm1, Pgrmc1, Pik3r1, Pik3r4, Pin1, Pkm, Plcg1, Pld3, Ppia, Ppp2ca, Ppp2cb, Ppp2r1a, Ppp3ca, Ppp3cb, Prdx6, Prkacb, Prss1, Psma1, Psma2, Psma3, Psma4, Psma5, Psma6, Psmb1, Psmb2, Psmb4, Psmb5, Psmb6, Psmc1, Psmc4, Psmc6, Psmd1, Psmd11, Psmd13, Psmd14, Psmd2, Psmd3, Psmd4, Psmd5, Psmd6, Psmd7, Psmd8, Psmd9, Ptpn11, Ptprn2, Pygb, Rab10, Rab14, Rab27a, Rab37, Rab3a, Rab4b, Rab5b, Rab5c, Rab6a, Rab7, Rac1, Rbsn, Rhog, Rps27a, Sarm1, Sdcbp, Serpina1c, Serpinb1a, Sh3glb2, Sirpa, Slc2a3, Snap25, Sos1, Src, Svip, Syngr1, Tomm70a, Traf3, Tubb4b, Tubb5, Ube2m, Ube2n, Ube2v1, Ubr4, Usp14, Vat1, Vcl, Vcp, Wasf1, Wasf2, Wasf3, Yes1

Table S7: Genes of enriched pathways *(continued)*

Enriched Reactome pathway	Genes
Transport of Mature mRNA derived from an Intron-Containing Transcript	Alyref, Ddx39b, Eif4a3, Nup93, Nup98, Poldip3, Ranbp2, Sarnp, Srsf2, Srsf3, Tpr
Membrane Trafficking	Aak1, Actg1, Actr10, Actr1a, Actr2, Actr3, Amph, Ank1, Ap1b1, Ap1g1, Ap1g2, Ap1m1, Ap1s1, Ap2a1, Ap2a2, Ap2m1, Ap2s1, Arcn1, Arf4, Arf5, Arfgap1, Arfgap2, Arpc1a, Arpc2, Arpc3, Arpc4, Arpc5, Bin1, Capza2, Capzb, Ccz1, Chmp2a, Chmp2b, Chmp4b, Chmp6, Cltb, Cltc, Copb2, Cope, Copg2, Cops2, Cops4, Cops7a, Cops8, Dctn1, Dctn2, Dctn3, Dctn4, Dennd1a, Dnajc6, Dnm1, Dnm3, Dync1h1, Dync1i1, Dync1i2, Dync1li1, Dync1li2, Epn2, Eps15l1, Fcho2, Gabarapl2, Gak, Galnt2, Gapvd1, Gdi1, Gdi2, Gja1, Golgb1, Gria1, Grk2, Hip1, Hip1r, Hspa8, Itsn1, Kif21a, Kif3a, Kif5b, Lman1, Madd, Map1lc3b, Mia3, Myo6, Napa, Napb, Necap1, Nsf, Ocrl, Pacsin1, Pacsin2, Pacsin3, Pafah1b1, Pafah1b3, Picalm, Pip5k1c, Preb, Pum1, Rab10, Rab13, Rab14, Rab1a, Rab27a, Rab27b, Rab33b, Rab35, Rab3a, Rab3gap2, Rab4a, Rab5b, Rab5c, Rab6a, Rab6b, Rab7, Rab8a, Rab8b, Rabgap1, Reps1, Reps2, Rps27a, Sbf1, Sbf2, Scfd1, Sec16a, Sec22b, Sec24b, Sec24c, Sec31a, Serpina1c, Sh3gl1, Sh3gl3, Sh3kbp1, Snx2, Snx5, Snx9, Spta1, Sptbn1, Sptbn2, Sptbn4, Src, Syt1, Syt11, Syt2, Tbc1d10b, Tbc1d24, Tfrc, Tjp1, Tmed10, Trappc11, Trappc3, Trappc4, Trappc5, Trappc6b, Trappc9, Trf, Tuba1b, Tuba8, Tubb1, Tubb2a, Tubb2b, Tubb3, Tubb4a, Tubb4b, Uso1, Vps45, Vps4a, Vps4b, Vps51, Vps52, Vps53, Ywhae
Recycling pathway of L1	Actg1, Ap2a1, Ap2a2, Ap2m1, Ap2s1, Cltc, Dnm1, Dnm3, Dpysl2, Ezr, Mapk1, Msn, Numb, Rdx, Src, Tuba1b, Tuba8, Tubb1, Tubb2a, Tubb2b, Tubb3, Tubb4a, Tubb4b
Lysosome Vesicle Biogenesis	Ap1b1, Ap1g1, Ap1g2, Ap1m1, Ap1s1, Chmp2a, Cltb, Cltc, Dnajc6, Hspa8
MHC class II antigen presentation	Actr10, Actr1a, Actr1b, Ap1b1, Ap1g1, Ap1m1, Ap1s1, Ap2a1, Ap2a2, Ap2m1, Ap2s1, Canx, Capza2, Capzb, Cltc, Dctn1, Dctn2, Dctn3, Dctn4, Dnm1, Dnm3, Dync1h1, Dync1i1, Dync1i2, Dync1li1, Dync1li2, Kif3a, Kif5b, Rab7, Sec24b, Sec24c, Sec31a, Sptbn2, Tuba1b, Tuba8, Tubb1, Tubb2a, Tubb2b, Tubb3, Tubb4a, Tubb4b
trans-Golgi Network Vesicle Budding	Ap1b1, Ap1g1, Ap1g2, Ap1m1, Ap1s1, Chmp2a, Cltb, Cltc, Dnajc6, Gak, Golgb1, Hip1r, Hspa8, Napa, Necap1, Ocrl, Picalm, Pum1, Rab5c, Snx2, Snx5, Snx9, Tfrc
Clathrin-mediated endocytosis	Aak1, Actg1, Actr2, Actr3, Amph, Ap2a1, Ap2a2, Ap2m1, Ap2s1, Arfgap1, Arpc1a, Arpc2, Arpc3, Arpc4, Arpc5, Bin1, Cltb, Cltc, Cops2, Cops4, Cops7a, Cops8, Dnajc6, Dnm1, Dnm3, Epn2, Eps15l1, Fcho2, Gak, Gapvd1, Grk2, Hip1, Hip1r, Hspa8, Itsn1, Necap1, Ocrl, Pacsin1, Pacsin2, Pacsin3, Picalm, Pip5k1c, Rab5b, Rab5c, Reps1, Reps2, Rps27a, Sh3gl1, Sh3gl3, Sh3kbp1, Snx9, Syt1, Syt11, Syt2, Tfrc, Trf
Translation	Dap3, Eef1a1, Eef1b2, Eef1d, Eef1g, Eef2, Eif2s1, Eif2s2, Eif2s3x, Eif3a, Eif3b, Eif3c, Eif3d, Eif3e, Eif3f, Eif3g, Eif3h, Eif3i, Eif3l, Eif4a1, Eif4a2, Eif4b, Eif4e, Eif4g1, Eif4h, Mrpl1, Mrpl12, Mrpl19, Mrpl27, Mrpl37, Mrpl39, Mrpl45, Mrpl53, Mrps28, Mrps36, Mrrf, Pabpc1, Ppa1, Rpl11, Rpl12, Rpl13, Rpl14, Rpl15, Rpl18, Rpl18a, Rpl19, Rpl21, Rpl22, Rpl22l1, Rpl23, Rpl23a, Rpl24, Rpl26, Rpl27, Rpl27a, Rpl28, Rpl29, Rpl3, Rpl30, Rpl31, Rpl34, Rpl35, Rpl35a, Rpl37, Rpl37a, Rpl4, Rpl5, Rpl6, Rpl7, Rpl8, Rplp0, Rplp2, Rps10, Rps11, Rps13, Rps14, Rps16, Rps17, Rps19, Rps2, Rps21, Rps23, Rps25, Rps28, Rps3, Rps3a1, Rps4x, Rps5, Rps6, Rps7, Rps8, Rps9, Rpsa, Srp54a
mRNA Splicing	Alyref, Bcas2, Cdc5l, Cherp, Ddx39b, Dhx15, Dhx9, Eif4a3, Hnrnpa1, Hnrnpa2b1, Hnrnpc, Hnrnpf, Hnrnph1, Hnrnph2, Hnrnpk, Hnrnpr, Hnrnpu, Hspa8, Ik, Lsm3, Lsm6, Pcbp1, Pnn, Prpf19, Prpf8, Sf3a1, Sf3a3, Sf3b2, Sf3b3, Snrnp70, Snrpa1, Snrpd1, Snrpd3, Snrpe, Snu13, Srrm2, Srsf2, Srsf3, Tcerg1, Wbp11, Zmat2
Formation of the ternary complex, and subsequently, the 43S complex	Eif2s1, Eif2s2, Eif2s3x, Eif3a, Eif3b, Eif3c, Eif3d, Eif3e, Eif3f, Eif3g, Eif3h, Eif3i, Eif3l, Rps10, Rps11, Rps13, Rps14, Rps16, Rps17, Rps19, Rps2, Rps21, Rps23, Rps25, Rps28, Rps3, Rps3a1, Rps4x, Rps5, Rps6, Rps7, Rps8, Rps9, Rpsa

Table S8: GSEA enriched Reactome pathways for proteins ranked by π(TF/EW)

Enriched pathway	FDR-adjusted p-value	Normalized enrichment score	Number of genes
Clathrin-mediated endocytosis	0.0002	2.1691	59
Golgi Associated Vesicle Biogenesis	0.0019	2.1157	22
Signaling by NTRKs	0.0149	1.9591	23
Protein-protein interactions at synapses	0.0031	-1.4830	44
Unblocking of NMDA receptors, glutamate binding and activation	0.0049	-1.5187	15

Table S9: Genes of enriched Reactome pathways for GSEA of proteins ranked by π(TF/EW)

Enriched pathway	Genes
Clathrin-mediated endocytosis	Aak1, Actg1, Actr2, Actr3, Agfg1, Amph, Ap2a1, Ap2a2, Ap2b1, Ap2m1, Ap2s1, Arfgap1, Arpc1a, Arpc2, Arpc3, Arpc4, Arpc5, Arrb1, Cltb, Cltc, Cops2, Cops4, Cops6, Cops7a, Cops8, Dnajc6, Dnm1, Dnm3, Epn2, Eps15l1, Fcho2, Gak, Gapvd1, Grb2, Grk2, Hip1, Hip1r, Hspa8, Itsn1, Necap1, Ocrl, Pacsin1, Pacsin2, Pacsin3, Picalm, Pip5k1c, Rab5b, Reps1, Reps2, Rps27a, Sh3gl2, Sh3gl3, Sh3kbp1, Snap91, Snx9, Syt1, Syt11, Tfrc, Vamp2
Golgi Associated Vesicle Biogenesis	Ap1g1, Ap1m1, Ap1s1, Arrb1, Cltc, Dnajc6, Gak, Golgb1, Hip1r, Hspa8, Napa, Necap1, Ocrl, Picalm, Pum1, Sh3d19, Sh3gl2, Snx2, Snx5, Snx9, Tfrc, Vamp2
Signaling by NTRKs	Ap2a1, Ap2a2, Ap2b1, Ap2m1, Ap2s1, Cltc, Crk, Fyn, Grb2, Kras, Map2k1, Mapk1, Mapk3, Ntrk3, Pik3r1, Plcg1, Ppp2ca, Ppp2r1a, Rac1, Sh3gl2, Sos1, Src, Ywhab
Protein-protein interactions at synapses	Cask, Dlg1, Dlg2, Dlg3, Dlg4, Dlgap1, Dlgap2, Dlgap3, Dlgap4, Epb41, Epb41l2, Epb41l3, Flot1, Flot2, Gria1, Gria4, Grin1, Grin2a, Grin2b, Grm1, Grm5, Homer1, Homer2, Homer3, Lrfn4, Lrrc4b, Lrrtm1, Lrrtm4, Nlgn2, Nlgn3, Nrxn1, Nrxn3, Ntrk3, Ppfia1, Ppfia2, Ppfia3, Ppfia4, Ptprd, Ptprs, Rtn3, Sh3glb2, Shank1, Shank2, Shank3
Unblocking of NMDA receptors, glutamate binding and activation	Calm2, Camk2a, Camk2b, Camk2d, Dlg1, Dlg2, Dlg3, Dlg4, Gria1, Gria4, Grin1, Grin2a, Grin2b, Lrrc7, Nefl

Table S10: GSEA enriched Reactome pathways for proteins ranked by π(TW/EW)

Enriched pathway	FDR-adjusted p-value	Normalized enrichment score	Number of genes
Metabolism of amino acids and derivatives	0.0351	-1.7100	81
Extracellular matrix organization	0.0363	-1.9221	21
Chaperonin-mediated protein folding	0.0351	-1.9759	20
GPCR ligand binding	0.0351	-1.9915	21

Table S11: Genes of enriched Reactome pathways for GSEA of proteins ranked by π(TW/EW)

Enriched pathway	Genes
Metabolism of amino acids and derivatives	Acad8, Acadsb, Acat1, Ado, Ahcy, Aldh18a1, Aldh6a1, Aldh7a1, Asrgl1, Ass1, Auh, Bckdk, Ckb, Ckmt1, Crym, Dbt, Ddc, Dlat, Dld, Dlst, Echs1, Gcdh, Gls, Glud1, Glul, Got1, Got2, Gstz1, Hibch, Ivd, Mccc1, Mccc2, Mpst, Nmral1, Oat, Ogdh, Pdha1, Pdhb, Pdhx, Phgdh, Psat1, Psma1, Psma2, Psma3, Psma4, Psma5, Psma6, Psma7, Psmb1, Psmb2, Psmb4, Psmb5, Psmb6, Psmc1, Psmc2, Psmc3, Psmc4, Psmc5, Psmc6, Psmd1, Psmd11, Psmd13, Psmd14, Psmd2, Psmd3, Psmd4, Psmd5, Psmd6, Psmd7, Psmd8, Psmd9, Pycr2, Qdpr, Rida, Slc25a10, Slc25a12, Slc3a2, Slc6a11, Srm, Tst, Txnrd1
Extracellular matrix organization	Acan, Adam10, App, Bcan, Capn5, Cd47, Ctsd, Fga, Hapln1, Icam5, Jam3, Lum, Matn4, Ncan, P4hb, Plg, Prkca, Prss1, Serpinh1, Tnc, Tnr
Chaperonin-mediated protein folding	Cct2, Cct3, Cct4, Cct5, Cct6a, Cct7, Cct8, Gna11, Gnaq, Gnb2, Gnb5, Gng10, Gng12, Gng13, Gng2, Gng5, Pdcl, Rgs6, Rgs7, Tcp1
GPCR ligand binding	App, Cck, Gabbr1, Gabbr2, Gnb2, Gnb5, Gng10, Gng12, Gng13, Gng2, Gng5, Grm1, Grm2, Grm3, Grm5, Grm7, Nln, Plppr3, Plppr4, Rln3, Sst

Table S12: GSEA enriched Reactome pathways for proteins ranked by π(EF/EW)

Enriched pathway	FDR-adjusted p-value	Normalized enrichment score	Number of genes
Metabolism of RNA	0.0404	-1.6270	173
HSF1-dependent transactivation	0.0404	-1.6292	11
Major pathway of rRNA processing in the nucleolus and cytosol	0.0404	-1.7048	61

Table S13: Genes of enriched Reactome pathways for GSEA of proteins ranked by π(EF/EW)

Enriched pathway	Genes
Metabolism of RNA	Alyref, Bcas2, Cdc5l, Cherp, Cnot1, Cnot3, Cnot9, Cpsf7, Csnk1e, Cstf2, Ddx39b, Ddx46, Ddx6, Dhx15, Dhx9, Edc4, Eif4a2, Eif4a3, Eif4b, Eif4e, Eif4g1, Elavl1, Gemin5, Heatr1, Hnrnpa1, Hnrnpa2b1, Hnrnpa3, Hnrnpc, Hnrnpd, Hnrnpf, Hnrnph1, Hnrnpk, Hnrnpl, Hnrnpu, Hspa1b, Hspa8, Hspb1, Ik, Khsrp, Lsm3, Lsm6, Lsm7, Ncl, Nop56, Nup93, Nup98, Pabpc1, Pcbp1, Pnn, Poldip3, Polr2a, Ppp2ca, Ppp2r1a, Ppp2r2a, Prkca, Prpf19, Prpf8, Psma1, Psma2, Psma3, Psma4, Psma5, Psma6, Psma7, Psmb1, Psmb2, Psmb4, Psmb5, Psmb6, Psmc1, Psmc2, Psmc3, Psmc4, Psmc5, Psmc6, Psmd1, Psmd11, Psmd13, Psmd14, Psmd2, Psmd3, Psmd4, Psmd5, Psmd6, Psmd7, Psmd8, Psmd9, Ranbp2, Rpl11, Rpl12, Rpl13, Rpl13a, Rpl14, Rpl15, Rpl18, Rpl18a, Rpl19, Rpl21, Rpl22, Rpl22l1, Rpl23, Rpl23a, Rpl24, Rpl26, Rpl27, Rpl27a, Rpl28, Rpl29, Rpl3, Rpl30, Rpl31, Rpl34, Rpl35, Rpl35a, Rpl37, Rpl37a, Rpl4, Rpl5, Rpl6, Rpl7, Rpl8, Rplp0, Rplp2, Rps11, Rps13, Rps14, Rps15a, Rps16, Rps17, Rps19, Rps2, Rps21, Rps23, Rps25, Rps27a, Rps28, Rps3, Rps4x, Rps5, Rps6, Rps7, Rps8, Rps9, Rpsa, Sarnp, Set, Sf3a1, Sf3a3, Sf3b2, Sf3b3, Snrnp70, Snrpa, Snrpa1, Snrpd1, Snrpd3, Snrpe, Snu13, Srrm2, Srsf1, Srsf2, Srsf3, Srsf7, Tcerg1, Tnks1bp1, Tnpo1, Tpr, Upf1, Wbp11, Xpo1, Xrn2, Ywhab, Ywhaz, Zmat2
HSF1-dependent transactivation	Camk2a, Camk2b, Camk2d, Cryab, Dnajb1, Hsp90aa1, Hsp90ab1, Hspa1b, Hspa8, Mtor, Ptges3
Major pathway of rRNA processing in the nucleolus and cytosol	Csnk1e, Heatr1, Ncl, Nop56, Rpl11, Rpl12, Rpl13, Rpl13a, Rpl14, Rpl15, Rpl18, Rpl18a, Rpl19, Rpl21, Rpl22, Rpl22l1, Rpl23, Rpl23a, Rpl24, Rpl26, Rpl27, Rpl27a, Rpl28, Rpl29, Rpl3, Rpl30, Rpl31, Rpl34, Rpl35, Rpl35a, Rpl37, Rpl37a, Rpl4, Rpl5, Rpl6, Rpl7, Rpl8, Rplp0, Rplp2, Rps11, Rps13, Rps14, Rps15a, Rps16, Rps17, Rps19, Rps2, Rps21, Rps23, Rps25, Rps28, Rps3, Rps4x, Rps5, Rps6, Rps7, Rps8, Rps9, Rpsa, Snu13, Xrn2
